# Refactoring pikromycin synthase for the modular biosynthesis of macrolide antibiotics in *E. coli*

**DOI:** 10.1038/s44160-026-01108-2

**Published:** 2026-06-29

**Authors:** Takeshi Miyazawa, Ramesh Bista, Adrian T. Keatinge-Clay

**Affiliations:** Department of Molecular Biosciences, The University of Texas at Austin, Austin, TX USA

**Keywords:** Biocatalysis, Multienzyme complexes, Multienzyme complexes, Drug discovery and development, Synthetic biology

## Abstract

While engineering modular polyketide synthases (PKSs) using the recently updated module boundary has yielded libraries of triketide–pentaketides, this strategy has not yet been applied to the combinatorial biosynthesis of macrolactones or macrolide antibiotics. Here we developed a two-plasmid system for the construction and expression of PKSs and used it to obtain a refactored pikromycin PKS in *Escherichia* *coli* that produces 49 mg of narbonolide per litre of culture. The replacement, insertion, deletion and mutagenesis of modules enabled access to hexaketide, heptaketide and octaketide derivatives. Supplying enzymes for desosamine biosynthesis and transfer enabled production of narbomycin, pikromycin, YC-17, methymycin and five derivatives thereof. By using native docking domains, knocking out pathways competing with desosamine biosynthesis and supplying the editing thioesterase PikAV, the titre of narbomycin was boosted 58-fold to 37 mg l^−1^. The second and third modules of the refactored pikromycin synthase were swapped with others to yield three new narbomycin analogues, investigate structure–activity relationships and demonstrate how libraries of macrolide antibiotics can be readily accessed.

**Figure:**
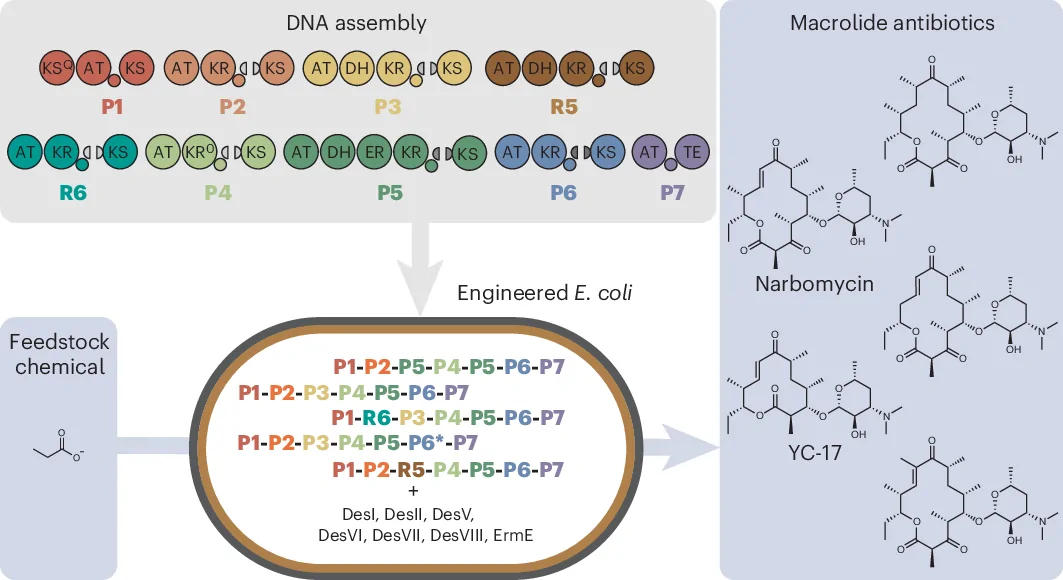

## Abstract (web summary)

A biosynthetic platform is developed to rapidly construct large, engineered polyketide synthases using the updated module boundary. This system produces a library of macrolide antibiotics in *Escherichia coli*.

## Main

Antimicrobial resistance is an ever-increasing threat to human health. As a consequence, there is a growing need to discover and develop new antibiotics^1^. Unfortunately, the pace of both discovering new antibiotics and developing antibiotics through medicinal chemistry has slowed^2^. Modular synthesis, through which diversity is created from relatively simple building blocks, can accelerate these processes^3^. A modular chemical synthesis recently yielded a library of >300 macrolide antibiotic derivatives, 2 of which displayed superior potencies compared with those in clinical use^4^.

The modular syntheses of macrolide antibiotics, such as erythromycin, pikromycin and tylosin, were first conducted by polyketide synthase (PKS) assembly lines within actinomyces bacteria^5–8^. These assembly lines are themselves modular, where each module (updated definition used^9,10^) adds one ketide monomer to a growing polyketide chain. They minimally contain three domains: an acyltransferase (AT), an acyl carrier protein (ACP) and a ketosynthase (KS). During the catalytic cycle of a module, ACP acquires the polyketide chain from the KS of the upstream module through an α-carboxyacyl extender unit obtained from AT, shuttles the extended chain to optional processing enzymes, such as ketoreductase, dehydratase and enoylreductase domains, and to KS, which acquires properly processed chains through transacylation. The most downstream domain of the assembly line is most commonly a thioesterase (TE) that releases the polyketide chain through cyclization or hydrolysis.

Most efforts to engineer PKSs have been based on the traditional module boundary and have not been successful^10^. This boundary was defined immediately upstream of KS when the genes encoding the erythromycin PKS were sequenced in 1990, in analogy with the mammalian fatty acid synthase, an iterative PKS in which KS is the most upstream domain^11,12^. In 2017, genome-mining of modular PKSs that produce related aminopolyols helped reveal that the KSs of modular PKSs evolutionarily comigrate with processing domains upstream of them and that the module boundary is actually immediately downstream of KS^9,10^. Indeed, synthases engineered with the updated module boundary consistently outperform synthases engineered with the traditional module boundary^13–15^. Our laboratory recently developed a BioBricks-like platform to rapidly assemble synthases on a single expression plasmid through the ligation of DNA fragments encoding updated modules. We used it to combinatorially assemble the modules of the pikromycin PKS (*Streptomyces venezuelae* ATCC 15439) into 5 triketide, 25 tetraketide and 125 pentaketide synthases and assess their function^16^.

**Fig. 1:**
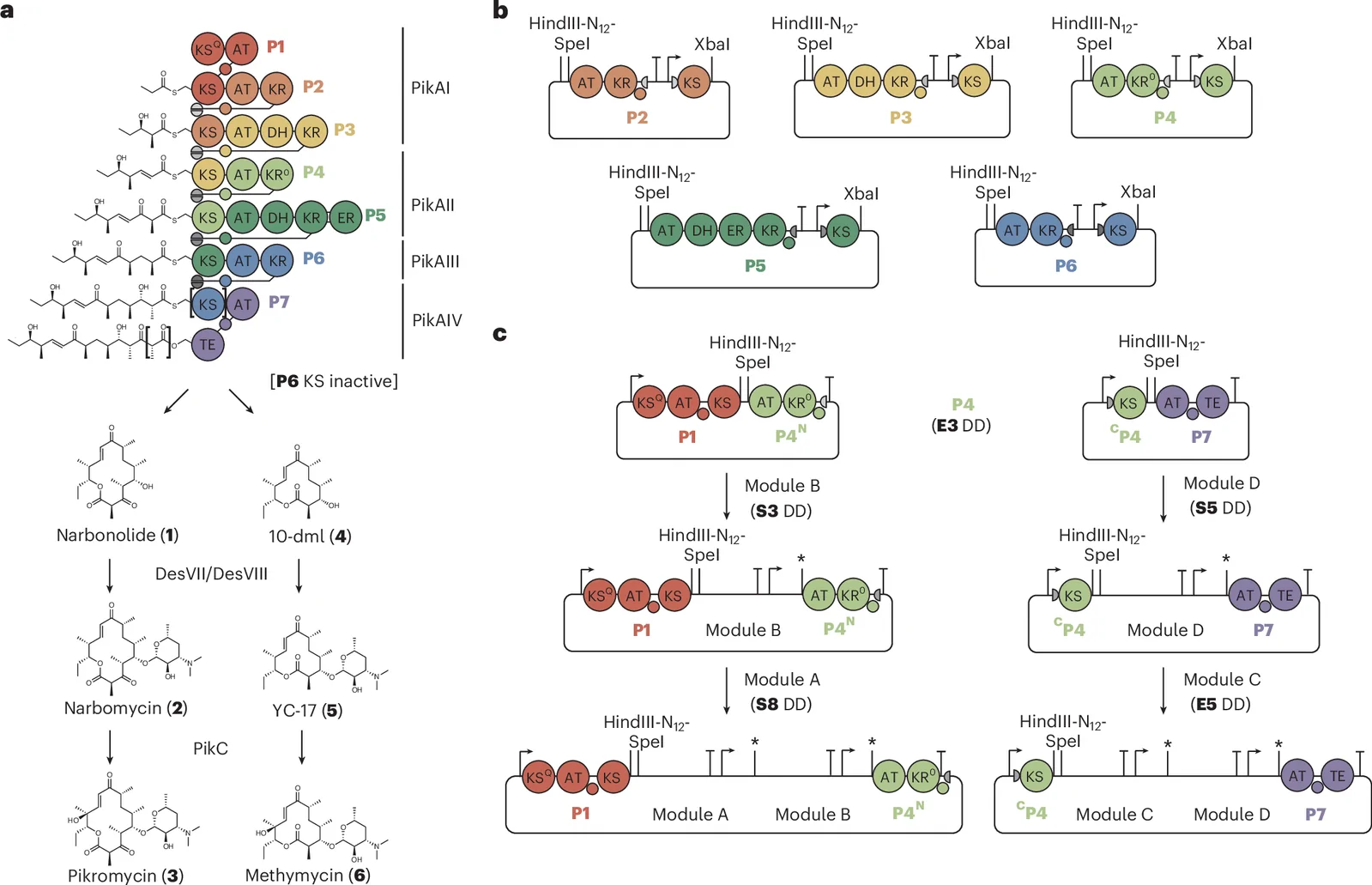
Pikromycin PKS and two-plasmid platform for constructing engineered PKSs. **a**, Pikromycin/methymycin biosynthetic pathway (PKS coloured by updated modules). **b**, Construction of BioBricks-like units encoding pikromycin modules. T7 promoters and terminators are shown. **c**, The two-plasmid system enables the BioBricks-like assembly of PKSs such as **P1**-**A**-**B**-**P4**-**C**-**D**-**P7**. Docking domains are specific for each position in the constructed synthase. The ligation of HindIII–XbaI fragments into the HindIII-N_12_-SpeI insertion sites yields XbaI/SpeI scars (*) encoding two serines at module boundaries. DD, docking domain; DH, dehydratase; ER, enoylreductase; KS^Q^, priming KS; KR, ketoreductase; KR^0^, epimerase.

Here we describe a two-plasmid system that enables the expression of a refactored pikromycin PKS in *Escherichia* *coli* as well as the combinatorial biosynthesis of new macrolactones and macrolide antibiotics^17^ (Fig. 1). One plasmid encodes the first pikromycin module (**P1**) and the upstream portion of the fourth module (**P4**^**N**^), while the other plasmid encodes the downstream portion of the fourth module (^**C**^**P4**) and the seventh module (**P7**). Module-encoding inserts can be sequentially ligated into both plasmids, making it easy to construct and modify large synthases. The incorporation of **P2**, **P3**, **P5** and **P6** into this system yielded a functional, refactored pikromycin PKS in *E. coli* K207-3, composed of six polypeptides rather than the four natural ones (PikAI–PikAIV)^18^. Docking domain optimization and expression of the editing TE PikAV improved narbonolide (**1**) production 2.1-fold to 49 ± 4 mg l^−1^ in shake flasks. Replacing modules with others from the pikromycin and rapamycin PKSs yielded new narbonolide derivatives. Deleting, inserting and mutating modules also resulted in new derivatives, including those of the methymycin precursor 10-deoxymethynolide (10-dml, **4**). The expression of enzymes that biosynthesize desosamine, modify macrolactones with it, hydroxylate the resulting macrolides and confer macrolide antibiotic resistance to *E. coli* enabled the generation of narbomycin (**2**), pikromycin (**3**), YC-17 (**5**) and methymycin (**6**), as well as five derivatives thereof. Knocking out pathways that compete with desosamine biosynthesis with CRISPR–Cas9 increased narbomycin production to 37 ± 3 mg l^−1^. New narbomycin derivatives (**21**, **22** and **23**), resulting from the replacement of **P3** by **P5**, **P2** by **R6** and **P3** by **R5** (**R5** and **R6** are the fifth and sixth modules of the rapamycin PKS), were purified, characterized and observed to possess antibacterial activity.

## Results

### Construction and evaluation of the two-plasmid system

Plasmids p**P1**-**P4**^**N**^ and p^**C**^**P4**-**P7** were generated through Gibson assembly using pCDF-1b and pET-28b, respectively (Fig. 1 and Supplementary Figs. 1 and 2). Synthetic DNA encoding docking domain motifs (^C^DD and ^N^DD) from the third module of the erythromycin PKS (also known as the 6-deoxyerythronolide B synthase or DEBS), **E3**, was inserted to help non-covalently connect **P4**^**N**^ and ^**C**^**P4**^19^. A HindIII-N_12_-SpeI insertion site was placed between the regions encoding **P1** and **P4**^**N**^ as well as ^**C**^**P4** and **P7** to enable the sequential insertion of fragments encoding modules **A**–**D** and generate synthases such as **P1**-**A**-**B**-**P4**-**C**-**D**-**P7**. The two-plasmid system, while necessary due to the size limit of plasmids in *E. coli* (~30 kb), has the benefit that synthases can be combinatorially constructed more efficiently. The ^C^DD–^N^DD pairs used for **A**, **B**, **C** and **D** are from the **S8**, **S3**, **E5** and **S5** modules of the spinosyn and erythromycin PKSs, respectively. The insertion of the DNA encoding a module preserves the HindIII-N_12_-SpeI insertion site and creates a downstream XbaI/SpeI scar that appears on the polypeptide level as two serines in a permissive loop at the updated module boundary^20,21^. The alanines, prolines and serines encoded by the insertion site and scar are common in KS–AT linkers and unlikely to affect protein folding^21^. The cloning plasmids that maintain the updated modules have been previously described^16^. Briefly, they were constructed by inserting synthetic DNA between the HindIII and XbaI sites of pUC19 to introduce a T7 promoter, a *lac* operator, a ribosome binding site, a T7 terminator, restriction sites for importing DNA fragments encoding the upstream and downstream portions of a module (SpeI/BmtI and MfeI/XbaI), and DNA encoding docking domain motifs to aid the noncovalent assembly of the module (Supplementary Fig. 3).

**Fig. 2:**
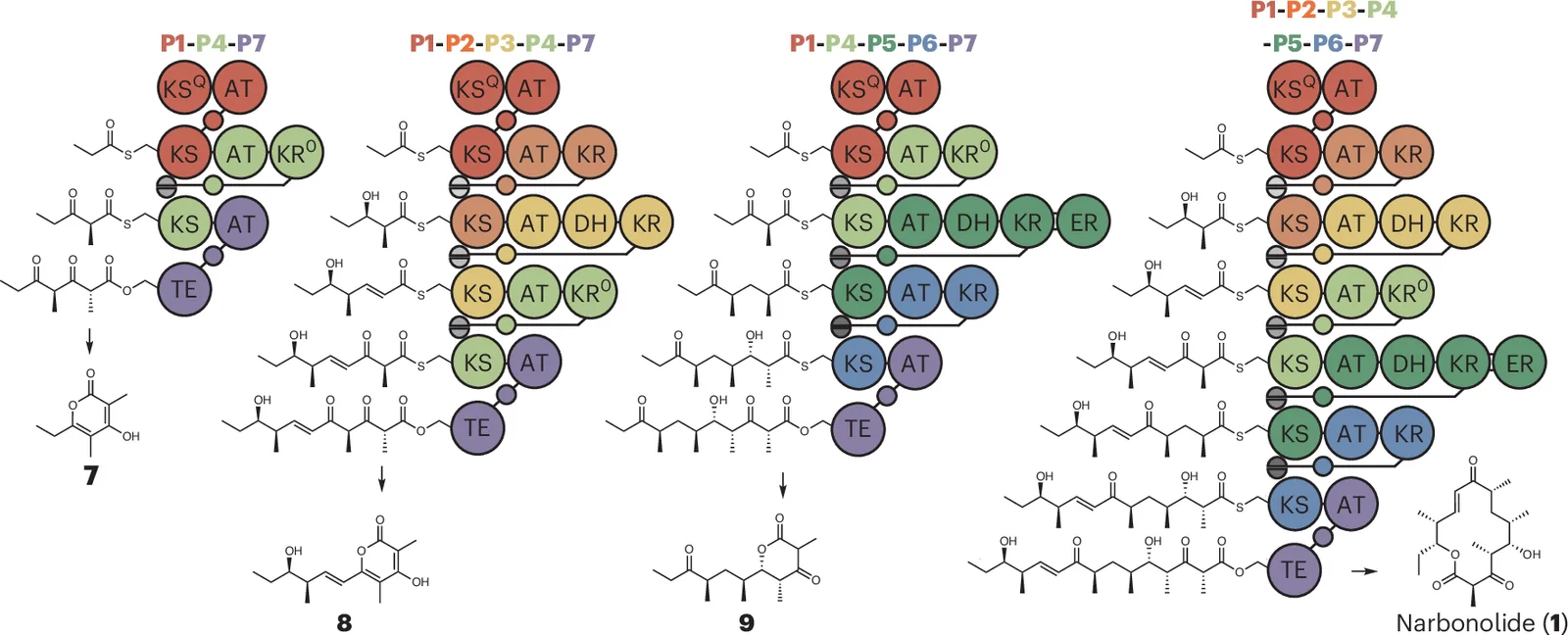
Evaluation of the two-plasmid system and narbonolide biosynthesis by the refactored pikromycin PKS. Expected products are observed from **P1**-**P4**-**P7**, **P1**-**P2**-**P3**-**P4**-**P7**, **P1**-**P4**-**P5**-**P6**-**P7** and **P1**-**P2**-**P3**-**P4**-**P5**-**P6**-**P7**.

To test the 2-plasmid system, p**P1**-**P4**^**N**^ and p^**C**^**P4**-**P7** were transformed into *E. coli* K207-3^18^. This engineered strain harbours the *Bacillus subtilis* phosphopantetheinyl transferase Sfp and *Streptomyces coelicolor* propionyl-coenzyme A (CoA) carboxylase to respectively activate ACP domains and help convert propionate supplied to the media into (2*S*)-methylmalonyl-CoA. Transformed cells were cultured in polyketide production media, and the anticipated product of **P1**-**P4**-**P7**, triketide pyrone **7**, was identified by liquid chromatography–mass spectrometry (LC–MS) (high resolution used throughout) of the media extract (Fig. 2 and Supplementary Fig. 4). Plasmids p**P1**-**P2**-**P3**-**P4**^**N**^ and p^**C**^**P4**-**P5**-**P6**-**P7** were then constructed and cotransformed with p^**C**^**P4**-**P7** and p**P1-P4**^**N**^, respectively. Pentaketides **8** and **9**, the anticipated products of **P1**-**P2**-**P3**-**P4**-**P7** and **P1**-**P4**-**P5**-**P6**-**P7**, were identified by LC–MS of the media extracts (Fig. 2 and Supplementary Figs. 5 and 6). Plasmids p**P1**-**P2**-**P3**-**P4**^**N**^ and p^**C**^**P4**-**P5**-**P6**-**P7** were then cotransformed. The media extract contained a compound with the mass expected for narbonolide (**1**) (Fig. 2 and Supplementary Fig. 7). It was purified by silica gel chromatography and semipreparative high-performance liquid chromatography, and its identity was confirmed by NMR spectroscopy^22^ (Supplementary Figs. 8–10).

### Optimizing narbonolide production from the refactored synthase

Fermentation conditions were identified (10 days at 16 °C with 60 mM sodium propionate) that yield a narbonolide titre of 23 ± 2 mg l^−1^ (Supplementary Fig. 11). LC–MS analysis of the media extract revealed that shunt products (the products expected of **P1**-**P2**-**P7**, **P1**-**P4**-**P7**, **P1**-**P6**-**P7**, **P1**-**P5**-**P6**-**P7**, **P1**-**P2**-**P3**-**P4**-**P7**, **P1**-**P2**-**P5**-**P6**-**P7** and **P1**-**P4**-**P5**-**P6**-**P7**) were also generated at lower levels^16^ (Supplementary Fig. 12a). We previously proposed such shunt products can be produced due to ACPs not being sufficiently restrained by their downstream docking domain motifs^16^. In the refactored synthase, the ^C^DD–^N^DD pairs of **P3**, **P5** and **P6** come from **S3**, **E5** and **S5**, respectively. Thus, replacing these with their natural docking domain motifs could result in more restrained ACPs, fewer shunt products and an increased narbonolide titre. The DNA fragments encoding the native ^C^DD–^N^DD pairs for **P3**, **P5** and **P6** were inserted into the **P3**, **P5** and **P6** cloning plasmids and the natural versions of **P3**, **P5** and **P6** were independently inserted into the refactored pikromycin PKS. This increased narbonolide production 1.1-fold, 1.2-fold and 1.1-fold, respectively, and the combined replacement of natural **P3**, **P5** and **P6** increased narbonolide production 1.3-fold to 30 ± 2 mg l^−1^ (Supplementary Fig. 12b). Shunt product titres from synthases harbouring the natural docking domains were slightly lower (Supplementary Fig. 12a).

Next, the effect of the editing TE PikAV on the refactored system was investigated. This TEII is thought to hydrolyse acetyl or propionyl groups inappropriately bound to ACPs formed by untimely, KS-mediated decarboxylations of malonyl and methylmalonyl extender units^23^. Within *S. venezuelae*, the overexpression, but not the inactivation, of PikAV has been observed to decrease pikromycin production^23,24^. However, for other PKS-containing gene clusters the inactivation of TEII has resulted in decreased polyketide production^25–28^. Proposing that some level of PikAV would improve the activity of the refactored pikromycin PKS in *E. coli*^29–31^, PikAV expression plasmids were constructed^32^. A single copy of *pikAV* on a pACYC vector was observed to maximally increase narbonolide production (1.6-fold to 49 ± 4 mg l^−1^ for the docking-domain-optimized PKS) (Supplementary Fig. 13).

### Module swapping to yield narbonolide derivatives

**Fig. 3:**
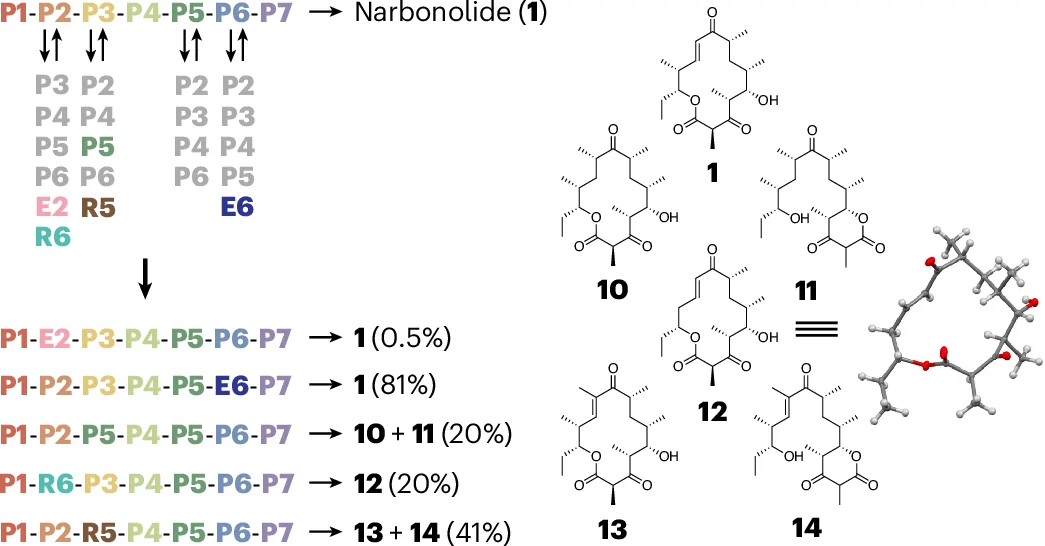
Module swapping of the refactored pikromycin PKS. While 16 swaps with pikromycin modules were attempted, only the replacement of **P3** with **P5** yielded an active PKS. This synthase generates anticipated macrolactone **10** as well as δ-lactone **11**. Swapping **P2** and **P6** for **E2** and **E6** (from the erythromycin PKS), respectively, yielded hybrid synthases that generate narbonolide (**1**). Swapping **P2** for **R6** and **P3** for **R5** (from the rapamycin PKS) yielded hybrid synthases that generate macrolactones **12** and **13**, as well as δ-lactone **14**. A crystal structure shows the absolute stereochemistry of **12**. Titres are reported in comparison to that of narbonolide by the refactored pikromycin PKS. Source data

With the refactored pikromycin PKS in hand, module-swapping experiments were performed (Fig. 3). Because a recent study, in which we constructed 155 triketide–pentaketide synthases from the updated modules of the pikromycin PKS, suggested that synthases containing fewer module swaps are more likely to function, we decided to perform single module swaps in the refactored pikromycin PKS^16^. Thus, **P2**, **P3**, **P5** and **P6** were replaced with each of the other extension modules of the pikromycin PKS. Of the 16 module-swapped PKSs, only **P1**-**P2**-**P5**-**P4**-**P5**-**P6**-**P7** produced its expected macrolactone (**10**), as confirmed by LC–MS and NMR spectroscopy (Fig. 3 and Supplementary Figs. 14 and 15). This synthase produced an equivalent quantity of δ-lactone **11**, as determined by LC–MS and its absorbance spectrum (Supplementary Fig. 14). The overall heptaketide titre from **P1**-**P2**-**P5**-**P4**-**P5**-**P6**-**P7** (**10** + **11**) is 20% that of the narbonolide titre from **P1**-**P2**-**P3**-**P4**-**P5**-**P6**-**P7**.

To evaluate the compatibility of the refactored pikromycin PKS with modules from other PKSs, we first replaced **P2** and **P6** with **E2** and **E6**, erythromycin modules that conduct the same chemistry (Fig. 3). The hybrid synthases **P1**-**E2**-**P3**-**P4**-**P5**-**P6**-**P7** and **P1**-**P2**-**P3**-**P4**-**P5**-**E6**-**P7** produce 0.5% and 81%, respectively, the narbonolide titre of **P1**-**P2**-**P3**-**P4**-**P5**-**P6**-**P7** (Supplementary Figs. 16 and 17). Next, the hybrid synthases **P1**-**R6**-**P3**-**P4**-**P5**-**P6**-**P7** and **P1-P2-R5-P4-P5-P6-P7** were constructed using modules from the rapamycin PKS. **P1**-**R6**-**P3**-**P4**-**P5**-**P6**-**P7** produced its expected macrolactone **12**, while **P1**-**P2**-**R5**-**P4**-**P5**-**P6**-**P7** produced its expected macrolactone **13** as well as twice as much δ-lactone **14**. Compounds **12**, **13** and **14** were characterized by LC–MS, ultraviolet absorbance and NMR spectroscopy, and the absolute configuration of **12** was determined by X-ray crystallography (Supplementary Figs. 18–28 and Methods). The overall heptaketide titres from **P1**-**R6**-**P3**-**P4**-**P5**-**P6**-**P7** (**12**) and **P1**-**P2**-**R5**-**P4**-**P5**-**P6**-**P7** (**13** + **14**), relative to the narbonolide titre from **P1**-**P2**-**P3**-**P4**-**P5**-**P6**-**P7**, were 20% and 41%, respectively.

### Accessing hexaketide and octaketide derivatives

**Fig. 4:**
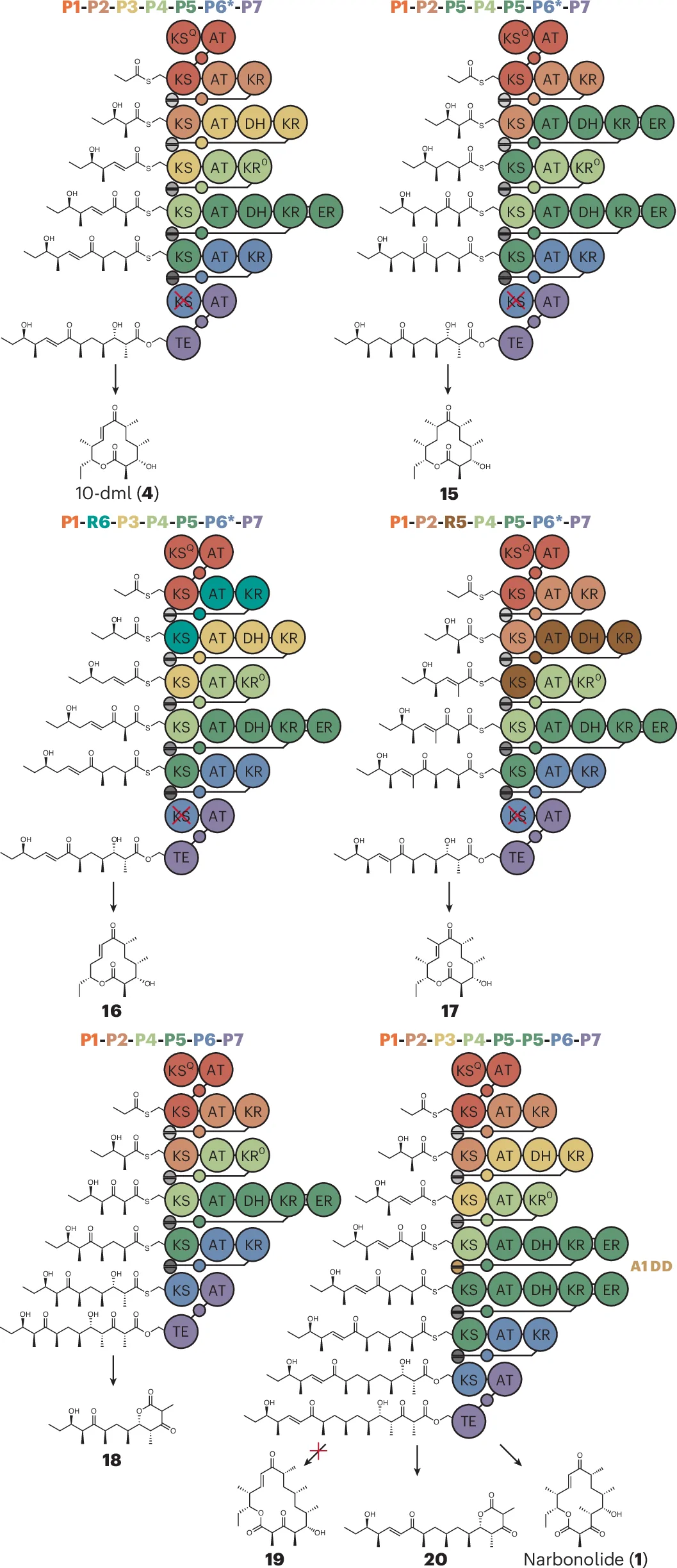
Construction of hexaketide and octaketide synthases. Hexaketide synthases **P1**-**P2**-**P3**-**P4**-**P5**-P6*-**P7**, **P1**-**P2**-**P5**-**P4**-**P5**-P6*-**P7**, **P1**-**R6**-**P3**-**P4**-**P5**-P6*-**P7**, **P1**-**P2**-**R5**-**P4**-**P5**-P6*-**P7** and **P1**-**P2**-**P4**-**P5**-**P6**-**P7** produce **4** (10-dml), **15**, **16**, **17** and **18**, respectively. The asterisk (*) next to **P6** indicates the C-to-A mutation of its KS. The octaketide synthase **P1**-**P2**-**P3**-**P4**-**P5**-**P5**-**P6**-**P7** does not produce the anticipated octaketide macrolactone **19** but does produce octaketide δ-lactone **20** and 3.7 times as much heptaketide macrolactone **1** (narbonolide). Docking domain motifs from **A1** of the amphotericin PKS were used for the upstream **P5**.

Within *S. venezuelae*, the heptaketide narbonolide and the hexaketide 10-dml are produced as intermediates in the biosynthesis of pikromycin and methymycin, respectively^17^. Hexaketide production results from an alternate translation start site for PikAIV that inactivates the **P6** KS^33^. To produce 10-dml (**4**) in *E. coli*, a mutagenic approach was used whereby the **P6** KS reactive cysteine is replaced with an alanine^34–36^ (Figs. 1a and 4). As confirmed by LC–MS and NMR spectroscopy, the mutated synthase **P1**-**P2**-**P3**-**P4**-**P5**-P6*-**P7** selectively produces 10-dml with a titre 1.2-fold greater than the narbonolide titre from **P1**-**P2**-**P3**-**P4**-**P5**-**P6**-**P7** (Fig. 4 and Supplementary Figs. 29–32). **P1**-**P2**-**P5**-**P4**-**P5**-P6*-**P7,** **P1**-**R6**-**P3**-**P4**-**P5**-P6*-**P7** and **P1**-**P2**-**R5**-**P4**-**P5**-P6*-**P7** were constructed, and their anticipated 10-dml derivatives, **15**, **16** and **17**, were detected (Fig. 4 and Supplementary Figs. 33–35). Hexaketide synthase **P1**-**P2**-**P4**-**P5**-**P6**-**P7** was also constructed and observed to produce δ-lactone **18** at a titre 1.4-fold greater than the narbonolide titre from **P1**-**P2**-**P3**-**P4**-**P5**-**P6**-**P7** (Fig. 4 and Supplementary Fig. 36). δ-Lactonization of **18** was confirmed by NMR spectroscopy (Supplementary Figs. 37–39). Because octaketide macrolide antibiotics like tylosin and mycinamycin are naturally occurring, the octaketide synthase **P1**-**P2**-**P3**-**P4**-**P5**-**P5**-**P6**-**P7** was also constructed (Fig. 4). A product of the expected mass was observed; however, its absorbance spectrum revealed it to be δ-lactone **20** rather than the anticipated octaketide macrolactone **19** (Supplementary Fig. 40). Narbonolide (**1**) is the main product of this synthase, at a titre 3.7-fold that of **20**.

### Accessing macrolide antibiotics

**Fig. 5:**
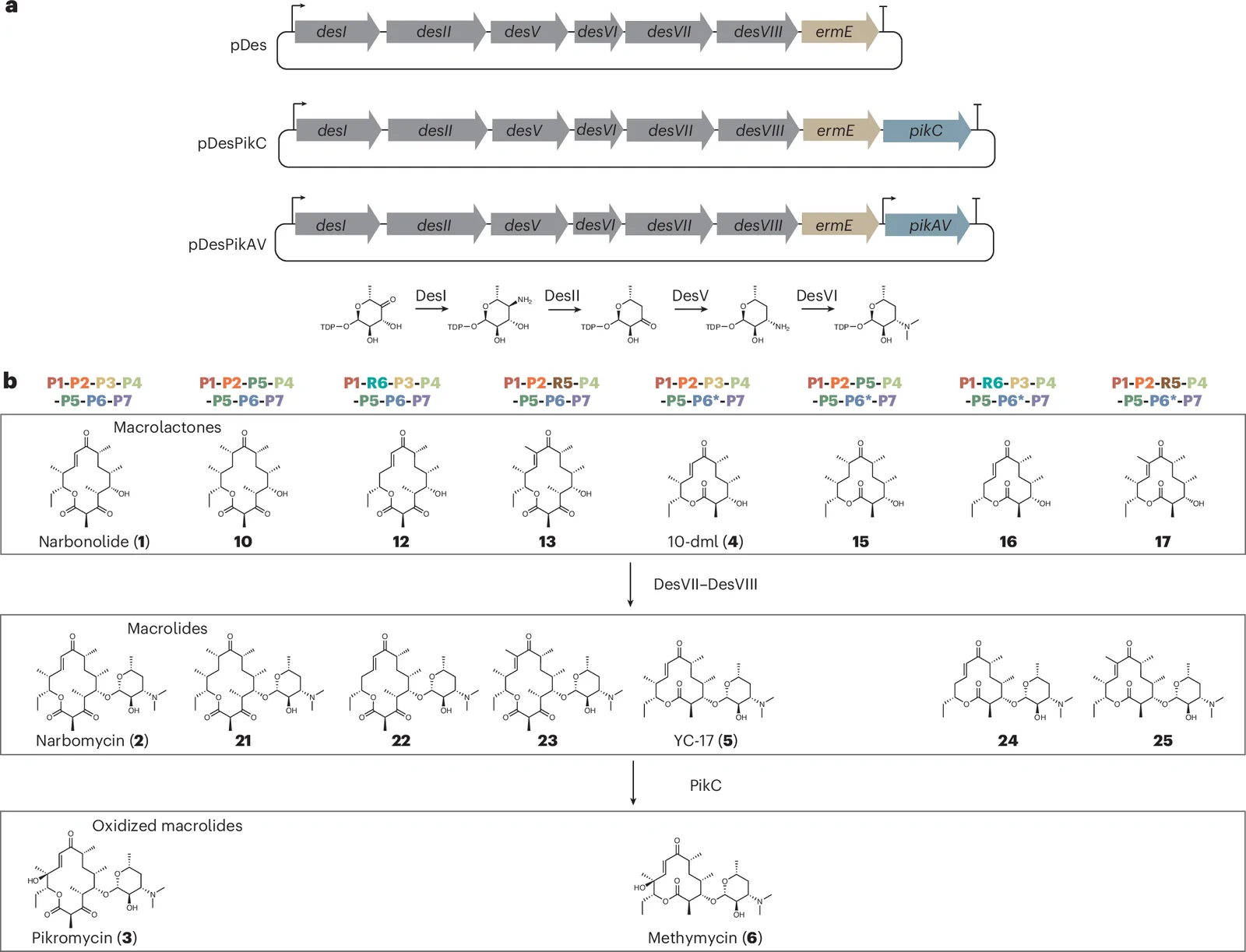
Production of macrolide antibiotics in *E. coli*. **a**, Plasmids pDes, pDesPikC and pDesPikAV encode desosamine biosynthesis/transfer and macrolide resistance enzymes. Plasmids pDesPikC and pDesPikAV also encode the P450 monooxygenase PikC and the editing TE PikAV, respectively. PikAV is controlled by a second T7 promoter in pDesPikAV. TDP, thymidine diphosphate. **b**, With the exception of **15**, each of the macrolactones in this study is glycosylated by DesVII/DesVIII. Only narbomycin (**2**) and YC-17 (**5**) were hydroxylated by PikC. The stereochemistries shown are based on those naturally generated by the pikromycin pathway.

The desosamine moiety is critical to the biological activities of macrolide antibiotics^37^. Plasmid pDes was constructed to express the desosamine biosynthetic pathway (DesI, DesII, DesV and DesVI), the desosamine transferase (DesVII/DesVIII)^38,39^ and the macrolide antibiotic resistance enzyme ErmE^40^ (Fig. 5a and Supplementary Fig. 41). The gene encoding the P450 monooxygenase PikC that converts narbomycin into pikromycin and YC-17 into methymycin, was added to create plasmid pDesPikC^41^. pDes and pDesPikC were transformed into *E. coli* K207-3 harbouring PKS expression plasmids. The transformants were cultured in polyketide production media at 16 °C for 10 days, and the media extracts were analysed by LC–MS. *E. coli* K207-3 with pDes completely converted narbonolide to narbomycin (1.1 ± 0.2 mg l^−1^), as confirmed by LC–MS and NMR spectroscopy^22^ (Fig. 5b and Supplementary Figs. 42–45). LC–MS indicates that cells with pDesPikC convert 24% of narbomycin to pikromycin, to provide a pikromycin titre of 0.45 ± 0.02 mg l^−1^ (Fig. 5b and Supplementary Figs. 46–47). Proposing that the low PikC activity is from low protein expression and/or inefficient reduction by *E. coli* proteins, PikC expression was supplemented with plasmid pPikC, which contains another copy of *pikC*^42^. This improved the conversion from narbomycin to 38%, and the pikromycin titre reached 0.58 ± 0.02 mg l^−1^ (Supplementary Fig. 47). **P1**-**P2**-**P3**-**P4**-**P5**-P6*-**P7** was also expressed with pDes and pDesPikC to yield compounds with mass spectra consistent with **5** (YC-17) and **6** (methymycin), respectively, and no detectable 10-dml (**4**) (Fig. 5b and Supplementary Figs. 48 and 49). NMR spectroscopy confirmed the identity of YC-17 (Supplementary Figs. 50–52).

Next, cells expressing **P1**-**P2**-**P5**-**P4**-**P5**-**P6**-**P7**, **P1**-**R6**-**P3**-**P4**-**P5**-**P6**-**P7**, **P1**-**P2**-**R5**-**P4**-**P5**-**P6**-**P7**, **P1**-**P2**-**P5**-**P4**-**P5**-P6*-**P7**, **P1**-**R6**-**P3**-**P4**-**P5**-P6*-**P7** and **P1**-**P2**-**R5**-**P4**-**P5**-P6*-**P7** were transformed with pDes. While the desosaminylated macrolide anticipated from **P1**-**P2**-**P5**-**P4**-**P5**-P6*-**P7** was not observed by LC–MS, the desosaminylated macrolides **21**, **22**, **23**, **24** and **25** were observed from the other synthases without traces of their respective aglycone precursors, **10**, **12**, **13**, **16** and **17** (Fig. 5b and Supplementary Figs. 53–57). Narbomycin derivatives **21**, **22** and **23** were isolated and characterized by NMR spectroscopy (Supplementary Figs. 58–66). The addition of PikC through pDesPikC + pPikC did not convert **21**–**25** into their anticipated oxidized macrolide products. This is in agreement with recent studies by the Sherman laboratory in which PikC showed a large preference for narbomycin over similar, chemoenzymatically generated derivatives^43^ (Fig. 5b).

### Metabolic engineering boosts titres of macrolide antibiotics

**Fig. 6:**
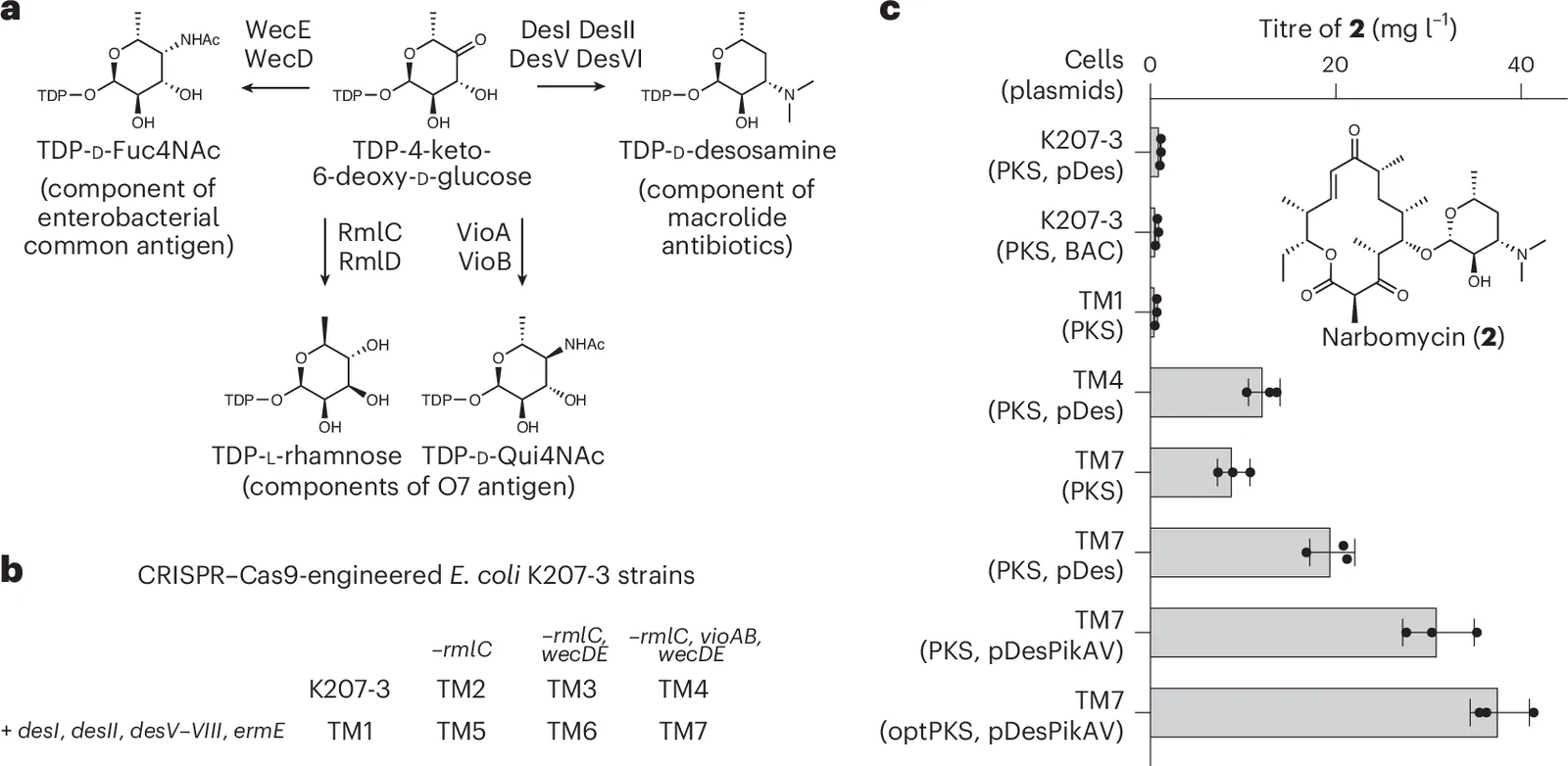
CRISPR–Cas9 engineering helps boost titres of macrolide antibiotics. **a**, Biosynthetic pathways in *E. coli* compete with desosamine biosynthesis for TDP-4-keto-6-deoxy-d-glucose. **b**, The TM1 strain was engineered by inserting the desosamine biosynthesis/transfer genes into the *E. coli* K207-3 genome. The TM2–TM4 and TM5–TM7 strains were engineered through sequentially inactivating *rmlC*, *wecD*/*E*, *vioA*/*B* in *E. coli* K207-3 and TM1, respectively (Supplementary Table 1). **c**, Narbomycin production is reported for engineered *E. coli* strains transformed with p**P1**-**P2**-**P3**-**P4**^**N**^ and p^**C**^**P4**-**P5**-**P6**-**P7** (PKS), encoding the refactored pikromycin PKS, as well as an additional plasmid or bacterial artificial chromosome (BAC) that encodes enzymes from the pikromycin biosynthetic pathway (Fig. 5a). TM7 cells with plasmids encoding the docking-domain-optimized PKS (optPKS) and pDesPikAV yield a narbomycin (**2**) titre of 37 ± 3 mg l^−1^. Data are from biological triplicates (mean ± standard deviation). Source data

The low titres of narbomycin, pikromycin, YC-17, methymycin and their derivatives hampers investigations of their structures and antibacterial activities. Because no aglycones were observed from *E. coli* K207-3 generating these macrolides, we proposed that the overexpression of the relatively small proteins from pDes, pDesPikC and pDesPikAV outcompeted PKS expression. Thus, the operon from pDes was transferred to the low copy number (1–2) bacterial artificial chromosome pMKBAC02^44^. While narbonolide was completely converted to narbomycin, the narbomycin titre was 65% that of cells transformed with pDes (Fig. 6). The operon was also integrated into the *E. coli* K207-3 genome with CRISPR–Cas9 to yield TM1 cells (Supplementary Fig. 67). Although TM1 cells expressing the refactored pikromycin synthase completely convert narbonolide to narbomycin, the productivity is still only 56% that of *E. coli* K207-3 transformed with pDes.

In a previous study, pathways that use TDP-4-keto-6-deoxy-d-glucose were knocked out of *E. coli* K207-3, and the resulting strains, transformed with plasmids encoding desosamine biosynthesis/transfer genes, were observed to more efficiently add desosamine to supplied macrolactones^45^. TDP-4-keto-6-deoxy-d-glucose is not only a precursor in the biosynthesis of the pikromycin monosaccharide, TDP-d-desosamine, but also of the *O*-specific polysaccharide chain of the lipopolysaccharide and the glycolipid on the cell surface of *E. coli*^45–47^ (Fig. 6a). Thus, *rmlC*, *wecD*, *wecE*, *vioA* and *vioB* were inactivated by CRISPR–Cas9 in both *E. coli* K207-3 and TM1 cells to yield TM4 and TM7 cells, respectively (as well as intermediate strains TM2, TM3, TM5 and TM6) (Fig. 6b and Supplementary Fig. 68). TM4 and TM7 cells, transformed with the plasmids encoding the refactored pikromycin PKS (and pDes for TM4 cells), produce 19-fold and 14-fold higher narbomycin titres than TM1 cells, respectively (Fig. 6c). TM7 cells transformed with the plasmids encoding the docking-domain-optimized pikromycin PKS and pDesPikAV produce a 58-fold higher narbomycin titre than that of TM1 cells transformed with the plasmids encoding the docking-domain-unoptimized pikromycin PKS (37 ± 3 mg l^−1^). Cotransforming TM7 cells with PKS expression plasmids and pDesPikAV or pDesPikC was also observed to boost the titres of other desosaminylated macrolides (Supplementary Table 4).

### Antibacterial activity

Following purification and NMR spectroscopy characterization, narbomycin (**2**), YC-17 (**5**), **21**, **22** and **23** were evaluated for antibacterial activity against both gram-positive (*B. subtilis*, *Staphylococcus epidermidis*, *Micrococcus luteus*) and gram-negative (*E. coli* and *Pseudomonas putida*) bacteria (Table 1 and Supplementary Fig. 69). Commercial pikromycin (**3**) was used as a positive control. Pikromycin (**3**), narbomycin (**2**) and **22** are active against gram-positive bacteria, with minimum inhibitory concentrations (MICs) of 3.12–6.25 μM, and YC-17 is active against gram-positive bacteria, with an MIC of 100–200 μM^48,49^. Compound **21** only showed weak antibacterial activity against *B. subtilis*, with an MIC of 200 μM. Compound **23** showed weak antibacterial activity against all three gram-positive bacteria, with an MIC of 200 μM. Only pikromycin (**3**) showed activity against gram-negative bacteria, with MICs of 200 μM.

**Table 1:** Antibacterial activities of purified macrolides

	MIC values (µM)					
Bacteria	Pikromycin (3)	Narbomycin (2)	YC-17 (5)	21	22	23
*B. subtilis*	6.25	6.25	100	200	6.25	200
*S. epidermidis*	6.25	6.25	200	>200	6.25	200
*M. luteus*	3.12	3.12	100	>200	3.12	200
*E. coli*	200	>200	>200	>200	>200	>200
*P. putida*	200	>200	>200	>200	>200	>200

*B. subtilis*, *S. epidermidis* and *M. luteus* are gram-positive bacteria; *E. coli* and *P. putida* are gram-negative bacteria.

## Discussion

Since the erythromycin PKS was sequenced in 1990, scientists have been trying to harness the synthetic power of this and other modular PKSs for the production of designer polyketides and new medicines^11,12^. As *E. coli* is more genetically tractable than the natural erythromycin producer *Saccharopolyspora erythraea*, a main goal was realized in 2001 when the erythromycin skeleton, 6-deoxyerythronolide B (6-dEB), was produced by *E. coli*^50^. In 2010, production of the macrolide antibiotic erythromycin A by *E. coli* was reported^51^.

Next to the erythromycin PKS, the pikromycin PKS is the most-studied modular PKS^17,52,53^. This study reports the reconstitution of the pikromycin PKS in *E. coli* to yield narbonolide and the macrolide antibiotics narbomycin and pikromycin. The pikromycin pathway has several advantages over the erythromycin pathway: (1) fewer tailoring enzymes are needed. The erythromycin pathway requires six plasmids, which are not stably maintained by cells containing them. (2) Macrolide antibiotics such as narbomycin and YC-17 can be accessed without P450 enzymes, which are not very active in *E. coli*. The erythromycin pathway contains two P450 enzymes, with EryF performing a requisite, first tailoring step. (3) Higher yields of narbomycin (37 mg l^−1^) can be generated compared with erythromycin A (~1 mg l^−1^). (4) The methymycin–pikromycin pathway is more versatile, with the PKS having evolved to generate both a hexaketide and a heptaketide and DesVII–DesVIII having evolved to transfer d-desosamine to these 12- and 14-membered macrolactones. (5) The BioBricks-like platform developed here for the refactored pikromycin PKS facilitates the modular synthesis of new macrolide antibacterials through the combinatorial construction of PKSs.

Both the recently reported one-plasmid system and the two-plasmid system described here for PKS expression in *E. coli* rely on heterologous docking domain pairs to non-covalently connect the N-terminal and C-terminal portions of modules, which are expressed on separate polypeptides^16,54,55^. As the current study uses as many as six heterologous docking domain pairs per synthase, we compared the performances of heterologous pairs with their natural counterparts. The combined replacement of the **S3**, **E5** and **S5** docking domain pairs with the docking domains pairs native to **P3**, **P5** and **P6** improved narbonolide production by 30% (Supplementary Fig. 12). This difference may reflect some degree of co-evolution between docking domain pairs and the modules with which they are associated^9,10^. While maximal titres may not be important when first accessing synthases that generate new polyketides, native docking domain pairs may be subsequently used to optimize synthase productivity. We have observed that differences in polypeptide expression can result in module-skipping^56^. Attenuated T7 promoters could be used for more highly expressed polypeptides to balance stoichiometries and increase the production of desired polyketides.

The largest impediment to combinatorially engineering the refactored pikromycin PKS is KS gatekeeping. While the use of the updated modules ensures compatibility of KSs with the α-substituents and β-substituents they are presented, it does not ensure compatibility with substituents beyond the β-carbon. Comprehensive module-swapping experiments with the pikromycin modules showed that only 1 out of the 16 singly swapped synthases in which **P2**, **P3**, **P4**, **P5** or **P6** replaces **P2**, **P3**, **P5** or **P6** in the refactored pikromycin PKS yields its expected product. That **P1**-**P2**-**P5**-**P4**-**P5**-**P6**-**P7** is functional is consistent with our studies with the one-plasmid system in which **P5** was observed to function downstream of **P1**-**P2** in **P1**-**P2**-**P5**-**P7**, **P1**-**P2**-**P5**-**P5**-**P7** and **P1**-**P2**-**P5**-**P6**-**P7**^16^. In these synthases, the **P5** KS immediately downstream of **P1**-**P2** accepts a triketide with the same substituents and stereochemistries as in its native substrate at the α–γcarbons. However, differences beyond the γ-carbon are not always tolerated by this KS. In the octaketide synthase **P1**-**P2**-**P3**-**P4**-**P5**-**P5**-**P6**-**P7**, the downstream **P5** KS is presented the same substituents and stereochemistries as in its native substrate at the α–γcarbons, yet four-fifths of pentaketide intermediates skip the downstream **P5** module, probably due to low flux through its KS. Thus, even **P5** KS, the most promiscuous of the pikromycin KSs, shows a substantial preference for its natural substrate. A similar observation was made when a heterologous module was inserted into the erythromycin PKS^57^. Even though the module was inserted into the first polypeptide, it was skipped and the heptaketide 6-deoxyerythronolide B, rather than the anticipated octaketide, was produced. To obtain a greater octaketide titre from **P1**-**P2**-**P3**-**P4**-**P5**-**P5**-**P6**-**P7**, the KS of the downstream **P5** could be replaced with one that naturally accepts a polyketide with l-oriented methyl groups at the α-position and γ-position and methylenes at the β-position and δ-position. Alternatively, KS gatekeeping can be circumvented by using naturally promiscuous KSs, such as those from the rapamycin PKS, as exemplified by **P1-R6-P3-P4-P5-P6-P7** and **P1-P2-R5-P4-P5-P6-P7**^16,58,59^. We are now systematically investigating the use of promiscuous KSs within engineered synthases.

Our results indicate some level of co-evolution between the upstream modules of synthases. As all of the interactions between acyl-ACPs and downstream KSs are native in **P1**-**E2**-**P3**-**P4**-**P5**-**P6**-**P7**, KS gatekeeping does not account for its poor activity. This hybrid synthase may be suboptimal due to poor intermodular interactions (acyl-ACPs and upstream KSs); however, the robust activity of **P1**-**P2**-**P3**-**P4**-**P5**-**E6**-**P7** suggests this is not a general phenomenon. The specialization of at least the upstream KS domains of PKSs for receiving short substrates indicates that upstream modules may evolutionarily comigrate^16^.

Several of the engineered PKSs in this study produce a δ-lactone instead of, or in addition to, a macrolactone (**P1**-**P2**-**P4**-**P5**-**P6**-**P7** produces **18**, **P1**-**P2**-**P5**-**P4**-**P5**-**P6**-**P7** produces **11** in addition to **10**, **P1**-**P2**-**R5**-**P4**-**P5**-**P6**-**P7** produces **14** in addition to **13**, **P1**-**P2**-**P3**-**P4**-**P5**-**P5**-**P6**-**P7** produces **20** in addition to **1**) (Figs. 3 and 4 and Supplementary Figs. 14, 22, 26–28 and 36–40). Biochemical and structural studies of the pikromycin TE have revealed that functional groups installed by the first four pikromycin modules (**P1**–**P4**) are complementary to features in the TE active site and aid in the macrocyclization of the natural hexaketide and heptaketide intermediates^60–62^. A crystal structure of a version of the pikromycin TE in which 2,3-diaminopropionate replaces the catalytic serine and is connected through an amide linkage to the heptaketide intermediate (Protein Data Bank (PDB) 9CGN) shows the C8 methyl group fitting into an active site pocket as well as a hydrogen bond between the enone oxygen and the side chain of Thr1125^62^. These interactions help position the C13 hydroxyl group, normally generated by **P2**, for nucleophilic attack on the C1 carbonyl. The production of a similar quantity of δ-lactone **14** to macrolactone **13** by **P1**-**P2**-**R5**-**P4**-**P5**-**P6**-**P7**, suggests that the introduced C10 methyl group clashes with Tyr1073 and hinders the macrocyclization reaction. The production of macrolactones **10** and **15** by **P1**-**P2**-**P5**-**P4**-**P5**-**P6**-**P7** and **P1**-**P2**-**P5**-**P4**-**P5**-P6*-**P7**, respectively, demonstrates that the double bond normally generated by **P3** is not required for macrocyclization. However, because **P1**-**P2**-**P5**-**P4**-**P5**-**P6**-**P7** produces a similar quantity of δ-lactone **11** to macrolactone **10** indicates that the introduced C10 methyl group sterically impedes closure of the macrolactone ring. Generating 16-membered octaketide macrolactones such as those of tylosin and mycinamycin may not be trivial with PikTE. As **P1**-**P2**-**P3**-**P4**-**P5**-**P5**-**P6**-**P7** yields an octaketide δ-lactone but no macrolactone, this indicates that even when a substrate contains the natural moiety generated by **P1**-**P2**-**P3**-**P4**, δ-lactone formation can outcompete macrocyclization. Another strategy to access 16-membered octaketide macrolactones is to use the tylosin–mycinamycin TEs in synthases in which three modules are inserted between **P1** and **P4**.

Why narbomycin titres decrease in cells genomically expressing desosamine biosynthetic enzymes (TM7 cells) relative to narbonolide titres in cells not expressing these enzymes (K207-3 cells) yet increase when a plasmid supplements genomic expression of desosamine biosynthesis enzymes (TM7 cells with pDes) is unclear (Fig. 6). Multiple factors seem to be at play, such as expression of the desosamine biosynthetic enzymes competing with the PKS polypeptide expression and one or more of the desosamine biosynthesis enzymes being weakly active. Individually tuning the expression levels of the desosamine biosynthetic enzymes could lead to higher narbomycin titres.

The described platform provides a means to study mysterious assembly line phenomena. For example, it has been debated whether the full pikromycin PKS produces the hexaketide 10-dml or not. In this study, no 10-dml was detected from **P1**-**P2**-**P3**-**P4**-**P5**-**P6**-**P7**. However, the single cysteine-to-alanine point mutation to the **P6** KS renders **P1**-**P2**-**P3**-**P4**-**P5**-P6*-**P7** an efficient producer of 10-dml. Why **P6** ACP does not transfer the hexaketide intermediate to TE in **P1**-**P2**-**P3**-**P4**-**P5**-**P6**-**P7** is not clear. One hypothesis is that in the absence of heptaketide-bound **P7** ACP, hexaketide-bound **P6** ACP and TE approach one another. **P1**-**P2**-**P3**-**P4**-**P5**-**P5**-**P6**-**P7** may predominantly produce narbonolide due to a similar module-skipping phenomenon that can be investigated through the platform.

Many opportunities exist to boost the titres of polyketides and macrolide antibiotics from *E. coli*. Genomic deletion of a putative propionyl-CoA:succinate CoA transferase, *ygfH*, increased the titre of erythromycin A sevenfold^63,64^. The use of bioreactors rather than shake flasks increased the titre of 6-dEB fivefold^65^. *E. coli* K207-3 has not been optimized for general polyketide production; however, now that this chassis organism can produce macrolide antibiotics, avenues to evolve it for enhanced polyketide production have been opened^18^. For example, the macrolide-sensing transcription factor MphR could be used to help select for *E. coli* K207-3 variants that produce higher titres of narbomycin^66^. To access higher titres of hydroxylated products such as pikromycin and methymycin, PikC activity could be increased through its fusion to a reductase domain^42^.

While **22** exhibited antibacterial activity comparable to that of narbomycin, **21** and **23** showed weak activity. The crystal structure of pikromycin in complex with the *Thermus thermophilus* 70S ribosome, messenger RNA and transfer RNAs (PDB 5WIT) shows that, in addition to the desosamine moiety making contact with ribosomal RNA, the enone oxygen interacts with A2080 through a hydrogen bond with its ribose hydroxy group^67^. The enone is in an *s-trans* conformation, in contrast to the *s-cis* conformation it adopts in the crystal structure of pikromycin (Cambridge Crystallographic Data Centre (CCDC) deposition number 1109615)^68^. An intriguing possibility is that the narbomycin–pikromycin enone normally reacts as a Michael acceptor with nucleophilic residues, such as cysteines, in nascent polypeptide chains. The weak activity of **21** could be due to it lacking an enone. The weak activity of **23** could be due to the *s-trans* form of its enone being disfavoured due to a steric clash that would occur between the introduced C10 methyl group and the C12 methyl group. As more derivatives of narbomycin and pikromycin are accessed through the described platform, a better understanding of the mechanism of action for these monosaccharide macrolide antibiotics will emerge.

In summary, we have developed a two-plasmid BioBricks-like system to assemble PKSs from updated modules and used it to express a refactored pikromycin PKS in *E. coli*. Through swapping modules and expressing tailoring enzymes from the pikromycin pathway, we modularly synthesized new derivatives of narbonolide, narbomycin and pikromycin as well as 10-dml, YC-17 and methymycin. As many strategies beyond those used here can further boost the yields of macrolide antibiotics, the described platform may be used beyond advancing our understanding of PKS enzymology to access new macrolide antibiotics at preparative levels. Our *E. coli*-based PKS engineering platform is also higher throughput than *Streptomyces*-based platforms^69–71^. We foresee it being used to generate combinatorial libraries of promising polyketide drug leads that will accelerate the medicinal chemistry of these synthetically challenging compounds.

## Methods

### Construction of the two-plasmid system

p**P1**-**P4**^**N**^ was constructed by Gibson assembly and SLiCE (seamless ligation cloning extract)^72^. First, amplicons of **P1** and **P4**^**N**^ were generated by polymerase chain reaction (PCR) using *Streptomyces venezuelae* ATCC 15439 genomic DNA (gDNA) and joined with KpnI/XhoI-digested pCDF-1b by Gibson assembly (all oligonucleotide sequences are provided in Supplementary Data 1). Then, amplicons containing the BmtI site and **E3** ^C^DD, generated using *S. erythraea* NRRL 2338 gDNA as the template, were inserted into the XhoI site through SLiCE assembly.

p^**C**^**P4**-**P7** was constructed by Gibson assembly. An amplicon containing **E3** ^N^DD was initially cloned into NcoI/XhoI-digested pET-28b. The resulting plasmid was used as a PCR template to generate an amplicon containing **E3** ^N^DD. Amplicons containing ^**C**^**P4** and **P7** were generated by PCR using *S. venezuelae* gDNA. These three amplicons were joined by Gibson assembly.

### Construction of the module-cloning plasmids

Most of the module-cloning plasmids were constructed in our previous report^16^. Briefly, the module-cloning plasmids contain DNA encoding AT–ACP and KS separated by a ‘regulator’ in a pUC19 vector. The regulator includes a T7 terminator, a T7 promoter, a lac operator, a ribosomal binding site, an ATG start codon and cognate docking domains that enable the association of PKS polypeptides. Module-containing plasmids were constructed by swapping regulator sequences using the MfeI and BmtI sites. Construction of pUT21-P3, pUT21-P5 and pUT21-P6, which contain the native **P3**, **P5** and **P6** docking domains, respectively, was performed by SLiCE so that the MfeI and BmtI sites are removed. pUT21-P6 (C to A) was constructed through site-directed mutagenesis.

### Construction of hexaketide, heptaketide and octaketide synthases

HindIII/XbaI-digested, module-containing fragments were inserted between the HindIII and SpeI sites in both p**P1**-**P4**^**N**^ and p^**C**^**P4**-**P7**. As the insertions preserve the HindIII-N_12_-SpeI sequence, subsequent insertions of module-containing fragments can be performed.

### Construction of the desosamine biosynthesis/transfer operon

DNA containing the d-desosamine biosynthetic genes, the d-desosamine transfer genes and *pikC* was amplified by PCR using *S. venezuelae* gDNA as the template (Supplementary Fig. 32). The erythromycin resistance gene *ermE* was also amplified by PCR using *S. erythraea* NRRL 2338 gDNA as the template. Each amplicon contains an SpeI site after the stop codon to enable XbaI/SpeI ligation as described below. DNA containing *desI* was cloned between the BamHI and EcoRI sites of pACYCDuet-1, while DNA containing the other genes was cloned between the NdeI and EcoRI sites of pET-28b. To construct pDes, the plasmid containing the d-desosamine biosynthesis/transfer pathway, DNA containing the d-desosamine biosynthesis/transfer genes and *ermE* were combined into a single operon through the sequential ligation of XbaI/EcoRI inserts into the SpeI/EcoRI-digested pACYC-derived vector. To generate pDesPikC, an equivalently constructed *pikC* insert was further ligated into pDes. To construct pDesPikAV, the d-desosamine biosynthetic operon with *ermE* was amplified by PCR using pDes as a template and cloned between the BamHI and EcoRI sites of pACYC-pikAV. To express the d-desosamine biosynthetic genes from a bacterial artificial chromosome, the operon was cloned between the BmtI and AvrII sites of pMKBAC02^44^.

### Construction of PikAV expression vectors

*pikAV* was amplified by PCR using *S. venezuelae* gDNA as the template and cloned between the EcoRI and HindIII sites of pACYCDuet-1 to yield pACYC-pikAV. One more copy of *pikAV* was cloned between the NdeI and XhoI sites to yield pACYC-pikAV-pikAV. To generate a set of pikAV expression plasmids with different expression levels, site-directed mutagenesis was performed.

### Genomic integration of the desosamine biosynthesis/transfer operon

The genetic manipulation of *E. coli* K207-3 was performed with the CRISPR–Cas9 genome-editing tool pEcCas/pEcgRNA^73,74^. To target the *cadA* locus for genome integration, CATCGCCGCAGCGGTTTCAGTGG was selected as the target sequence. Oligonucleotides TAGTCATCGCCGCAGCGGTTTCAG and AAACCTGAAACCGCTGCGGCGATG were synthesized, annealed and ligated into the BsaI site of pEcgRNA to yield pEcgRNA-cadA.

Possibly because each gene in the d-desosamine biosynthesis/transfer + *ermE* operon in pDes is preceded by an equivalent 104-base-pair (bp) sequence, homologous recombination occurred when λ Red recombinase was expressed. To minimize the length of these sequences, each gene was amplified to include the minimal sequence (AAGGAGATATACCATG) and combined by overlap extension PCR.

The 1,000-bp homology arms homologous to the upstream and downstream regions of the *cadA* locus were prepared by PCR using *E. coli* K207-3 gDNA as the template. The homology arms were combined by overlap extension PCR such that an XbaI site was created between them. The resulting amplicon was ligated between the EcoRI and HindIII sites of pEcgRNA-cadA to yield pEcgRNA-ΔcadA. The d-desosamine synthesis operon was ligated into the XbaI site of the resulting plasmid to yield pEcgRNA-ΔcadA:desI,II,V,VI,VII,VIII,ermE. As linear DNA was found to be more effective in guiding homologous recombination, it was generated by PCR using pEcgRNA-ΔcadA:desI,II,V,VI,VII,VIII,ermE as the template.

*E. coli* K207-3 harbouring pEcCas was shaken at 240 rpm in 6 ml of Luria–Bertani (LB) media containing 50 mg l^−1^ kanamycin at 37 °C. Then 1 ml of the culture was inoculated into 50 ml of LB media containing 50 mg l^−1^ kanamycin. Cells were shaken until an optical density at 600 nm of 0.2 was reached, and 10 mM arabinose was added to induce expression of the λ Red system. When optical density at 600 nm was 0.6, cells were collected by centrifugation and washed with 50 ml of ice-cold 10% (v/v) glycerol. After centrifugation, the cells were resuspended in 200 μl of ice-cold 10% (v/v) glycerol. Then 100 μl of the cells were mixed with 1 μg of the donor DNA and 100 ng of pEcgRNA-*cadA* and then electroporated in a 0.1-cm-gap Gene Pulser cuvette at 1.6 kV. The positive colonies were selected on an LB plate containing 50 mg l^−1^ kanamycin and streptomycin at 37 °C. Genome integration was confirmed by colony PCR.

To cure pEcgRNA-*cadA* from the cells, single colonies were picked and grown at 240 rpm in 2 ml of LB media containing 50 mg l^−1^ kanamycin and 10 mM rhamnose at 37 °C for 8 h. Then 200 μl of each culture was added to 2 ml of LB media, which was shaken at 240 rpm at 37 °C for 3 h. Cells were spread on LB plates containing 5 g l^−1^ glucose and 5 g l^−1^ sucrose and incubated overnight at 37 °C. Colonies sensitive to both kanamycin and streptomycin were selected.

### Inactivation of pathways competitive with desosamine biosynthesis

CRISPR–Cas9 genome editing was performed as described above. To inactivate *vioAB*, *rmlC* and *wecDE*, CATCGCCGCAGCGGTTTCAG, CTATGGAAAAAGGTATAAGA and CGACTATATGCAGTCGGCAA were respectively selected as the target sites. Homology arms homologous to regions 500 bp upstream and downstream of the target sites were prepared by PCR using *E. coli* K207-3 gDNA as the template. These were combined by overlap extension PCR to yield the donor DNA that guides homologous recombination. Next, 600 ng of the donor DNA and 100 ng of the targeting plasmid (pEcgRNA-rmlC, pEcgRNA-vioAB, pEcgRNA-wecDE) were used for each electroporation. Plasmids were cured as above, and gene inactivations were confirmed by colony PCR.

### Polyketide production and quantification

*E. coli* K207-3 (or TM1–7 derivative strains) transformed with PKS expression plasmids were shaken at 240 rpm in 6 ml of LB media containing the appropriate antibiotics (50 mg l^−1^ kanamycin and 50 mg l^−1^ streptomycin, as well as 30 mg l^−1^ chloramphenicol when using the desosamine biosynthesis/transfer plasmids) in a culture tube at 37 °C. From these precultures, 0.3 ml was used to inoculate 30 ml of production media (5 g l^−1^ yeast extract, 10 g l^−1^ casein, 15 g l^−1^ glycerol, 10 g l^−1^ NaCl and 100 mM potassium phosphate, pH 7.6 with appropriate antibiotics)^13^ in 250 ml non-baffled Erlenmeyer flasks. Cells were cultured at 240 rpm at 37 °C until optical density at 600 nm = 0.6. They were then cooled to 16 °C, supplied with 0.1 mM isopropyl-β-d-thiogalactoside and 60 mM sodium propionate, and cultured for 10 days. Then 500 μl of the culture broth was extracted twice with the same volume of ethyl acetate and concentrated in vacuo. The extract was resuspended in 500 μl of methanol, and 1-μl samples were injected into a 6230 time-of-flight LC–MS (solvent A: water with 0.1% (v/v) formic acid; solvent B: acetonitrile with 0.1% (v/v) formic acid; 5–100% B for 15 min, 100% B for 3 min, flow rate of 0.2 ml min^−1^, positive mode, 180 V, 300 °C).

To quantify polyketide production, extracted ion chromatography peak areas or ultraviolet peak areas of the expected products were measured and compared with calibration curves generated by authentic standards. Narbonolide was used to quantify macrolactones, while commercial pikromycin was used to quantify macrolides. The purified product of **P1-P5-P6-P7** was used to quantify δ-lactone products (using 247 nm), while 4-hydroxy-6-methyl-2-pyrone (Sigma-Aldrich) was used for pyrone products (using 285 nm).

### Isolation of polyketide products

Culture broth was extracted twice with the same volume of ethyl acetate, dried with MgSO_4_, filtered and concentrated in vacuo. To extract the 6-membered lactones and macrolides from the culture broth, the pH was adjusted to 3 and 9.5, respectively. The polyketides were purified by flash silica gel chromatography, followed by semipreparative high-performance liquid chromatography.

### Crystallization of 12

Purified **12** was dissolved in a hexane:ethyl acetate ratio of 70:30 at 50 °C, filtered using a cotton plug and evaporated at room temperature overnight.

### MIC assay

The antibacterial activities of the purified compounds were evaluated against several bacterial strains using the Clinical and Laboratory Standards Institute broth microdilution method. The test strains were grown in Mueller–Hinton medium overnight (*B. subtilis*, *S. epidermidis* and *E. coli* were grown at 37 °C, while *M. luteus* and *P. putida* were grown at 30 °C). The broths were diluted by the Mueller–Hinton medium (1:500) and used as an inoculum. Then, 50 μl of the inoculum was mixed with 50 μl of Mueller–Hinton medium containing twofold serial dilutions of the compounds in 96-well plates. After incubating the cells for 24 h at 30 °C or 37 °C, each MIC was determined as the lowest concentration that prevented visible bacterial growth.

### Biological materials

*E. coli* K207-3 cells are available from the authors upon request.

### Reporting summary

Further information on research design is available in the Nature Portfolio Reporting Summary linked to this article.

## Supplementary information

Supplementary Information Experimental details, Supplementary Figs. 1–73 and Tables 1–4.

Reporting Summary

Supplementary Data 1 Oligonucleotides used in this study.

Supplementary Data 2 X-ray crystallographic data for compound **12**, CCDC 2535796.

Supplementary Data 3 Source data for Supplementary Fig. 11. Optimization of narbonolide production.

Supplementary Data 4 Source data for Supplementary Fig. 12. Replacement of the docking domain pairs in the refactored pikromycin PKS and analysis of shunt products.

Supplementary Data 5 Source data for Supplementary Fig. 13. Analysis of the effect of TEII expression level on narbonolide production.

Supplementary Data 6 Source data for Supplementary Fig. 47. PikC oxidation efficiency.

Supplementary Data 7 Source data for Supplementary Table 4. Summary of polyketide production.

## Source data

Source Data Fig. 3 Quantification of macrolactone production in *E. coli*.

Source Data Fig. 6 Quantification of narbomycin production in *E. coli*.

## Data Availability

All data are available in the Supplementary Information. The X-ray crystallographic coordinates for the structure reported in this study (**12**) have been deposited at the CCDC, under deposition number 2535796. These data can be obtained free of charge from the CCDC via www.ccdc.cam.ac.uk/data_request/cif. Source data are provided with this paper.
