# Supplementary material for: Refactoring pikromycin synthase for the modular biosynthesis of macrolide antibiotics in E. coli
Source: Nat Synth. 2026 Jun 29;5(11):1736–47. doi: 10.1038/s44160-026-01108-2 (PMC13739727; doi:10.1038/s44160-026-01108-2)
Supplement: Supplementary file 1 [file 44160_2026_1108_MOESM1_ESM.pdf]

nature synthesis

Supplementary information

https://doi.org/10.1038/s44160-026-01108-2

Refactoring pikromycin synthase for the modular biosynthesis of macrolide antibiotics in *E. coli*

In the format provided by the authors and unedited

Table of Contents

Content	Page
**METHODS**	5
**Figure S1.** Construction of the 2-plasmid system	23
**Figure S2.** Sequences of **P1-**^{N}**P4-E3**^{C}DD and **E3**^{N}DD-^{C}**P4-P7**	24
**Figure S3.** Regulator sequences	28
**Figure S4.** LC/MS analysis of the anticipated **P1-P4-P7** product, **7**	29
**Figure S5.** LC/MS analysis of the anticipated **P1-P2-P3-P4-P7** product, **8**	30
**Figure S6.** LC/MS analysis of the anticipated **P1-P4-P5-P6-P7** product, **9**	32
**Figure S7.** LC/MS analysis of the anticipated **P1-P2-P3-P4-P5-P6-P7** product, narbonolide (**1**)	33
**Figure S8.** ^{1}H NMR spectrum of the anticipated **P1-P2-P3-P4-P5-P6-P7** product, narbonolide (**1**)	34
**Figure S9.** ^{13}C NMR spectrum of the anticipated **P1-P2-P3-P4-P5-P6-P7** product, narbonolide (**1**)	35
**Figure S10.** ^{1}H-^{13}C HSQC spectrum of the anticipated **P1-P2-P3-P4-P5-P6-P7** product, narbonolide (**1**)	36
**Figure S11.** Optimization of narbonolide production	37
**Figure S12.** Replacement of docking domain pairs in refactored pikromycin PKSs and analysis of shunt products	38
**Figure S13.** Analysis of the effect of TEII expression level on narbonolide production	39
**Figure S14.** LC/MS analysis of the anticipated **P1-P2-P5-P4-P5-P6-P7** product, **10**, and byproduct **11**	40
**Figure S15.** ^{1}H NMR spectrum of anticipated **P1-P2-P5-P4-P5-P6-P7** product, **10**	41
**Figure S16.** LC/MS analysis of the anticipated **P1-E2-P3-P4-P5-P6-P7** product, narbonolide (**1**)	42
**Figure S17.** LC/MS analysis of the anticipated **P1-P2-P3-P4-P5-E6-P7** product, narbonolide (**1**)	43
**Figure S18.** LC/MS analysis of the anticipated **P1-R6-P3-P4-P5-P6-P7** product, **12**	44
**Figure S19.** ^{1}H NMR spectrum of the **P1-R6-P3-P4-P5-P6-P7** product, **12**	45
**Figure S20.** ^{13}C NMR spectrum of the **P1-R6-P3-P4-P5-P6-P7** product, **12**	46
**Figure S21.** ^{1}H-^{13}C HSQC spectrum of the **P1-R6-P3-P4-P5-P6-P7** product, **12**	47
**Figure S22.** LC/MS analysis of the anticipated **P1-P2-R5-P4-P5-P6-P7** product, **13** and **14**	48
**Figure S23.** ^{1}H NMR spectrum of the **P1-P2-R5-P4-P5-P6-P7** product, **13**	49

**Figure S24.** ^{13}C NMR spectrum of the **P1-P2-R5-P4-P5-P6-P7** product, **13**	50
**Figure S25.** ^{1}H-^{13}C HSQC spectrum of the **P1-P2-R5-P4-P5-P6-P7** product, **13**	51
**Figure S26.** ^{1}H NMR spectrum of the **P1-P2-R5-P4-P5-P6-P7** product, **14**	52
**Figure S27.** ^{13}C NMR spectrum of the **P1-P2-R5-P4-P5-P6-P7** product, **14**	53
**Figure S28.** ^{1}H-^{13}C HSQC spectrum of the **P1-P2-R5-P4-P5-P6-P7** product, **14**	54
**Figure S29.** LC/MS analysis of the anticipated **P1-P2-P3-P4-P5-P6*-P7** product, 10-dml (**4**)	55
**Figure S30.** ^{1}H NMR spectrum of the anticipated **P1-P2-P3-P4-P5-P6*-P7** product, **4** (10-dml)	56
**Figure S31.** ^{13}C NMR spectrum of the anticipated **P1-P2-P3-P4-P5-P6*-P7** product, **4** (10-dml)	57
**Figure S32.** ^{1}H-^{13}C HSQC spectrum of the anticipated **P1-P2-P3-P4-P5-P6*-P7** product, **4** (10-dml)	58
**Figure S33.** LC/MS analysis of the anticipated **P1-P2-P5-P4-P5-P6*-P7** product, **15**	59
**Figure S34.** LC/MS analysis of the anticipated **P1-R6-P3-P4-P5-P6*-P7** product, **16**	60
**Figure S35** LC/MS analysis of the anticipated **P1-P2-R5-P4-P5-P6*-P7** product, **17**	61
**Figure S36.** LC/MS analysis of the anticipated **P1-P2-P4-P5-P6-P7** product, **18**	62
**Figure S37.** ^{1}H NMR spectrum of the anticipated **P1-P2-P4-P5-P6-P7** product, **18**	63
**Figure S38.** ^{13}C NMR spectrum of the anticipated **P1-P2-P4-P5-P6-P7** product, **18**	64
**Figure S39.** ^{1}H-^{13}C HSQC spectrum of the anticipated **P1-P2-P4-P5-P6-P7** product, **18**	65
**Figure S40.** LC/MS analysis of the anticipated **P1-P2-P3-P4-P5-P5-P6-P7** product, **20**	66
**Figure S41.** Construction of D-desosamine biosynthesis/transfer pathway	67
**Figure S42.** LC/MS analysis of the anticipated **P1-P2-P3-P4-P5-P6-P7**+Des product, narbomycin (**2**)	68
**Figure S43.** ^{1}H NMR spectrum of the anticipated **P1-P2-P3-P4-P5-P6-P7**+Des product, narbomycin (**2**)	69
**Figure S44.** ^{13}C NMR spectrum of the anticipated **P1-P2-P3-P4-P5-P6-P7**+Des product, narbomycin (**2**)	70
**Figure S45.** ^{1}H-^{13}C HSQC spectrum of the anticipated **P1-P2-P3-P4-P5-P6-P7**+Des product, narbomycin (**2**)	71
**Figure S46.** LC/MS analysis of the anticipated **P1-P2-P3-P4-P5-P6-P7**+Des+PikC product, pikromycin (**3**)	72
**Figure S47.** Efficiency of conversion of narbomycin to pikromycin by PikC	73
**Figure S48.** LC/MS analysis of the anticipated **P1-P2-P3-P4-P5-P6*-P7**+Des product, YC-17 (**5**)	74

**Figure S49.** LC/MS analysis of the anticipated **P1-P2-P3-P4-P5-P6*-P7**+Des+PikC product, methymycin (**6**)	75
**Figure S50.** ^{1}H NMR spectrum of the anticipated **P1-P2-P3-P4-P5-P6*-P7**+Des product, YC-17 (**5**)	76
**Figure S51.** ^{13}C NMR spectrum of the anticipated **P1-P2-P3-P4-P5-P6*-P7**+Des product, YC-17 (**5**)	77
**Figure S52.** ^{1}H-^{13}C HSQC spectrum of the anticipated **P1-P2-P3-P4-P5-P6*-P7**+Des product, YC-17 (**5**)	78
**Figure S53.** LC/MS analysis of the anticipated **P1-P2-P5-P4-P5-P6-P7**+Des product, **21**	79
**Figure S54.** LC/MS analysis of the anticipated **P1-R6-P3-P4-P5-P6-P7**+Des product, **22**	80
**Figure S55.** LC/MS analysis of the anticipated **P1-P2-R5-P4-P5-P6-P7**+Des product, **23**	81
**Figure S56.** LC/MS analysis of the anticipated **P1-R6-P3-P4-P5-P6*-P7**+Des product, **24**	82
**Figure S57.** LC/MS analysis of the anticipated **P1-P2-R5-P4-P5-P6-P7**+Des product, **25**.	83
**Figure S58.** ^{1}H NMR spectrum of the anticipated **P1-P2-P5-P4-P5-P6-P7**+Des product, **21**	84
**Figure S59.** ^{13}C NMR spectrum of the anticipated **P1-P2-P5-P4-P5-P6-P7**+Des product, **21**	85
**Figure S60.** ^{1}H-^{13}C HSQC spectrum of the anticipated **P1-P2-P5-P4-P5-P6-P7**+Des product, **21**	86
**Figure S61.** ^{1}H NMR spectrum of the anticipated **P1-R6-P3-P4-P5-P6-P7**+Des product, **22**	87
**Figure S62.** ^{13}C NMR spectrum of the anticipated **P1-R6-P3-P4-P5-P6-P7**+Des product, **22**	88
**Figure S63.** ^{1}H-^{13}C HSQC spectrum of the anticipated **P1-R6-P3-P4-P5-P6-P7**+Des product, **22**	89
**Figure S64.** ^{1}H NMR spectrum of the anticipated **P1-P2-R5-P4-P5-P6-P7**+Des product, **23**	90
**Figure S65.** ^{13}C NMR spectrum of the anticipated **P1-P2-R5-P4-P5-P6-P7**+Des product, **23**	91
**Figure S66.** ^{1}H-^{13}C HSQC spectrum of the anticipated **P1-P2-R5-P4-P5-P6-P7**+Des product, **23**	92
**Figure S67.** Integration of the desosamine biosynthesis/transfer (Des) operon into *cadA* locus of *E. coli* K207-3	93

**Figure S68.** Inactivation of pathways competing with desosamine biosynthesis	95
**Figure S69.** MIC assay	96
**Figure S70.** Calibration curve for quantification of narbonolide, 10-dml, and their derivatives	97
**Figure S71.** Calibration curve for quantification of narbomycin, pikromycin, YC-17, methymycin, and their derivatives	97
**Figure S72.** Calibration curve for quantification of pyrone products.	98
**Figure S73.** Calibration curve for quantification of δ-lactone products	98
**Table S1.** Strains used in this study	99
**Table S2.** Plasmids used in this study	100
**Table S3.** Crystal data and structure refinement for **12**	104
**Table S4.** Summary of polyketide production	105
**REFERENCES**	107

METHODS

Reagents and equipment

KOD One PCR master mix is from Toyobo. The KAPA HiFi DNA polymerase is from KAPA Biosystems. All restriction enzymes are from New England Biolabs. All primers are from Sigma-Aldrich. All gBlocks are from IDT. Luria-Bertani (LB) Miller Broth, sodium phosphate, potassium phosphate, sodium chloride, sodium propionate, and glycerol are from Fisher Scientific. Casein is from Spectrum. Yeast extract is from Gibco. Streptomycin sulfate, kanamycin sulfate, ampicillin sodium salt, chloramphenicol, and apramycin sulfate are from VWR. Isopropyl-β-D-thio-galactopyranoside (IPTG) is from Carbosynth. Ethyl acetate (EtOAc), acetonitrile, methanol, chloroform, and hexanes are from Fisher Scientific. $CDCl_3$ is from Cambridge Isotope Laboratories. SiliaFlash Silica Gel is from SiliCycle. Pikromycin is from AdipoGene. All strains, plasmids, and oligonucleotides are reported (Supplementary Tables 1–2 and Supplementary Data 1).

High-resolution LC/MS data was obtained with an Agilent 6230 TOF LC/MS connected to a ZORBAX Eclipse Plus C18 column (50 x 2.1 mm, 1.8 μm). NMR data were obtained on Bruker AVANCE III 500 NMR or Bruker NEO 600 NMR spectrometers.

Construction of the 2-plasmid system

p**P1**-**P4**^{N} was constructed by Gibson assembly and SliCE (Figure S1a)[1]. First, amplicons of **P1** and **P4**^{N} were generated by PCR using *Streptomyces venezuelae* ATCC 15439 genomic DNA and joined with KpnI/XhoI-digested pCDF-1b by Gibson assembly. Then, amplicons containing the BmtI site and **E3** ^{C}DD, generated using *Saccharopolyspora erythraea* NRRL 2338 genomic DNA as the template, were inserted into the XhoI site through SLiCE assembly.

p^{C}**P4**-**P7** was constructed by Gibson assembly (Figure S1b). An amplicon containing **E3** ^{N}DD was initially cloned into NcoI/XhoI-digested pET-28b. The resulting plasmid was used as a PCR template to generate an amplicon containing **E3** ^{N}DD. Amplicons containing ^{C}**P4** and **P7** were generated by PCR using *S. venezuelae* gDNA. These 3 amplicons were joined by Gibson assembly.

The sequences of p**P1**-**P4**^{N} and p^{C}**P4**-**P7** are reported (Figure S2).

Construction of the module-cloning plasmids

Most of the module-cloning plasmids were constructed in our previous report[2]. Briefly, the module-cloning plasmids contain DNA encoding the AT, processing enzymes, and ACP, and DNA encoding KS, separated by a "regulator" in a pUC19 vector. The regulator includes a T7 terminator, a T7 promoter, a lac operator, a ribosomal binding site, an ATG start codon, and cognate docking domains that enable the association of PKS polypeptides. Since the construction of heptaketide and octaketide synthases requires 5 and 6 sets of docking domain pairs, respectively, more regulators were created using gBlocks containing the **E3** and **A1** docking domains. Module-containing plasmids were constructed by swapping regulator sequences using the MfeI and BmtI sites. Construction of pUT21-P3, pUT21-P5, pUT21-P6, which respectively contain the native **P3**, **P5**, and **P6** docking domains, was performed by SLiCE so that the MfeI and BmtI sites are replaced by DNA encoding the native amino acids. pUT21-P6 (C to A) was constructed through site-directed mutagenesis. The gBlock sequences used to construct the regulators are provided (Figure S3).

Construction of hexaketide, heptaketide, and octaketide synthases

HindIII/XbaI-digested module-containing fragments were inserted between the HindIII and SpeI sites in both p**P1**-**P4**^{N} and p^{C}**P4**-**P7**. Since the insertions preserve the HindIII-N_{12}-SpeI sequence upstream of the inserted module and generate an inactive SpeI/XbaI site downstream of the inserted module, subsequent insertions of module-containing fragments can be performed.

Construction of operons encoding desosamine biosynthesis/transfer and macrolide antibiotic resistance enzymes

The DNA encoding *desI*, *desII*, *desV*, *desVI* (D-desosamine biosynthesis); *desVII*, *desVIII* (D-desosamine transfer); and *pikC* (P450 monooxygenase that hydroxylates narbomycin and YC-17) was amplified by PCR using *S. venezuelae* gDNA as the template (Figure S32). The erythromycin resistance gene *ermE* was also amplified by PCR using *Saccharopolyspora erythraea* NRRL 2338 gDNA as the template. Each amplicon contains an SpeI site after the stop codon to enable XbaI/SpeI ligation as described below. The DNA encoding *desI* was cloned between the BamHI and EcoRI sites of pACYCDuet-1, while the DNA encoding each of *desII*, *desV*, *desVI*, *desVII*, *desVIII*, and *ermE* was cloned between the NdeI and EcoRI sites of pET-28b. To construct pDes, the plasmid encoding the D-desosamine biosynthesis/transfer pathway, DNA encoding *desI*, *desII*, *desV*, *desVI*, *desVII*, *desVIII*, and *ermE* was combined into single operon through the sequential ligation of XbaI/EcoRI inserts into the SpeI/EcoRI-digested pACYC-derived vector. To generate pDesPikC, an equivalently-constructed *pikC* insert was further ligated into pDes. To construct pDesPikAV, the D-

desosamine biosynthetic operon with *ermE* was amplified by PCR using pDes as a template and cloned between BamHI and EcoRI sites of pACYC-pikAV.

To express the D-desosamine biosynthetic genes from a BAC (copy number = 1 or 2), the operon was amplified by PCR and cloned between the BmtI and AvrII sites of pMKBAC02[3].

Construction of PikAV expression vectors

pikAV was amplified by PCR using *S. venezuelae* gDNA as the template and cloned between the EcoRI and HindIII sites of pACYCDuet-1 to yield pACYC-pikAV. One more copy of *pikAV* was cloned between the NdeI and XhoI sites to yield pACYC-pikAV-pikAV. To generate a set of pikAV expression plasmids with different expression levels, site-directed mutagenesis of the T7 promoters of pACYC-pikAV and pACYC-pikAV-pikAV was performed (Figure S13).

Integration of the D-desosamine biosynthesis/transfer pathway and *ermE* into the *E. coli* K207-3 genome

The genetic manipulation of *E. coli* K207-3 was performed with CRISPR/Cas9 genome editing tool pEcCas/pEcgRNA (Figure S67)[4,5]. To target the *cadA* locus for genome integration, CATCGCCGCAGCGGTTTCAGTGG was selected as the target sequence. Oligonucleotides TAGTCATCGCCGCAGCGGTTTCAG and AAACCTGAAACCGCTGCGGCGATG were synthesized, annealed, and ligated into BsaI site of pEcgRNA to yield pEcgRNA-cadA.

Likely because each gene in the D-desosamine biosynthesis/transfer+*ermE* operon in pDes is preceded by an equivalent 104 bp sequence encoding a His-tag and ribosome binding site (from pET-28b), homologous recombination occurred when λ-red recombinase was expressed. To minimize the length of these equivalent sequences, each gene was amplified to include the minimal sequence (AAGGAGATATACCATG) and combined by overlap extension PCR to yield pEcgRNA-cadA.

1000 bp homology arms homologous to the upstream and downstream regions of the *cadA* locus were prepared by PCR using *E. coli* K207-3 gDNA as the template. The homology arms were combined by overlap extension PCR such that an XbaI site was created between them. The resulting amplicon was ligated between the EcoRI and HindIII sites of pEcgRNA-cadA to yield pEcgRNA-ΔcadA. The D-desosamine synthesis operon was ligated into the XbaI site of the resulting plasmid to yield pEcgRNA-ΔcadA:desI,II,V,VI,VII,VIII,ermE. As linear DNA was found to be more effective guiding homologous recombination, it was generated by PCR using pEcgRNA-ΔcadA:desI,II,V,VI,VII,VIII,ermE as the template.

E. coli K207-3 harboring pEcCas was shaken at 240 rpm in 6 mL of LB media containing 50 mgL^{-1} kanamycin at 37 ºC. 1 mL of the culture was inoculated into 50 mL of LB media containing 50 mgL^{-1} kanamycin. Cells were shaken until OD_{600} = 0.2, and 10 mM arabinose was added to induce expression of the λ-red system. When OD_{600} = 0.6, cells were collected by centrifugation (4000 × g for 5 min, 4 ºC) and washed with 50 mL of ice-cold 10% (v/v) glycerol. After centrifugation (4000 × g for 5 min, 4 ºC), the cells were resuspended in 200 μL of ice-cold 10% (v/v) glycerol. 100 μL of the cells were mixed with 1 μg of the donor DNA and 100 ng of pEcgRNA-*cadA* and then electroporated in a 0.1 cm-gap Gene Pulser cuvette (FisherBio) at 1.6 kV. 1 mL of LB medium was immediately added to the mixture after electroporation. The cells were allowed to recover at 37 ºC for 1 h before plating. The positive colonies were selected on an LB plate containing 50 mgL^{-1} kanamycin and streptomycin at 37 ºC. Genome integration was confirmed by colony PCR (Figure S62).

To cure pEcgRNA-*cadA* from the cells, single colonies of transformants were picked and grown in 2 mL of LB media containing 50 mgL^{-1} kanamycin and 10 mM rhamnose at 37 ºC and 240 rpm for 8 h. 200 μL of the cultures were inoculated into 2 mL of LB media and shaken at 240 rpm at 37 ºC for 3 h. To identify cells without pEcCas, they were spread on LB plates containing 5 gL^{-1} glucose and 5 gL^{-1} sucrose and incubated overnight at 37 ºC. Colonies sensitive to both kanamycin and streptomycin were selected – 3 out of 20 colonies grew in LB media without these antibiotics but did not in media with these antibiotics.

Inactivation of pathways competitive with D-desosamine biosynthesis

CRISPR/Cas9 genome-editing was performed as described above. To inactivate *vioAB*, *rmlC*, and *wecDE*, CATCGCCGCAGCGGTTTCAG, CTATGGAAAAAGGTATAAGA, and CGACTATATGCAGTCGGCAA were respectively selected as the target sites. Homology arms homologous to regions 500 bp upstream and downstream of the target sites were prepared by PCR using *E. coli* K207-3 gDNA as the template. These were combined by overlap extension PCR to yield the donor DNA that guides homologous recombination. 600 ng of the donor DNA and 100 ng of the targeting plasmid (pEcgRNA-rmlC, pEcgRNA-vioAB, pEcgRNA-wecDE) were used for each electroporation. Plasmids were cured as above, and gene inactivations were confirmed by colony PCR (Figure S68).

Polyketide production and quantification

E. coli K207-3 (or TM1–7 derivative strains) transformed with PKS expression plasmids were shaken at 240 rpm in 6 mL LB media containing the appropriate antibiotics (50 mgL^{-1} kanamycin and 50 mgL^{-1} streptomycin, as well as 30 mgL^{-1} chloramphenicol when employing

the desosamine biosynthesis/transfer plasmids) in a culture tube at 37 ºC. From these precultures, 0.3 mL was used to inoculate 30 mL of production media (5 gL^{-1} yeast extract, 10 gL^{-1} casein, 15 gL^{-1} glycerol, 10 gL^{-1} NaCl, and 100 mM potassium phosphate, pH 7.6 with appropriate antibiotics)[6] in 250 mL non-baffled Erlenmeyer flasks. Cells were cultured at 240 rpm at 37 ºC until OD_{600} = 0.6. They were then cooled to 16 ºC, supplied with 0.1 mM IPTG and 60 mM sodium propionate, and cultured for 10 d. 500 μL of the culture broth was extracted twice with the same volume of ethyl acetate and concentrated in *vacuo.* The extract was resuspended in 500 μL of methanol, and 1 μL samples were injected into a 6230 TOF LC/MS connected to a ZORBAX Eclipse Plus C18 column (2.1 x 50 mm, 1.8 μm) [Solvent A: water with 0.1% (v/v) formic acid; Solvent B: acetonitrile with 0.1% (v/v) formic acid. 5-100% B for 15 min, 100% B for 3 min, flow rate of 0.2 mL min^{-1}, positive mode, 180 V, 300 ºC].

To quantify polyketide production, EIC peak areas or UV peak areas of the expected products were measured and compared with calibration curves generated by authentic standards (Figures S70–73, Table S4). A calibration curve generated using purified narbonolide was employed to quantify macrolactones, while a calibration curve generated using commercial pikromycin was employed to quantify macrolides (Figs. S70–71). A calibration curve generated using the purified product of **P1-P5-P6-P7** was employed for δ-lactone products (using 247 nm), while a calibration curve generated using 4-hydroxy-6-methy-2-pyrone (purchased from Sigma-Aldrich) was employed for pyrone products (using 285 nm) (Figs. S72–73).When necessary, extracts were diluted with MeOH before quantification (10x dilution for narbomycin, pikromycin, and YC-17). 1 μL samples were injected into a LC/MS to measure EIC peak areas, while 10 μL samples were injected into a LC/MS to measure UV peak areas.

Isolation of narbonolide (1)

600 mL of culture broth from K207-3 cells expressing **P1**-**P2**-**P3**-**P4**-**P5**-**P6**-**P7** was extracted twice with the same volume of ethyl acetate, dried with $MgSO_4$, filtered, and concentrated *in vacuo.* The extract was subjected to a flash silica gel chromatography (hexanes:ethyl acetate = 70:30). Fractions containing narbonolide were further purified by semi-preparative HPLC [Waters 1525 HPLC system equipped with a Microsorb 300-5 C18 Dynamax column (250 × 10.0 mm) with a flow rate of 4 mL min^{-1} [solvent A, water with 0.1% (v/v) formic acid; solvent B, acetonitrile with 0.1% (v/v) formic acid. 30–100% B for 15 min] to obtain 8.2 mg of narbonolide after lyophilization.

Theoretical $[M+H]^+$ *m/z* of **1,** 353.2322; observed *m/z*, 353.2310 (-3.3 ppm).

^{1}H NMR (600 MHz, $CDCl_3$) δ 6.90 (dd, *J* = 16.3, 4.8 Hz, 1H, H-11), 6.10 (dd, *J* = 16.3, 1.9 Hz, 1H, H-10), 5.17 – 5.12 (m, 1H, H-13), 3.86 (q, *J* = 5.2 Hz, 1H, H-5), 3.71 (q, *J* = 7.0 Hz, 1H, H-2), 3.04 (q, *J* = 6.9 Hz, 1H, H-8), 2.73 – 2.64 (m, 1H, H-4), 2.11 (d, *J* = 5.1 Hz, 1H, H-5), 1.71 (m, 2H, H-6, H-7a), 1.66 – 1.57 (m, 2H, H-14ab), 1.53 – 1.46 (m, 1H, H-7b), 1.36 (d, *J* = 7.0 Hz, 3H, H-16), 1.14 (m, 6H, H-19, H-17), 1.10 (d, *J* = 6.7 Hz, 3H, H-18), 0.96 – 0.90 (m, 6H, H-20, H-15).

^{13}C NMR (151 MHz, $CDCl_3$) δ 207.61 (C3), 205.06 (C9), 171.06 (C1), 148.52 (C11), 129.00 (C10), 78.09 (C13), 72.61 (C5), 50.28 (C2), 50.23 (C4), 39.63 (C4), 38.86 (C5), 36.45 (C12), 35.02 (C6), 24.22 (C14), 18.62 (C18), 18.31 (C19), 14.33 (C16), 10.87 (C17), 10.70 (C20), 10.41 (C15).

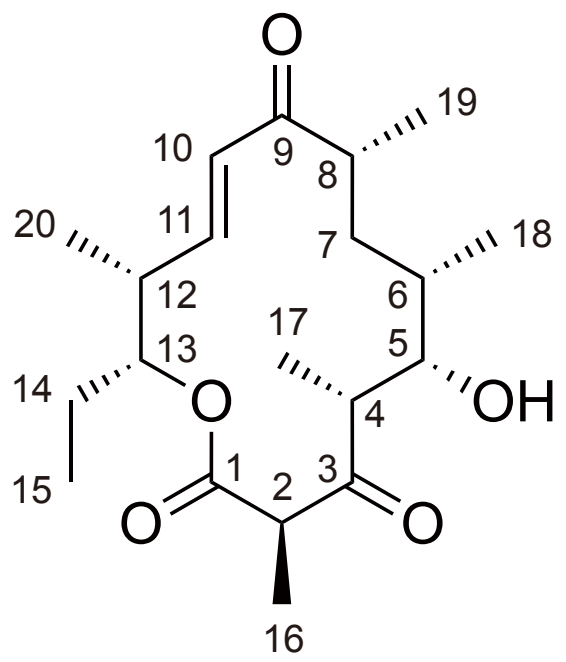

narbonolide (**1**)

Isolation of 10-deoxymethynolide (4)

300 mL of culture broth from *E. coli* K207-3 cells expressing **P1**-**P2**-**P3**-**P4**-**P5**-**P6***-**P7** was extracted twice with the same volume of ethyl acetate, dried with $MgSO_4$, filtered, and concentrated *in vacuo.* The extract was subjected to flash silica gel chromatography (hexanes:ethyl acetate = 70:30). Fractions containing 10-dML were further purified by semi-preparative HPLC [Waters 1525 HPLC system equipped with a Microsorb 300-5 C18 Dynamax column (250 × 10.0 mm) with a flow rate of 4 mL min^{-1} [solvent A, water with 0.1% (v/v) formic acid; solvent B, acetonitrile with 0.1% (v/v) formic acid. 30–100% B for 15 min] to obtain 7.2 mg of 10-dml after lyophilization.

Theoretical $[M+H]^+$ *m/z* of **4**, 297.2060; observed *m/z*, 297.2063 (1.0 ppm).

^{1}H NMR (500 MHz, $CDCl_3$) δ 6.77 (dd, *J* = 15.7, 5.4 Hz, 1H, H-9), 6.44 (dd, *J* = 15.7, 1.3 Hz, 1H, H-8), 5.02 (ddd, *J* = 8.8, 5.5, 2.4 Hz, 1H, H-11), 3.58 (d, *J* = 10.4 Hz, 1H, H-3), 2.71 – 2.59 (m, 2H, H-10, H-2), 2.59 – 2.50 (m, 1H, H-6), 1.80 – 1.64 (m, 3H, H-12ab, H-5a), 1.37 – 1.28 (m, 5H, H-4, H-5b, H-14), 1.24 (d, *J* = 7.0 Hz, 3H, H-16), 1.14 (d, *J* = 6.8 Hz, 3H, H-17), 1.03 (d, *J* = 6.2 Hz, 3H, H-15), 0.94 (t, *J* = 7.4 Hz, 3H, H-13).

^{13}C NMR (126 MHz, $CDCl_3$) δ 204.98 (C7), 174.76 (C1), 147.14 (C9), 125.67 (C8), 78.25 (C3), 73.73 (C11), 45.17 (C6), 43.37 (C2), 38.06 (C10), 33.24 (C5), 33.22 (C4), 25.17 (C12), 17.72 (C16), 17.47 (C15), 16.42 (C14), 10.34 (C13), 9.59 (C17).

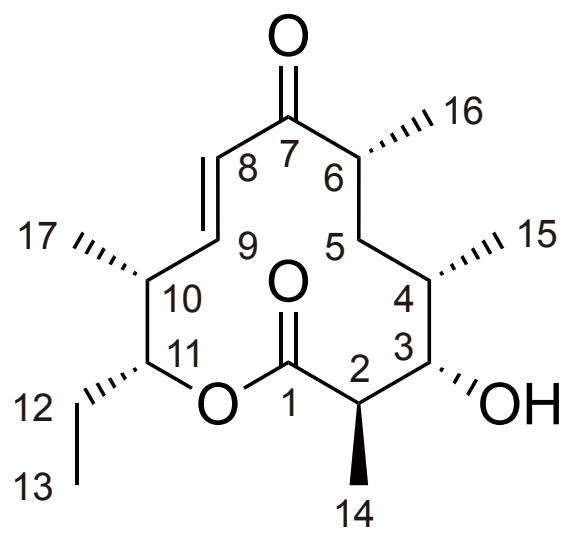

10-dml (**4**)

Isolation of 10

900 mL of culture broth from *E. coli* K207-3 cells expressing **P1**-**P2**-**P5**-**P4**-**P5**-**P6**-**P7**+PikAV was extracted twice with the same volume of ethyl acetate, dried with $MgSO_4$, filtered, and concentrated *in vacuo.* The extract was subjected to flash silica gel chromatography (hexanes:ethyl acetate = 70:30 to 40:60). Fractions containing **10** were further purified by HPLC [Waters 1525 HPLC system equipped with a Microsorb-MV 300-5 C_{18} column (250 × 4.6 mm) with a flow rate of 1 mL min^{-1} (solvent A, water with 0.1% formic acid; solvent B, acetonitrile with 0.1% formic acid. 10–90% B for 20 min)] to obtain 0.6 mg of **10** after lyophilization.

Theoretical $[M+H]^+$ *m/z* of **10**, 369.2635; observed *m/z*, 369.2632 (-0.8 ppm).

^{1}H NMR (400 MHz, $cdcl_3$) δ 5.10 (dq, *J* = 7.8, 2.5 Hz, 1H, H13), 3.97 (q, *J* = 3.6 Hz, 1H, H-5), 3.82 – 3.73 (m, 1H, H-2), 2.86 (d, *J* = 3.3 Hz, 1H, H-4), 2.84 – 2.76 (m, 1H, H-8), 2.72 (m, 1H, H-10), 2.63 (m, 1H, H-6), 1.96 – 1.61 (m, 5H, H-12, H-11ab, H-14ab), 1.49 – 1.27 (m, 2H, H-7ab), 1.36 (d, *J* = 7.0 Hz, 3H, H-16), 1.17 (d, *J* = 7.2 Hz, 3H, H-17), 1.07 – 0.94 (m, 12H, H-18, H-19,H20, H21), 0.90 (t, *J* = 7.6 Hz, 3H, H-15).

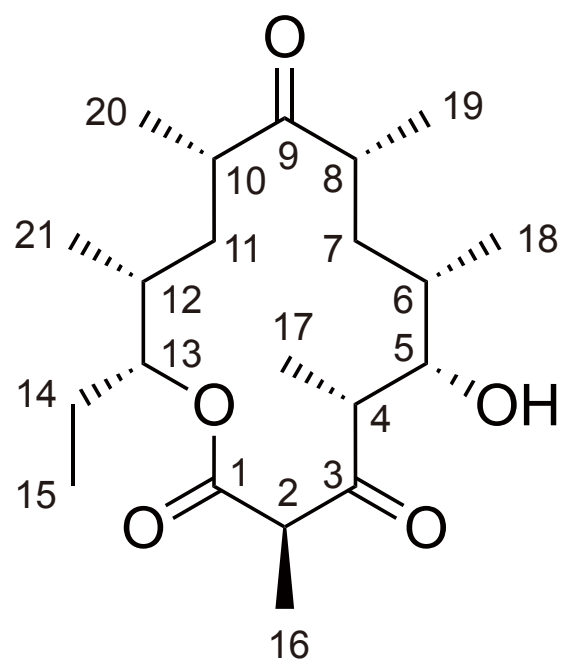

10

Isolation of 12

900 mL of culture broth from K207-3 cells expressing **P1**-**R6**-**P3**-**P4**-**P5**-**P6**-**P7** was extracted twice with the same volume of ethyl acetate, dried with $MgSO_4$, filtered, and concentrated *in vacuo.* The extract was subjected to a flash silica gel chromatography (hexanes:ethyl acetate = 70:30). Fractions containing the expected product were further purified by semi-preparative HPLC [Waters 1525 HPLC system equipped with an Atlantis T3 OBST prep column (250 × 10.0 mm) with a flow rate of 4 mL min^{-1} [solvent A, water with 0.1% (v/v) formic acid; solvent B, acetonitrile with 0.1% (v/v) formic acid. 40% B isocratic elution] to obtain 3.2 mg of white powder after lyophilization.

Theoretical $[M+H]^+$ *m/z* of **12**, 339.2166; observed *m/z*, 339.2166 (0 ppm).

^{1}H NMR (500 MHz, C_6D_6) δ 6.53 (ddd, *J* = 15.9, 9.4, 4.9 Hz, 1H, H-11), 5.98 (d, *J* = 16.0 Hz, 1H, H-10), 4.72 (td, *J* = 8.8, 4.6 Hz, 1H, H-13), 3.82 – 3.72 (m, 1H, H-5), 3.29 (q, *J* = 7.0 Hz, 1H, H-2), 3.08 – 2.99 (m, 1H, H-8), 2.44 – 2.35 (m, 1H, H-4), 1.88 – 1.75 (m, 2H, H-12ab), 1.75 – 1.62 (m, 2H, H-6, H-7a), 1.60 – 1.49 (m, 1H, H-7b), 1.24 (d, *J* = 7.1 Hz, 3H, H-16), 1.15 (d, *J* = 6.7 Hz, 1H, H-14a), 1.13 – 1.07 (m, 1H, H-14b), 1.03 (d, *J* = 6.8 Hz, 3H, H-17), 1.00 (d, *J* = 7.0 Hz, 3H, H-19), 0.88 (d, *J* = 7.0 Hz, 3H, H-18), 0.61 (t, *J* = 7.4 Hz, 3H, H-15).

^{13}C NMR (126 MHz, C_6D_6) δ 206.27 (C3), 202.94 (C9), 170.67 (C1), 141.64 (C11), 131.32 (C10), 74.16 (C13), 72.72 (C5), 50.57 (C4), 50.15 (C8), 37.27 (C12), 36.19 (C7), 34.85 (C6), 27.15 (C14), 18.39 (C18), 18.19 (C17), 14.50 (C16), 10.44 (C19), 9.39 (C15).

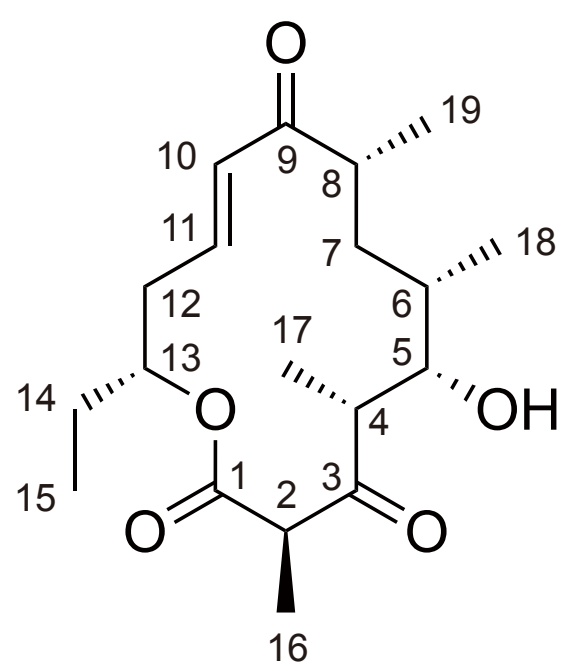

12

Isolation of 13

1.8 L of culture broth from K207-3 cells expressing **P1**-**P2**-**R5**-**P4**-**P5**-**P6**-**P7** was extracted twice with the same volume of ethyl acetate, dried with $MgSO_4$, filtered, and concentrated *in vacuo.* The extract was subjected to a flash silica gel chromatography (hexanes:ethyl acetate = 70:30). Fractions containing the expected product were further purified by semi-preparative HPLC [Waters 1525 HPLC system equipped with an Atlantis T3 OBST prep column (250 × 10.0 mm) with a flow rate of 4 mL min^{-1} [solvent A, water with 0.1% (v/v) formic acid; solvent B, acetonitrile with 0.1% (v/v) formic acid. 45% B isocratic elution] to obtain 1.1 mg of **13** after lyophilization.

Theoretical $[M+H]^+$ *m/z* of **13**, 367.2479; observed *m/z*, 367.2487 (2.1 ppm).

^1H NMR (500 MHz, $CDCl_3$) δ 6.49 (d, *J* = 8.4 Hz, 1H, H-11), 5.05 (ddd, *J* = 9.0, 5.1, 1.8 Hz, 1H, H-13), 4.10 (t, *J* = 3.6 Hz, 1H, H-5), 3.95 (q, *J* = 6.9 Hz, 1H, H-2), 3.21 – 3.13 (m, 1H, H-8), 2.90 – 2.84 (m, 1H, H-4), 2.84 – 2.76 (m, 1H, H-12), 2.08 – 2.00 (m, 1H, H-6), 1.88 – 1.80 (m, 1H, H-14a), 1.78 (d, *J* = 1.3 Hz, 3H, H-20), 1.68 – 1.61 (m, 1H, H-7a), 1.39 (d, *J* = 6.9 Hz, 3H, H-16), 1.37 – 1.29 (m, 1H, H-7b), 1.27 (d, *J* = 7.2 Hz, 3H, H-17), 1.14 (d, *J* = 7.1 Hz, 3H, H-21), 1.08 (d, *J* = 6.4 Hz, 3H, H-19), 1.01 (d, *J* = 7.1 Hz, 3H, H-18), 0.96 (t, *J* = 7.4 Hz, 3H, H-15).

^{13}C NMR (126 MHz, $CDCl_3$) δ 208.46 (C3), 206.01 (C9), 170.95 (C1), 142.04 (C11), 135.82 (C10), 78.17 (C13), 70.53 (C5), 50.81 (C2), 46.89 (C4), 39.31 (C7), 37.39 (C8), 36.00 (C12), 34.87 (C6), 24.55 (C14), 17.56 (C18), 15.07 (C19), 13.30 (C16), 12.29 (C21), 11.53 (C17), 11.46 (C20), 10.29 (C15).

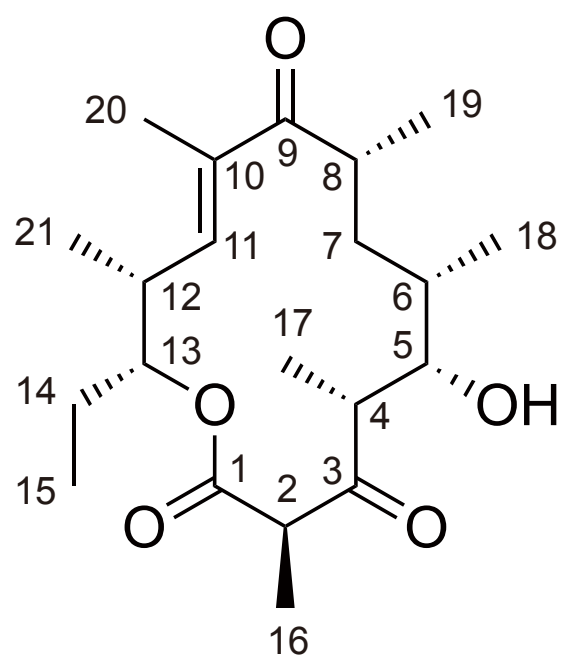

13

Isolation of 14

1.8 L of culture broth from K207-3 cells expressing **P1**-**P2**-**R5**-**P4**-**P5**-**P6**-**P7** was extracted twice with the same volume of ethyl acetate, dried with $MgSO_4$, filtered, and concentrated *in vacuo.* The extract was subjected to a flash silica gel chromatography (hexanes:ethyl acetate = 70:30 to 30:70). Fractions containing the expected product were further purified by semi-preparative HPLC [Waters 1525 HPLC system equipped with an Atlantis T3 OBST prep column (250 × 10.0 mm) with a flow rate of 4 mL min^{-1} [solvent A, water with 0.1% (v/v) formic acid; solvent B, acetonitrile with 0.1% (v/v) formic acid. 35% B isocratic elution] to obtain 3.7 mg of **14** after lyophilization.

Theoretical $[M+H]^+$ *m/z* of **14**, 367.2479; observed *m/z*, 367.2464 (-4 ppm).

^{1}H NMR (500 MHz, $CDCl_3$) δ 6.68 – 6.62 (m, 1H, H-11), 4.37 (dd, *J* = 9.8, 2.4 Hz, 1H, H-5), 3.61 (q, *J* = 6.6 Hz, 1H, H-2), 3.58 – 3.52 (m, 1H, H-13), 3.52 – 3.47 (m, 1H, H-8), 2.74 – 2.63 (m, 2H, H-4, H-12), 2.26 (ddd, *J* = 13.6, 7.7, 5.5 Hz, 1H, H-7a), 1.96 – 1.87 (m, 1H, H-6), 1.85 (s, 3H, H-20), 1.63 – 1.49 (m, 1H, H-14a), 1.49 – 1.38 (m, 1H, H-14b), 1.36 (d, *J* = 6.6 Hz, 3H, H-16), 1.29 (dt, *J* = 13.6, 6.4 Hz, 1H, H-7b), 1.17 – 1.08 (m, 9H, H-17, H-19, H-21), 1.00 (t, *J* = 7.4 Hz, 3H, H-15), 0.94 (d, *J* = 6.9 Hz, 3H, H-18).

^{13}C NMR (126 MHz, $CDCl_3$) δ 205.58 (C3), 169.62 (C1), 144.25 (C11), 135.64 (C10), 81.79 (C5), 76.35 (C8), 50.20 (C2), 43.27 (C4), 39.33 (C12), 38.50 (C7), 37.64 (C13), 31.63 (C6), 28.01 (C14), 19.04 (C19), 16.18 (C18), 14.62 (C21), 12.08 (C20), 10.30 (C15), 9.92 (C17), 8.10 (C16).
Signals for C4 and C12 are interchangeable.

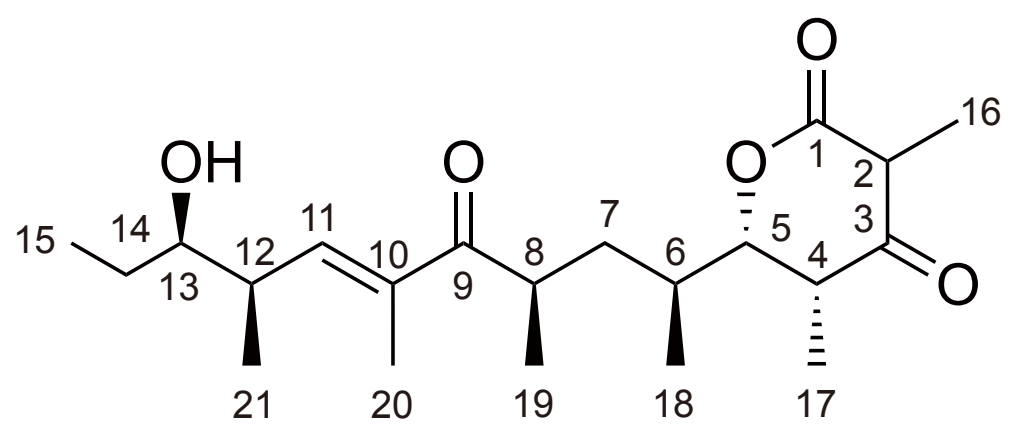

14

Isolation of 18

300 mL of culture broth from *E. coli* K207-3 cells expressing **P1**-**P2**-**P4**-**P5**-**P6**-**P7** was extracted twice with the same volume of ethyl acetate, dried with $MgSO_4$, filtered, and concentrated *in vacuo.* The extract was subjected to flash silica gel chromatography (hexanes:ethyl acetate = 60:40 to 50:50). Fractions containing **18** were further purified by HPLC [Waters 1525 HPLC system equipped with a Microsorb-MV 300-5 C_{18} column (250 × 4.6 mm) with a flow rate of 4 mL min^{-1} (solvent A, water with 0.1% formic acid; solvent B, acetonitrile with 0.1% formic acid. 30% B)] to obtain 9.3 mg of **18** after lyophilization.

Theoretical $[M+H]^+$ *m/z* of **18**, 327.2166; observed *m/z*, 327.2168 (0.61 ppm).

^{1}H NMR (500 MHz, $CDCl_3$) δ 4.35 (dd, *J* = 9.9, 2.6 Hz, 1H, H-5), 3.81 (dtd, *J* = 7.8, 5.1, 2.8 Hz, 1H, H-11), 3.63 (q, *J* = 6.7 Hz, 1H, H-2), 3.10 – 2.94 (m, 1H, H-8), 2.85 (dqd, *J* = 14.3, 7.1, 2.9 Hz, 1H, H-10), 2.75 – 2.66 (m, 1H, H4), 2.32 – 2.20 (m, 1H, H-7a), 1.96 – 1.84 (m, 1H, H-6), 1.61 – 1.49 (m, 1H,H-12a), 1.44 (dqd, *J* = 14.8, 7.4, 5.1 Hz, 1H, H12b), 1.37 (d, *J* = 6.6 Hz, 3H. H-14), 1.25 – 1.16 (m, 3H, H-17, H-7b), 1.16 – 1.11 (m, 6H, H-15, H-18), 1.04 – 0.94 (m, 6H, H-13, H-16).

^{13}C NMR (126 MHz, $CDCl_3$) δ 205.37 (C3), 169.55 (C1), 81.76 (C5), 72.79 (C11), 50.21 (C2), 47.90 (C8), 43.90 (C10), 43.18 (C4), 37.15 (C7), 31.65 (C6), 26.98 (C12), 17.51 (C17), 15.97 (C13), 10.50 (C16), 9.86 (C15), 9.78 (C18), 8.08 (C14).

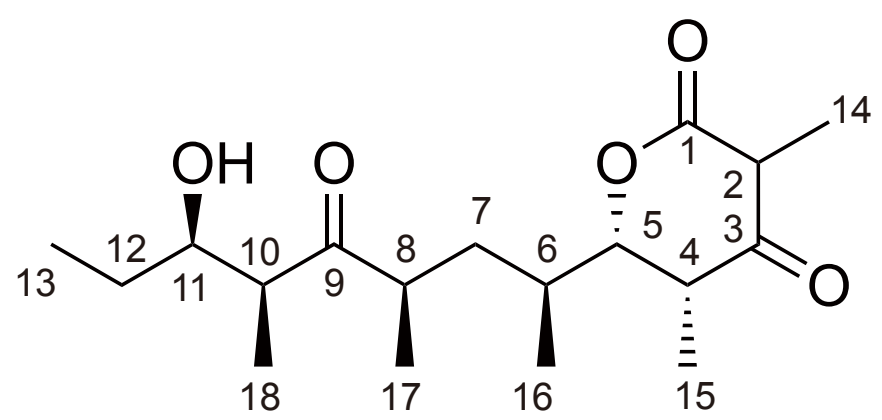

18

Isolation of narbomycin (2)

300 mL of culture broth from TM7 cells expressing **P1**-**P2**-**P3**-**P4**-**P5**-**P6**-**P7**+Des was adjusted to pH 9.5 with 1 M NaOH, extracted twice with the same volume of ethyl acetate, dried with $MgSO_4$, filtered, and concentrated *in vacuo.* The extract was subjected to flash silica gel chromatography (chloroform:methanol = 95:5). Fractions containing narbomycin were further purified by semi-preparative HPLC [Waters 1525 HPLC system equipped with an Atlantis T3 OBST prep column (250 × 10.0 mm) with a flow rate of 4 mL min^{-1} (solvent A, water with 0.1% formic acid; solvent B, acetonitrile with 0.1% formic acid. 15–90% B for 15 min)] to obtain 3.1 mg of narbomycin after lyophilization.

Theoretical $[M+H]^+$ *m/z* of **2**, 510.3425; observed *m/z*, 510.3425 (0 ppm).

^{1}H NMR (500 MHz, $CDCl_3$) δ 6.70 (dd, *J* = 16.1, 6.1 Hz, 1H, H-11), 6.11 (d, *J* = 16.2, 1H, H-10), 4.96 (m, 1H, H-13), 4.42 (d, *J* = 7.2 Hz, 1H, H-1'), 4.21 (brs, 1H, H-5), 3.87 (q, *J* = 7.0 Hz, 1H, H-2), 3.66 (dqd, *J* = 11.4, 5.9, 2.7 Hz, 1H, H-5'), 3.51 (dd, *J* = 10.1, 7.2 Hz, 1H, H-2'), 3.22 (m, 1H, H-3'), 2.94 (qd, *J* = 7.3, 4.8 Hz, 1H, H-4), 2.81 (m, 1H, H-8), 2.74 (s, 6H, H-C(NH_3)$_2$), 2.68 – 2.76 (m, overlap, 1H, H-12), 1.98 (dd, *J* = 12.2, 2.6 Hz, 1H, H-4'a), 1.78 (s, 1H, H-6), 1.67 – 1.41 (m, 4H, H-14ab, H-7a, H-4'b), 1.40 – 1.35 (m, 6H, H-19, H-18), 1.32 (d, *J* = 6.1 Hz, 3H, H-6'), 1.27 – 1.14 (m, 1H, H-7b), 1.13 (t, *J* = 6.5 Hz, 6H, H-19, H-20), 1.03 (d, *J* = 6.9 Hz, 3H, H-18), 0.92 (t, *J* = 7.3 Hz, 3H, H-15).

^{13}C NMR (126 MHz, $CDCl_3$) δ 208.86 (C3), 203.54 (C9), 169.47 (C1), 147.38 (C11), 128.07 (C10), 103.75 (C1'), 78.94 (C5), 78.72 (C13), 69.61 (C2'), 68.27 (C5'), 65.93 (C3'), 50.46 (C2), 48.79 (C4), 41.77 (C8), 39.76 (CN(CH_3)$_2$), 38.35 (C12), 36.91 (C7), 36.31 (C6), 30.87 (C4'), 22.91 (C14), 20.96 (C6'), 17.05 (C18), 15.49 (C19), 14.21 (C16), 13.41 (C17), 12.36 (C20), 10.50 (C15).

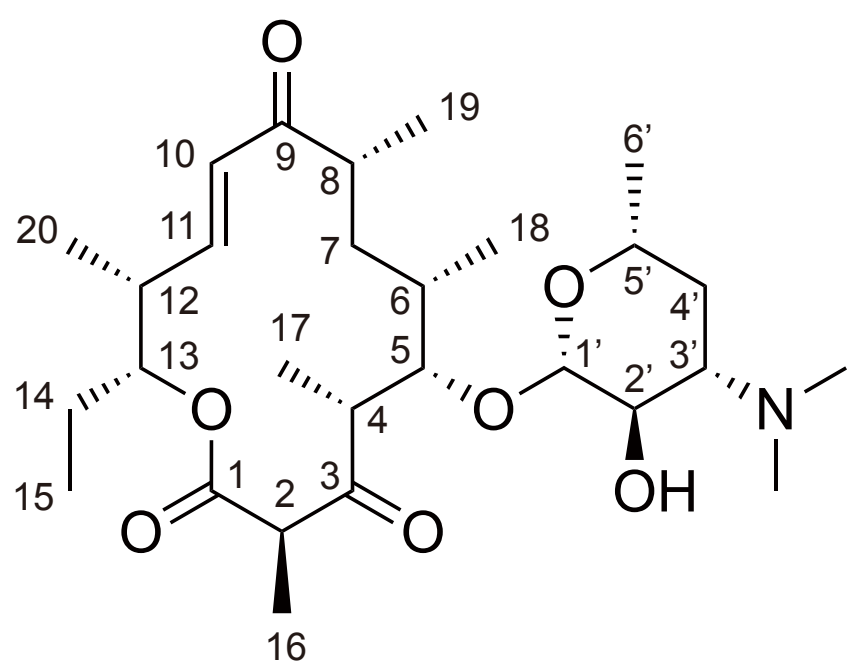

narbomycin (**2**)

Isolation of YC-17 (5)

600 mL of culture broth from TM7 cells expressing **P1**-**P2**-**P3**-**P4**-**P5**-**P6***-**P7**+Des was adjusted to pH 9.5 with 1 N NaOH, extracted twice with the same volume of ethyl acetate, dried with $MgSO_4$, filtered, and concentrated *in vacuo.* The extract was subjected to flash silica gel chromatography (chloroform:methanol = 95:5). Fractions containing YC-17 were further purified by semi-preparative HPLC [Waters 1525 HPLC system equipped with an Atlantis T3 OBST prep column (250 × 10.0 mm) with a flow rate of 4 mL min^{-1} (solvent A, water with 0.1% formic acid; solvent B, acetonitrile with 0.1% formic acid. 15–90% B for 15 min)] to obtain 3.8 mg of YC-17.

Theoretical $[M+H]^+$ *m/z* of **5**, 454.3163; observed *m/z*, 454.3165 (0.41 ppm).

^1H NMR (500 MHz, $CDCl_3$) δ 6.77 (dd, *J* = 15.7, 5.5 Hz, 1H, H-9), 6.49 – 6.40 (m, 1H, H-8), 4.97 (ddd, *J* = 8.3, 5.6, 2.3 Hz, 1H, H-11), 4.34 (d, *J* = 7.2 Hz, 1H, H-1'), 3.62 (d, *J* = 10.5 Hz, 1H, H-3), 3.60 – 3.52 (m, 1H, H-5'), 3.48 (dd, *J* = 10.2, 7.2 Hz, 1H, H-1'), 3.18 (ddd, *J* = 12.6, 10.1, 4.3 Hz, 1H, H-2'), 2.91 – 2.81 (m, 1H, H-2), 2.72 (s, 6H, H-$N(CH_3)_2$), 2.69 – 2.62 (m, 1H, C-10), 2.60 – 2.49 (m, 1H, H-6), 1.99 (m, 1H, H-4'a), 1.79 – 1.63 (m, overlap, 2H, H-12a, H-5a), 1.57 (ddd, *J* = 13.8, 7.6, 6.0 Hz, 1H, H-12b), 1.50 – 1.42 (m, overlap, 2H, H-4'b, H5b), 1.41 (d, *J* = 7.0 Hz, 3H, H-14), 1.30 (d, *J* = 6.1 Hz, 3H, H-6'), 1.28 – 1.23 (m, overlap, 1H, H-4), 1.22 (d, *J* = 6.9 Hz, 3H, H-16), 1.13 (d, *J* = 6.8 Hz, 3H, H-17), 1.01 (d, *J* = 6.7 Hz, 3H, H-15), 0.93 (t, *J* = 7.4 Hz, 3H, H-13).

^{13}C NMR (126 MHz, $CDCl_3$) δ 205.24 (C7), 175.03 (C1), 147.04 (C9), 125.91 (C8), 104.03 (H-1'), 85.97 (C3), 73.79 (C11), 69.79 (C1'), 68.16 (C5'), 66.25 (2'), 45.11 (C6), 43.87 (C2), 39.91($C(NCH_3)_2$), 37.89 (C10), 34.09 (C5), 33.52(C4), 30.70 (C4'), 25.15 (C12), 20.89 (C6'), 17.73 (C16), 17.39 (C15), 16.18 (C14), 10.31 (C13), 9.60 (C17).

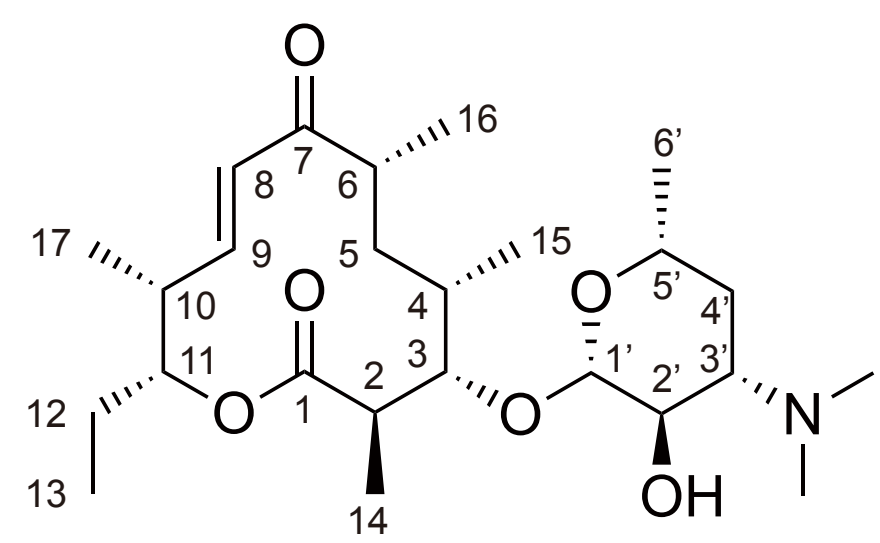

5

Isolation of 21

2.4 L of culture broth from TM7 cells expressing **P1**-**P2**-**P5**-**P4**-**P5**-**P6**-**P7**+Des+PikAV was adjusted to pH 9.5 with1 N NaOH, extracted twice with the same volume of ethyl acetate, dried with $MgSO_4$, filtered, and concentrated *in vacuo.* The extract was subjected to flash silica gel chromatography (chloroform:methanol = 95:5). Fractions containing **21** were further purified by semi-preparative HPLC [Waters 1525 HPLC system equipped with an Atlantis T3 OBST prep column (250 × 10.0 mm) with a flow rate of 4 mL min^{-1} (solvent A, water with 0.1% formic acid; solvent B, acetonitrile with 0.1% formic acid. 15–70% B for 20 min)] to obtain 1.9 mg of **21** after lyophilization.

Theoretical $[M+H]^+$ *m/z* of **21**, 526.3738; observed *m/z*, 526.3744 (1.1 ppm).

^1H NMR (500 MHz, $CDCl_3$) δ 4.99 (ddd, *J* = 9.2, 4.6, 2.2 Hz, 1H, H-13), 4.39 (d, *J* = 7.3 Hz, 1H, H-1'), 4.00 (dd, *J* = 4.8, 3.1 Hz, 1H, H-5), 3.83 (q, *J* = 7.1 Hz, 1H, H-2), 3.59 (dqd, *J* = 12.3, 6.0, 1.8 Hz, 1H, H-2), 3.41 (dd, *J* = 10.2, 7.3 Hz, 1H, H-5'), 2.93 (qd, *J* = 7.1, 4.8 Hz, 1H, H-4), 2.77 (m, 2H, H-3', H-8), 2.66 (m, 1H, H-10), 2.48 (s, 6H, H-N$(CH_3)_2$), 2.04 (s, 1H, H-6), 1.89 – 1.81 (m, 2H, H-12, H-4'a), 1.73 – 1.61 (m, 2H, H-11a, H-14a), 1.58 – 1.49 (m, 1H, H-14b), 1.44 – 1.39 (m, 4H, H-11b, H-16), 1.39 – 1.34 (m, 2H, H-4'b, H-7a), 1.32 (d, *J* = 7.1, 3H, H-17), 1.29 – 1.24 (d, *J* = 6.3 Hz, 3H, H-6'), 1.09 – 1.01 (m, 10H, H-7b, H-18, H-19, H-20), 0.98 (d, *J* = 7.0 Hz, 3H, H-21), 0.90 (t, *J* = 7.4 Hz, 3H, H-15).

^{13}C NMR (126 MHz, $CDCl_3$) δ 217.84 (C9), 209.09 (C3), 171.05 (C1), 105.05 (C1'), 79.60 (C5), 77.87 (C13), 70.28 (C2'), 69.27 (C5'), 65.35 (C3'), 52.09 (C2), 46.85 (C4), 43.38 (C8), 42.34 (C10), 40.35 (CN$(CH_3)_2$), 36.11 (C11), 35.92 (C7), 35.82 (C6), 34.08 (C12), 30.16 (C4'), 23.99 (C14), 21.12 (C6'), 17.50 (C18), 15.77 (C19), 15.60 (C21), 15.46 (C20), 14.05 (C17), 13.89 (C16), 10.49 (C15).
Signals for C16 and C17, C18, C19, C20, and C21, C6, C7, C11, and C12 are interchangeable.

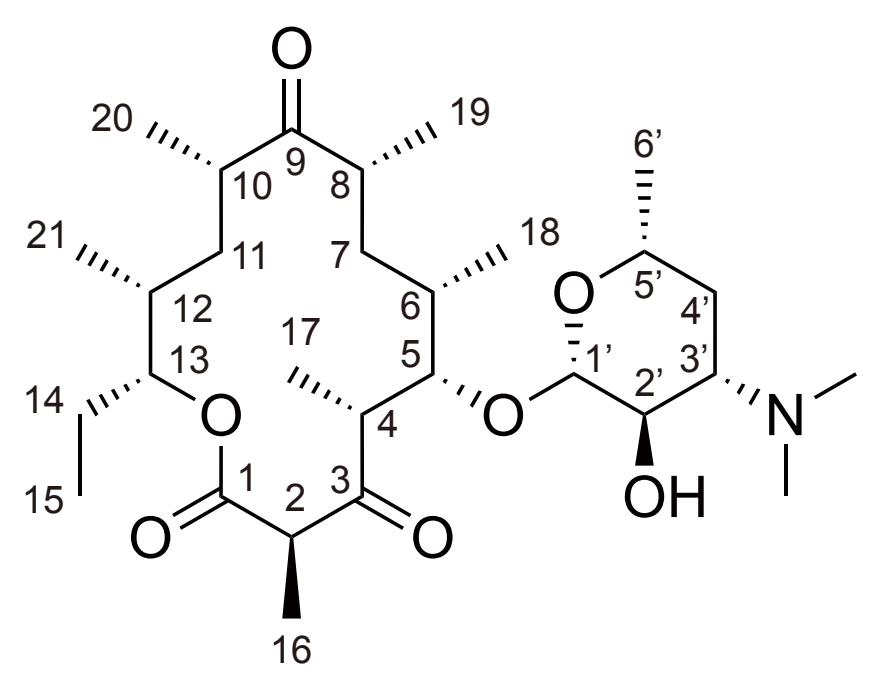

21

Isolation of 22

3.9 L of culture broth from TM7 cells expressing **P1**-**R6**-**P3**-**P4**-**P5**-**P6**-**P7** was adjusted to pH 9.5 with1 N NaOH, extracted twice with the same volume of ethyl acetate, dried with $MgSO_4$, filtered, and concentrated *in vacuo*. The extract was subjected to flash silica gel chromatography (chloroform:methanol = 95:5). Fractions containing **22** were further purified by semi-preparative HPLC [Waters 1525 HPLC system equipped with an Atlantis T3 OBST prep column (250 × 10.0 mm) with a flow rate of 4 mL min^{-1} (solvent A, water with 0.1% formic acid; solvent B, acetonitrile with 0.1% formic acid. 20-38% B for 30 min)] to obtain 0.9 mg of **22** after lyophilization.

Theoretical $[M+H]^+$ *m/z* of **22**, 496.3268; observed *m/z*, 496.3271 (0.6 ppm).

^{1}H NMR (500 MHz, $CDCl_3$) δ 6.61 (ddd, *J* = 15.3, 9.4, 5.3 Hz, 1H, H-11), 6.14 (d, *J* = 15.9 Hz, 1H, H-10), 5.09 – 4.98 (m, 1H, H-13), 4.40 (d, *J* = 7.2 Hz, 1H, H-1’), 4.17 – 4.11 (m, 1H, H-5), 3.86 (q, *J* = 6.9 Hz, 1H, H-2), 3.66 (dd, *J* = 11.2, 6.0 Hz, 1H, H-10), 3.54 (dd, *J* = 10.1, 7.2 Hz, 1H, H-2’), 3.27 – 3.18 (m, 1H, H-3’), 2.96 (dd, *J* = 7.5, 4.9 Hz, 1H, H-3), 2.88 – 2.82 (m, 1H, H-8), 2.80 (s, 6H, H-N(CH_3)$_2$), 2.64 (d, *J* = 14.9 Hz, 1H, H-12a), 2.39 (dt, *J* = 15.7, 8.5 Hz, 1H, H=12b), 2.19 (d, *J* = 12.6 Hz, 1H, H-4’a), 1.81 – 1.72 (m, 1H, H-14), 1.71 – 1.42 (m, 4H, H-14ab, H-7a, H-4’b), 1.38 (d, *J* = 6.9 Hz, 3H, H-16), 1.36 (d, *J* = 7.4 Hz, 3H, H-17), 1.32 (d, *J* = 6.1 Hz, 3H, H-6’), 1.31 – 1.25 (m, 1H, H-7b), 1.13 (d, *J* = 6.6 Hz, 3H, H-19), 1.02 (d, *J* = 6.9 Hz, 3H, H-18), 0.95 (t, *J* = 7.4 Hz, 3H, H-15).

^{13}C NMR (126 MHz, $CDCl_3$) δ 208.80 (C3), 203.40 (C9), 169.38 (C1), 141.98 (C11), 130.34 (C10), 103.64 (C1’), 79.49 (C5), 75.05 (C13), 69.75 (C2’), 68.44 (C5’), 66.10 (C3’), 50.53 (C2), 48.92 (C3), 41.11 (C8), 40.25 (C(NH_3)$_2$), 36.69 (C7), 36.21 (C12), 36.01 (C6), 31.55 (C4’), 27.06 (C14), 20.90 (C6’), 17.15 (C18), 15.83 (C19), 14.46 (C16), 13.03 (C17), 9.79 (C15).

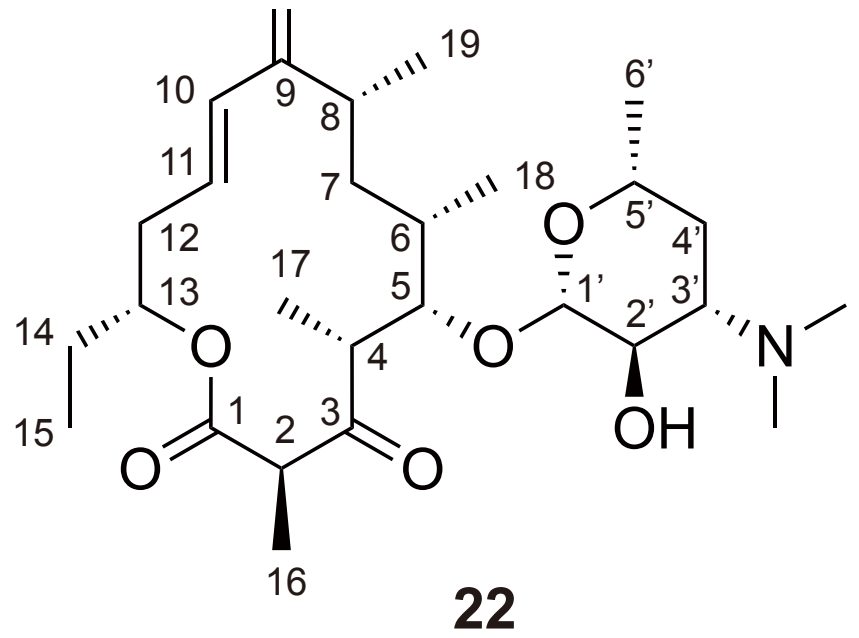

22

Isolation of 23

3.9 L of culture broth from TM7 cells expressing **P1**-**P2**-**R5**-**P4**-**P5**-**P6**-**P7** was adjusted to pH 9.5 with1 N NaOH, extracted twice with the same volume of ethyl acetate, dried with $MgSO_4$, filtered, and concentrated *in vacuo*. The extract was subjected to flash silica gel chromatography (chloroform:methanol = 92.5 : 7.5). Fractions containing **23** were further purified by semi-preparative HPLC [Waters 1525 HPLC system equipped with an Atlantis T3 OBST prep column (250 × 10.0 mm) with a flow rate of 4 mL min^{-1} (solvent A, water with 0.1% formic acid; solvent B, acetonitrile with 0.1% formic acid. 22-40% B for 30 min)] to obtain 1.9 mg of **23** after lyophilization.

Theoretical $[M+H]^+$ *m/z* of **23**, 524.3582; observed *m/z*, 524.3584 (0.6 ppm).

^{1}H NMR (500 MHz, $CDCl_3$) δ 6.36 (d, *J* = 8.9 Hz, 1H, H-17), 4.92 (ddd, *J* = 8.2, 6.0, 2.0 Hz, 1H, H-13), 4.48 (d, *J* = 7.2 Hz, 1H, H-1'), 4.21 – 4.16 (m, 1H, H-5), 3.99 (q, *J* = 6.8 Hz, 1H, H-2), 3.70 – 3.63 (m, 1H, H-5'), 3.57 – 3.52 (m, 1H, H-2'), 3.21 – 3.12 (m, 1H, H-3'), 3.03 (d, *J* = 10.4 Hz, 1H, H-8), 2.94 – 2.85 (m, 1H, H-4), 2.85 – 2.78 (m, 1H, H-12), 2.76 (s, 6H, H-N(CH_3)$_2$), 2.19 (brs, 1H, H-6), 2.15 – 2.08 (m, 1H, H-4'a), 1.91 – 1.79 (m, 1H, H-14a), 1.77 (s, 3H, H-20), 1.61 (dp, *J* = 14.1, 7.3 Hz, 1H, H-14b), 1.55 – 1.42 (m, 2H, H-7a, H-4'b), 1.40 (d, *J* = 6.8 Hz, 3H, H-16), 1.38 (d, *J* = 7.3 Hz, 3H, H-17), 1.30 (d, *J* = 6.1 Hz, 3H, H-6'), 1.28 – 1.22 (m, 1H, H-7b), 1.12 (d, *J* = 7.0 Hz, 3H, H-21), 1.08 (d, *J* = 6.3 Hz, 3H, H-19), 1.03 (d, *J* = 6.9 Hz, 3H, H-18), 0.96 (t, *J* = 7.4 Hz, 3H, H-15).

^{13}C NMR (126 MHz, $CDCl_3$) δ 208.34 (C3), 205.58 (C9), 170.43 (C1), 142.33 (C17), 104.41 (C1'), 78.27 (C5/13), 69.93 (C2'), 68.86 (C5'), 65.68 (C3'), 51.04 (C2'), 50.92 (C20), 46.50 (C4), 40.38 (C-N(CH_3)$_2$), 39.30 (C7), 37.37 (C8), 35.60 (C12), 35.56 (C6), 31.97 (C4'), 24.49 (C14), 21.06 (C6'), 17.31 (C18), 14.60 (C19), 14.15 (C16), 12.75 (C17), 12.37 (C21), 11.59 (C20), 10.26 (C15).

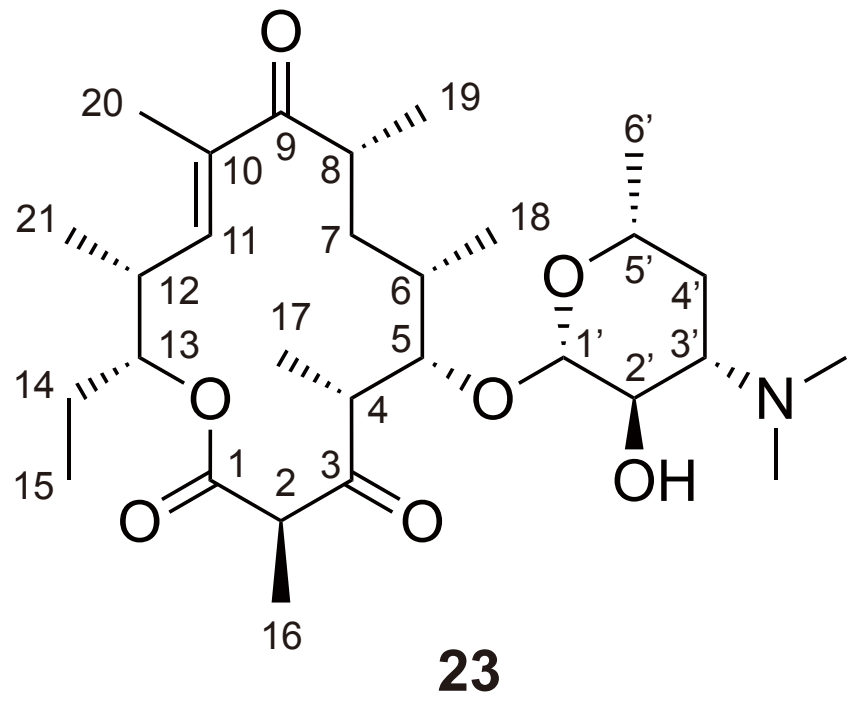

23

Crystallization of 12

Purified **12** was dissolved in a hexanes : ethyl acetate ratio of 70 : 30 at 50 °C, filtered using a cotton plug, and evaporated at room temperature overnight (Table S3 and Supplementary Data 2–3).

Minimum inhibitory concentration (MIC) assay

The antibacterial activities of the purified compounds were evaluated against *Bacillus subtilis*, *Staphylococcus epidermidis*, *Micrococcus luteus*, *Escherichia coli*, and *Pseudomonas putida* according to the Clinical and Laboratory Standards Institute (CLSI) broth microdilution method (Figure S64). The test strains were grown in Mueller-Hinton (M-H) medium overnight (*B. subtilis, S. epidermidis*, and *E.coli* were grown at 37 °C, while *M. luteus* and *P. putida* were grown at 30 °C). The broths were diluted by the M-H medium (1:500) and used as an inoculum. 50 μL of the inoculum was mixed with 50 μL of M-H medium containing 2-fold serial dilutions of the compounds in 96-well plates. After incubating the cells for 24 h at 30 or 37 °C, each MIC was determined as the lowest concentration that prevented visible bacterial growth.

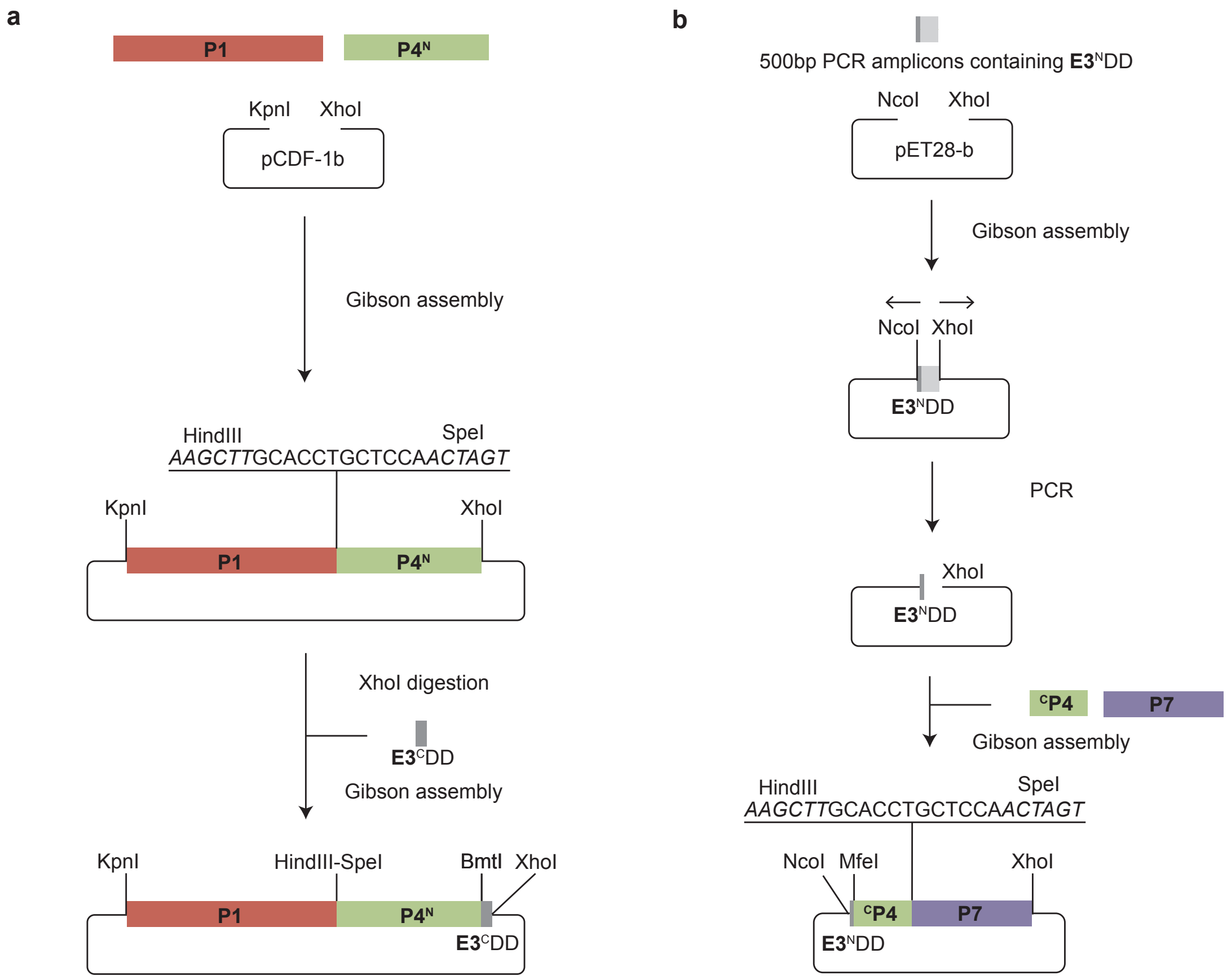

Figure S1. Construction of 2-plasmid system. a) Construction of p**P1-P4**^{N}. Amplicons of **P1** and **P4**^{N} were joined with KpnI/XhoI-digested pCDF-1b by Gibson assembly. The HindIII-N_{12}-SpeI sequence between **P1** and **P4**^{N} comes from the primers. An amplicon containing a BmtI site and **E3** ^{C}DD was inserted into the XhoI site through SLiCE assembly. b) Construction of p^{C}**P4-P7**. 500 bp PCR amplicons containing **E3** ^{N}DD was cloned into NcoI/XhoI-digested pET-28b. The resulting plasmid was used as a PCR template to generate an amplicon containing **E3** ^{N}DD. This amplicon and amplicons containing ^{C}**P4** and **P7** were joined by Gibson assembly. The HindIII-N_{12}-SpeI sequence between **P1** and **P4**^{N} comes from the primers.

P1-P4^{N}-E3^{C}DD

MAHHHHHHVGTSSAGITRTGARTPVTGRGAAAWDTGEVRVRRGLPPAGPDHAEHSFSRAPTG
DVRAELIRGEMSTVSKSESEEFVSVSNDAGSAHGTAEPVAVVGISCRVPGARDPREFWELLA
AGGQAVTDVPADRWNAGDFYDPDRSAPGRSNSRWGGFIEDVDRFDAAFFGISPREAAEMDPQ
QRLALELGWEALERAGIDPSSLTGTRTGVFAGAIWDDYATLKHRQGGAAITPHTVTGLHRGI
IANRLSYTLGLRGPSMVVDSGQSSSLVAVHLACESLRRGESELALAGGVSLNLVPDSIIGAS
KFGGLSPDGRAYTFDARANGYVRGEGGGFVVLKRLSRAVADGDPVLAVIRGSAVNNGGAAQG
MTTPDAQAQEAVLREAHERAGTAPADVRYVELHGTGTPVGDPIEAAALGAALGTGRPAGQPL
LVGSVKTNIGHLEGAAGIAGLIKAVLAVRGRALPASLNYETPNPAIPFEELNLRVNTEYLPW
EPEHDGQRMVVGVSSFGMGGTNAHVVLEEAPGGCRGASVVESTVGGSAVGGGVVPWVVSAKS
AAALDAQIERLAAFASRDRTDGVDAGAVDAGAVDAGAVARVLAGGRAQFEHRAVVVGSGPDD
LAAALAAPEGLVRGVASGVGRVAFVFPGQGTQWAGMGAELLDSSAVFAAAMAECEAALSPYV
DWSLEAVVRQAPGAPTLERVDVVQPVTFAVMVSLARVWQHHGVTPQAVVGHSQGEIAAAYVA
GALSLDDAARVVTLRSKSIAAHLAGKGGMLSLALSEDAVLERLAGFDGLSVAAVNGPTATVV
SGDPVQIEELARACEADGVRARVIPVDYASHSRQVEIIESELAEVLAGLSPQAPRVPFFSTL
EGAWITEPVLDGGYWYRNLRHRVGFAPAVETLATDEGFTHFVEVSAHPVLTMALPGTVTGLA
TLRRDNGGQDRLVASLAEAWANGLAVDWSPLLPSATGHHSDLPTYAFQTERHWLGEIEALAP
AGEPAVQPAVLRTEAAEPAELDRDEQLRVILDKVRAQTAQVLGYATGGQIEVDRTFREAGCT
SLTGVDLRNRINAAFGVRMAPSMIFDFPTPEALAEQLLLVVHGEAAANPAGAEPAPVAAAGA
VDEPVAIVGMACRLPGGVASPEDLWRLVAGGGDAISEFPQDRGWDVEGLYHPDPEHPGTSYV
RQGGFIENVAGFDAAFFGISPREALAMDPQQRLLLETSWEAVEDAGIDPTSLRGRQVGVFTG
AMTHEYGPSLRDGGEGLDGYLLTGNTASVMSGRVSYTLGLEGPALTVDTACSSSLVALHLAV
QALRKGEVDMALAGGVAVMPTPGMFVEFSRQRGLAGDGRSKAFAASADGTSWSEGVGVLLVE
RLSDARRNGHQVLAVVRGSAVNQDGASNGLTAPNGPSQQRVIRRALADARLTTSDVDVVEAH
GTGTRLGDPIEAQALIATYGQGRDDEQPLRLGSLKSNIGHTQAAAGVSGVIKMVQAMRHGLL
PKTLHVDEPSDQIDWSAGAVELLTEAVDWPEKQDGGLRRAAVSSFGISGTNAHVVLEEAPVV
VKLAPAPTSETPASEATPAVEPSVGAGLVPWLVSAKTPAALDAQIGRLAAFASQGRTDAADP
GAVARVLAGGRAEFEHRAVVLGTGQDDFAQALTAPEGLIRGTPSDVGRVAFVFPGQGTQWAG
MGAELLDVSKEFAAAMAECESALSRYVDWSLEAVVRQAPGAPTLERVDVVQPVTFAVMVSLA
KVWQHHGVTPQAVVGHSQGEIAAAYVAGALTLDDAARVVTLRSKSIAAHLAGKGGMISLALS
EEATRQRIENLHGLSIAAVNGPTATVVSGDPTQIQELAQACEADGVRARIIPVDYASHSAHV
ETIESELAEVLAGLSPRTPEVPFFSTLEGAWITEPVLDGTYWYRNLRHRVGFAPAVETLATD
EGFTHFIEVSAHPVLTMTLPETVTGLGTLRREQGGQERLVTSLAEAWTNGLTIDWAPVLPTA
TGHHPELPTYAFQRRHYWLHDSPAVQGSVQDSWRYRIDWKRLAVADASERAGLSGRWLVVVP
EDRSAEAAPVLAALSGAGADPVQLDVSPLGDRQRLAATLGEALAAAGGAVDGVLSLLAWDES
AHPGHPAPFTRGTGATLTLVQALEDAGVAAPLWCVTHGAVSVGRADHVTSPAQAMVWGMGRV
AALEHPERWGGLIDLPSDADRAALDRMTTVLAGGTGEDQVAVRASGLLARRLVRASLPAHGT
ASPWWQADGTVLVTGAEEPAAAEAARRLARDGAGHLLLHTTPSGSEGAEGTSGAAEDSGLAG

LVAELADLGATATVVTCDLTDAEAAARLLAGVSDAHPLSAVLHLPPTVDSEPLAATDADALA
RVVTAKATAALHLDRLLREAAAAGGRPPVLVLFSSVAAIWGGAGQGAYAAGTAFLDALAGQH
RADGPTVTSVAWSPWEGSRVTEGATGERLRRLGLRPLAPATALTALDTALGHGDTAVTIADV
DWSSFAPGFTTARPGTLLADLPEARRALDEQQSTTAADDTVLSRELGALTGAEQQRRMQELV
REHLAVVLNHPSPEAVDTGRAFRDLGFDSLTAVELRNRLKNATGLALPATLVFDYPTPRTLA
EFLLAEILGEQAGASGEAPSALAGLDALEAALPEVPATEREELVQRLERMLAALRPVAQAAD
ASGTGANPSGDDLGEAGVDELLEALGRELDGD

$E3^{N}DD-^{C}P4-P7$

MTDSEKVAEYLRRATLDLRAARQRIRELESDPIAIVGMACRLPGGVASPEDLWRLVAGGEDA
ISGFPQDRGWDVEGLYDPDPDASGRTYCRAGGFLDEAGEFDADFFGISPREALAMDPQQRLL
LETSWEAVEDAGIDPTSLQGQQVGVFAGTNGPHYEPLLRNTAEDLEGYVGTGNAASIMSGRV
SYTLGLEGPAVTVDTACSSSLVALHLAVQALRKGECGLALAGGVTVMSTPTTFVEFSRQRGL
AEDGRSKAFAASADGFGPAEGVGMLLVERLSDARRNGHRVLAVVRGSAVNQDGASNGLTAPN
GPSQQRVIRRALADARLTTADVDVVEAHGTGTRLGDPIEAQALIATYGQGRDTEQPLRLGSL
KSNIGHTQAAAGVSGIIKMVQAMRHGVLPKTLHVDRPSDQIDWSAGTVELLTEAMDWPRKQE
GGLRRAAVSSFGISGTNAHIVLEEAPVDEKLAPAPTSSPAVEPPAGGGVVPWPVSAKTSAAL
DAQIGQLAAYAEDRTDVDPAVAARALVDSRTAMEHRAVAVGDSREALRDALRMPEGLVRGTV
TDPGRVAFVFPGQGTQWAGMGAELLDSSPEFAAAMAECETALSPYVDWSLEAVVRQAPSAPT
LDRVDVVQPVTFAVMVSLAKVWQHHGITPEAVIGHSQGEIAAAYVAGALTLDDAARVVTLRS
KSIAAHLAGKGGMISLALSEEATRQRIENLHGLSIAAVNGPTATVVSGDPTQIQELAQACEA
DGIRARIIPVDYASHSAHVETIENELADVLAGLSPQTPQVPFFSTLEGTWITEPALDGGYWY
RNLRHRVGFAPAVETLATDEGFTHFIEVSAHPVLTMTLPDKVTGLATLRREDGGQHRLTTSL
AEAWANGLALDWASLLPATGALSPAVPDLPTYAFQHRSYWISPAGPGEAPAHTASGREAVAE
TGLAWGPGAEDLDEEGRRSAVLAMVMRQAASVLRCDSPEEVPVDRPLREIGFDSLTAVDFRN
RVNRLTGLQLPPTVVFEHPTPVALAERISDELAERNWAVAEPSDHEQAEEEKAAAPAGARSG
ADTGAGAGMFRALFRQAVEDDRYGEFLDVLAEASAFRPQFASPEACSERLDPVLLAGGPTDR
AEGRAVLVGCTGTAANGGPHEFLRLSTSFQEERDFLAVPLPGYGTGTGTGTALLPADLDTAL
DAQARAILRAAGDAPVVLLGHSGGALLAHELAFRLERAHGAPPAGIVLVDPYPPGHQEPIEV
WSRQLGEGLFAGELEPMSDARLLAMGRYARFLAGPRPGRSSAPVLLVRASEPLGDWQEERGD
WRAHWDLPHTVADVPGDHFTMMRDHAPAVAEAVLSWLDAIEGIEGAGK

Figure S2. Sequences of **$P1-P4^{N}-E3^{C}$DD** and **$E3^{N}$DD-^{C}P4-P7**. Red, green, purple, and gray letters indicate **P1**, **P4**, **P7**, and **E3** DD, respectively (colored as in Fig. 1). Yellow letters indicate sequences encoded by HindIII-N_{12}-SpeI insertion sites.

S3 Regulator

ATAGACTACAAGCTTGCACCTGCTCCAACTAGTCATGTAGCGATTACACTTTGGCTAGCGCC
GGGACATTCGAGGAACTCGACAGGTGGGCGGCGAACCTGCCCACGCTGGCCAGGGATGAGGC
CACGCGGGCGCAGATCACCACCCGGCTACAGGCGATCTTGCAGAGCCTGGCGGACGTGTCCG
GCGGAACCGGCGGCGGCTCCGTGCCGGACCGGCTCAGATCGGCCACGGACGACGAGCTTTTC
CAACTCCTCGACAACGATCTCGAACTTCCCTGACAAAGCCCGAAAGGAAGCTGAGTTGGCTG
CTGCCACCGCTGAGCAATAACTAGCATAACCCCTTGGGGCCTCTAAACGGGTCTTGAGGGGT
TTTTTACTGCTACTTAATACGACTCACTATAGGGGAATTGTGAGCGGATAACAATTCCCCTG
TAGAAATAATTTTGTTTAACTTTAATAAGGAGATATACCATGTCGAATGAAGAGAAGCTCCG
GGAGTACTTGCGGCGTGCGCTCGTGGATCTGCACCAGGCGCGCGAGCGGCTGCACGAGGCGG
AGTCGGGAGAGCGGGAACCAATTGCGTAGGTACCAGACTCTAGCTCTAGAGACATAAAT

S5 Regulator

ATAGACTACAAGCTTGCACCTGCTCCAACTAGTCATGTAGCGATTACACTTTGGCTAGCACC
GTGGACTCGGCCCTTGCCGAGCTCGATCGAATCGAGCAGCAGCTCTCGATGCTCACCGGCGA
AGCGCGGGCACGGGACCGAATCGCGACACGACTGCGAGCCCTCCACGAGAAGTGGAACAGCG
CAGCTGAAGTACCGACCGGAGCCGATGTCCTGAGCACGCTCGATTCGGCGACGCACGACGAG
ATATTCGAGTTCATCGACAACGAGCTCGACCTGTCCTGACAAAGCCCGAAAGGAAGCTGAGT
TGGCTGCTGCCACCGCTGAGCAATAACTAGCATAACCCCTTGGGGCCTCTAAACGGGTCTTG
AGGGGTTTTTTACTGCTACTTAATACGACTCACTATAGGGGAATTGTGAGCGGATAACAATT
CCCCTGTAGAAATAATTTTGTTTAACTTTAATAAGGAGATATACCATGGCCAATGAAGAAAA
GCTCTTCGGCTATCTGAAGAAGGTAACTGCGGACCTGCATCAGACCCGGCAGCGCCTGCTCG
CGGCCGAGAGCCGGAGTCAGGAGCCAATTGCGTAGGTACCAGACTCTAGCTCTAGAGACATA
AAT

S8 Regulator

ATAGACTACAAGCTTGCACCTGCTCCAACTAGTCATGTAGCGATTACACTTTGGCTAGC
AACGGCATCGAATCGCTGCTCGCCGAGCTCGACAGGCTGGATACCACGTTGGCGCAAGGGCC
TTCGATCCCACTGGAAGACCAGGCCAAGGTGGCGGAGCGCTTGCACGCACTCCTCGCCAAGT
GGGACGGGGCGCGTGACGGCACGGCCAGAGCGACGTCACCCCAATCGCTGACGGCGGCCACG
GACGACGAAATCTTCGACCTCATCGACCGGAAGTTCCGGCGCTGA
CAAAGCCCGAAAGGAAGCTGAGTTGGCTGCTGCCACCGCTGAGCAATAACTAGCATAACCCC
TTGGGGCCTCTAAACGGGTCTTGAGGGGTTTTTTACTGCTACTTAATACGACTCACTATAGG
GGAATTGTGAGCGGATAACAATTCCCCTGTAGAAATAATTTTGTTTAACTTTAATAAGGAGA
TATACCATGGCCAATGAAGAAAAGCTCCGCGAGTACCTCAAGCGTGTCGTCGTCGAACTGGA
AGAGGCGCACGAACGCCTGCACGAGTTGGAGCGCCAGGAGCACGACCCAATTGCGTAGGTAC
CAGACTCTAGCTCTAGAGACATAAAT

E5 Regulator

ATAGACTACAAGCTTGCACCTGCTCCAACTAGTCATGTAGCGATTACACTTTGGCTAGCGCG
GTTGACATCGGGGACCGCCTGGATGAGTTGGAGAAAGCCCTGGAAGCACTGTCTGCCGAAGA
TGGGCACGACGATGTAGGGCAGCGTCTGGAGTCCCTTCTTCGCCGTTGGAATAGCCGCCGTG
CCGACGCTCCCTCCACCAGCGCAATCAGCGAAGATGCATCAGACGACGAGTTGTTCAGTATG
TTGGATCAACGTTTTGGTGGGGGAGAGGATTTGTAACAAAGCCCGAAAGGAAGCTGAGTTGG
CTGCTGCCACCGCTGAGCAATAACTAGCATAACCCCTTGGGGCCTCTAAACGGGTCTTGAGG
GGTTTTTTACTGCTACTTAATACGACTCACTATAGGGGAATTGTGAGCGGATAACAATTCCC
CTGTAGAAATAATTTTGTTTAACTTTAATAAGGAGATATACCATGAGCGGTGACAACGGCAT
GACCGAGGAAAAGCTCCGGCGCTACCTCAAGCGCACCGTCACCGAGCTCGACTCGGTGACCG
CGCGCCTGCGTGAAGTCGAGCACCGGGCCGGTGAGCCAATTGCGTAGGTACCAGACTCTAGC
TCTAGAGACATAAAT

P3 Regulator

ATAGACTACAAGCTTGCACCTGCTCCAACTAGTCATGTAGCGATTACACTTTGGCTAGCGAC
CAGGACGGAGCCGGGAACCGGAACGGGAACGAGAACGGGACGACGGCGTCCCGGAGCACCGC
CGAGACGGACGCGCTGCTGGCACAACTGACCCGCCTGGAAGGCGCCTTGGTGCTGACGGGCC
TCTCGGACGCCCCCGGGAGCGAAGAAGTCCTGGAGCACCTGCGGTCCCTGCGCTCGATGGTC
ACGGGCGAGACCGGGACCGGGACCGCGTCCGGAGCCCCGGACGGCGCCGGGTCCGGCGCCGA
GGACCGGCCCTGGGCGGCCGGGGACGGAGCCGGGGGCGGGAGTGAGGACGGCGCGGGAGTGC
CGGACTTCATGAACGCCTCGGCCGAGGAACTCTTCGGCCTCCTCGACCAGGACCCCAGCACG
GACTGACAAAGCCCGAAAGGAAGCTGAGTTGGCTGCTGCCACCGCTGAGCAATAACTAGCAT
AACCCCTTGGGGCCTCTAAACGGGTCTTGAGGGGTTTTTTACTGCTACTTAATACGACTCAC
TATAGGGGAATTGTGAGCGGATAACAATTCCCCTGTAGAAATAATTTTGTTTAACTTTAATA
AGGAGATATACCATGTCCACGGTGAACGAAGAGAAGTACCTCGACTACCTGCGTCGTGCCAC
GGCGGACCTCCACGAGGCCCGTGGCCGCCTCCGCGAGCTGGAGGCGAAGGCGGGCGAGCCAA
TTGCGTAGGTACCAGACTCTAGCTCTAGAGACATAAAT

P5 Regulator

ATAGACTACAAGCTTGCACCTGCTCCAACTAGTCATGTAGCGATTACACTTTGGCTAGCGGG
TCCTGGGCGGAAGGCACCGGGTCCGGAGACACGGCCTCGGCGACCGATCGGCAGACCACGGC
GGCCCTCGCCGAACTCGACCGGCTGGAAGGCGTGCTCGCCTCCCTCGCGCCCGCCGCCGGCG
GCCGTCCGGAGCTCGCCGCCCGGCTCAGGGCGCTGGCCGCGGCCCTGGGGGACGACGGCGAC
GACGCCACCGACCTGGACGAGGCGTCCGACGACGACCTCTTCTCCTTCATCGACAAGGAGCT
GGGCGACTCCGACTTCTGACAAAGCCCGAAAGGAAGCTGAGTTGGCTGCTGCCACCGCTGAG
CAATAACTAGCATAACCCCTTGGGGCCTCTAAACGGGTCTTGAGGGGTTTTTTACTGCTACT
TAATACGACTCACTATAGGGGAATTGTGAGCGGATAACAATTCCCCTGTAGAAATAATTTTG
TTTAACTTTAATAAGGAGATATACCATGGCGAACAACGAAGACAAGCTCCGCGACTACCTCA

AGCGCGTCACCGCCGAGCTGCAGCAGAACACCAGGCGTCTGCGCGAGATCGAGGGACGCACG
CACGAGCCAATTGCGTAGGTACCAGACTCTAGCTCTAGAGACATAAAT

P6 Regulator

ATAGACTACAAGCTTGCACCTGCTCCAACTAGTCATGTAGCGATTACACTTTGGCTAGCCCG
GCACCAACCGATTGGGAGGGGCGCGTTCGTCGTGCTCTTGCTGAATTGCCGTTAGATCGTCT
GCGTGATGCTGGAGTACTTGACACTGTTCTTCGTCTTACTGGAATTGAACCGGAACCTGGCT
CCGGAGGGTCCGATGGTGGAGCGGCAGATCCGGGAGCGGAACCCGAAGCGTCAATTGACGAT
CTTGACGCTGAAGCGTTAATTCGTATGGCTCTGGGGCCCCGCAACACCTGACAAAGCCCGAA
AGGAAGCTGAGTTGGCTGCTGCCACCGCTGAGCAATAACTAGCATAACCCCTTGGGGCCTCT
AAACGGGTCTTGAGGGGTTTTTTACTGCTACTTAATACGACTCACTATAGGGGAATTGTGAG
CGGATAACAATTCCCCTGTAGAAATAATTTTGTTTAACTTTAATAAGGAGATATACCATGAC
GAGTTCCAACGAACAGTTGGTGGACGCTCTGCGCGCCTCTCTCAAGGAGAACGAAGAACTCC
GGAAAGAGAGCCGTCGCCGGGCCGACCGTCGGCAGGAGCCAATTGCGTAGGTACCAGACTCT
AGCTCTAGAGACATAAAT

A1 Regulator

ATAGACTACAAGCTTGCACCTGCTCCAACTAGTCATGTAGCGATTACACTTTGGCTAGCCTC
ACCACAGGGGTCTTGTGATGATGAGCTGACTCGCTTTGAGGCCGTCGTACAGACACTTTCAG
CGGATGACCCAGGACGCCAGGCCGTAGCAGATCGCTTAGATGCTTTGGTGGCATCACTTCGT
CGCAATTCCGCGCCACAGGAAAATTTTAGTGACGAAGACATCGAATCCGTGTCGGTGGACCG
CCTTTTAGATATTATCGATGAAGAGTTCGAGATTAGCTAACAAAGCCCGAAAGGAAGCTGAG
TTGGCTGCTGCCACCGCTGAGCAATAACTAGCATAACCCCTTGGGGCCTCTAAACGGGTCTT
GAGGGGTTTTTTACTGCTACTTAATACGACTCACTATAGGGGAATTGTGAGCGGATAACAAT
TCCCCTGTAGAAATAATTTTGTTTAACTTTAATAAGGAGATATACCATGCCGGAACCCCAGC
AGAACCAGCAGGAAAAAGTTGTCGACTACCTTCGCCGGGTCACCAACGATCTGCGACGCGCC
CGCCGCCGGATAGGCGAGCTGGAATCCCGCGACAACGAGCCAATTGCGTAGGTACCAGACTC
TAGCTCTAGAGACATAAAT

AAGCTT- HindIII site
ACTAGT- SpeI site
GCTAGC- BmtI site
CTAGCATAACCCCTTGGGGCCTCTAAACGGGTCTTGAGGGGTTTTTT- T7 terminator
TAATACGACTCACTATAGG- T7 promoter
GGAATTGTGAGCGGATAACAATTC- LacO
AAGGAG- RBS
CAATTG- MfeI site
TCTAGA- XbaI site

NNNNNNN Docking domains

Figure S3. Regulator sequences. gBlocks were inserted between the HindIII and XbaI sites of pUC19 to construct cloning plasmids containing the **S3**, **S5**, **S8**, **E5**, **P3**, **P5**, **P6**, and **A1** docking domains.

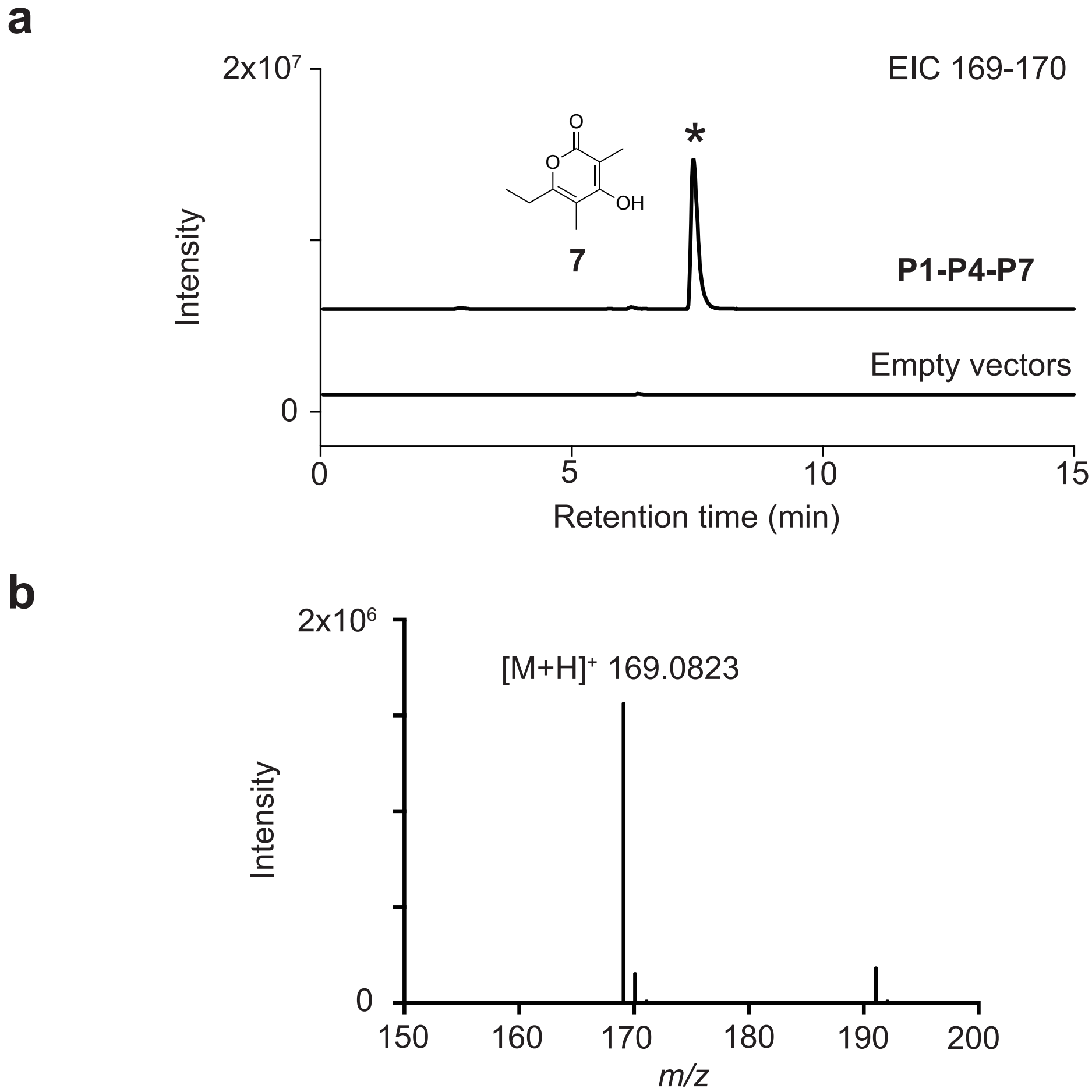

Figure S4. LC/MS analysis of the anticipated **P1**-**P4**-**P7** product, **7**. a) Extracted-ion chromatogram (EIC) for the $[M+H]^+$ of **7** from the ethyl acetate extract of cells transformed with the PKS expression plasmids or corresponding empty vectors. b) MS spectrum of the main peak (*). Theoretical $[M+H]^+$ *m/z* of **7,** 169.0859; observed *m/z*, 169.0823 (2.3 ppm).

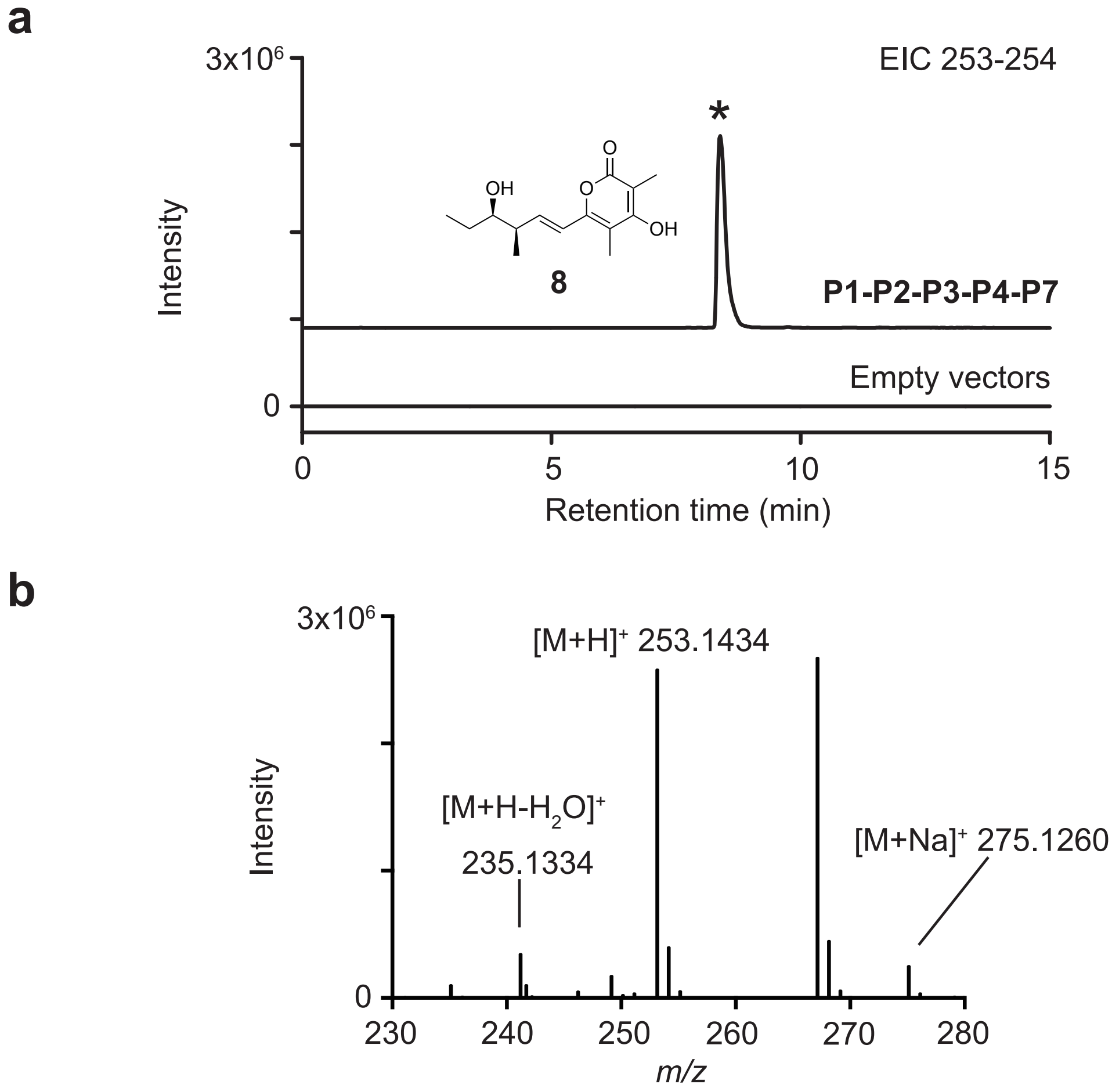

Figure S5. LC/MS analysis of the anticipated **P1-P2-P3-P4-P7** product, **8**. a) EIC for the $[M+H]^+$ of **8** from the ethyl acetate extract of cells transformed with the PKS expression plasmids or corresponding empty vectors. b) MS spectrum of the main peak (*). Theoretical $[M+H]^+$ *m/z* of **8,** 253.1434; observed *m/z*, 253.1434 (0 ppm).

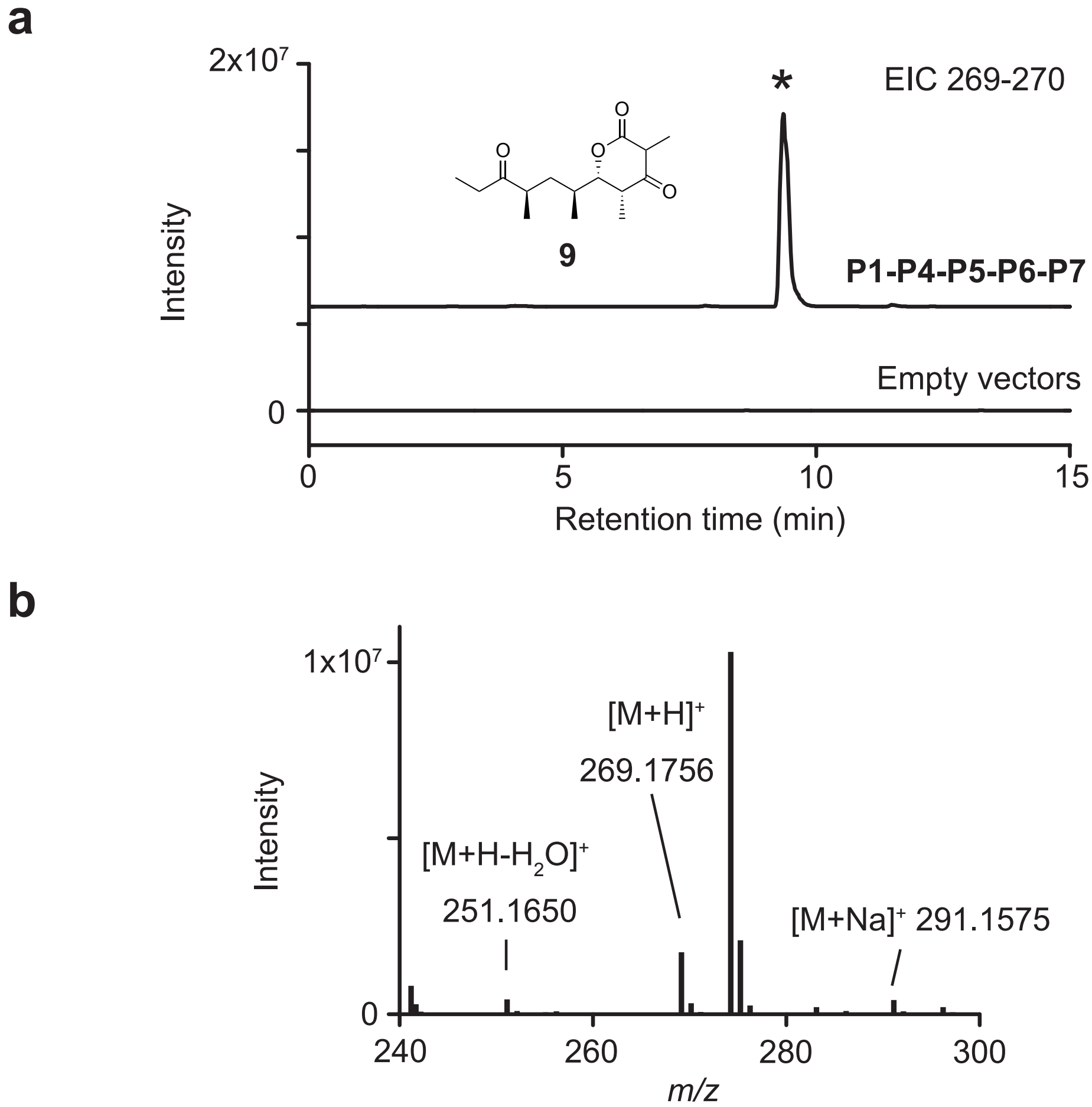

Figure S6. LC/MS analysis of the anticipated **P1-P4-P5-P6-P7** product, **9**. a) EIC for the $[M+H]^+$ of **9** from the ethyl acetate extract of cells transformed with the PKS expression plasmids or corresponding empty vectors. b) MS spectrum of the main peak (*). Theoretical $[M+H]^+$ *m/z* of **9,** 269.1747; observed *m/z*, 269.1575 (3.3 ppm).

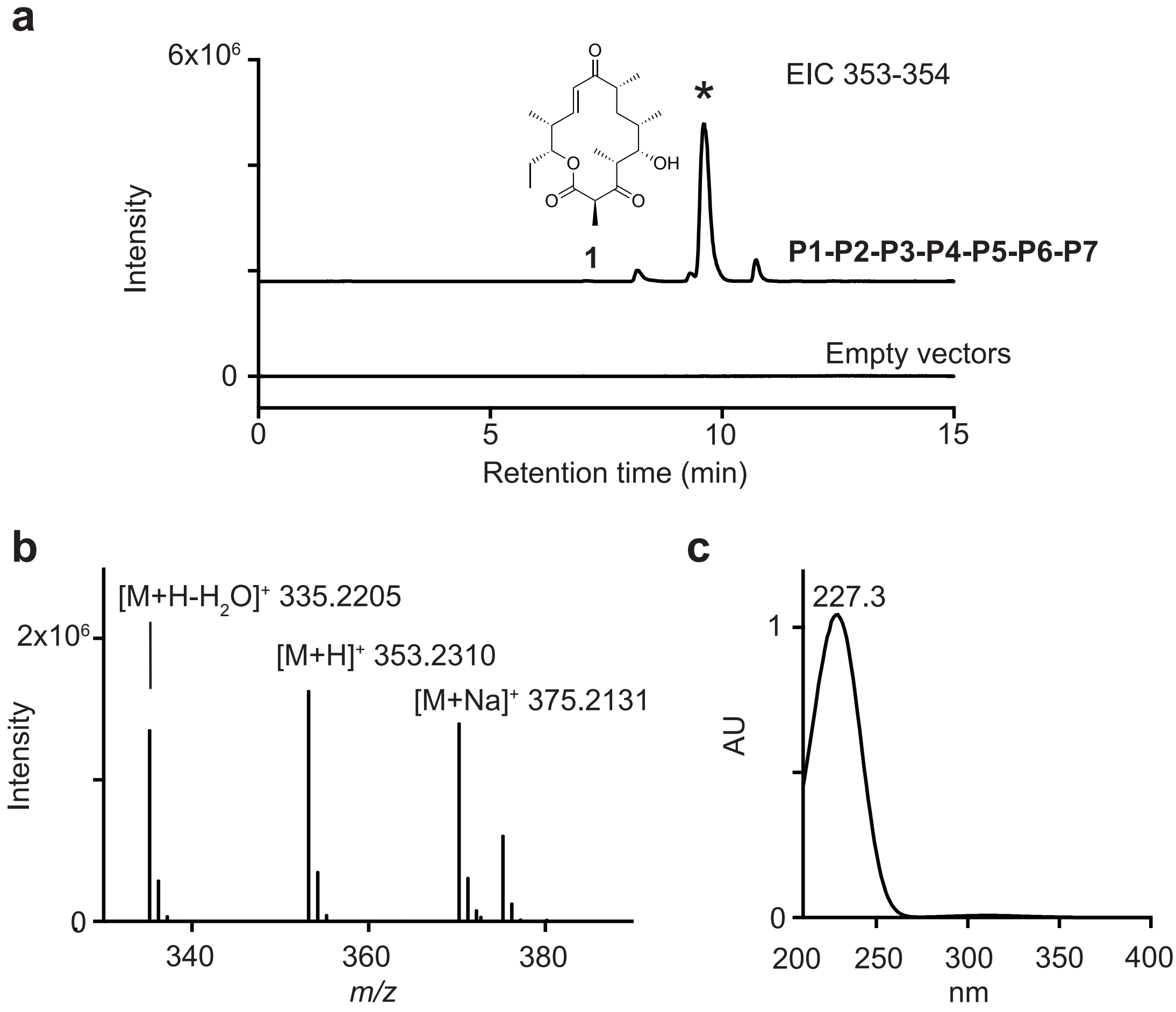

Figure S7. LC/MS analysis of the anticipated **P1-P2-P3-P4-P5-P6-P7** product, narbonolide (**1**). a) EIC for the $[M+H]^+$ of **1** from the ethyl acetate extract of cells transformed with the PKS expression plasmids or corresponding empty vectors. b) MS spectrum of the main peak (*). Theoretical $[M+H]^+$ *m/z* of **1,** 353.2322; observed *m/z*, 353.2310 (-3.3 ppm). c) Absorbance spectrum of **1**.

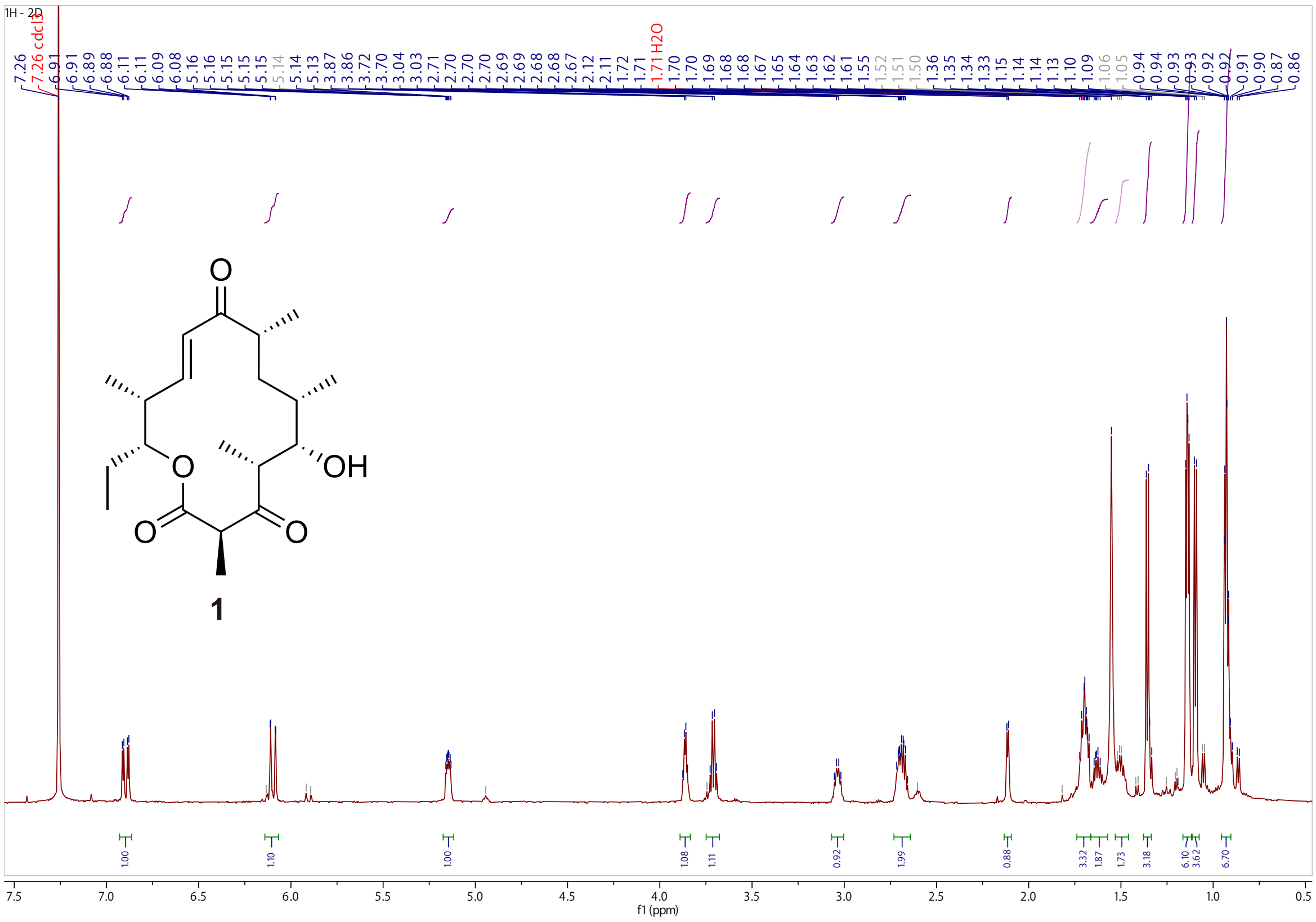

Figure S8. ^{1}H NMR spectrum of the anticipated **P1-P2-P3-P4-P5-P6-P7** product, narbonolide (**1**).

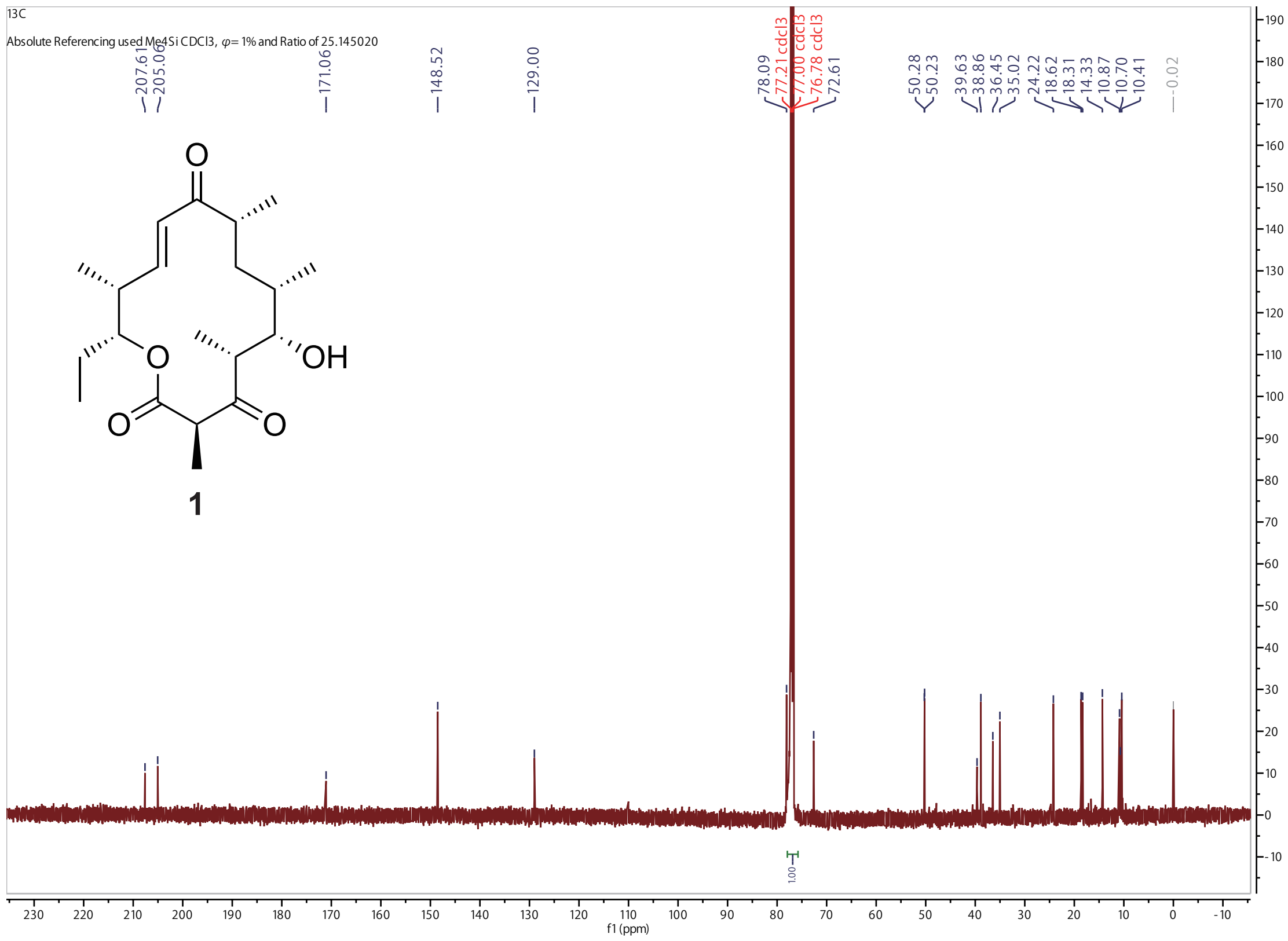

Figure S9. ^{13}C NMR spectrum of the anticipated **P1-P2-P3-P4-P5-P6-P7** product, narbonolide (**1**).

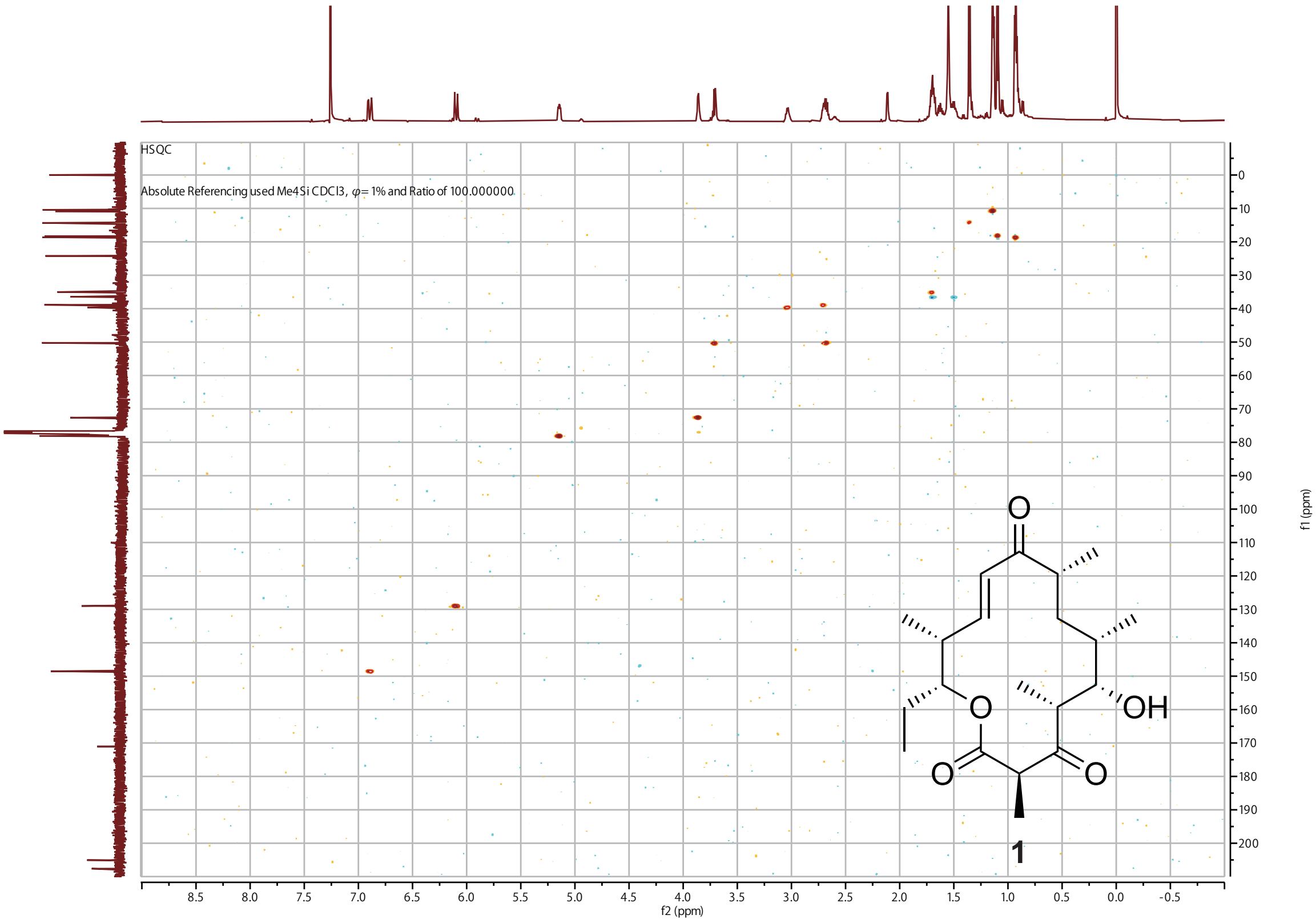

Figure S10. ^{1}H-^{13}C HSQC spectrum of the anticipated **P1-P2-P3-P4-P5-P6-P7** product, narbonolide (**1**).

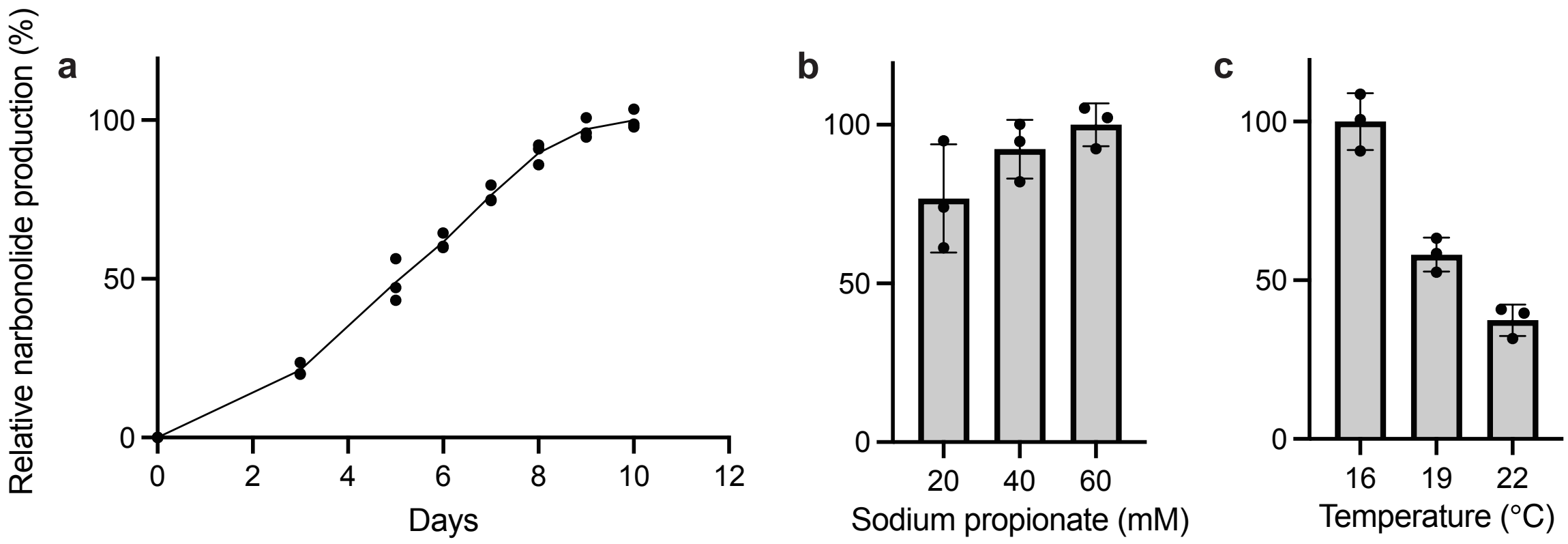

Figure S11. Optimization of narbonolide production. a) Time course of narbonolide production. b) Relative production of narbonolide at different concentrations of sodium propionate. c) Relative production of narbonolide at different temperatures. Data are mean ± s.d., n = 3 independent experiments (Supplementary Data 4).

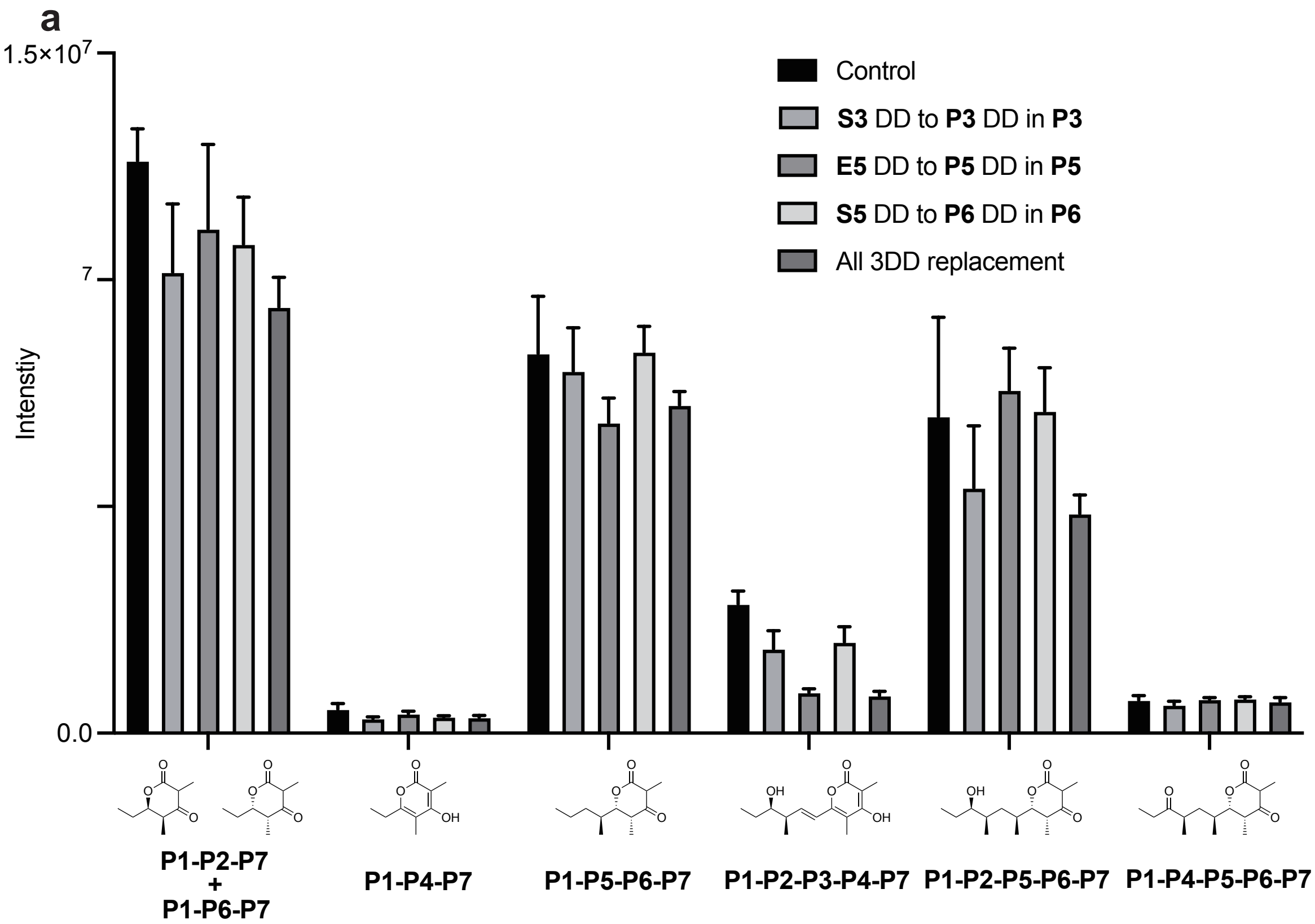

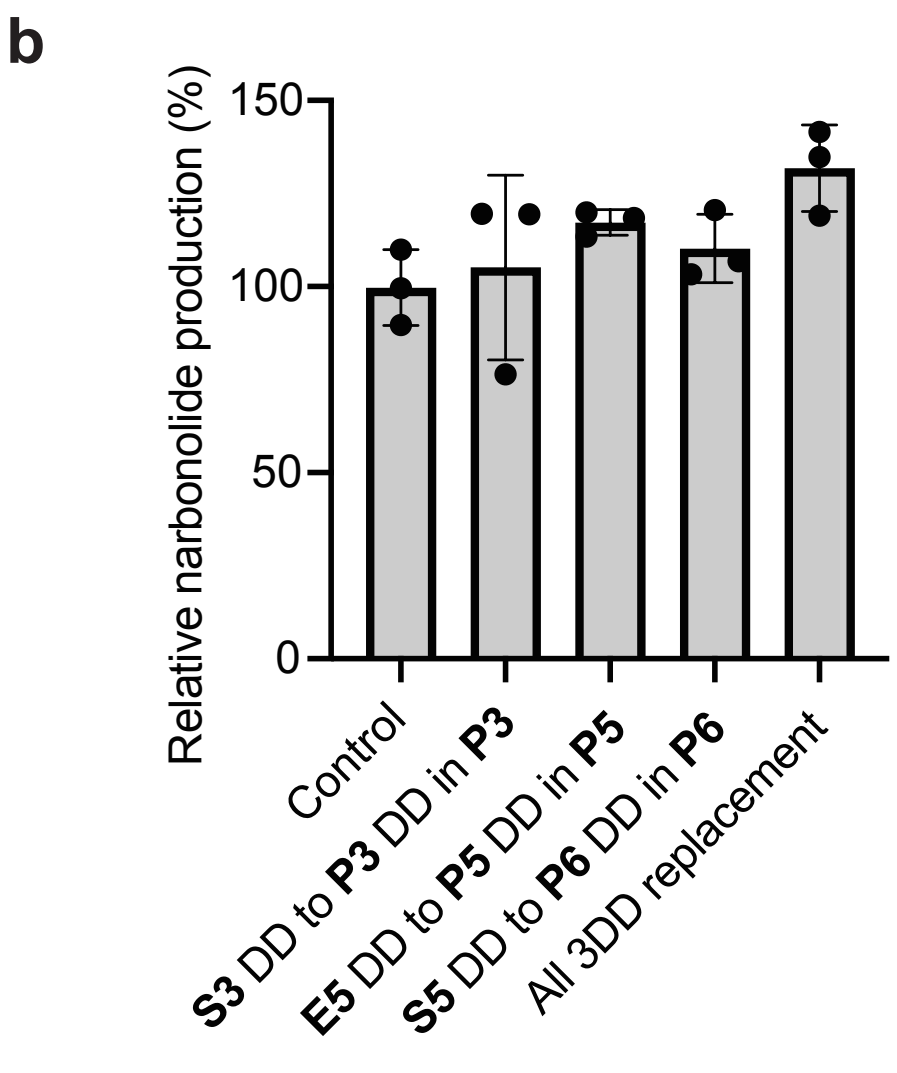

Figure S12. Replacement of the docking domain pairs in the refactored pikromycin PKS and analysis of shunt products. a) Shunt products analysis of the refactored pikromycin PKSs and their docking domain(DD)-swapped variants. Shunt product accumulation was quantified based on the peak area of EIC of the shunt products[2]. b) Comparison of the narbonolide productivities between the DD-swapped variants. Data are mean ± s.d., n = 3 independent experiments (Supplementary Data 5).

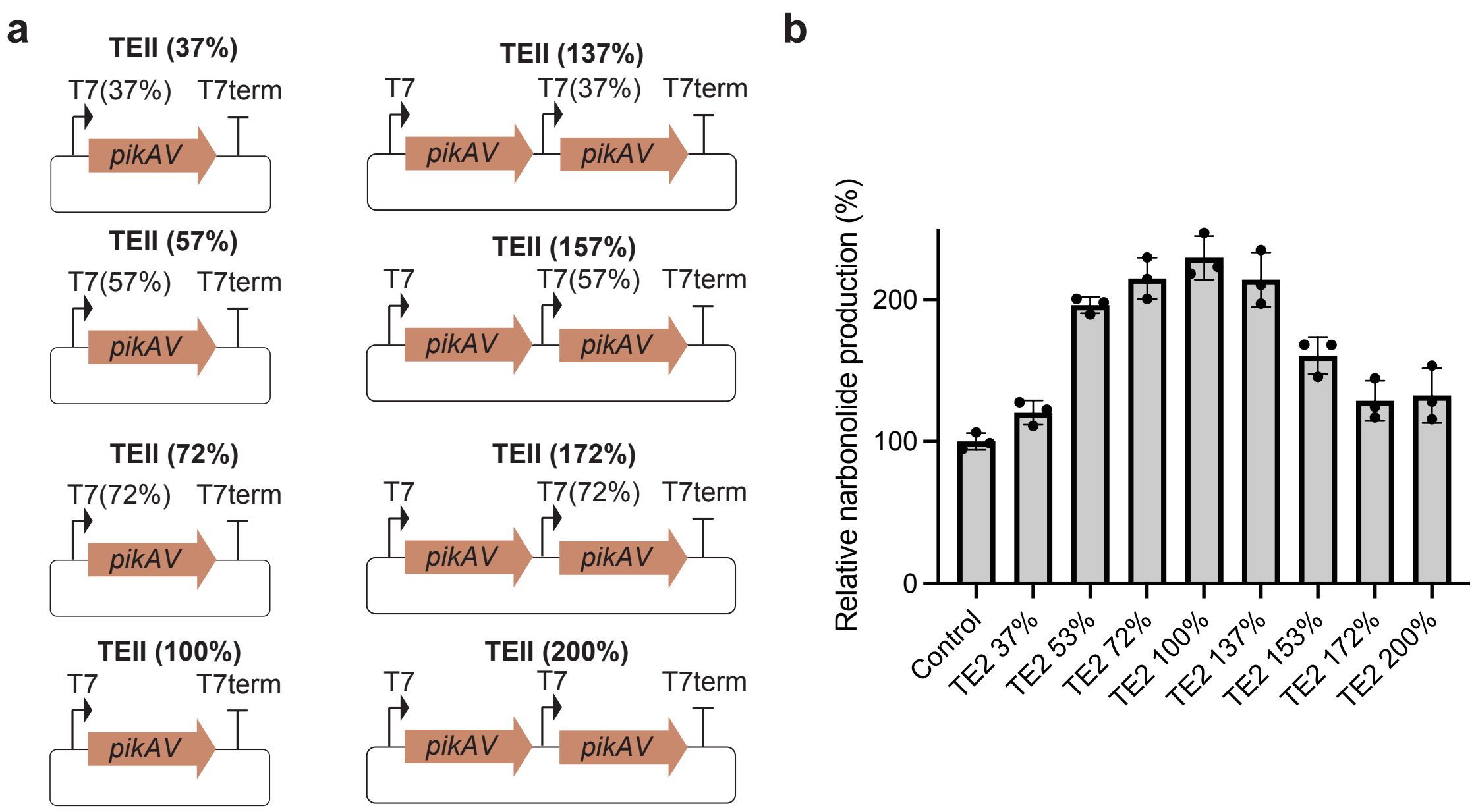

Figure S13. Analysis of the effect of TEII expression level on narbonolide production. a) A series of pACYC-derived *pikAV* expression vectors with different expression levels were co-transformed with the plasmids encoding the refactored pikromycin synthase. Expression of PikAV is controlled by the T7 promoter and attenuated T7 promotors[7]. b) Relative production of narbonolide was quantified by LC/MS. Data are mean ± s.d., n = 3 independent experiments (Supplementary Data 6).

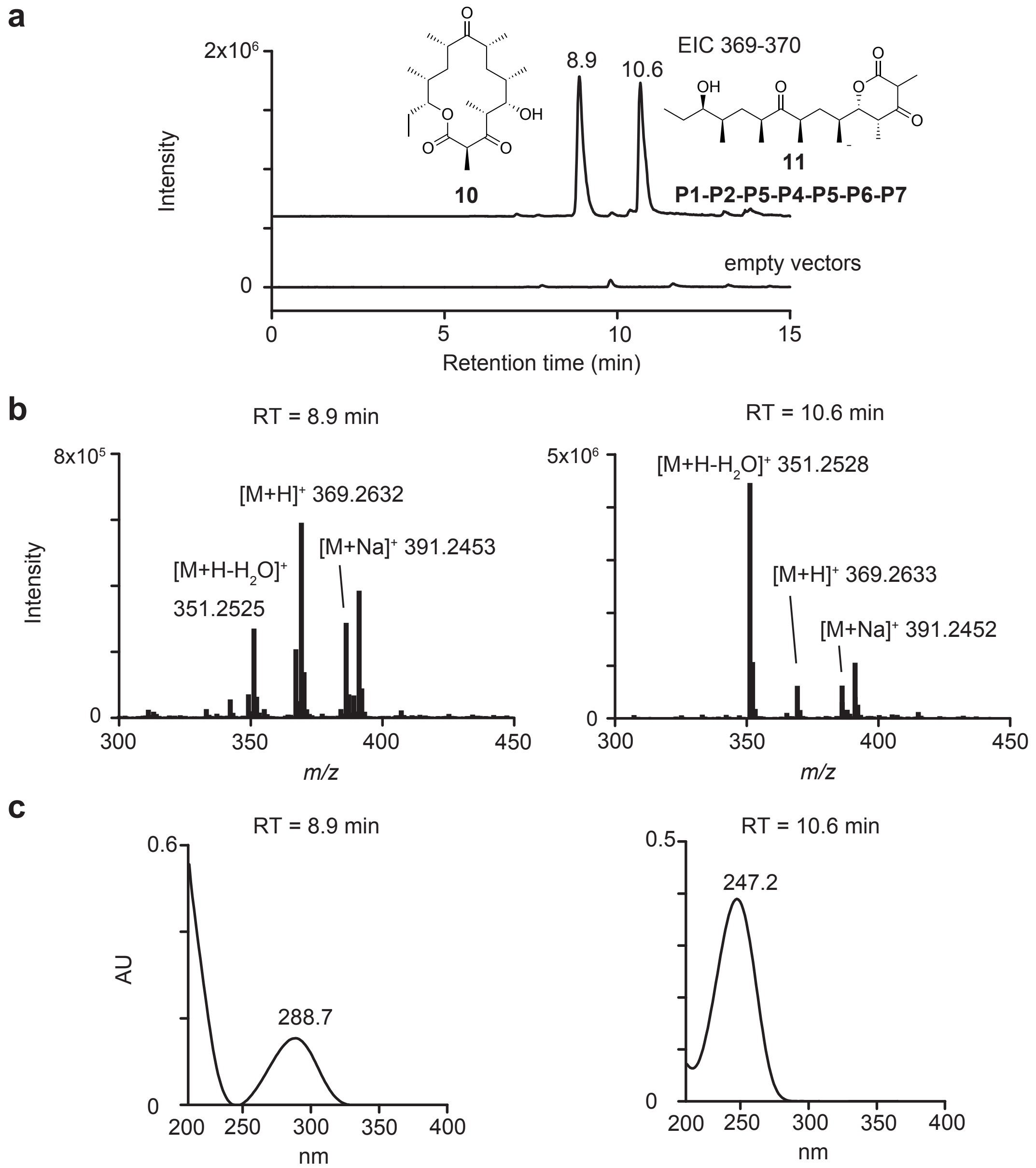

Figure S14. LC/MS analysis of the anticipated **P1-P2-P5-P4-P5-P6-P7** product, **10**, and byproduct **11**. a) EIC for the $[M+H]^+$ of **10** from the ethyl acetate extract of cells transformed with the PKS expression plasmids or corresponding empty vectors. b) MS spectra of the two main peaks (RT = 8.9 and 10.6 min). Theoretical $[M+H]^+$ *m/z* of **10**, 369.2635; observed *m/z*, 369.2632 (-0.8 ppm). Theoretical $[M+H]^+$ *m/z* of **11**, 369.2635; observed *m/z*, 369.2633 (-0.5 ppm). c) UV absorbance spectra correlated with the main peaks. The λ_{max} of **11** is characteristic of β-keto δ-lactones.

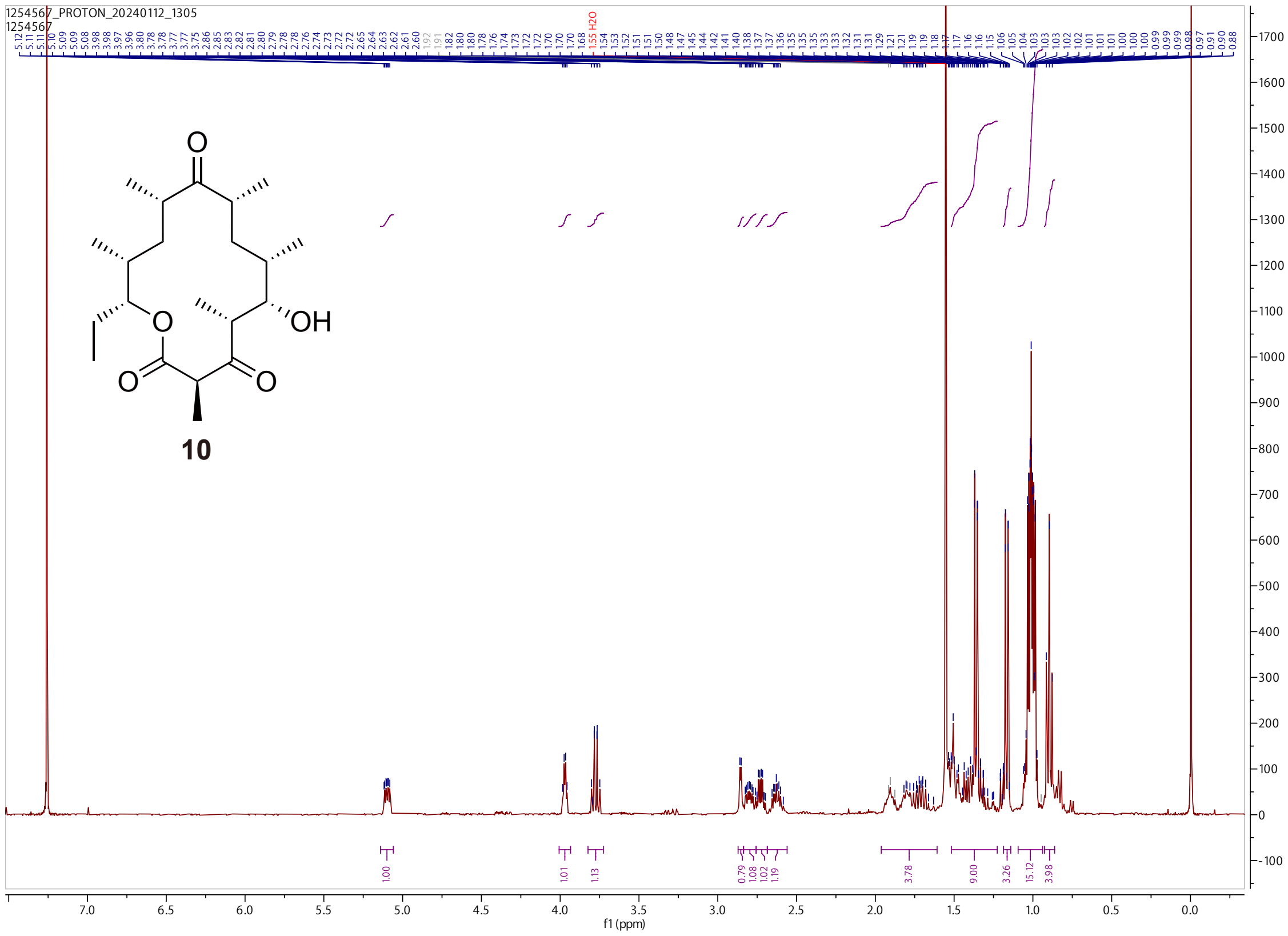

Figure S15. ^{1}H NMR spectrum of anticipated **P1-P2-P5-P4-P5-P6-P7** product, **10**.

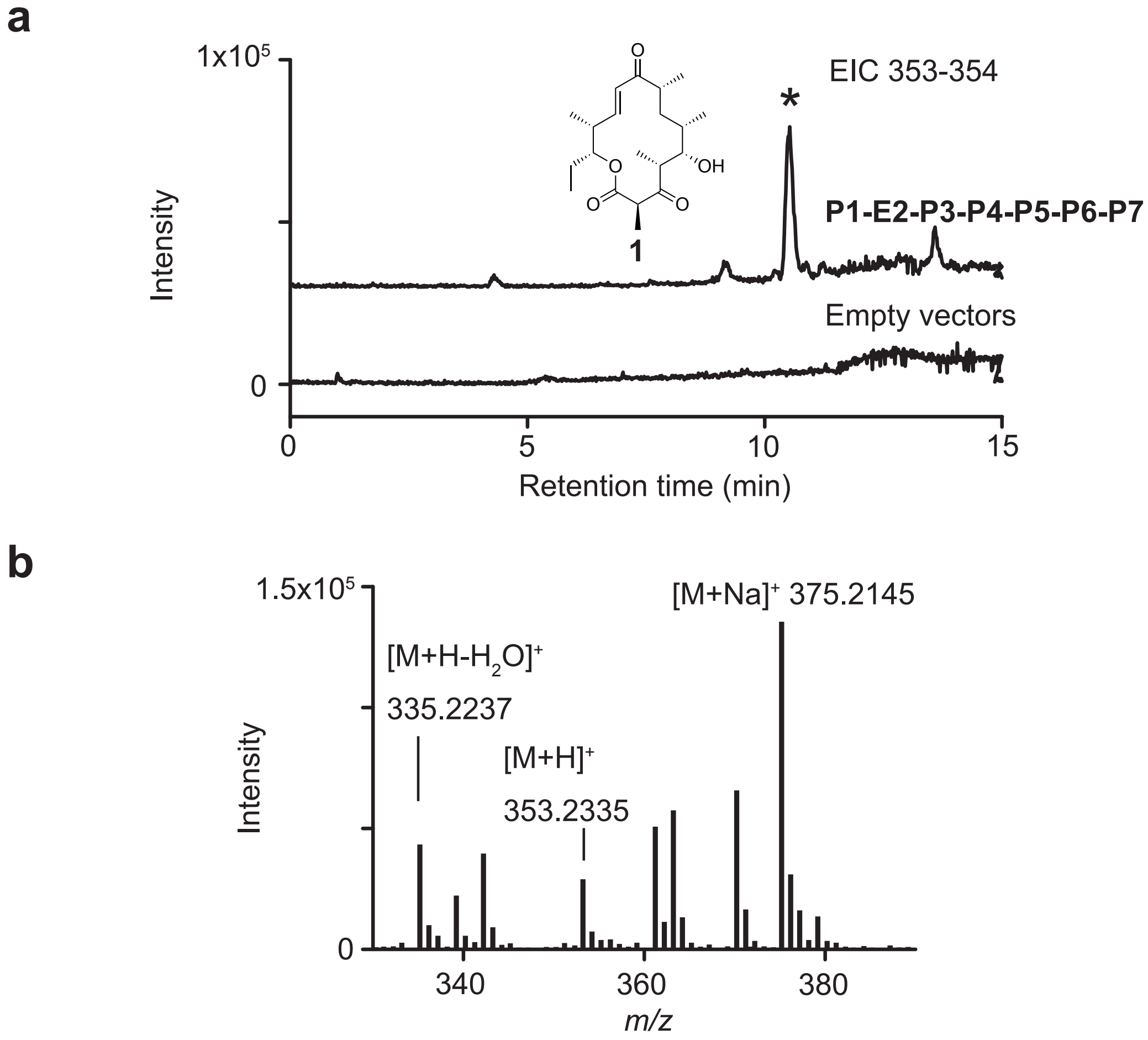

Figure S16. LC/MS analysis of the anticipated **P1-E2-P3-P4-P5-P6-P7** product, narbonolide (**1**). a) EIC for the $[M+H]^+$ of **1** from the ethyl acetate extract of cells transformed with the PKS expression plasmids or corresponding empty vectors. b) MS spectrum of the main peak (*). Theoretical $[M+H]^+$ *m/z* of **1**, 353.2317; observed *m/z*, 353.2335 (3.6 ppm).

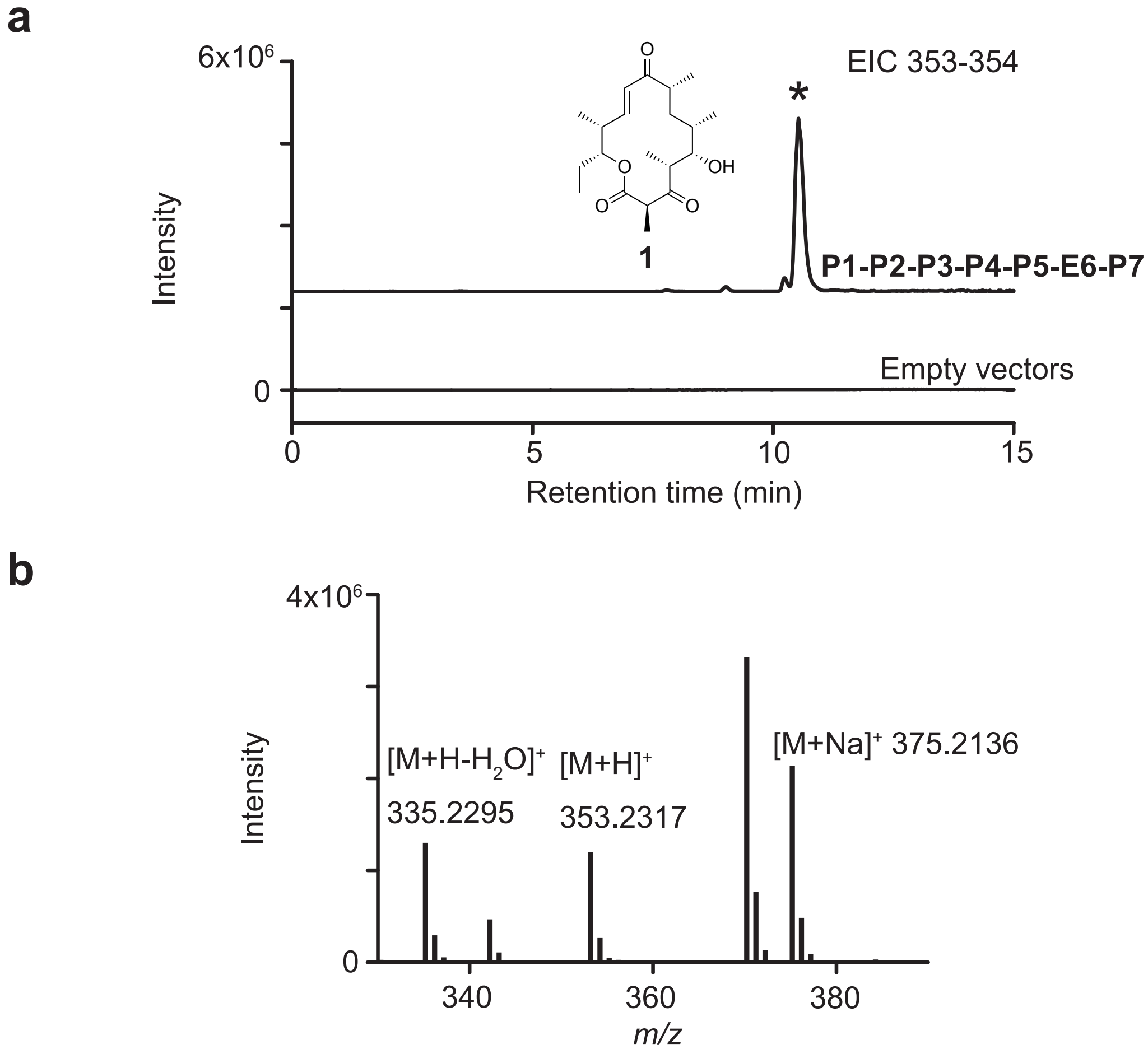

Figure S17. LC/MS analysis of the anticipated **P1-P2-P3-P4-P5-E6-P7** product, narbonolide (**1**). a) EIC for the $[M+H]^+$ of **1** from the ethyl acetate extract of cells transformed with the PKS expression plasmids or corresponding empty vectors. b) MS spectrum of the main peak (*). Theoretical $[M+H]^+$ *m/z* of **1**, 353.2317; observed *m/z*, 353.2317 (-1.4 ppm).

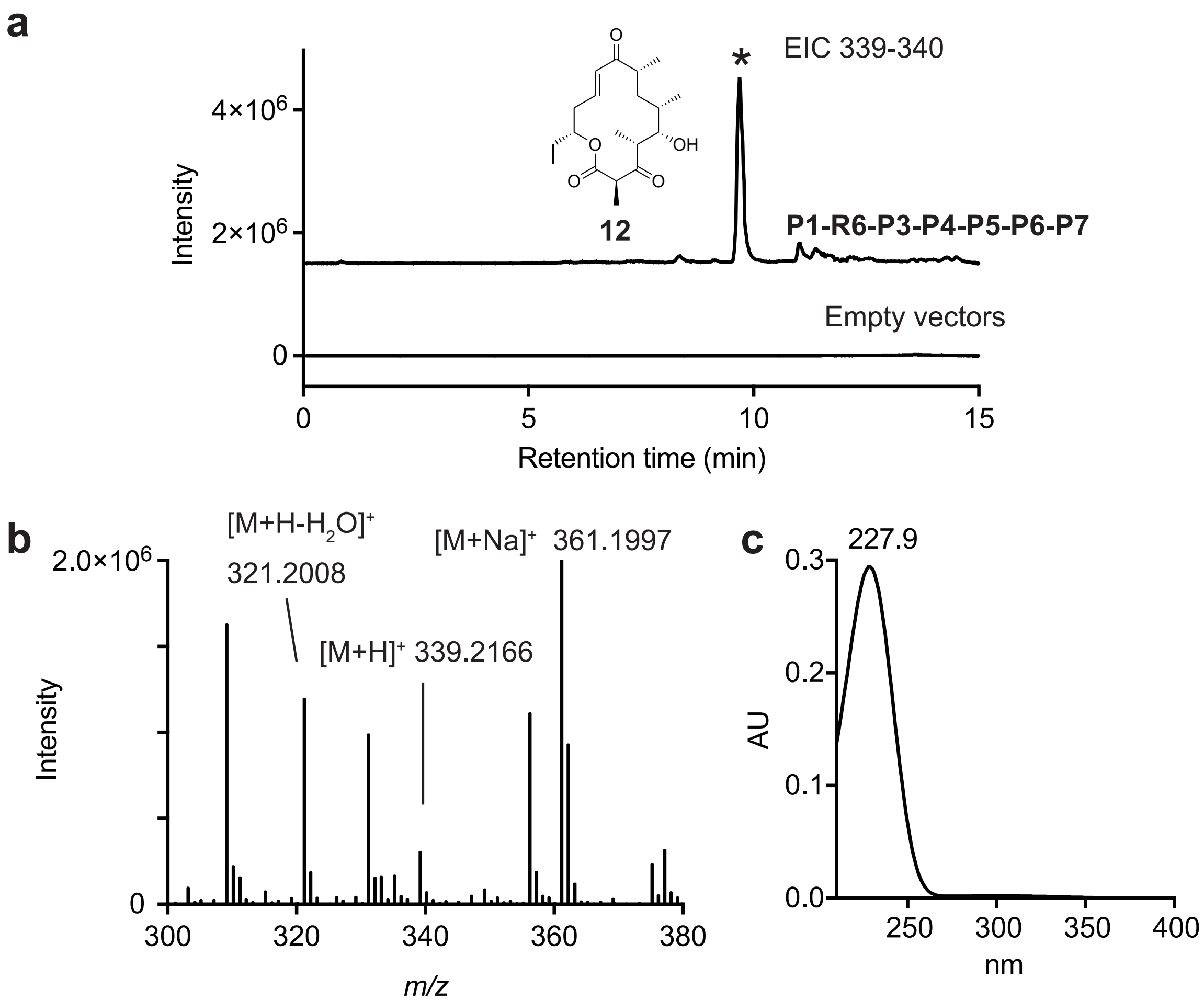

Figure S18. LC/MS analysis of the anticipated **P1-R6-P3-P4-P5-P6-P7** product, **12**. a) EIC for the $[M+H]^+$ of **12** from the ethyl acetate extract of cells transformed with the PKS expression plasmids or corresponding empty vectors. b) MS spectrum of the main peak (*). Theoretical $[M+H]^+$ *m/z* of **12**, 339.2166; observed *m/z*, 339.2166 (0 ppm). c) UV absorbance spectrum for the main peaks. The λ_{max} of 227.9 nm is similar to that of narbonolide (Fig. S7c).

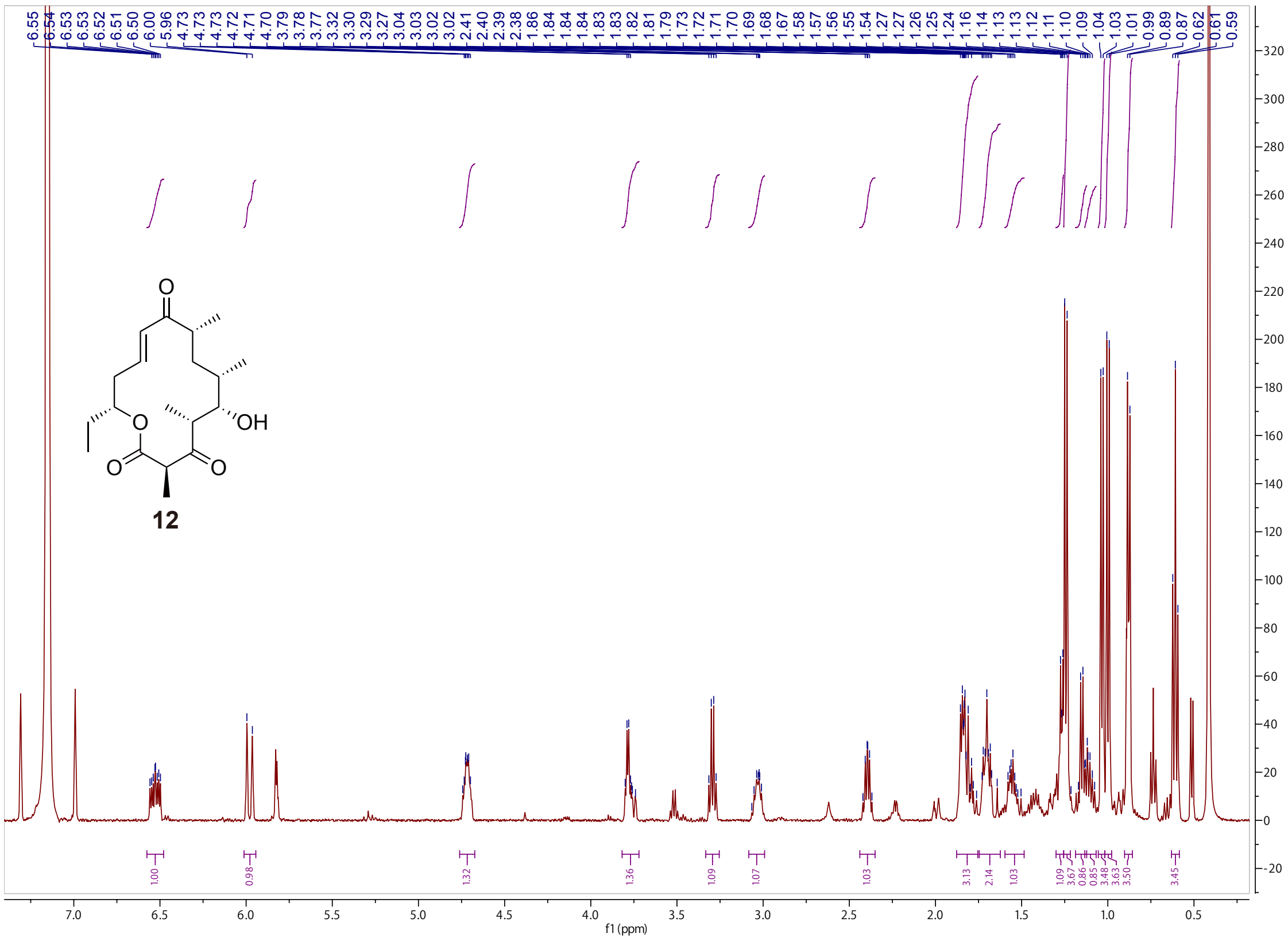

Figure S19. ^{1}H NMR spectrum of the **P1-R6-P3-P4-P5-P6-P7** product, **12**.

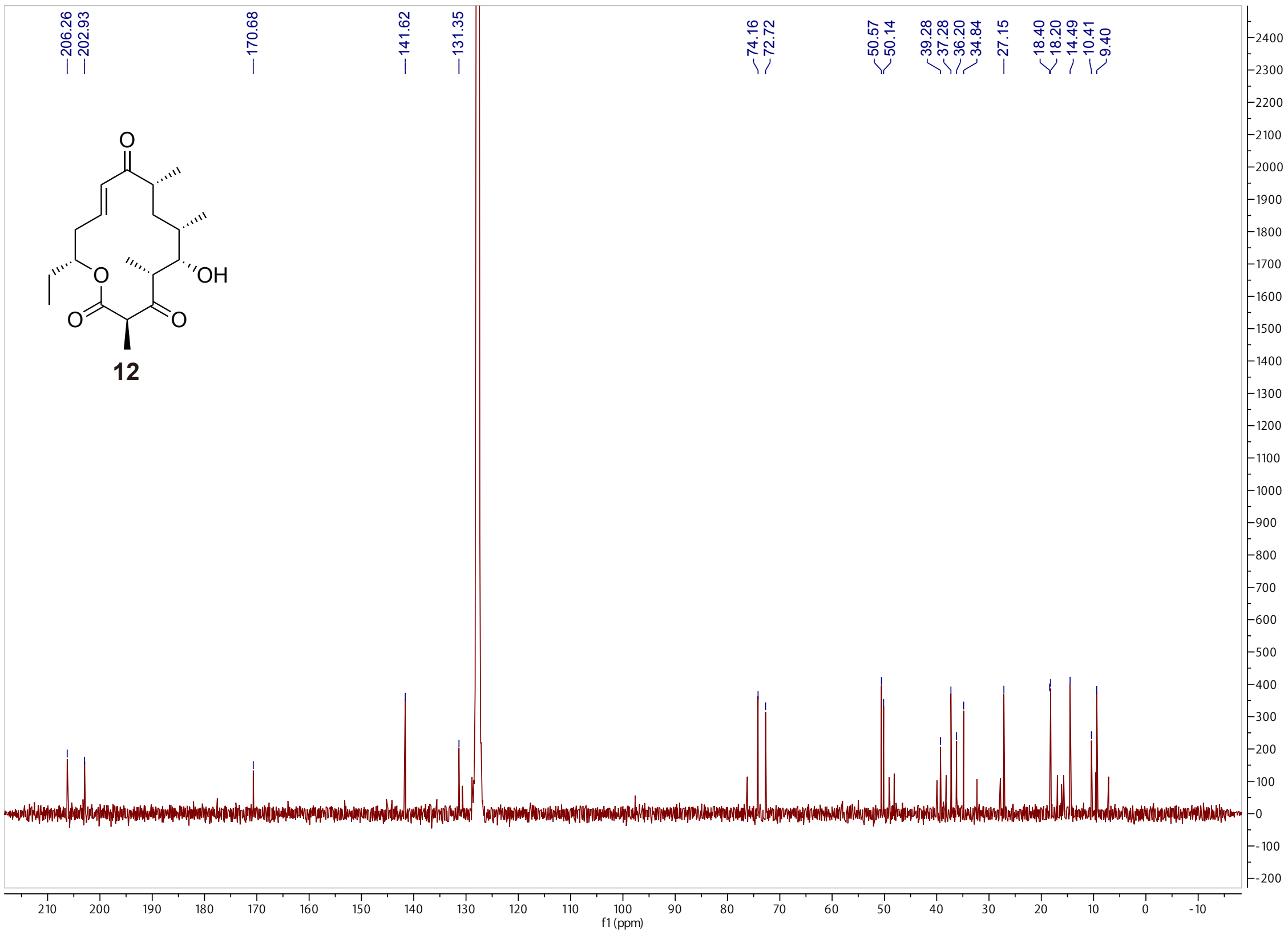

Figure S20. ^{13}C NMR spectrum of the **P1-R6-P3-P4-P5-P6-P7** product, **12**.

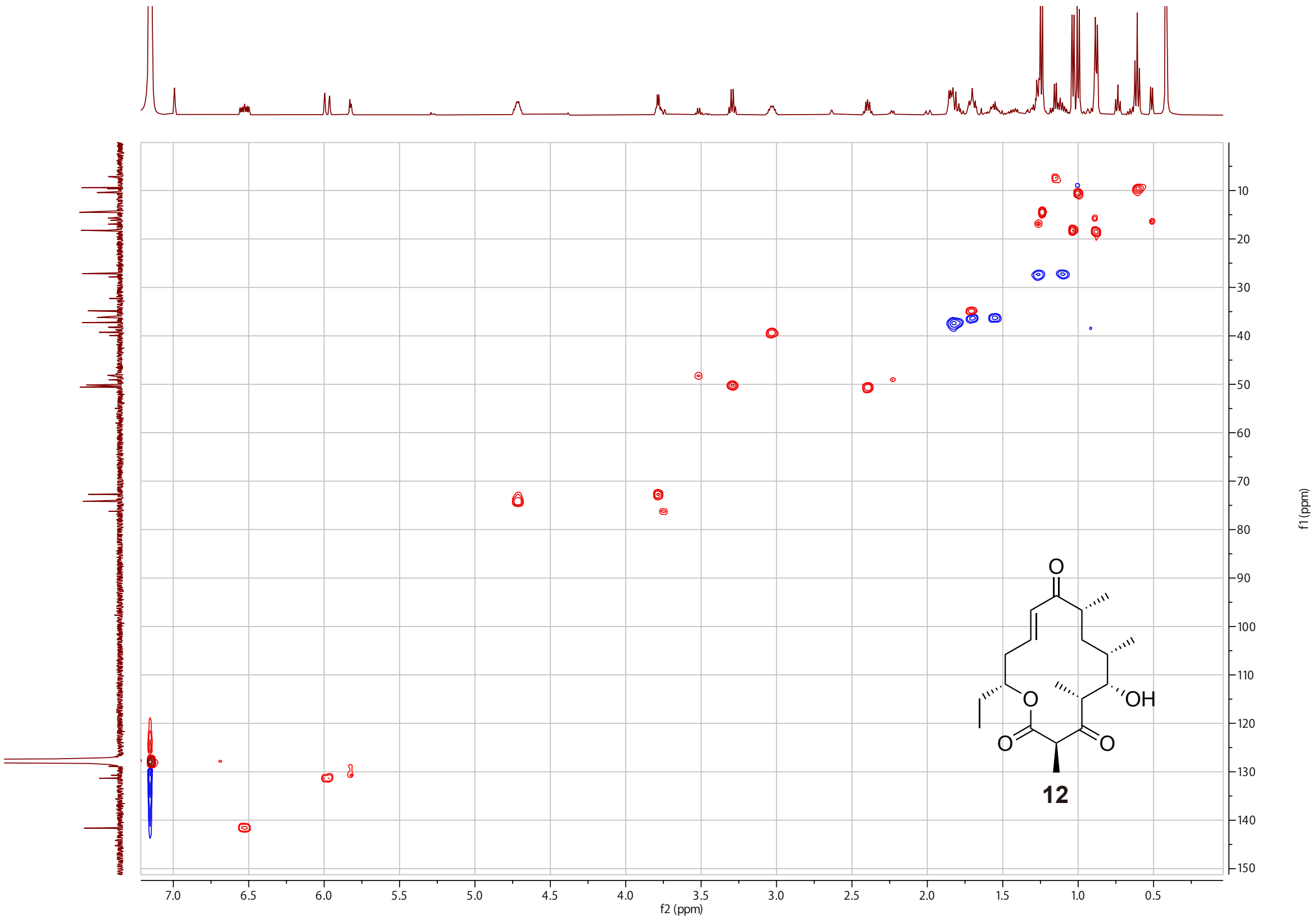

Figure S21. ^{1}H-^{13}C HSQC spectrum of the **P1-R6-P3-P4-P5-P6-P7** product, **12**.

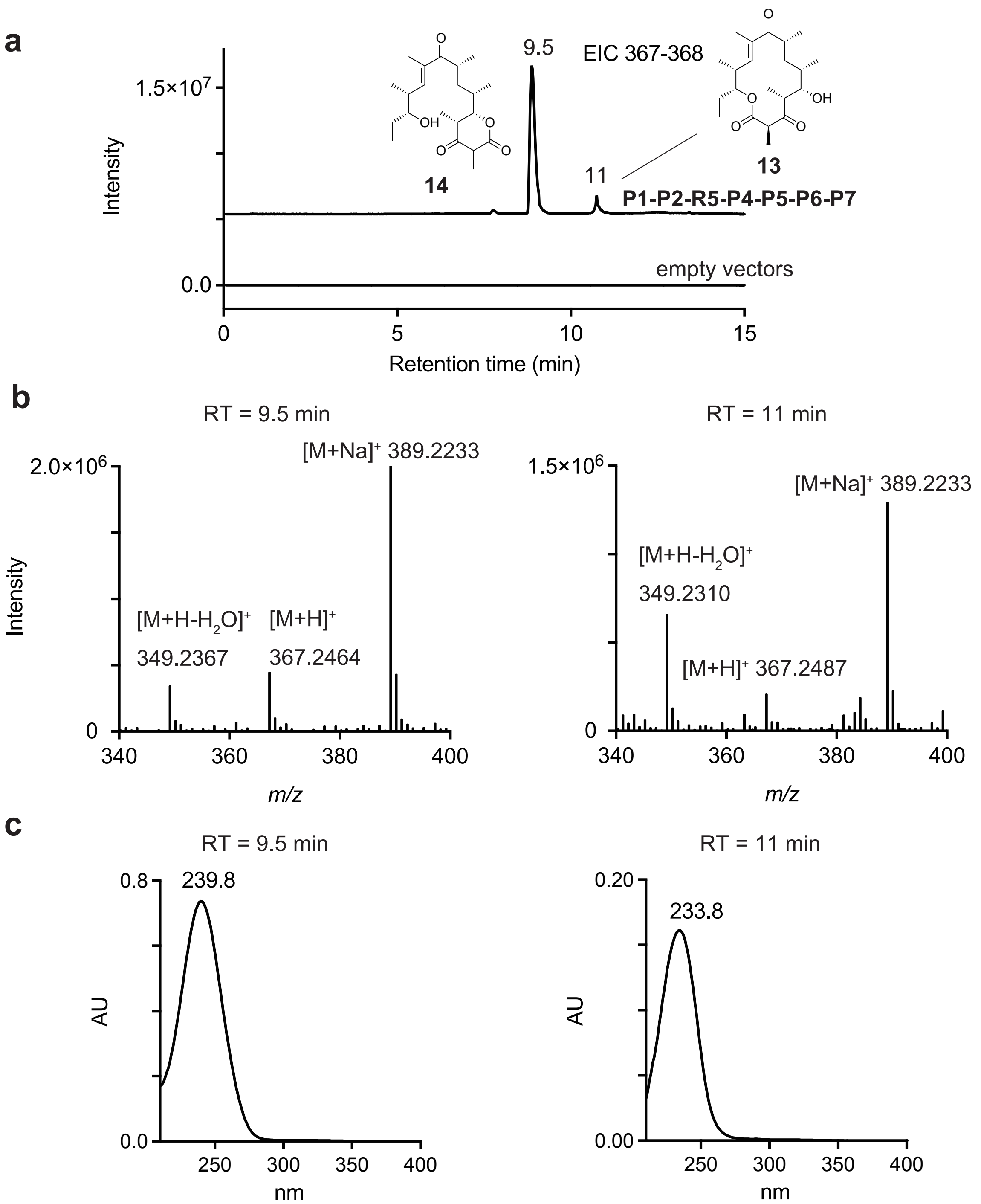

Figure S22. LC/MS analysis of the anticipated **P1-P2-R5-P4-P5-P6-P7** product, **13**, and **14**. a) EIC for the $[M+H]^+$ of **13** and **14** from the ethyl acetate extract of cells transformed with the PKS expression plasmids or corresponding empty vectors. b) MS spectrum of the main peaks (RT = 9.5 and 11 min). Theoretical $[M+H]^+$ *m/z* of **13** and **14**, 367.2479; observed *m/z*, 367.2464 (-4 ppm, RT = 9.5 min) and 367.2487(2.1 ppm, RT = 11 min). c) UV absorbance spectrum for the main peaks.

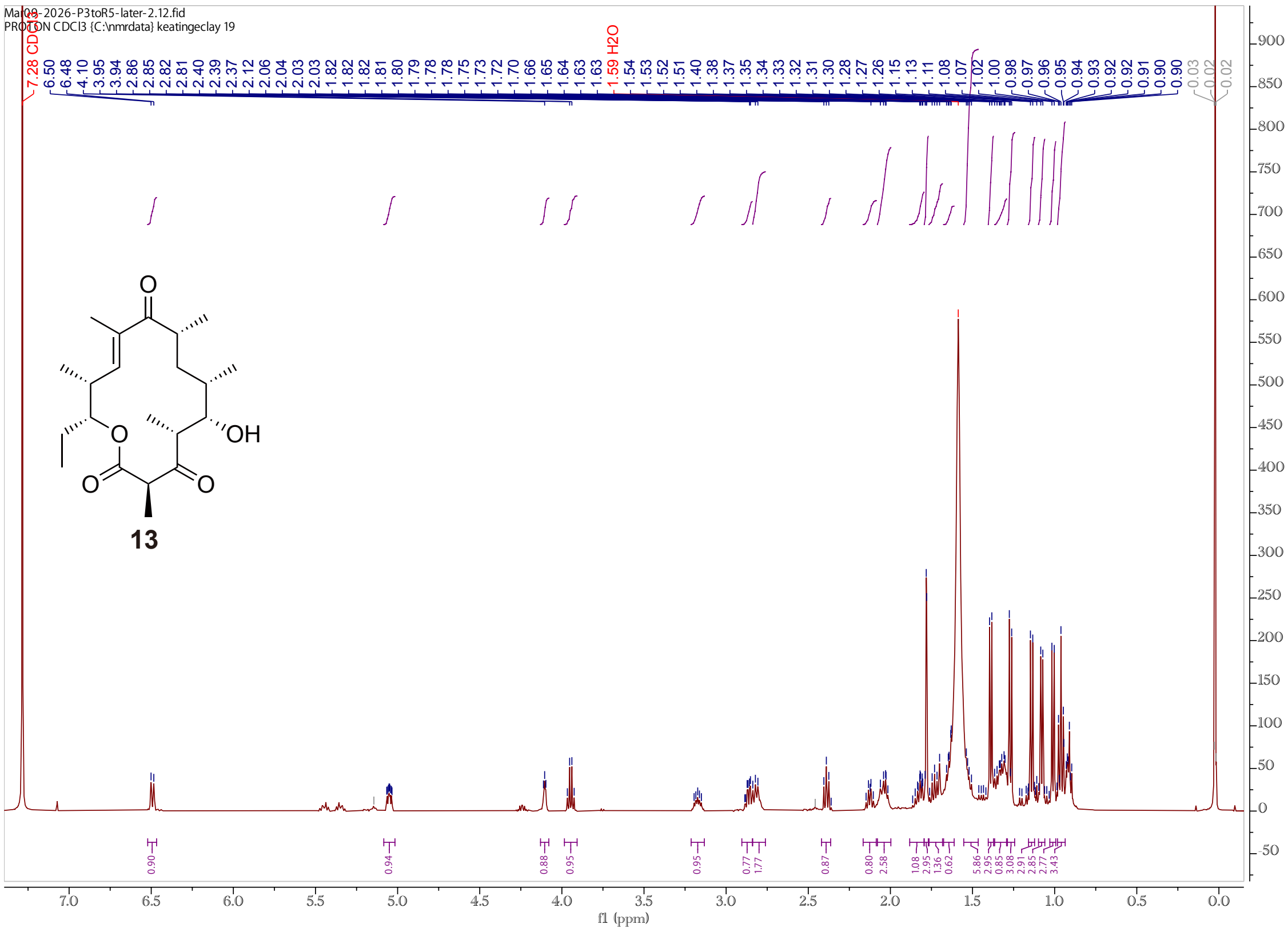

Figure S23. ^{1}H NMR spectrum of the **P1-P2-R5-P4-P5-P6-P7** product **13**.

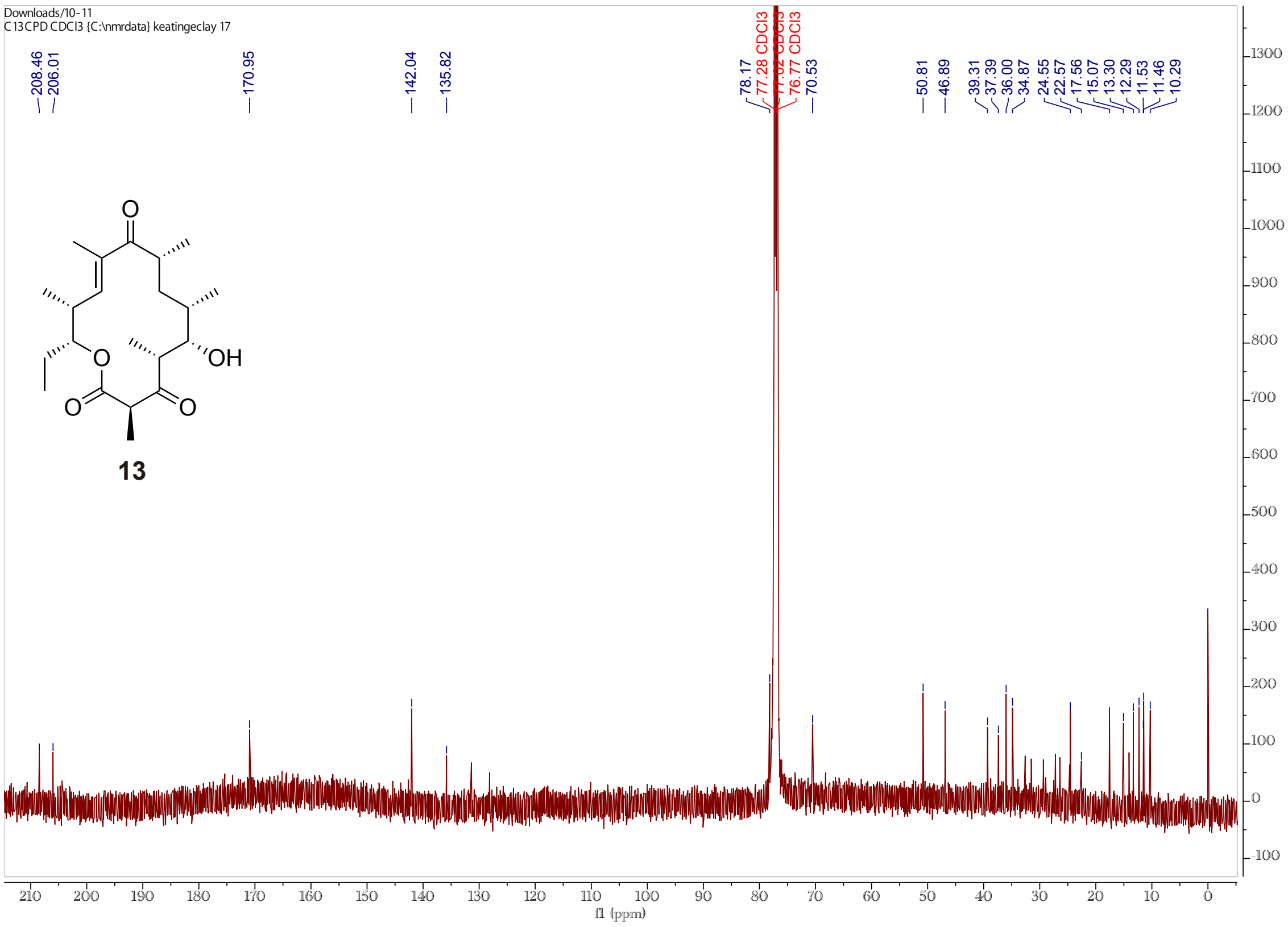

Figure S24. ^{13}C NMR spectrum of the **P1-P2-R5-P4-P5-P6-P7** product **13**.

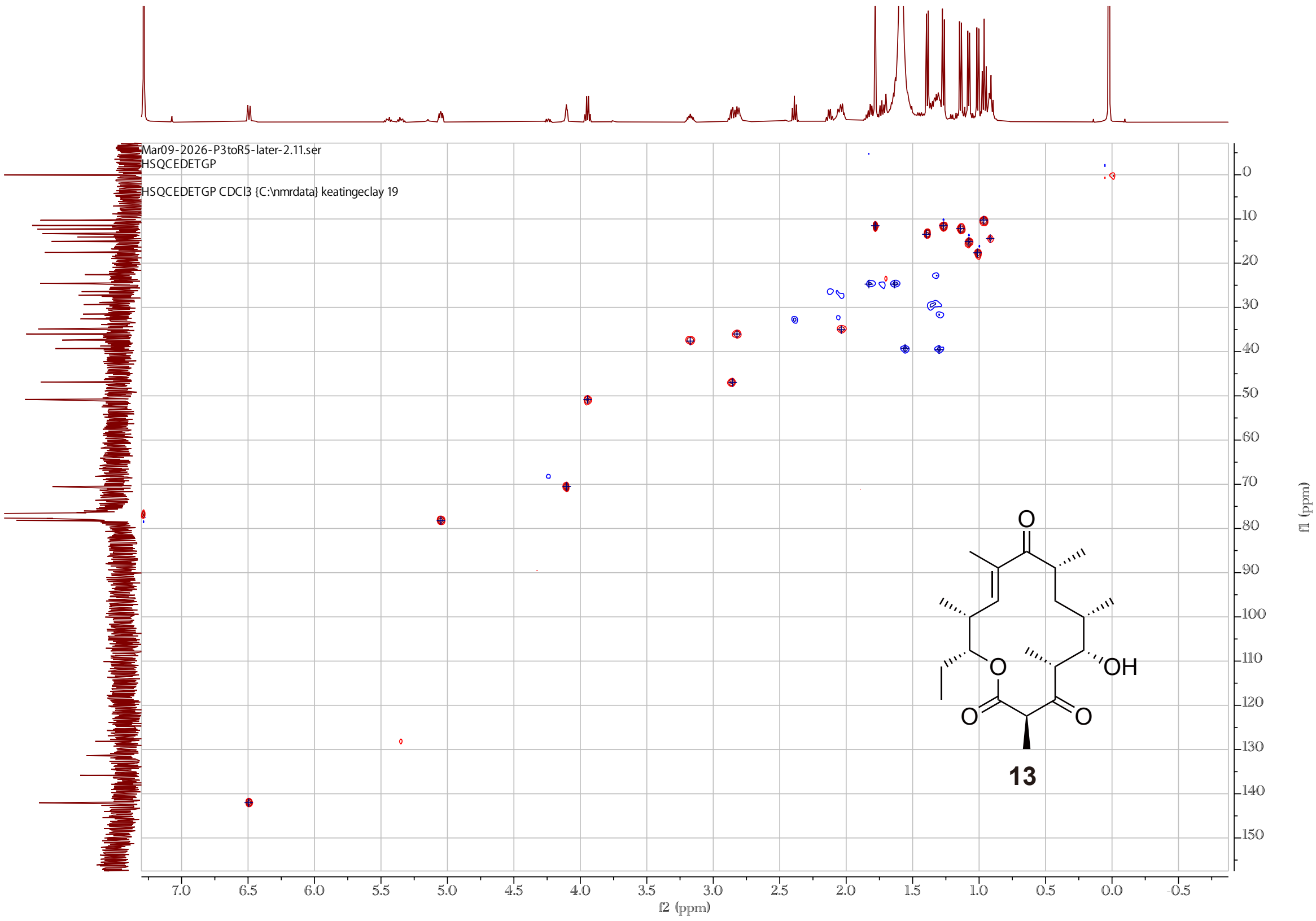

Figure S25. ^{1}H-^{13}C HSQC spectrum of the **P1-P2-R5-P4-P5-P6-P7** product **13**.

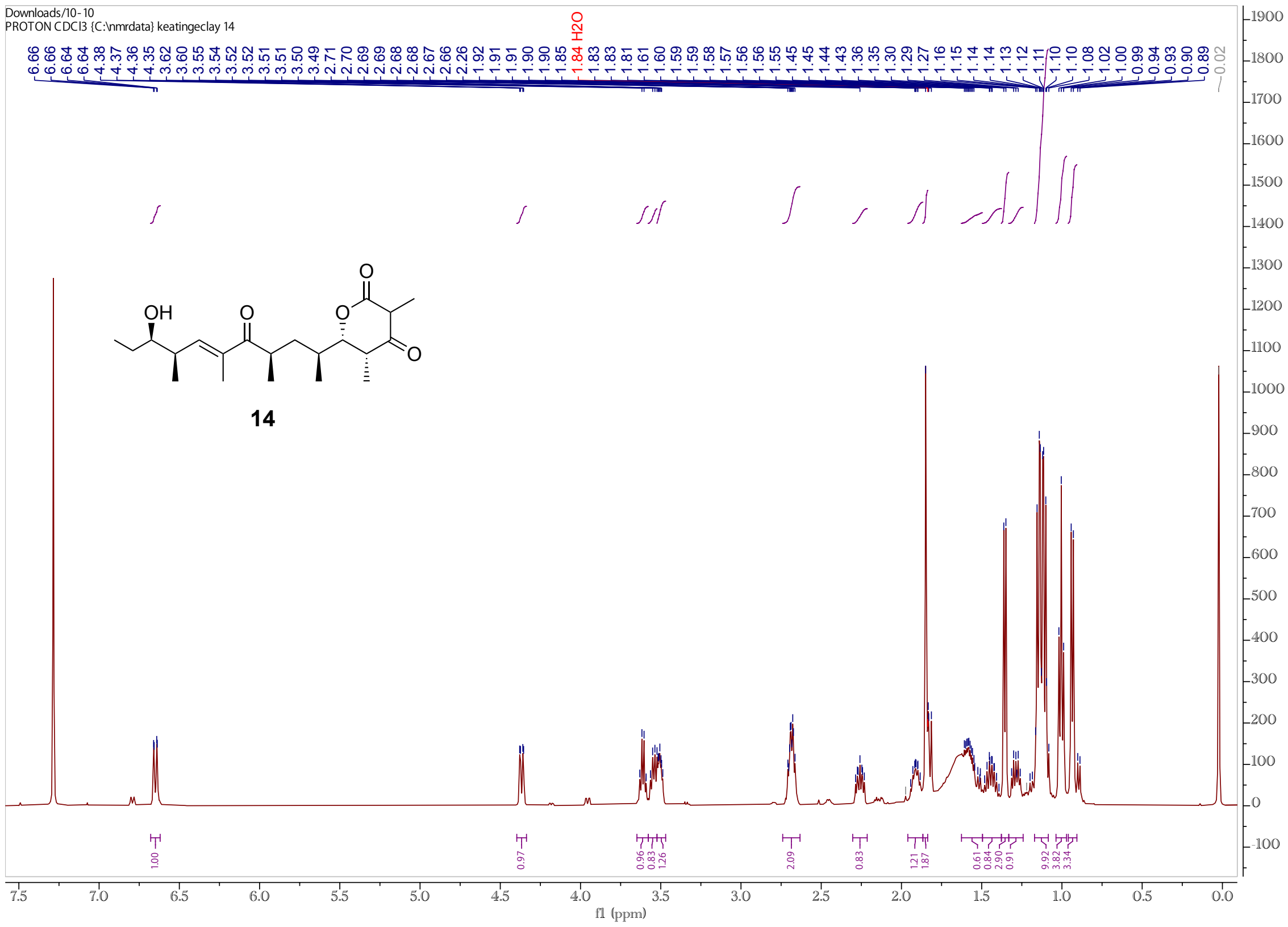

Figure S26. ^{1}H NMR spectrum of the **P1-P2-R5-P4-P5-P6-P7** product **14**.

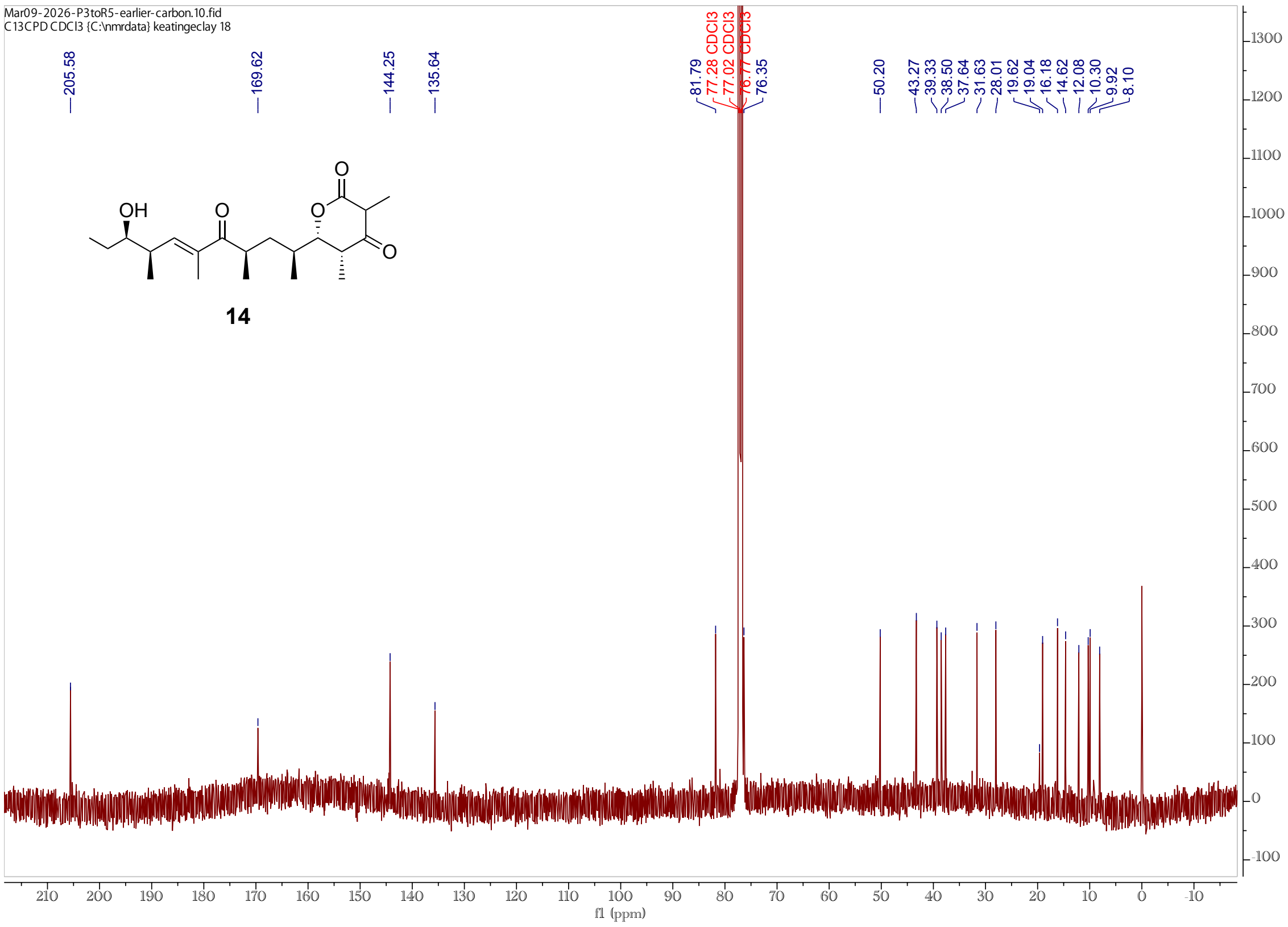

Figure S27. ^{13}C NMR spectrum of the **P1-P2-R5-P4-P5-P6-P7** product **14**.

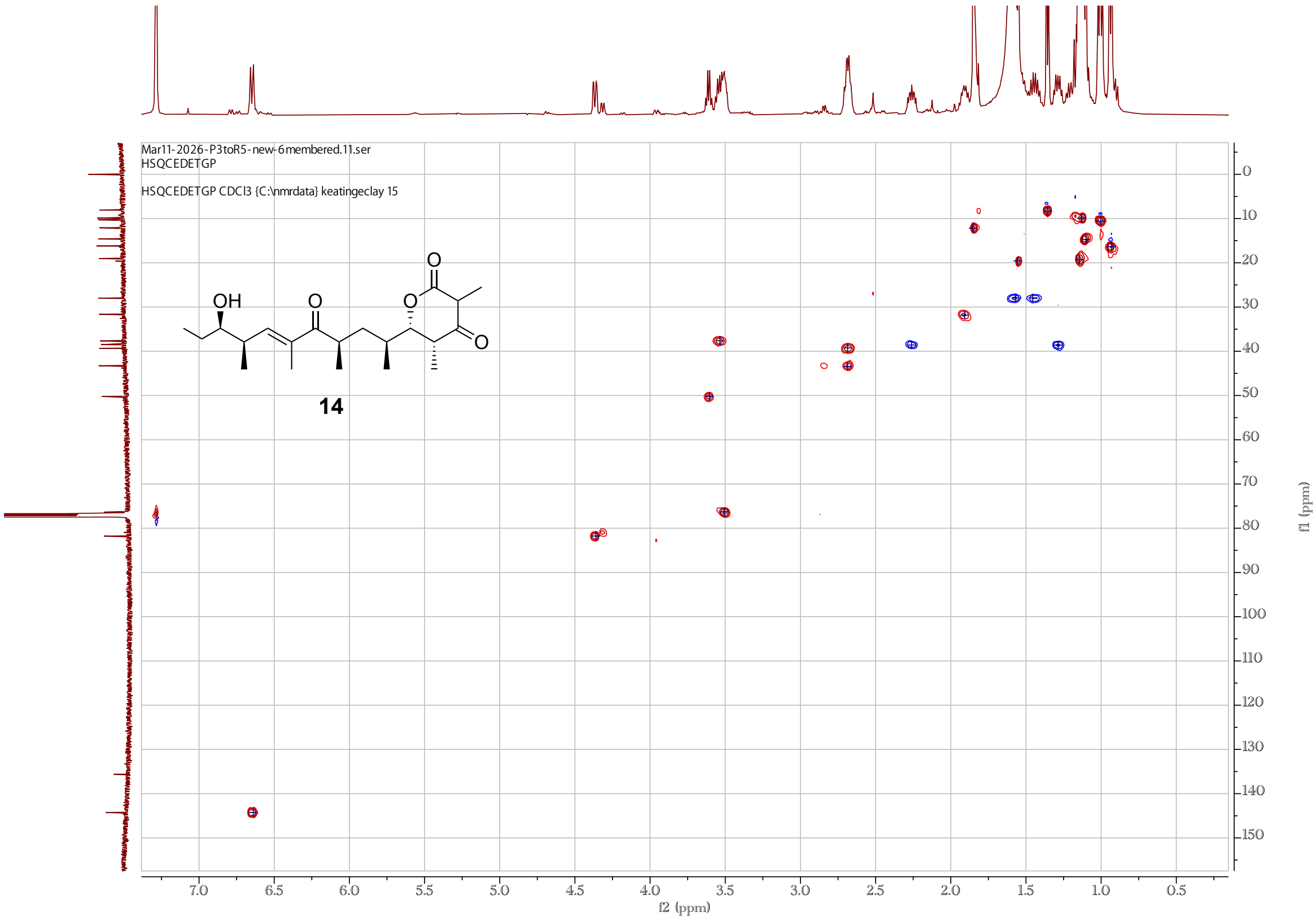

Figure S28. ^{1}H-^{13}C HSQC spectrum of the **P1-P2-R5-P4-P5-P6-P7** product **14**.

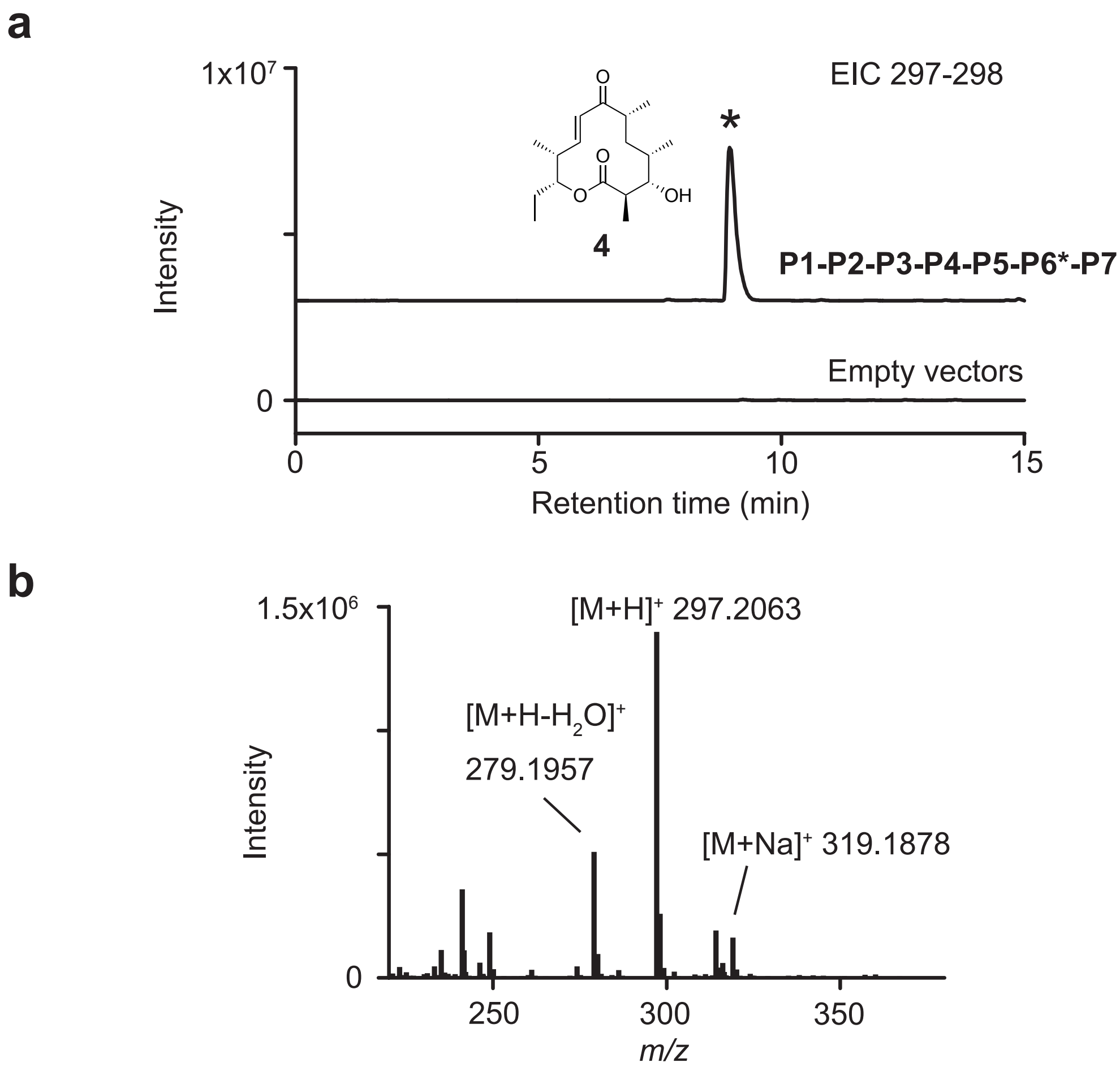

Figure S29. LC/MS analysis of the anticipated **P1-P2-P3-P4-P5-P6*-P7** product, 10-dML (**4**). a) EIC for the $[M+H]^+$ of **4** from the ethyl acetate extract of cells transformed with the PKS expression plasmids or corresponding empty vectors. b) MS spectrum of the main peak (*). Theoretical $[M+H]^+$ *m/z* of **4**, 297.2060; observed *m/z*, 297.2063 (1.0 ppm).

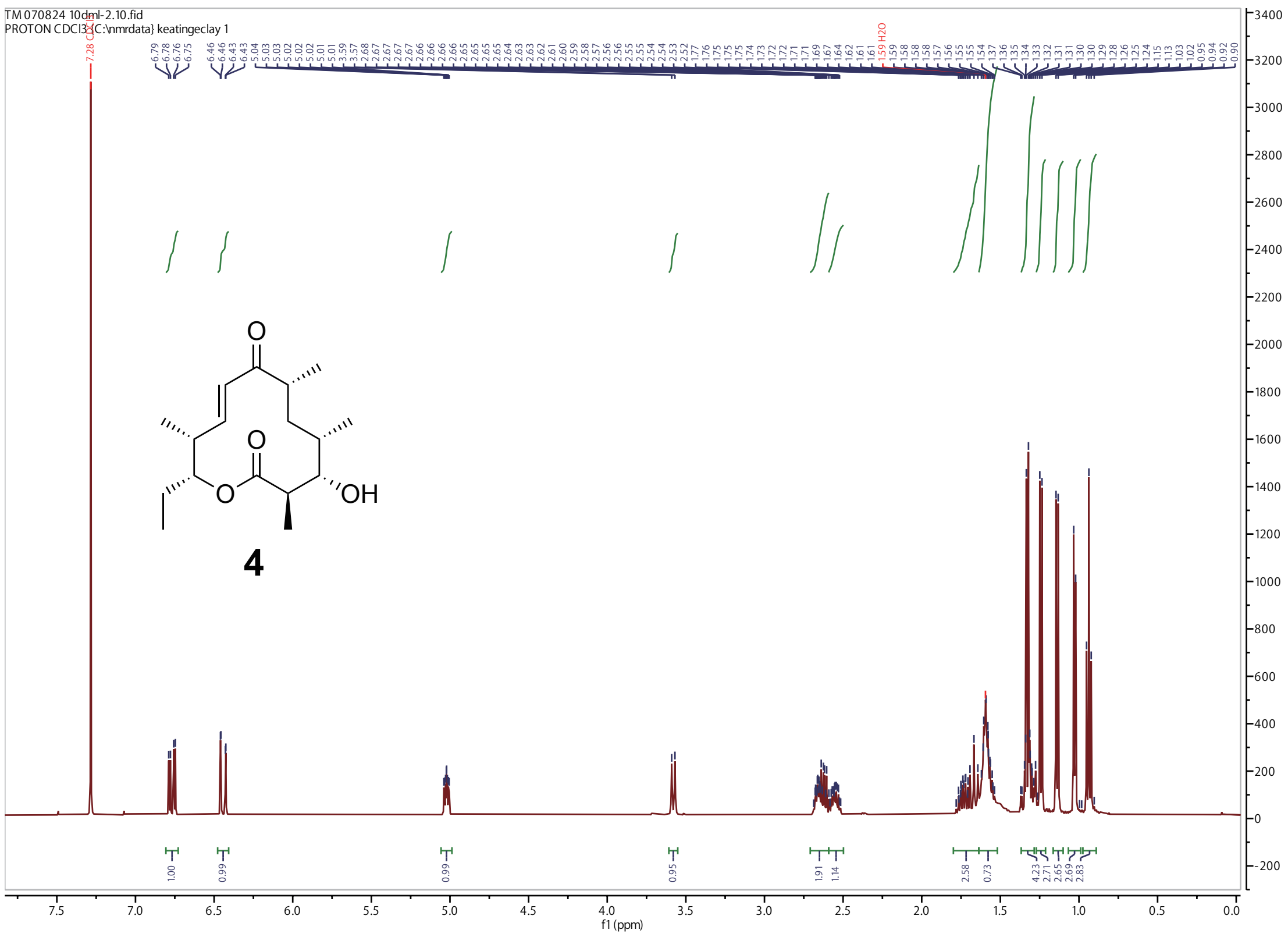

Figure S30. ^{1}H NMR spectrum of the anticipated **P1-P2-P3-P4-P5-P6*-P7** product, **4** (10-dml).

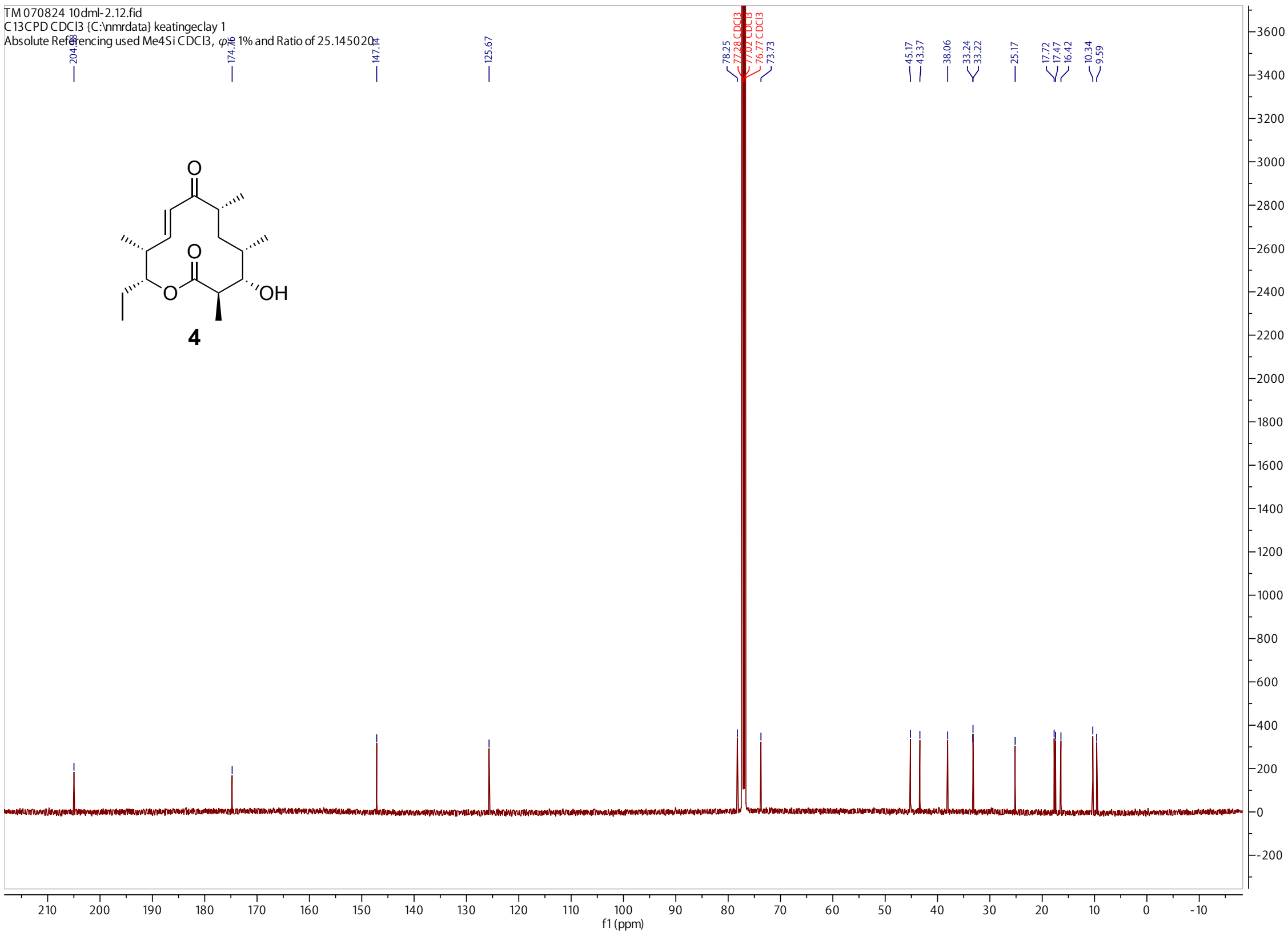

Figure S31. ^{13}C NMR spectrum of the anticipated **P1-P2-P3-P4-P5-P6*-P7** product, **4** (10-dml).

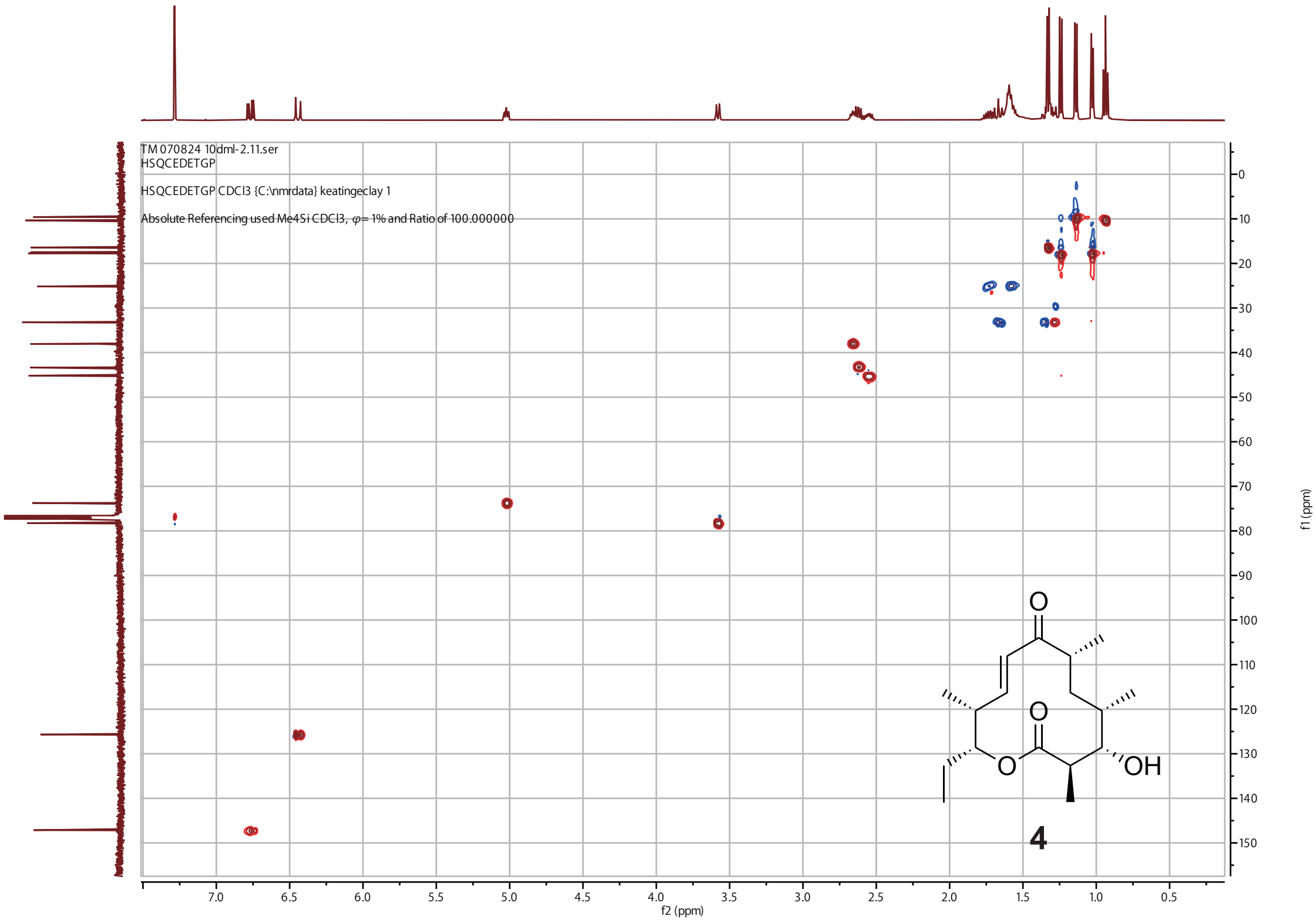

Figure S32. ^{1}H-^{13}C HSQC spectrum of the anticipated **P1**-**P2**-**P3**-**P4**-**P5**-**P6***-**P7** product, **4** (10-dml).

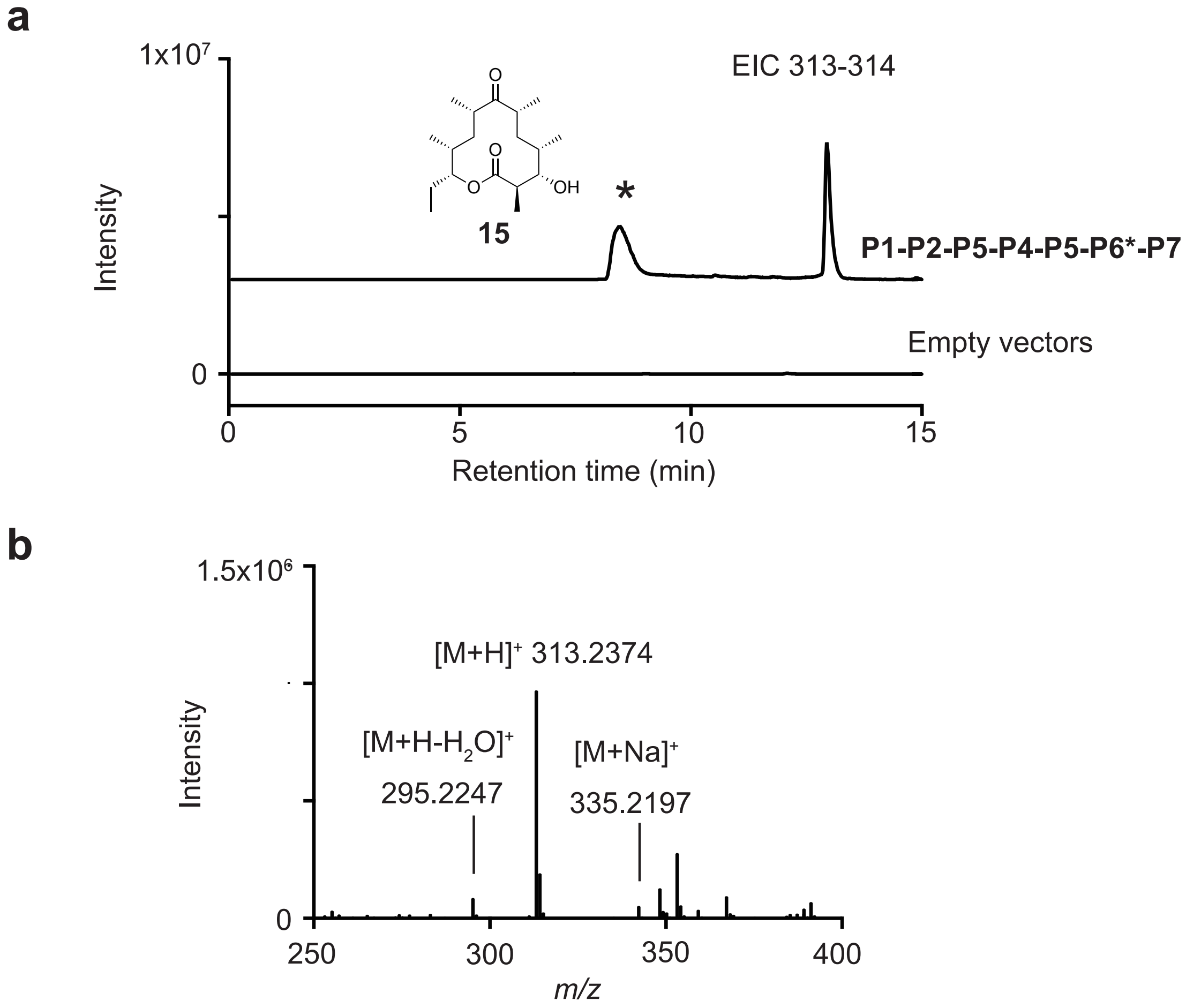

Figure S33. LC/MS analysis of the anticipated **P1-P2-P5-P4-P5-P6*-P7** product, **15.** a) EIC for the $[M+H]^+$ of **15** from the ethyl acetate extract of cells transformed with the PKS expression plasmids or corresponding empty vectors. b) MS spectrum of the main peak (*). Theoretical $[M+H]^+$ *m/z* of **15**, 313.2373; observed *m/z*, 313.2370 (0.31 ppm).

a

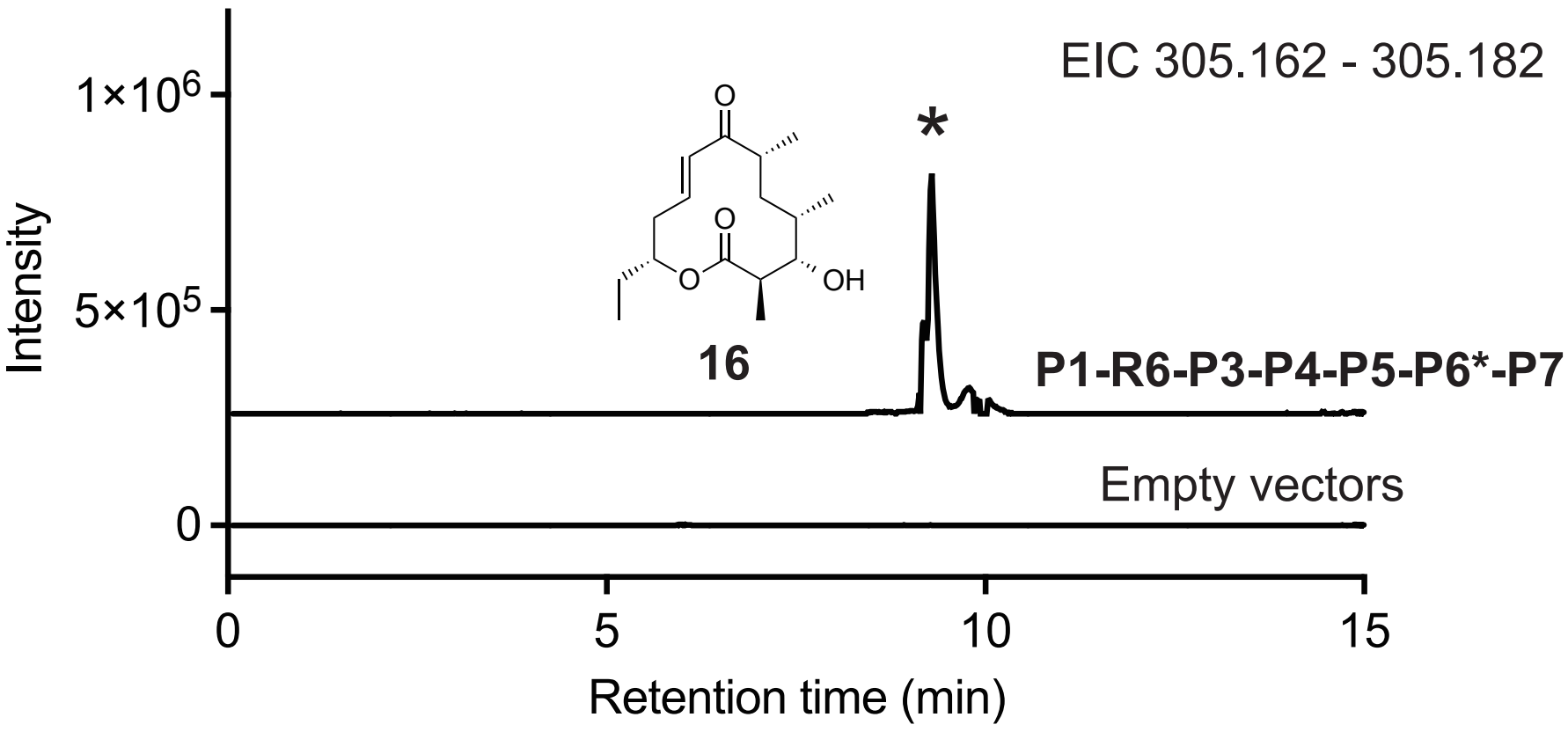

b

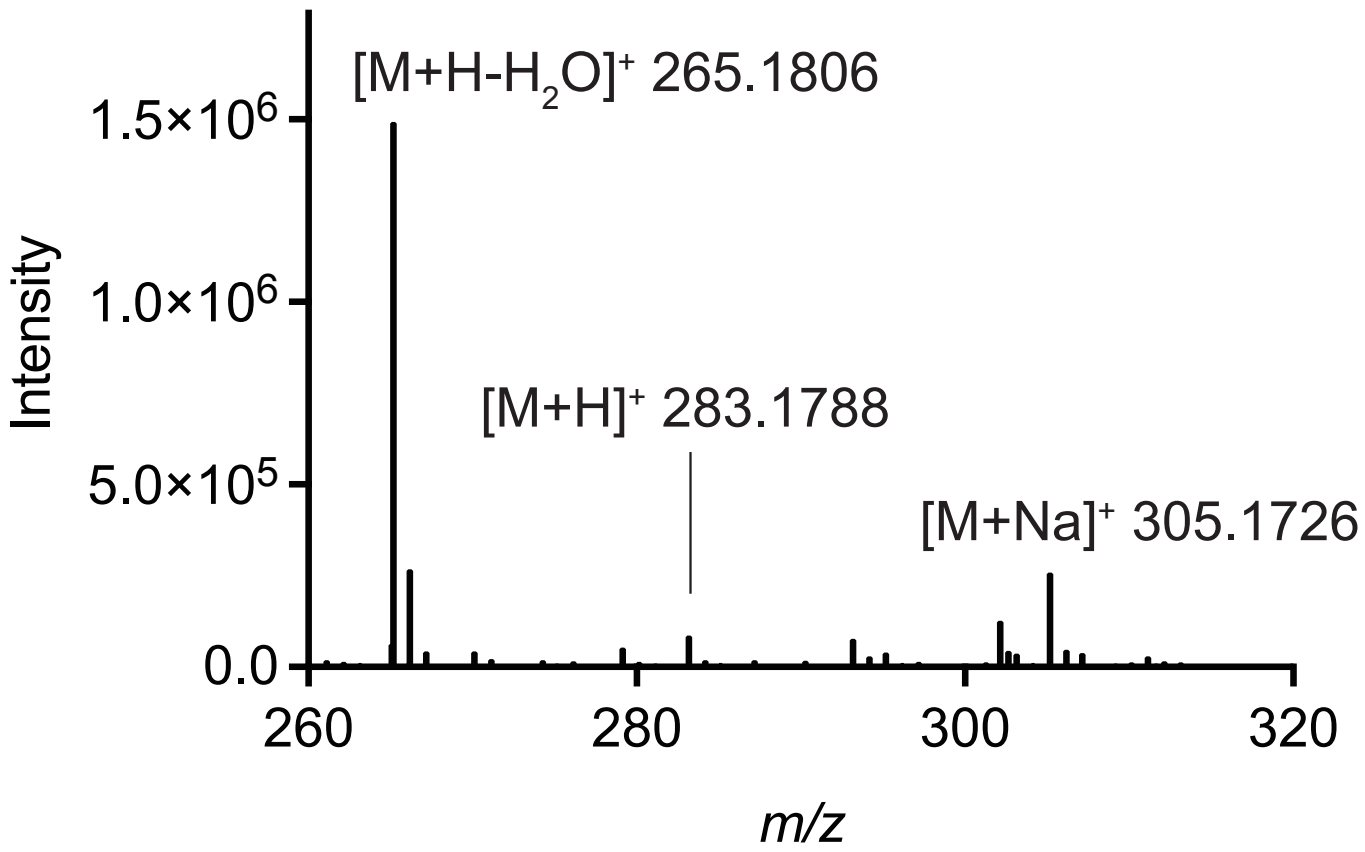

Figure S34. LC/MS analysis of the anticipated **P1-R6-P3-P4-P5-P6*-P7** product, **16**. a) EIC for the $[M+Na]^+$ of **16** from the ethyl acetate extract of cells transformed with the PKS expression plasmids or corresponding empty vectors. b) MS spectrum of the main peak (*). Theoretical $[M+Na]^+$ *m/z* of **16**, 305.1723; observed *m/z*, 305.1726 (0.98 ppm).

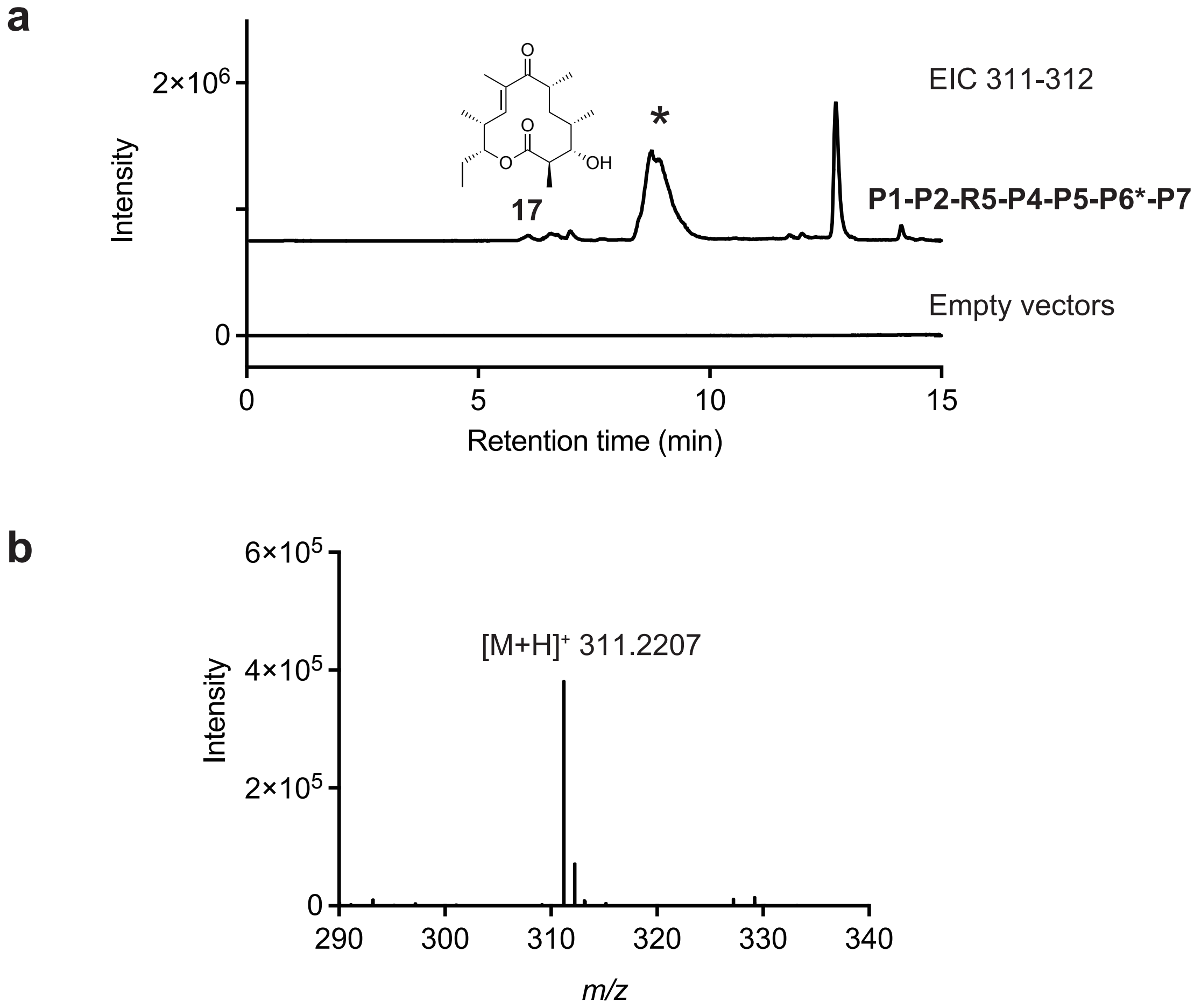

Figure S35. LC/MS analysis of the anticipated **P1-P2-R5-P4-P5-P6*-P7** product, **17**. a) EIC for the $[M+H]^+$ of **17** from the ethyl acetate extract of cells transformed with the PKS expression plasmids or corresponding empty vectors. b) MS spectrum of the main peak (*). Theoretical $[M+H]^+$ *m/z* of **17**, 311.2217; observed *m/z*, 311.2207 (3.2 ppm).

a

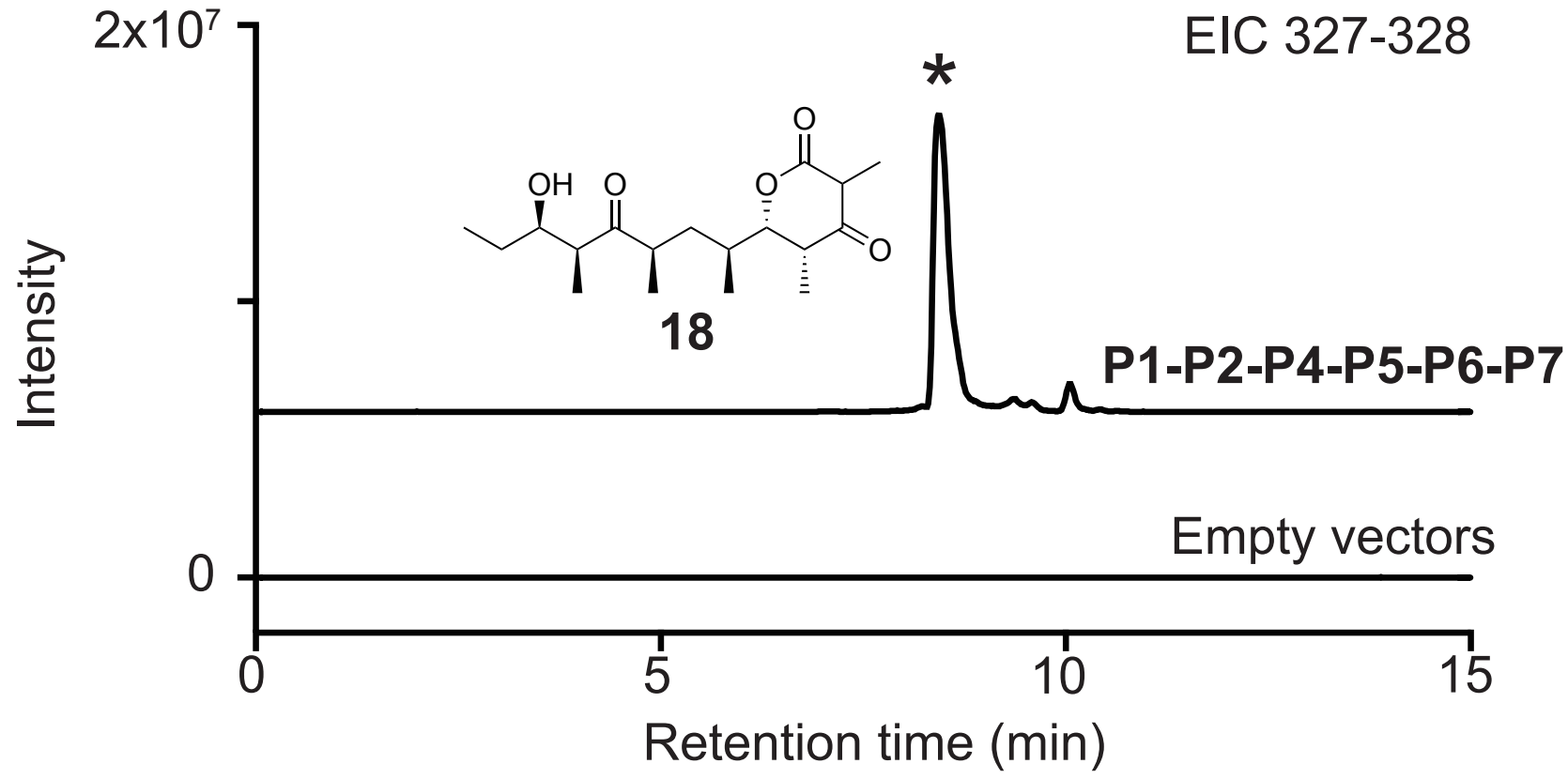

b

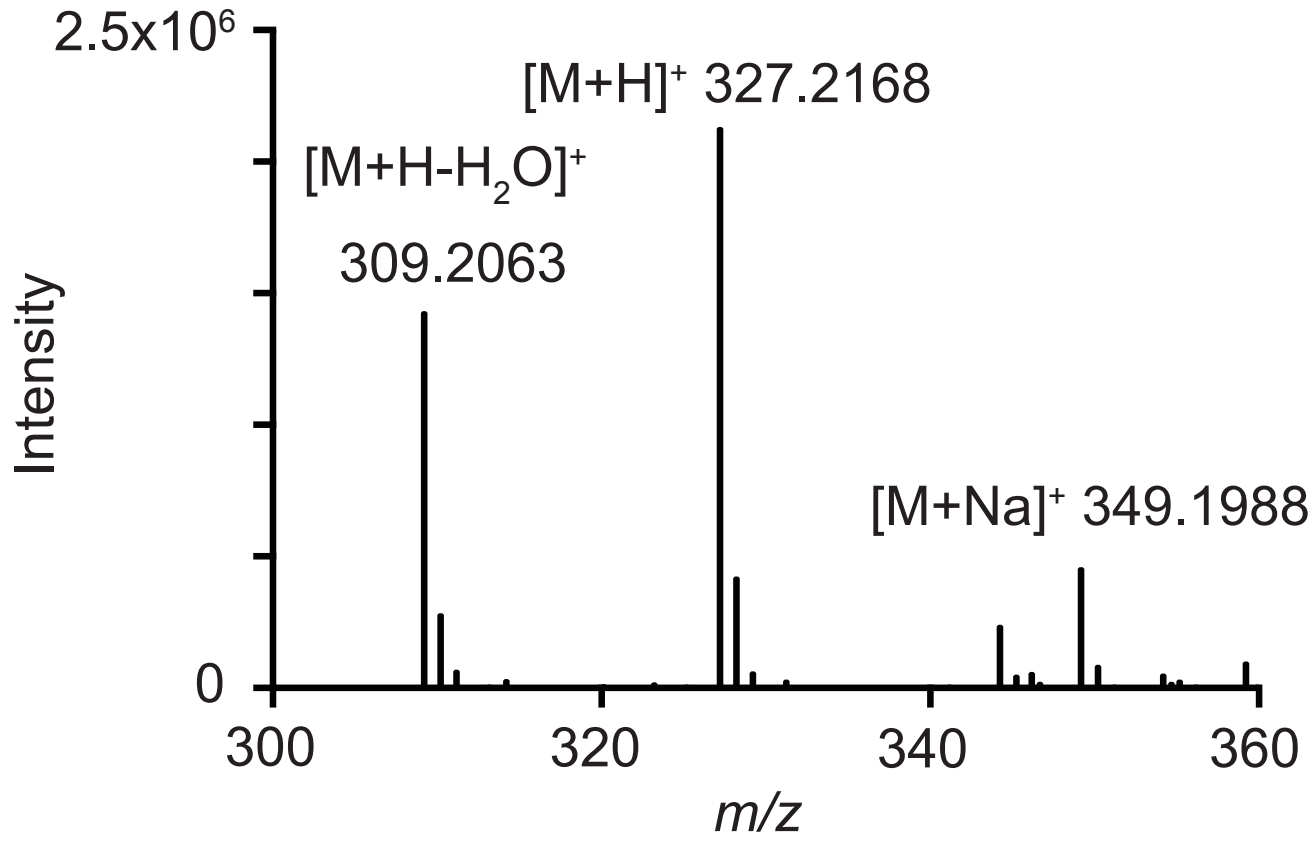

Figure S36. LC/MS analysis of the anticipated **P1**-**P2**-**P4**-**P5**-**P6**-**P7** product, **18**. a) EIC for the $[M+H]^+$ of **18** from the ethyl acetate extract of cells transformed with the PKS expression plasmids or corresponding empty vectors. b) MS spectrum of the main peak (*). Theoretical $[M+H]^+$ *m/z* of **18**, 327.2166; observed *m/z*, 327.2168 (0.61 ppm).

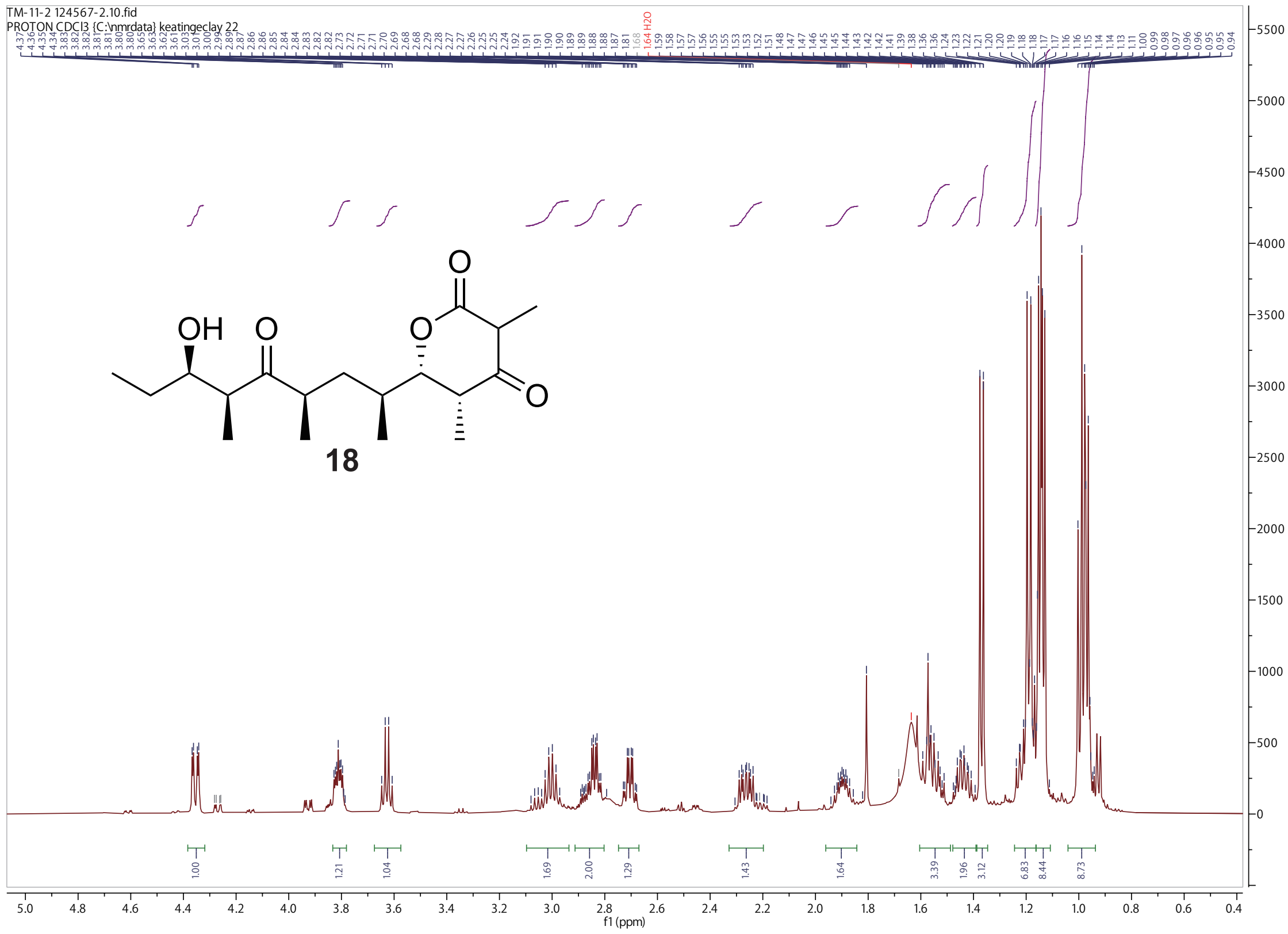

Figure S37. ^{1}H NMR spectrum of the anticipated **P1-P2-P4-P5-P6-P7** product, **18**.

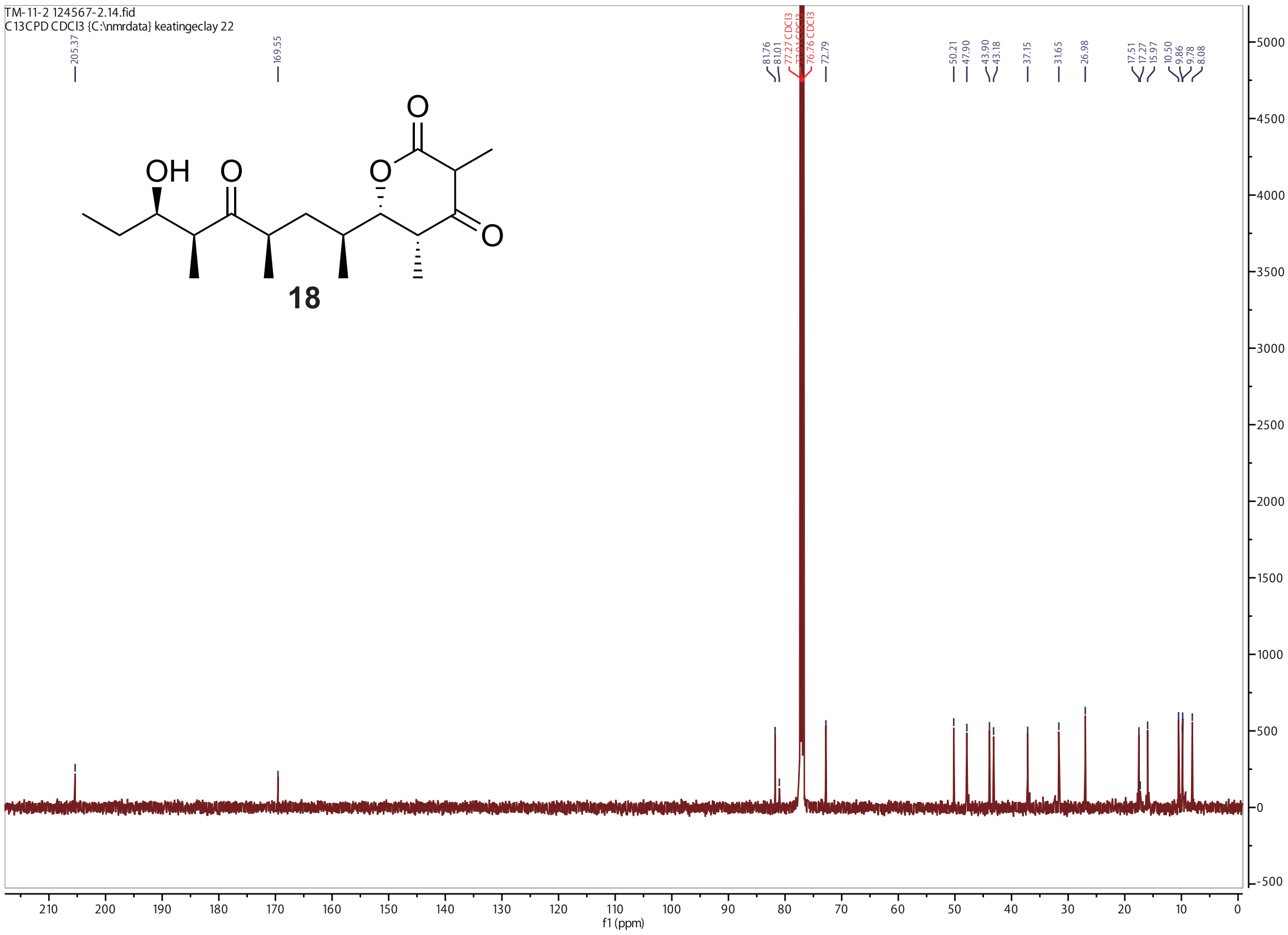

Figure S38. ^{13}C NMR spectrum of the anticipated **P1-P2-P4-P5-P6-P7** product **18**.

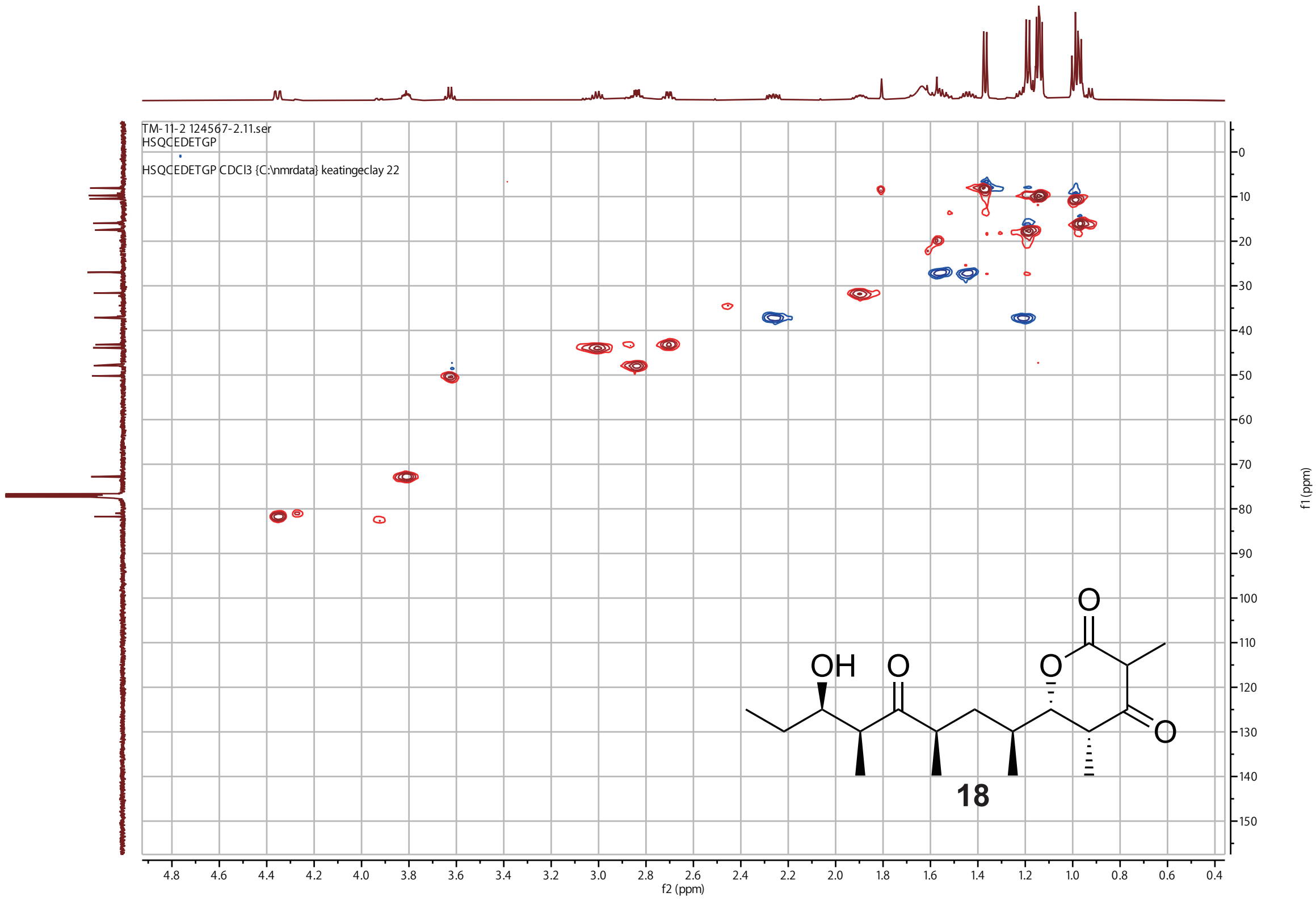

Figure S39. ^{1}H-^{13}C HSQC spectrum of the anticipated **P1-P2-P4-P5-P6-P7** product, **18**.

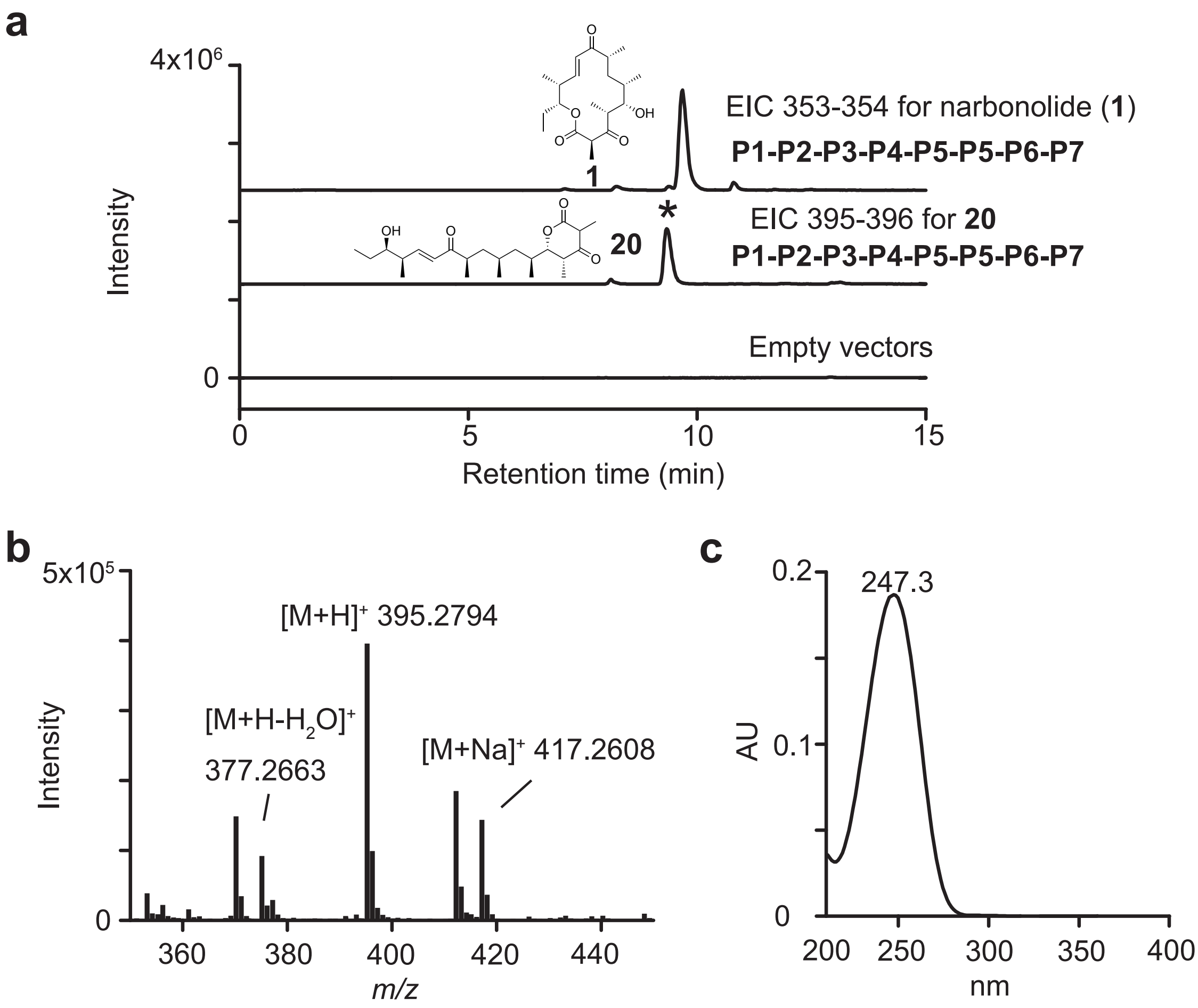

Figure S40. LC/MS analysis of the anticipated **P1-P2-P3-P4-P5-P5-P6-P7** product, **20**. a) EIC for the $[M+H]^+$ of **20** from the ethyl acetate extract of cells transformed with the PKS expression plasmids or corresponding empty vectors. b) MS spectrum of the first peak (*). Theoretical $[M+H]^+$ *m/z* of **20**, 395.2792; observed *m/z*, 395.2794 (0.51 ppm). c) UV spectrum corresponding to the first peak (*) . Since its λ_{max} is equivalent to that of β-keto δ-lactones, the product of **P1-P2-P3-P4-P5-P5-P6-P7** product is **19**, rather than the anticipated octaketide macrolactone.

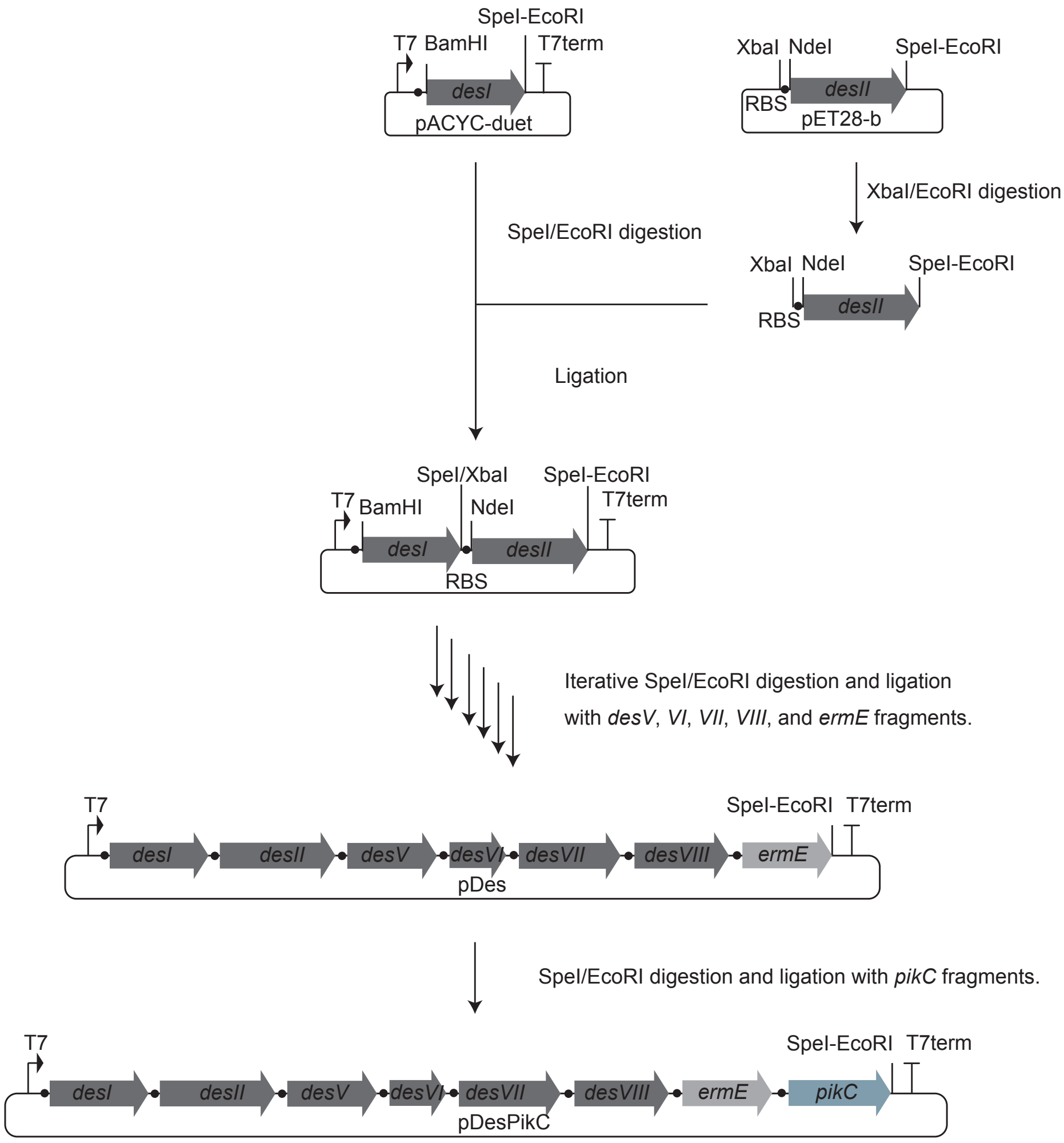

Figure S41. Construction of D-desosamine biosynthesis/transfer pathway. The *desI* gene with an SpeI site after its stop codon was cloned into the BamHI/EcoRI site of pACYC-duet vector, while *desII, V, VI, VII, VIII,* and *ermE* with SpeI sites after their stop codons were cloned into the NdeI/EcoRI site of a pET28b vector. The XbaI/EcoRI fragment containing *desII* was inserted into the SpeI/EcoRI site of the pACYC-*desI*. Since a nonfunctional SpeI/XbaI sites are formed through such ligations, XbaI/EcoRI-digested fragments can be sequentially inserted into the SpeI/EcoRI site of the pACYC plasmid. Iterative SpeI/EcoRI digestion and ligation of *desV*, *VI*, *VII*, *VIII*, and *ermE* fragments yielded pDes. The *pikC* gene was equivalently inserted into the Spe/EcoRI site of pDes to yield pDesPikC. Black dots indicate ribosome binding sites (RBSs).

a

b

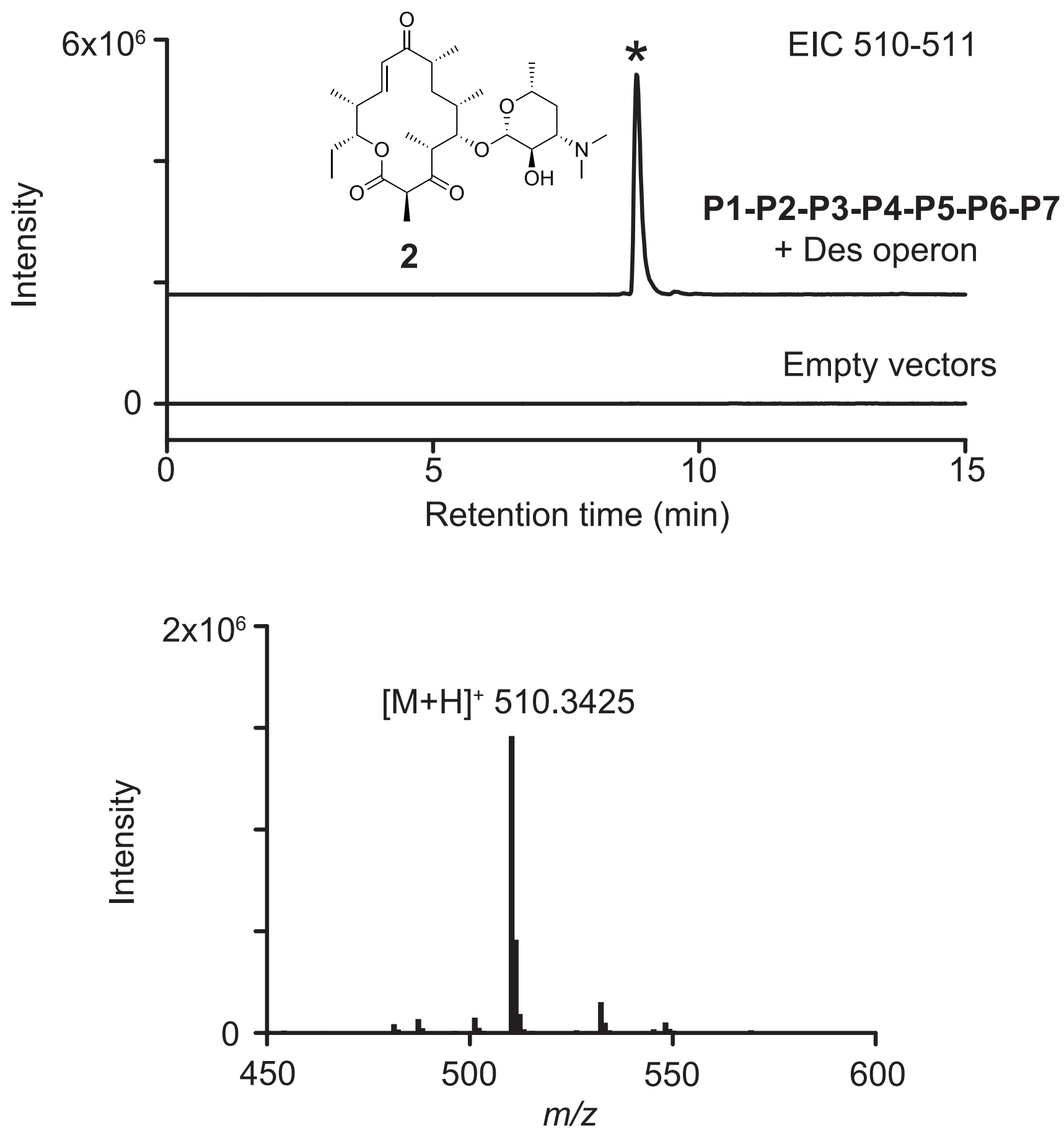

Figure S42. LC/MS analysis of the anticipated **P1-P2-P3-P4-P5-P6-P7**+Des product, narbomycin (**2**). a) EIC for the $[M+H]^+$ of **2** from the ethyl acetate extract of cells transformed with the PKS expression plasmids or corresponding empty vectors. b) MS spectrum of the main peak (*). Theoretical $[M+H]^+$ *m/z* of **2**, 510.3425; observed *m/z*, 510.3425 (0 ppm).

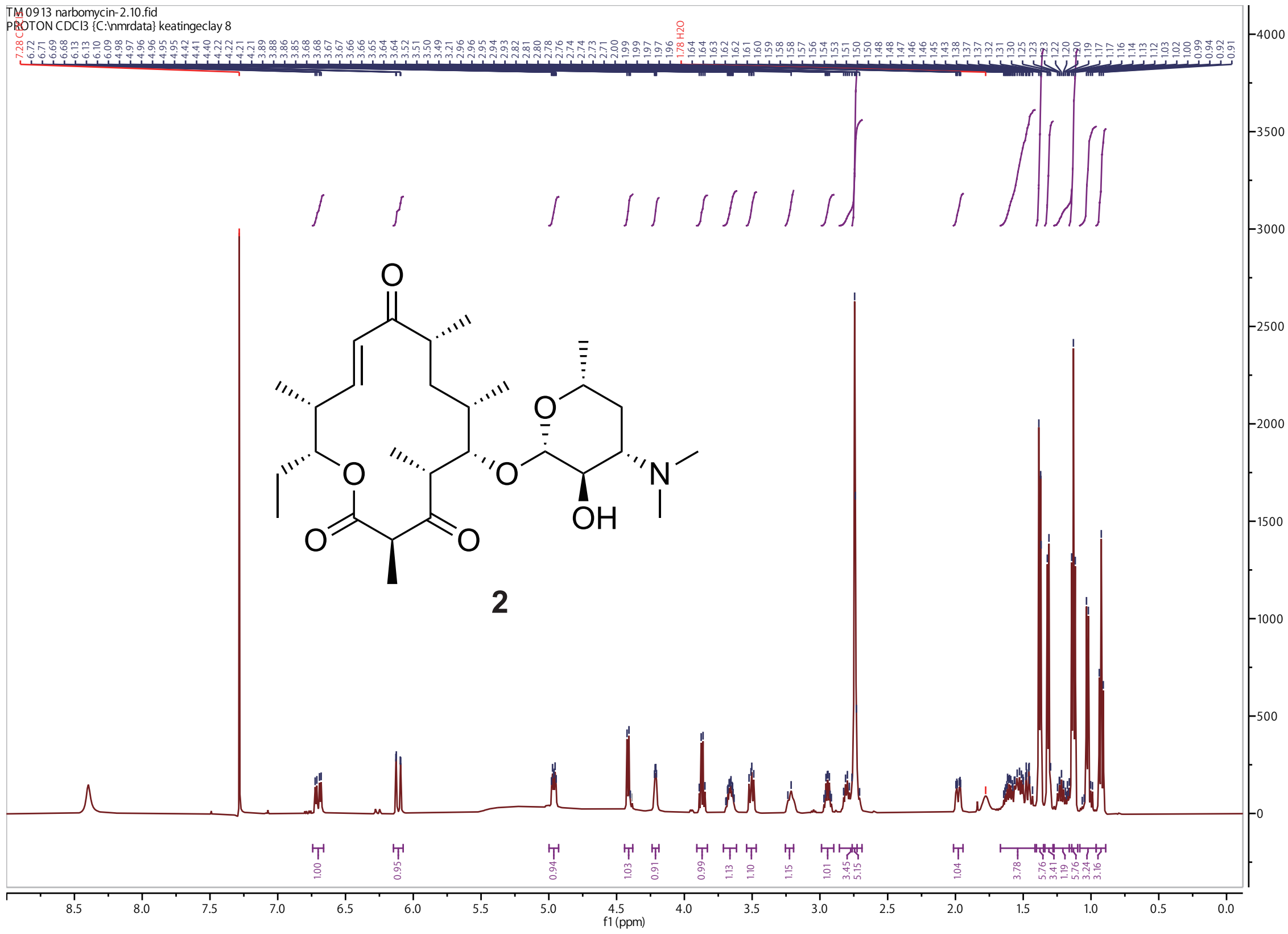

Figure S43. ^{1}H NMR spectrum of the anticipated **P1**-**P2**-**P3**-**P4**-**P5**-**P6**-**P7**+Des product, narbomycin (**2**).

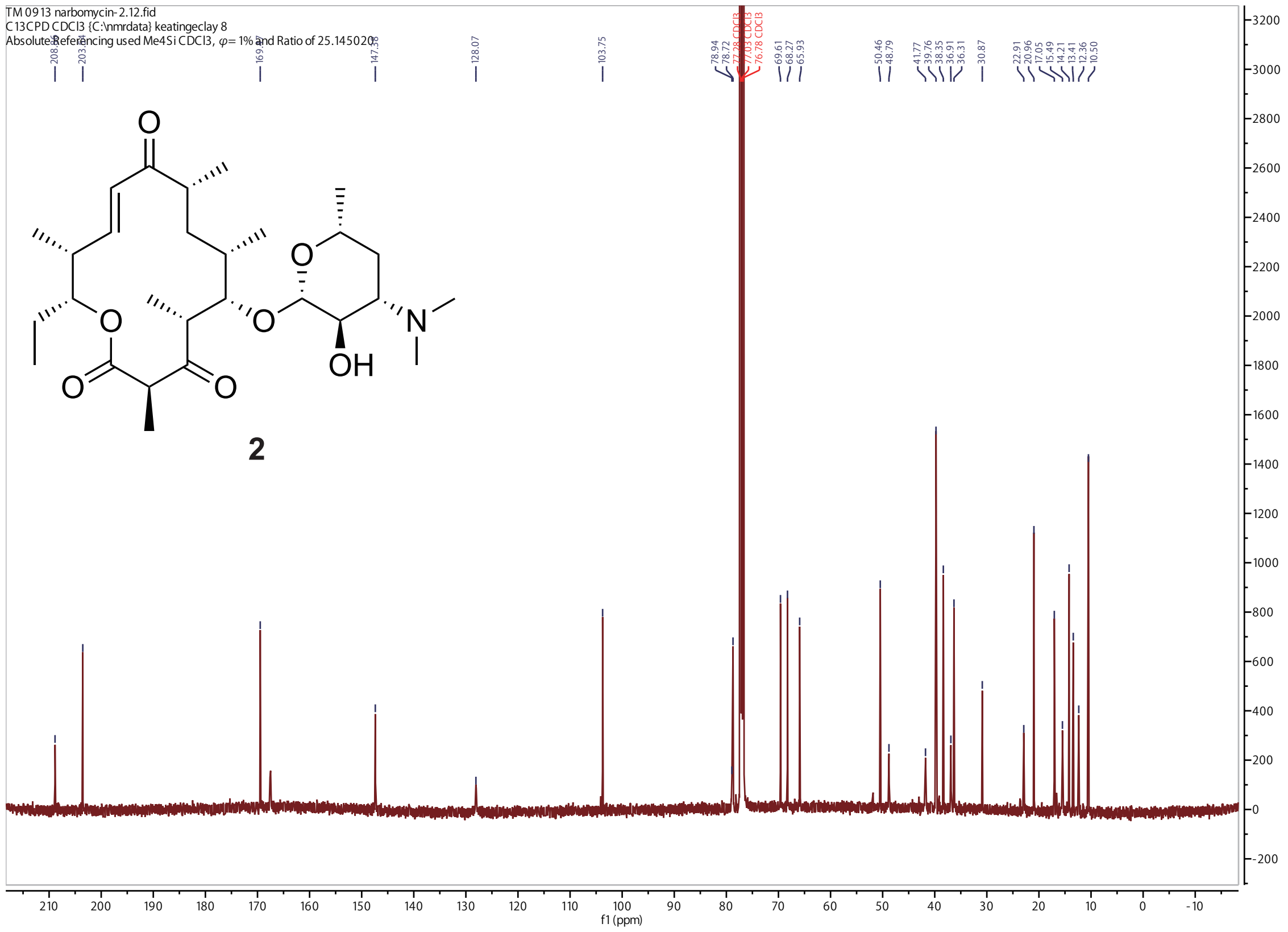

Figure S44. ^{13}C NMR spectrum of the anticipated **P1-P2-P3-P4-P5-P6-P7**+Des product, narbomycin (**2**).

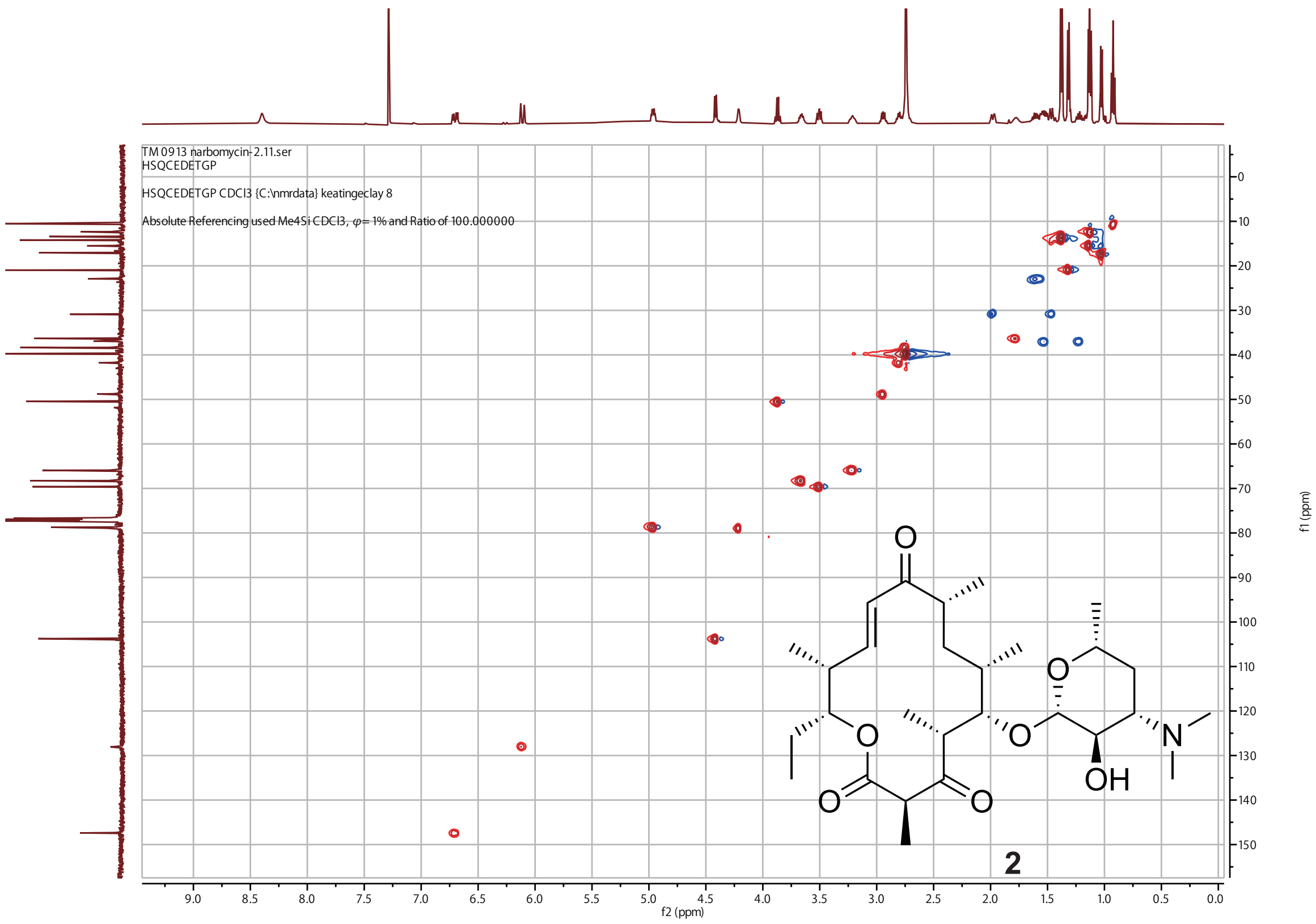

Figure S45. ^{1}H-^{13}C HSQC spectrum of the anticipated **P1-P2-P3-P4-P5-P6-P7**+Des product, narbomycin (**2**).

a

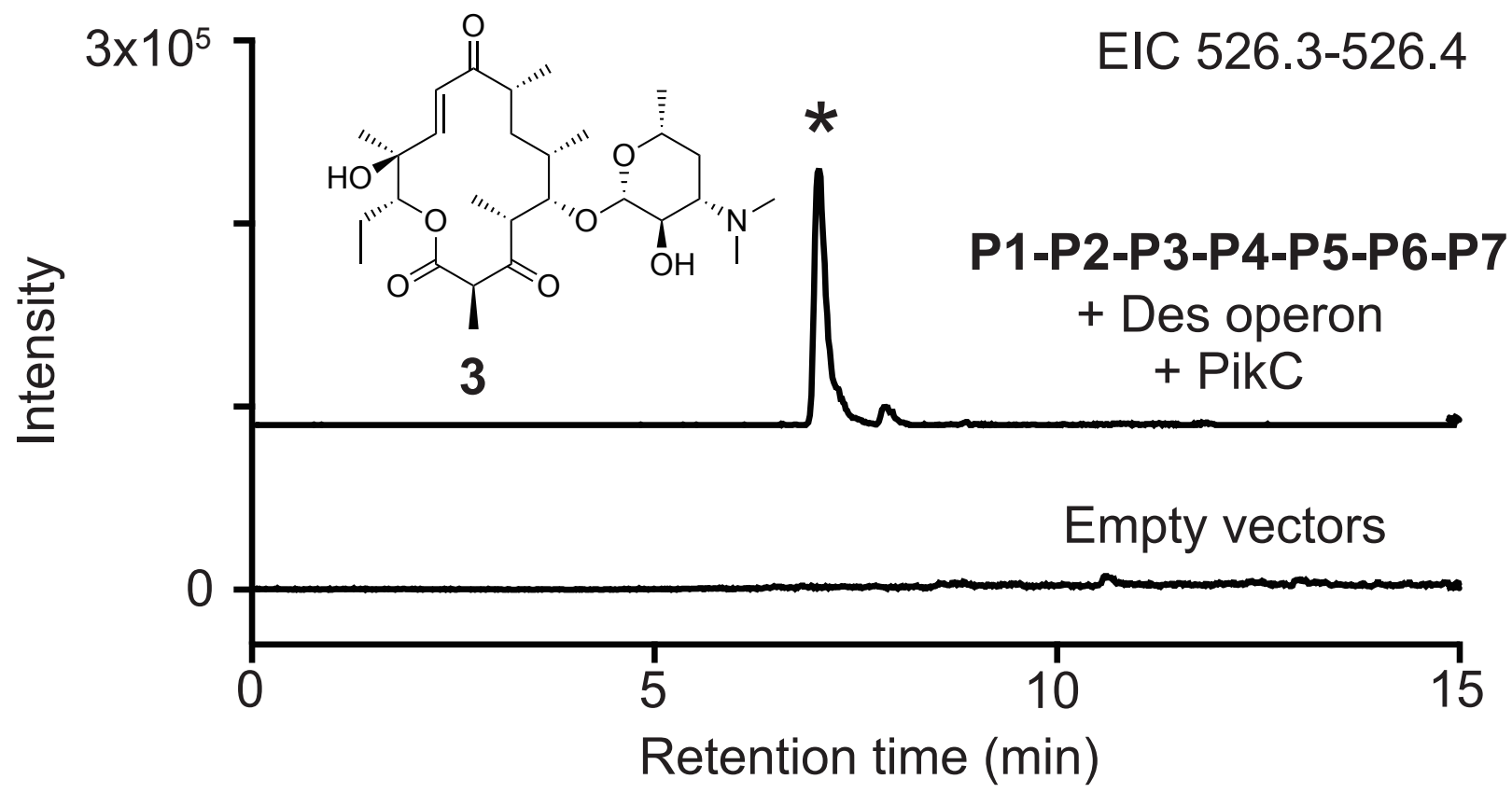

b

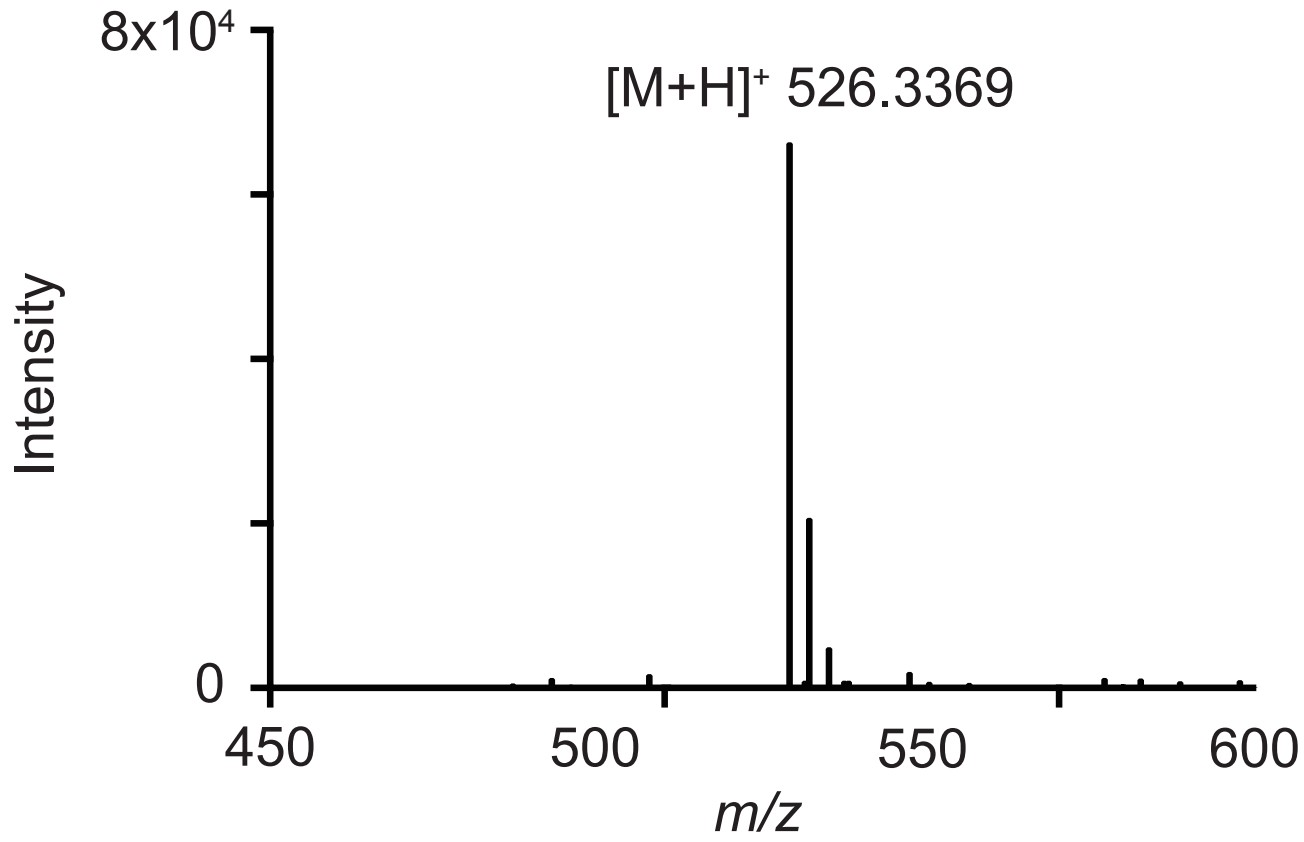

Figure S46. LC/MS analysis of the anticipated **P1-P2-P3-P4-P5-P6-P7**+Des+PikC product, pikromycin (**3**). a) EIC for the $[M+H]^+$ of **3** from the ethyl acetate extract of cells transformed with the PKS expression plasmids or corresponding empty vectors. b) MS spectrum of the main peak (*). Theoretical $[M+H]^+$ *m/z* of **3**, 526.3374; observed *m/z*, 526.3369 (-0.94 ppm).

a

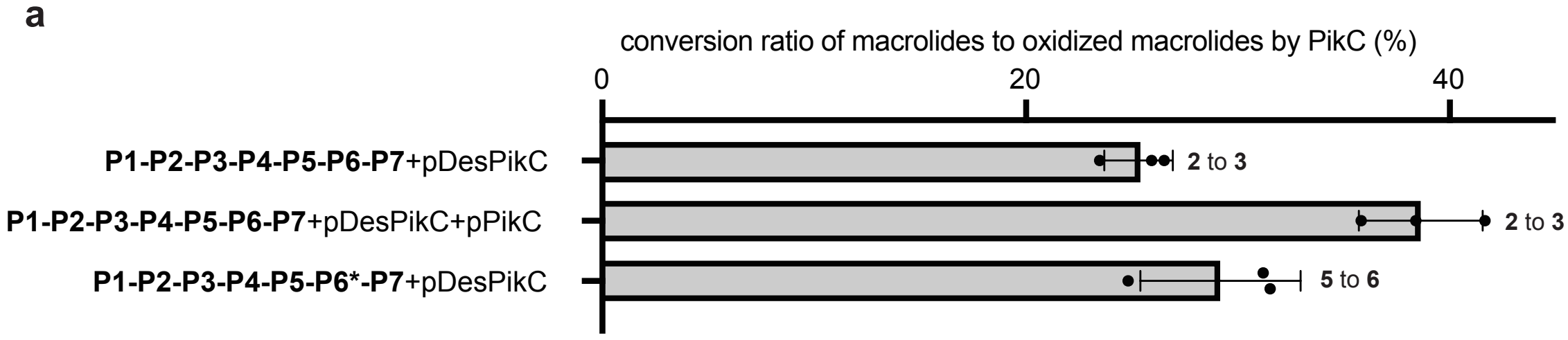

b

	PikC conversion ratio (%)	macrolide (mgL^{-1})	oxidized macrolide (mgL^{-1})
**P1-P2-P3-P4-P5-P6-P7**+pDesPikC	25 ± 2	1.3 ± 0.1	0.45 ± 0.02
**P1-P2-P3-P4-P5-P6-P7**+pDesPikC+pPikC	38 ± 3	0.90 ± 0.11	0.58 ± 0.02
**P1-P2-P3-P4-P5-P6*-P7**+pDesPikC	29 ± 4	0.66 ± 0.09	0.27 ± 0.04

Figure S47. PikC oxidation efficiency**.** PKS expression plasmids were co-transformed with pDesPikC or pDesPikC and pPikC into *E. coli* K207-3. Percent conversion was estimated using the EIC peak areas of the unoxidized and oxidized compounds. EIC ranges (*m*/*z*): 510-511 for narbomycin (**1**), 526-527 for pikromycin (**3**). Data are mean ± s.d., n = 3 independent experiments (Supplementary Data 7).

a

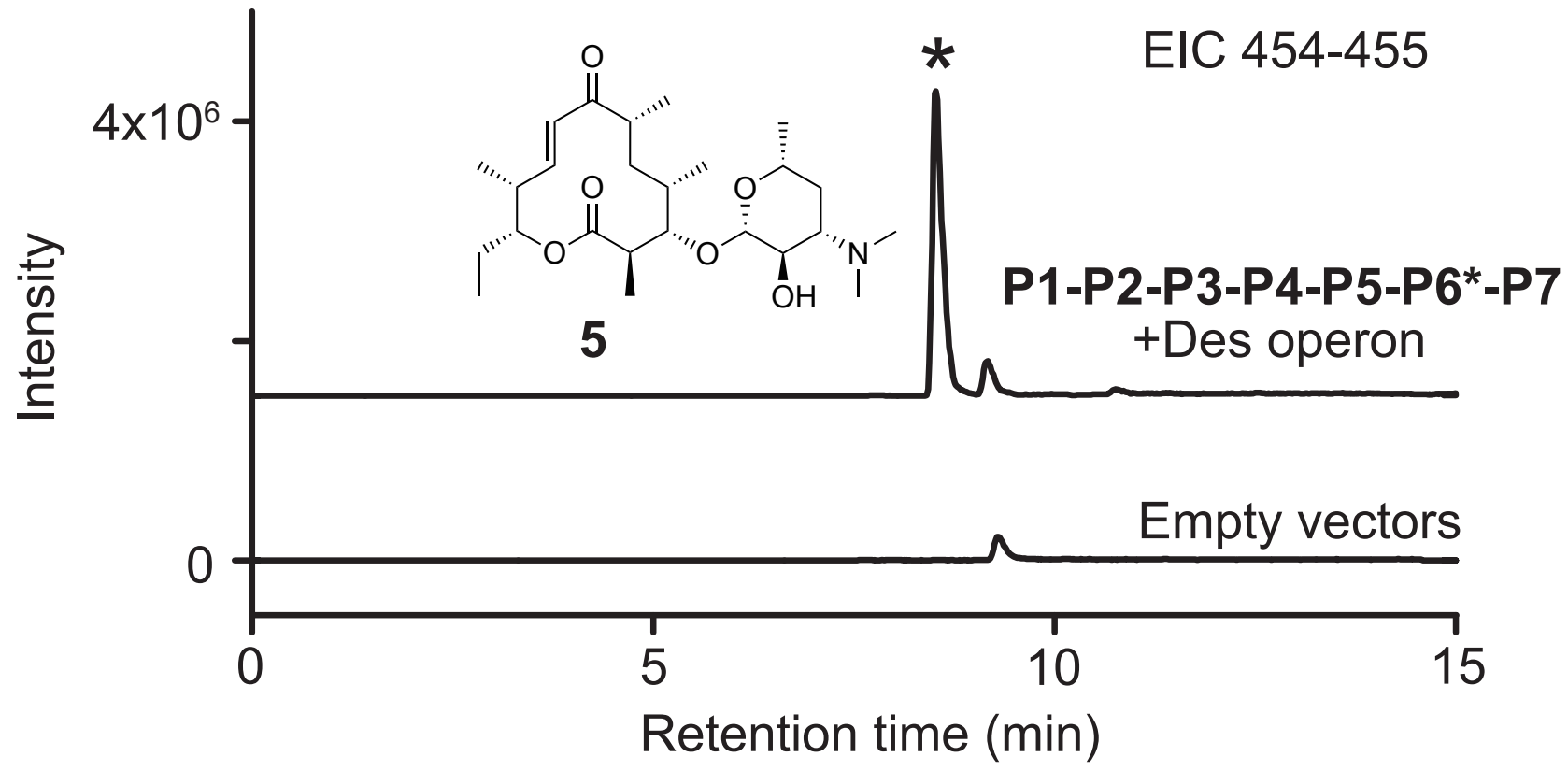

b

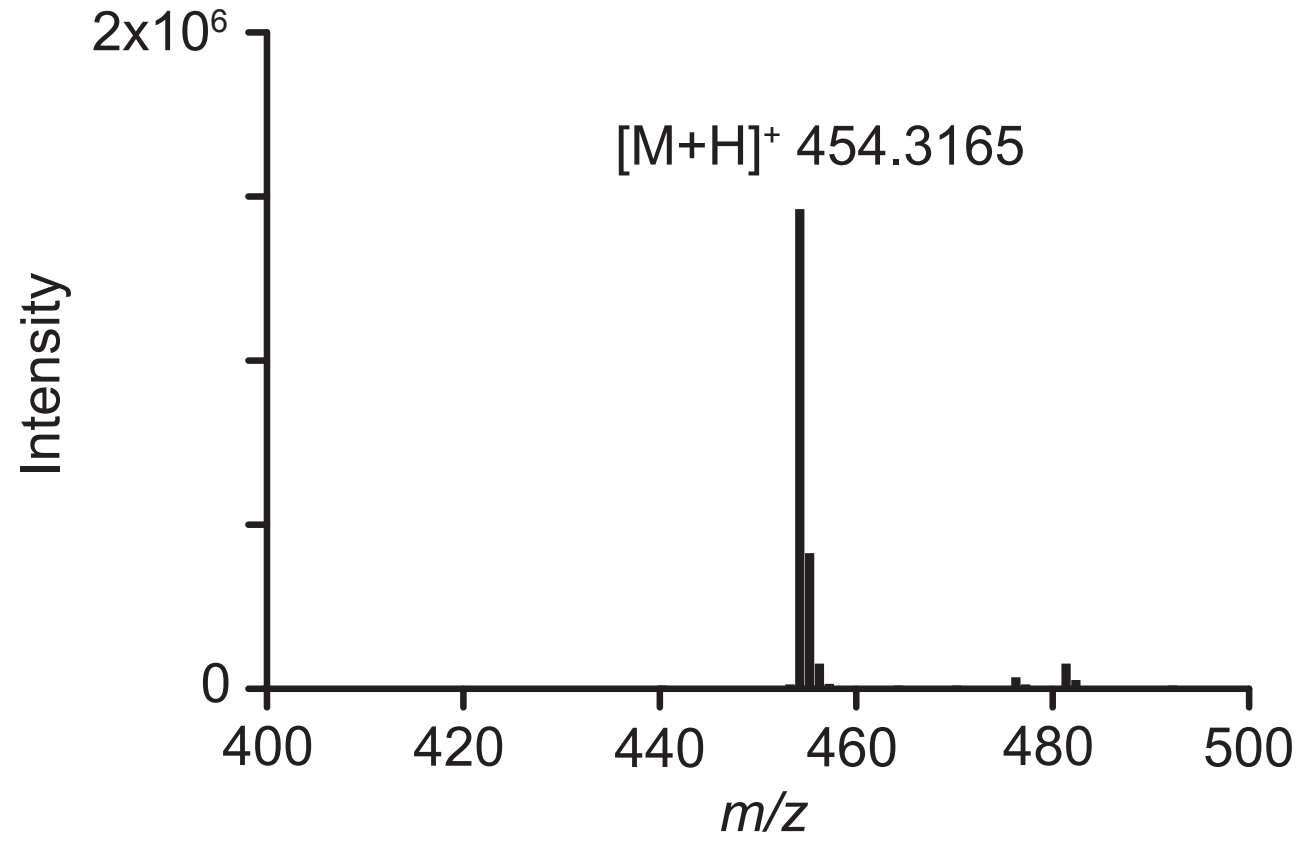

Figure S48. LC/MS analysis of the anticipated **P1**-**P2**-**P3**-**P4**-**P5**-**P6***-**P7**+Des product, YC-17 (**5**). a) EIC for the $[M+H]^+$ of **5** from the ethyl acetate extract of cells transformed with the PKS expression plasmids or corresponding empty vectors. b) MS spectrum of the main peak (*). Theoretical $[M+H]^+$ *m/z* of **5**, 454.3163; observed *m/z*, 454.3165 (0.41 ppm).

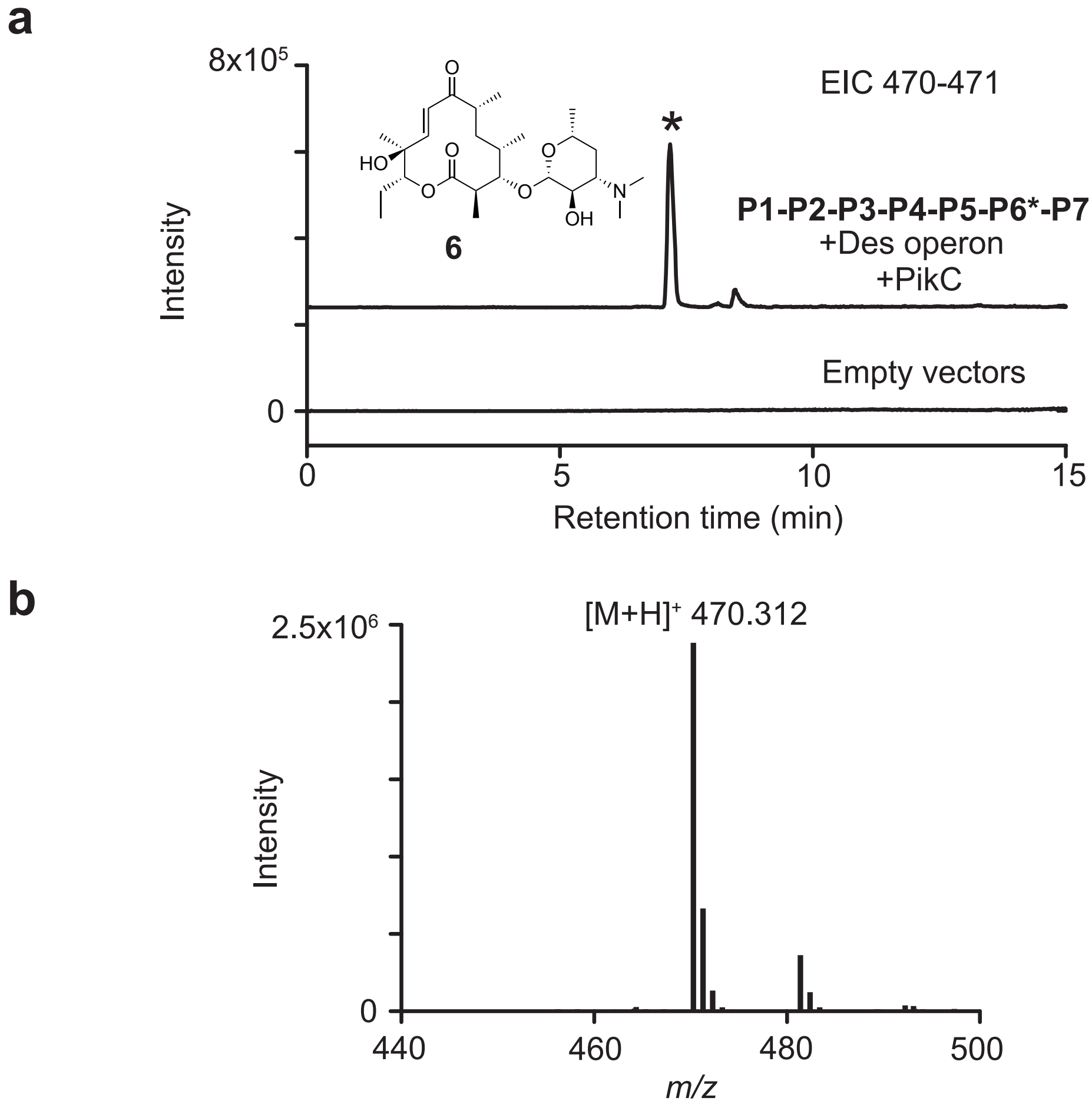

Figure S49. LC/MS analysis of the anticipated **P1-P2-P3-P4-P5-P6*-P7**+Des+PikC product, methymycin (**6**). a) EIC for the $[M+H]^+$ of **6** from the ethyl acetate extract of cells transformed with the PKS expression plasmids or corresponding empty vectors. b) MS spectrum of the main peak (*). Theoretical $[M+H]^+$ *m/z* of **6**, 470.3112; observed *m/z*, 470.3120 (1.6 ppm).

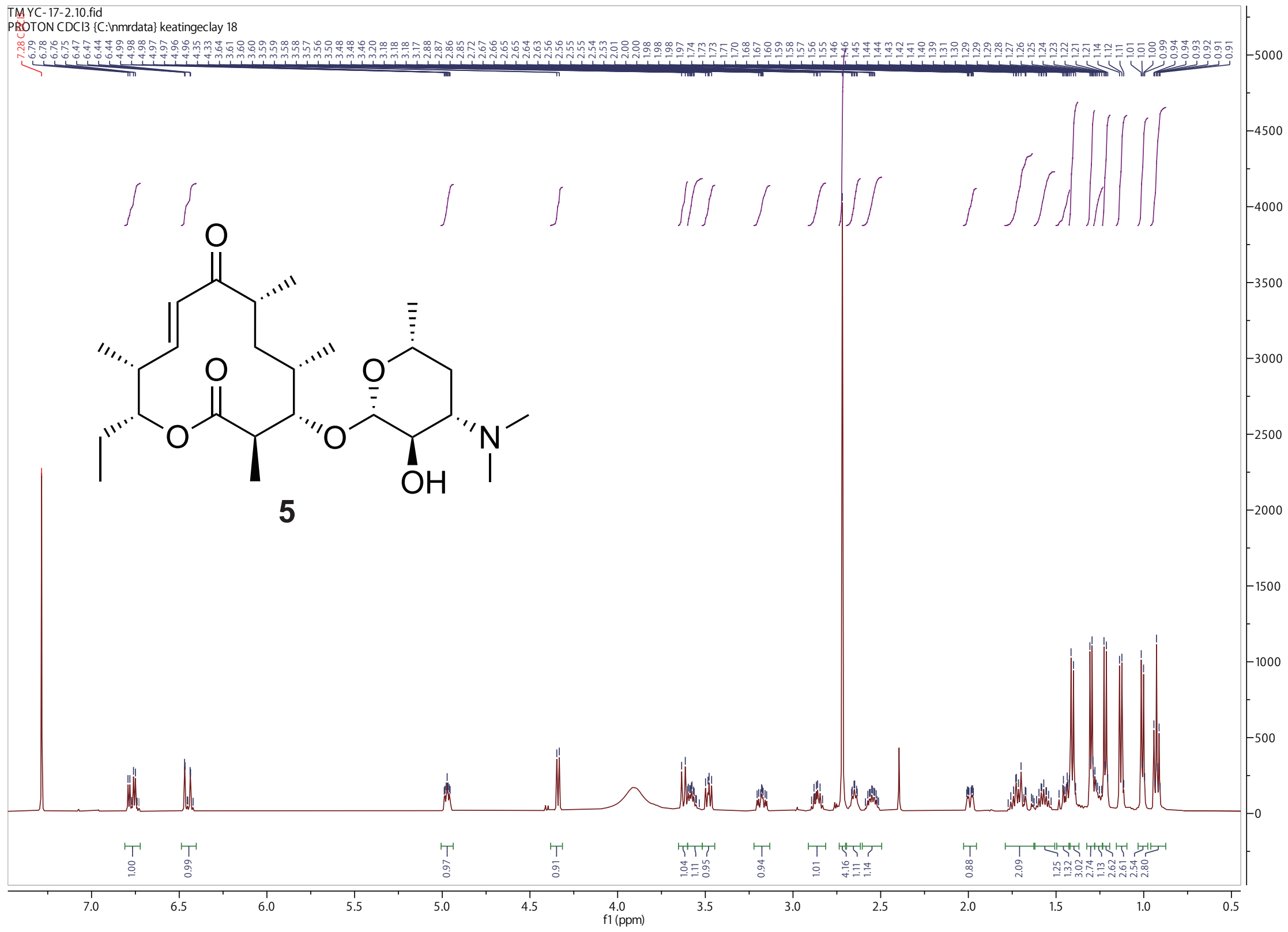

Figure S50. ^{1}H NMR spectrum of the anticipated **P1-P2-P3-P4-P5-P6*-P7**+Des product, YC-17 (**5**).

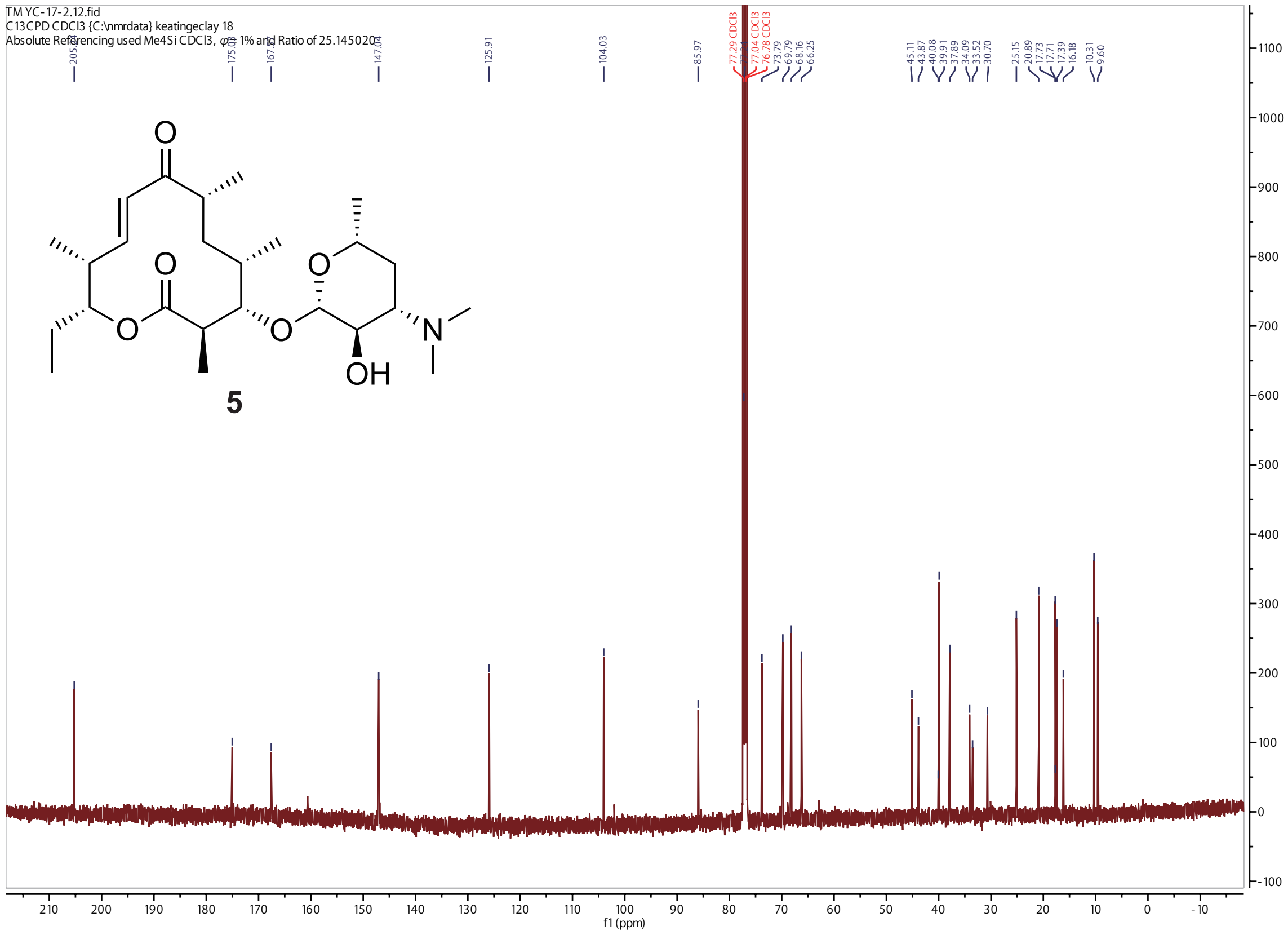

Figure S51. ^{13}C NMR spectrum of the anticipated **P1**-**P2**-**P3**-**P4**-**P5**-**P6***-**P7**+Des product, YC-17 (**5**).

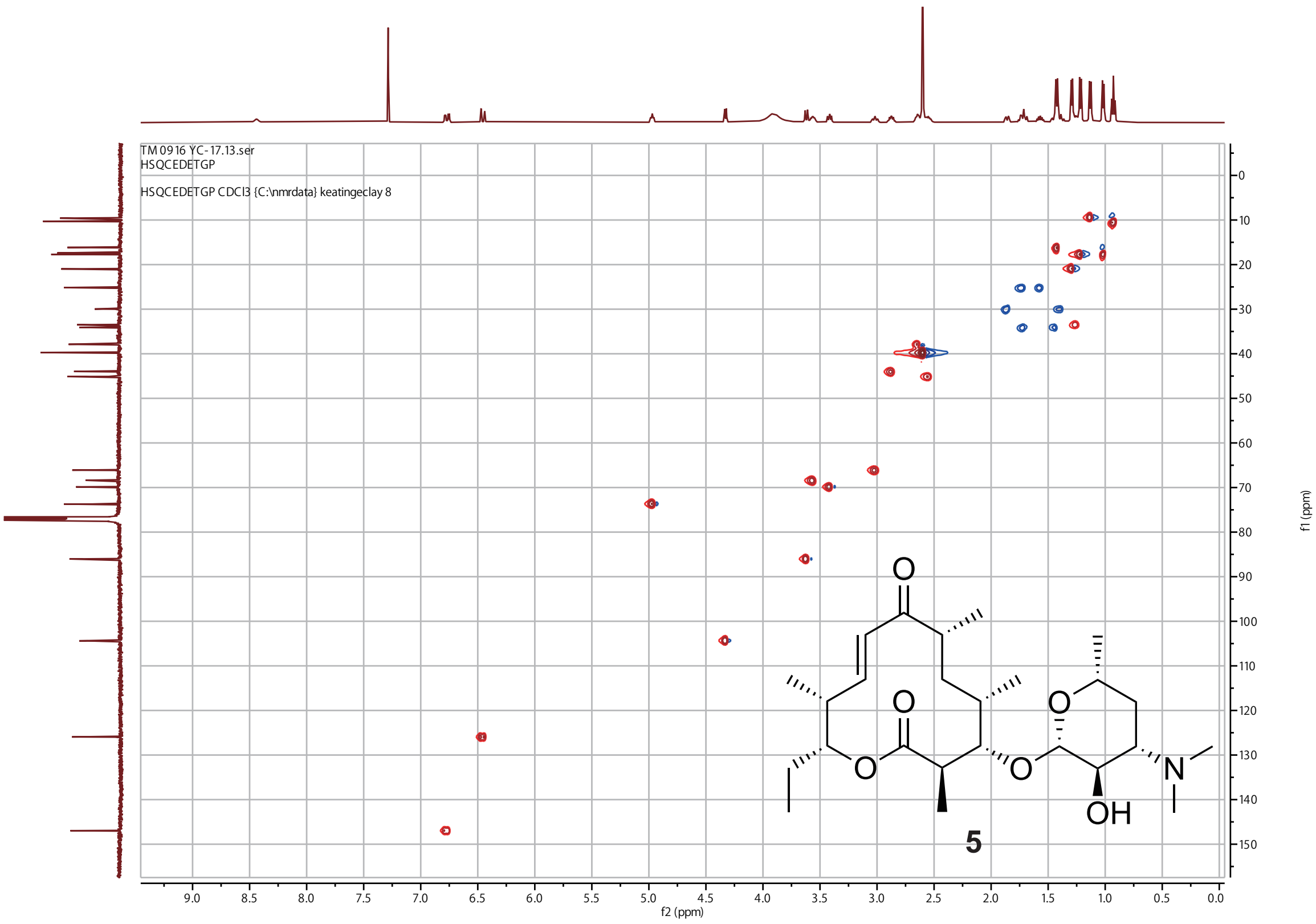

Figure S52. ^{1}H-^{13}C HSQC spectrum of the anticipated **P1-P2-P3-P4-P5-P6*-P7**+Des product, YC-17 (**5**).

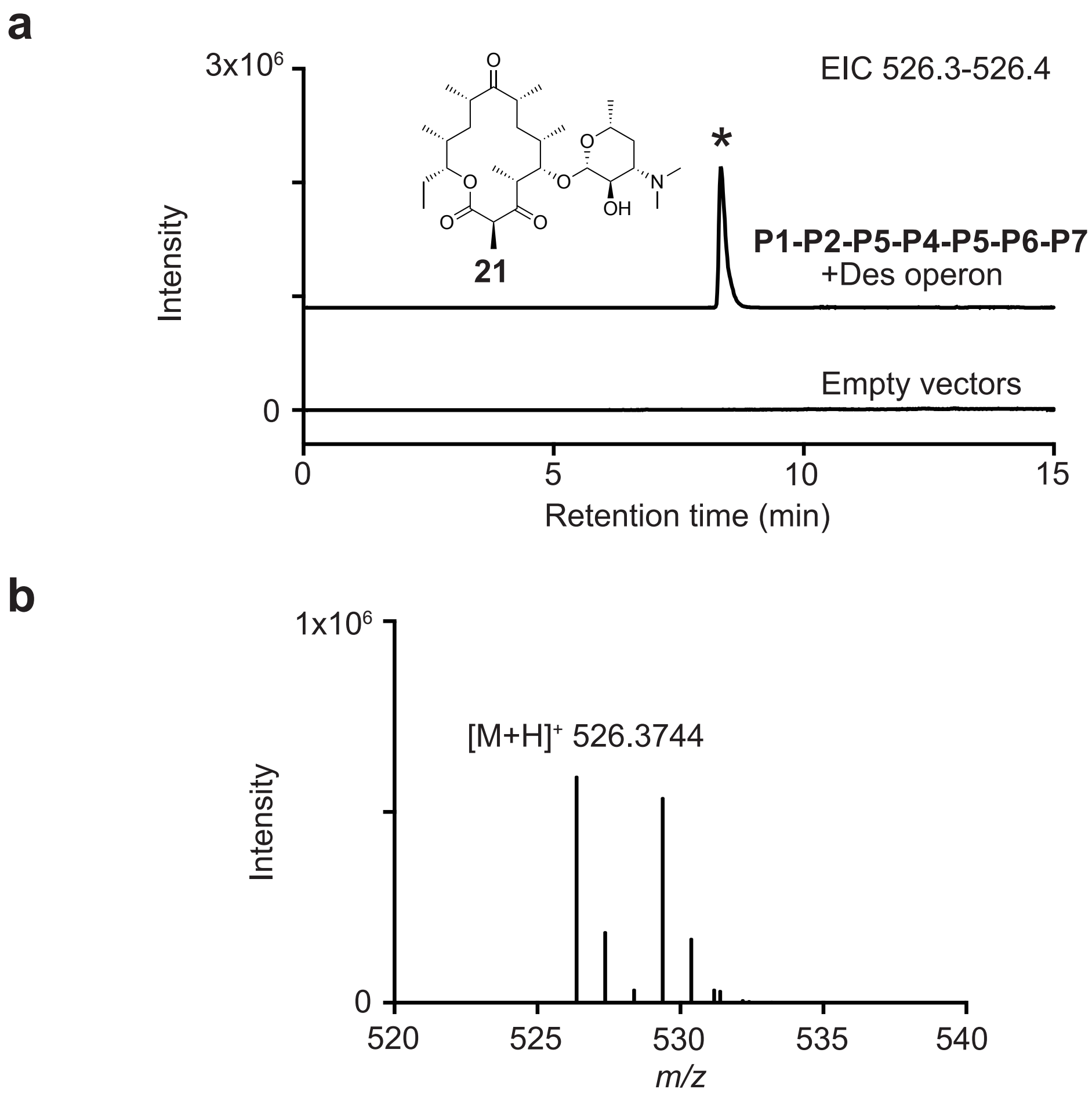

Figure S53. LC/MS analysis of the anticipated **P1-P2-P5-P4-P5-P6-P7**+Des product, **21**. a) EIC for the $[M+H]^+$ of **21** from the ethyl acetate extract of cells transformed with the PKS expression plasmids or corresponding empty vectors. b) MS spectrum of the main peak (*). Theoretical $[M+H]^+$ *m/z* of **21**, 526.3738; observed *m/z*, 526.3744 (1.1 ppm).

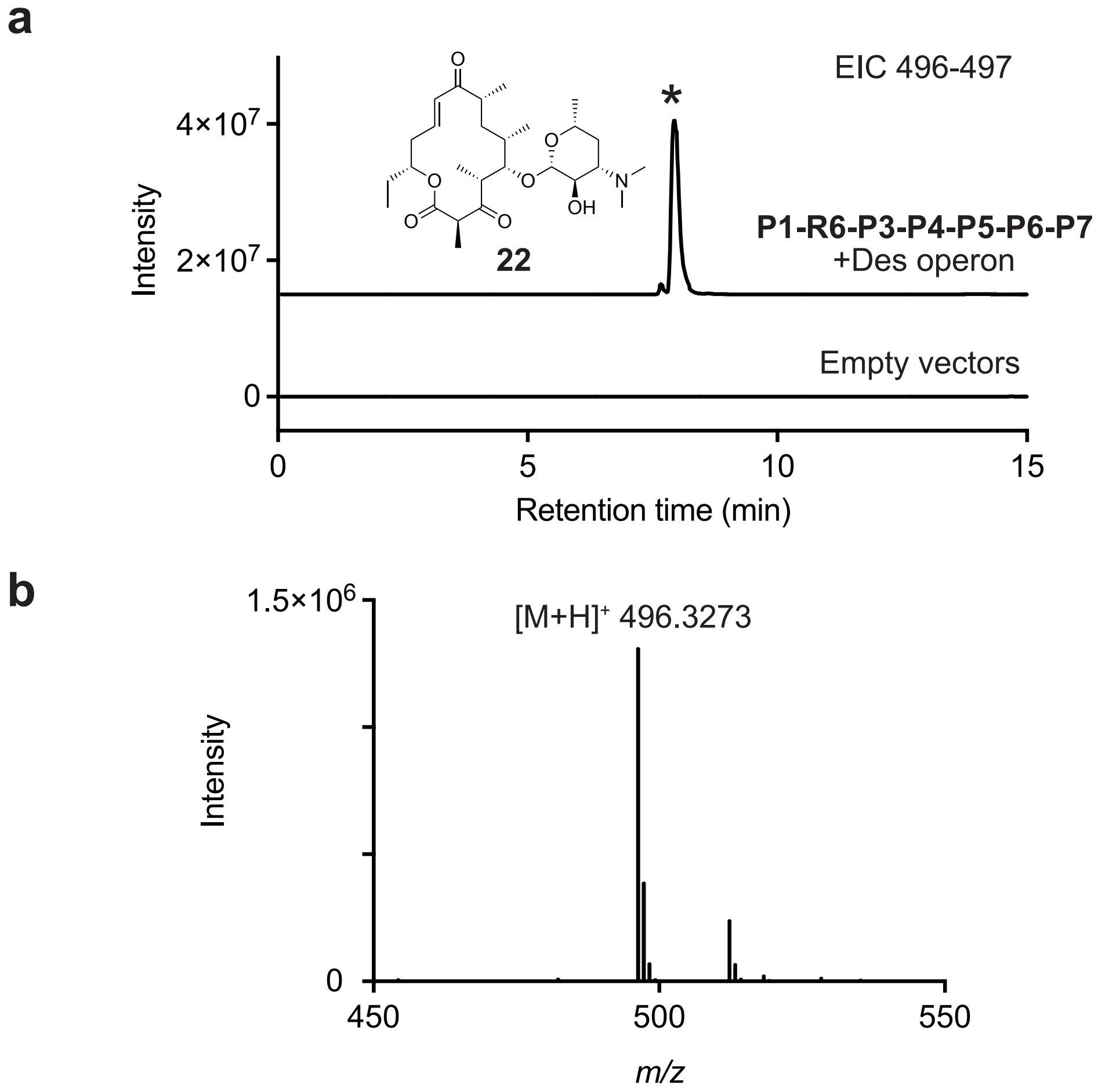

Figure S54. LC/MS analysis of the anticipated **P1**-**R6**-**P3**-**P4**-**P5**-**P6**-**P7**+Des product, **22**. a) EIC for the $[M+H]^+$ of **22** from the ethyl acetate extract of cells transformed with the PKS expression plasmids or corresponding empty vectors. b) MS spectrum of the main peak (*). Theoretical $[M+H]^+$ *m/z* of **22**, 496.3268; observed *m/z*, 496.3271 (0.6 ppm).

a

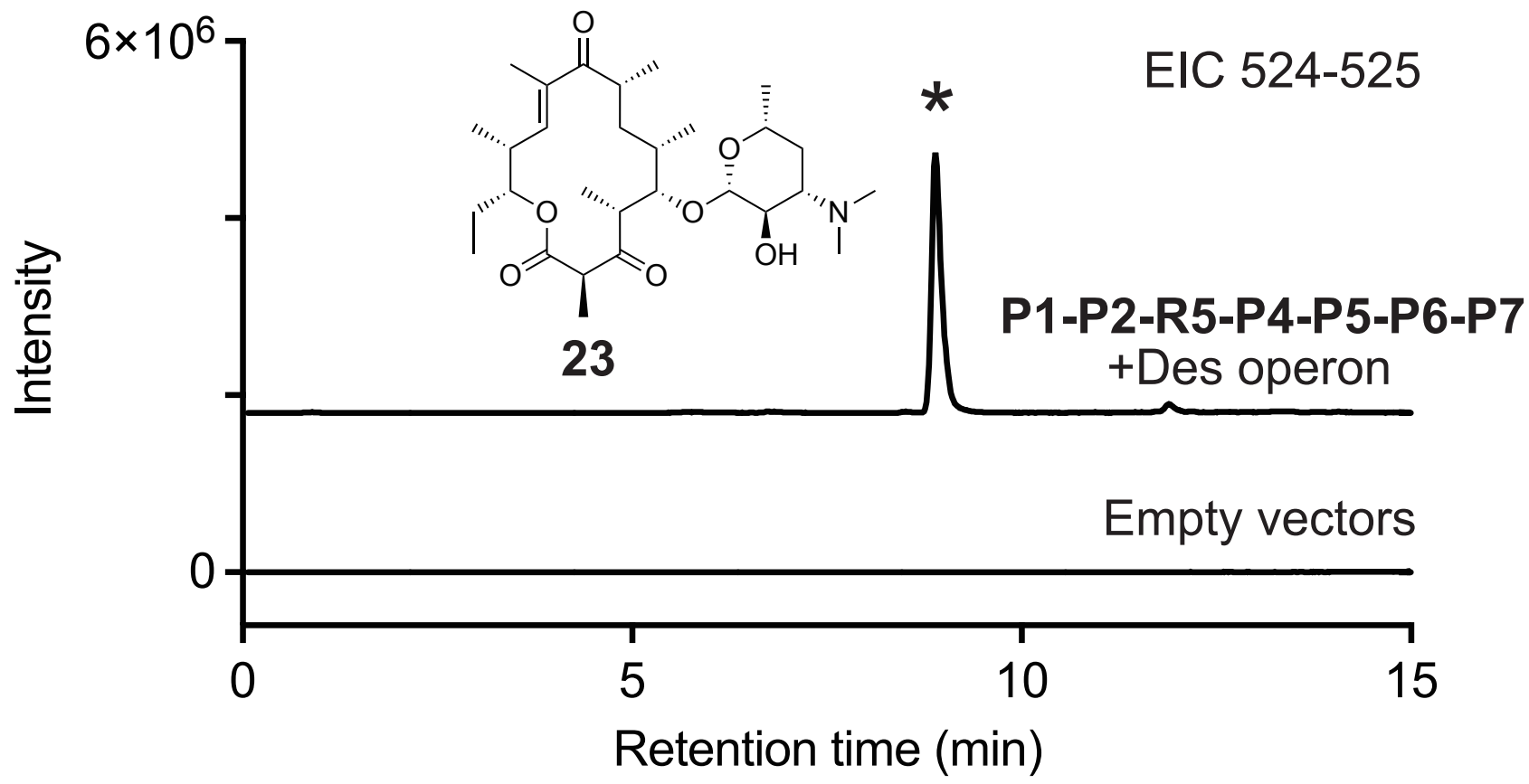

b

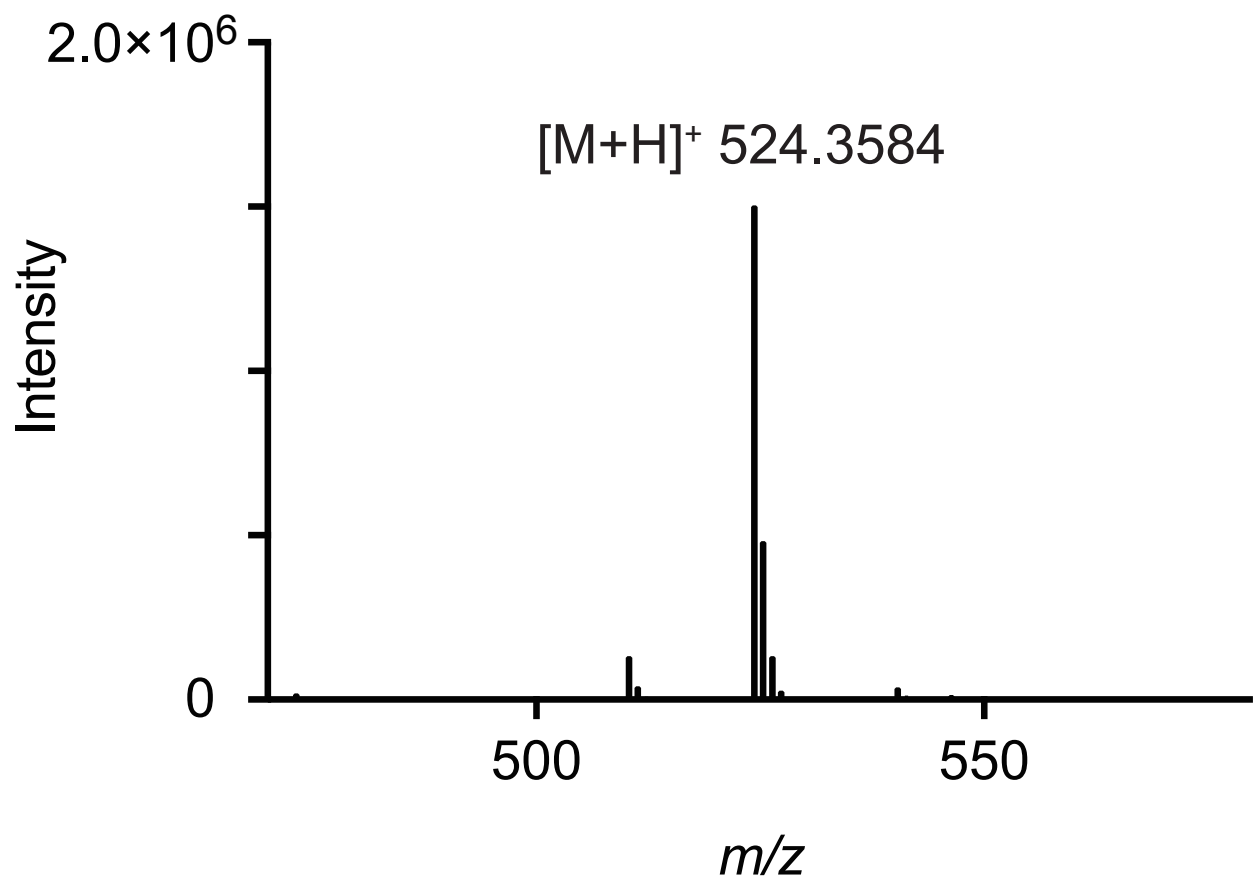

Figure S55. LC/MS analysis of the anticipated **P1-P2-R5-P4-P5-P6-P7**+Des product, **23**. a) EIC for the $[M+H]^+$ of **23** from the ethyl acetate extract of cells transformed with the PKS expression plasmids or corresponding empty vectors. b) MS spectrum of the main peak (*). Theoretical $[M+H]^+$ *m/z* of **23**, 524.3582; observed *m/z*, 524.3584 (0.6 ppm).

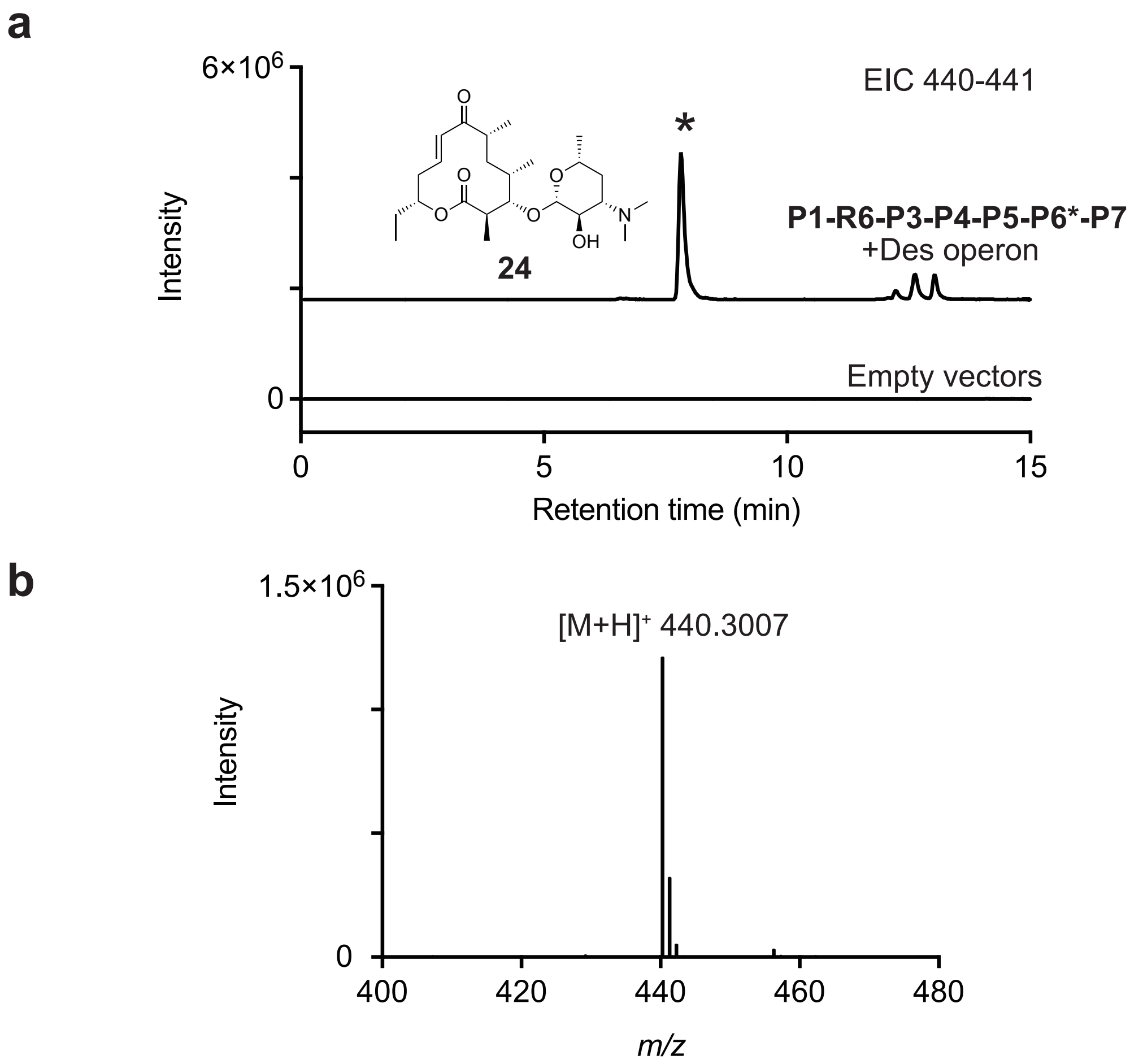

Figure S56. LC/MS analysis of the anticipated **P1-R6-P3-P4-P5-P6*-P7**+Des product, **24**. a) EIC for the $[M+H]^+$ of **24** from the ethyl acetate extract of cells transformed with the PKS expression plasmids or corresponding empty vectors. b) MS spectrum of the main peak (*). Theoretical $[M+H]^+$ *m/z* of **24**, 440.3006; observed *m/z*, 440.3007 (-0.22 ppm).

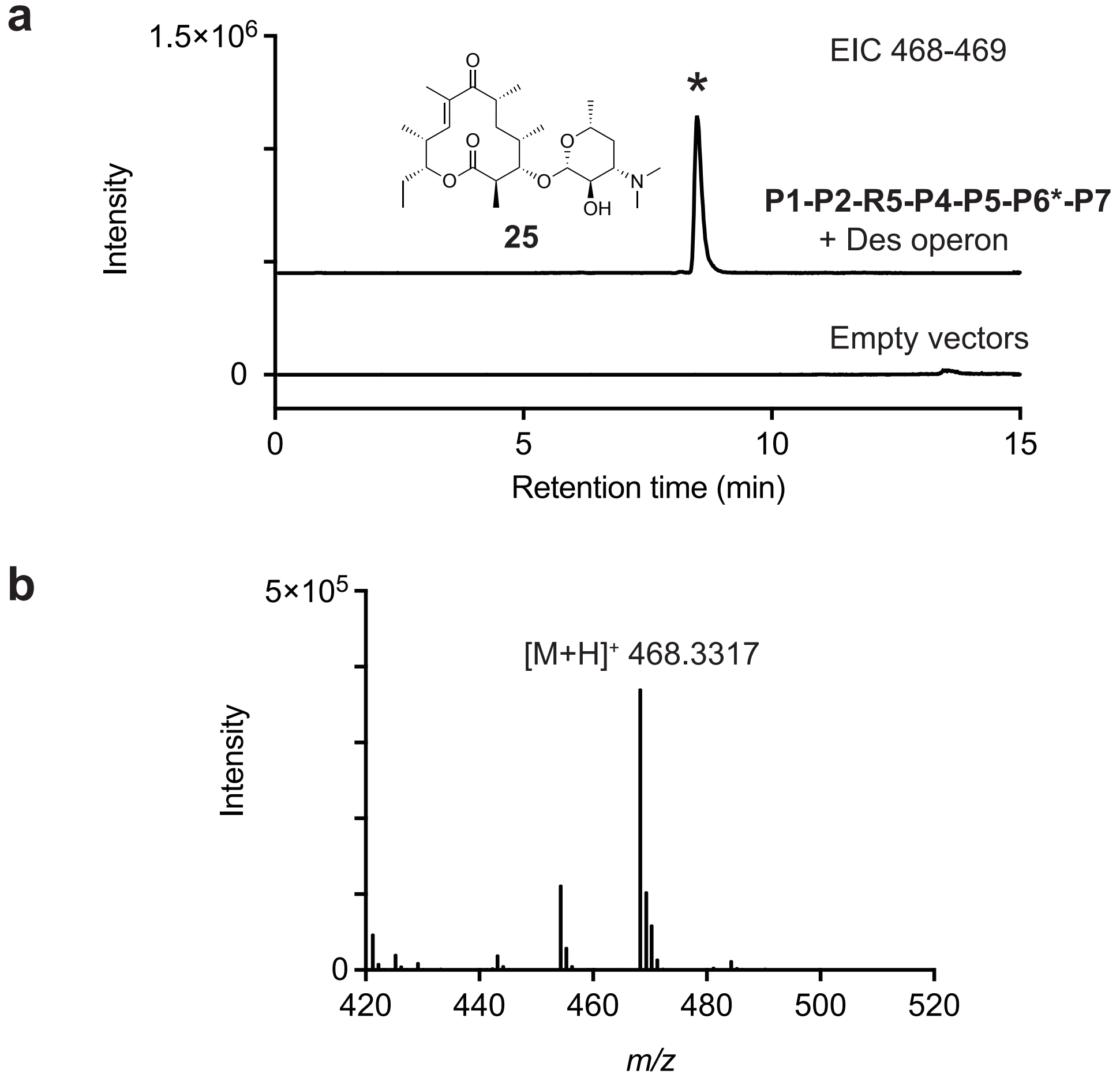

Figure S57. LC/MS analysis of the anticipated **P1-P2-R5-P4-P5-P6*-P7**+Des product, **25**. a) EIC for the $[M+H]^+$ of **25** from the ethyl acetate extract of cells transformed with the PKS expression plasmids or corresponding empty vectors. b) MS spectrum of the main peak (*). Theoretical $[M+H]^+$ *m/z* of **25**, 440.3006; observed *m/z*, 440.3005 (-0.22 ppm).

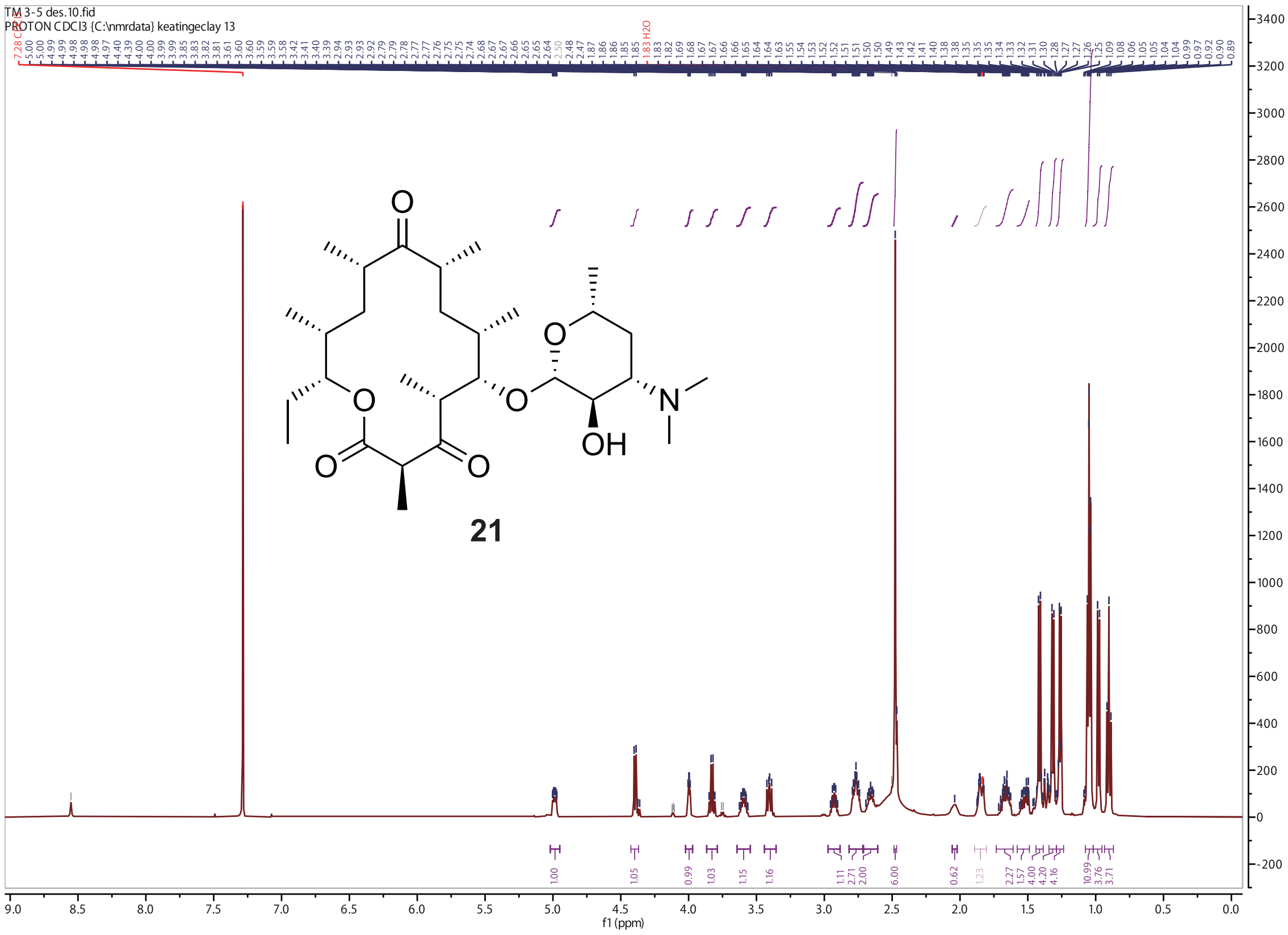

Figure S58. ^{1}H NMR spectrum of the anticipated **P1-P2-P5-P4-P5-P6-P7**+Des product, **21**.

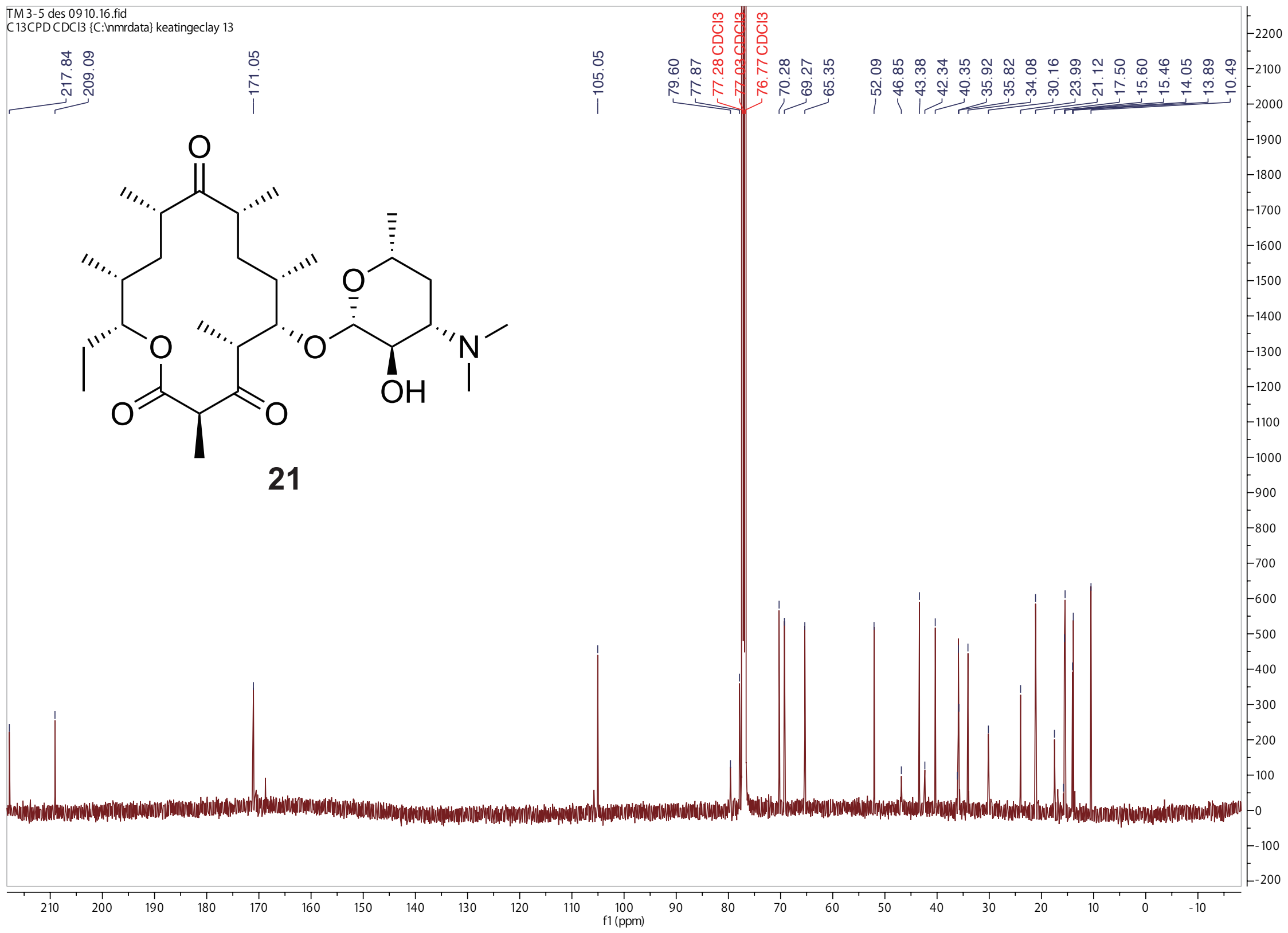

Figure S59. ^{13}C NMR spectrum of the anticipated **P1**-**P2**-**P5**-**P4**-**P5**-**P6**-**P7**+Des product, **21**.

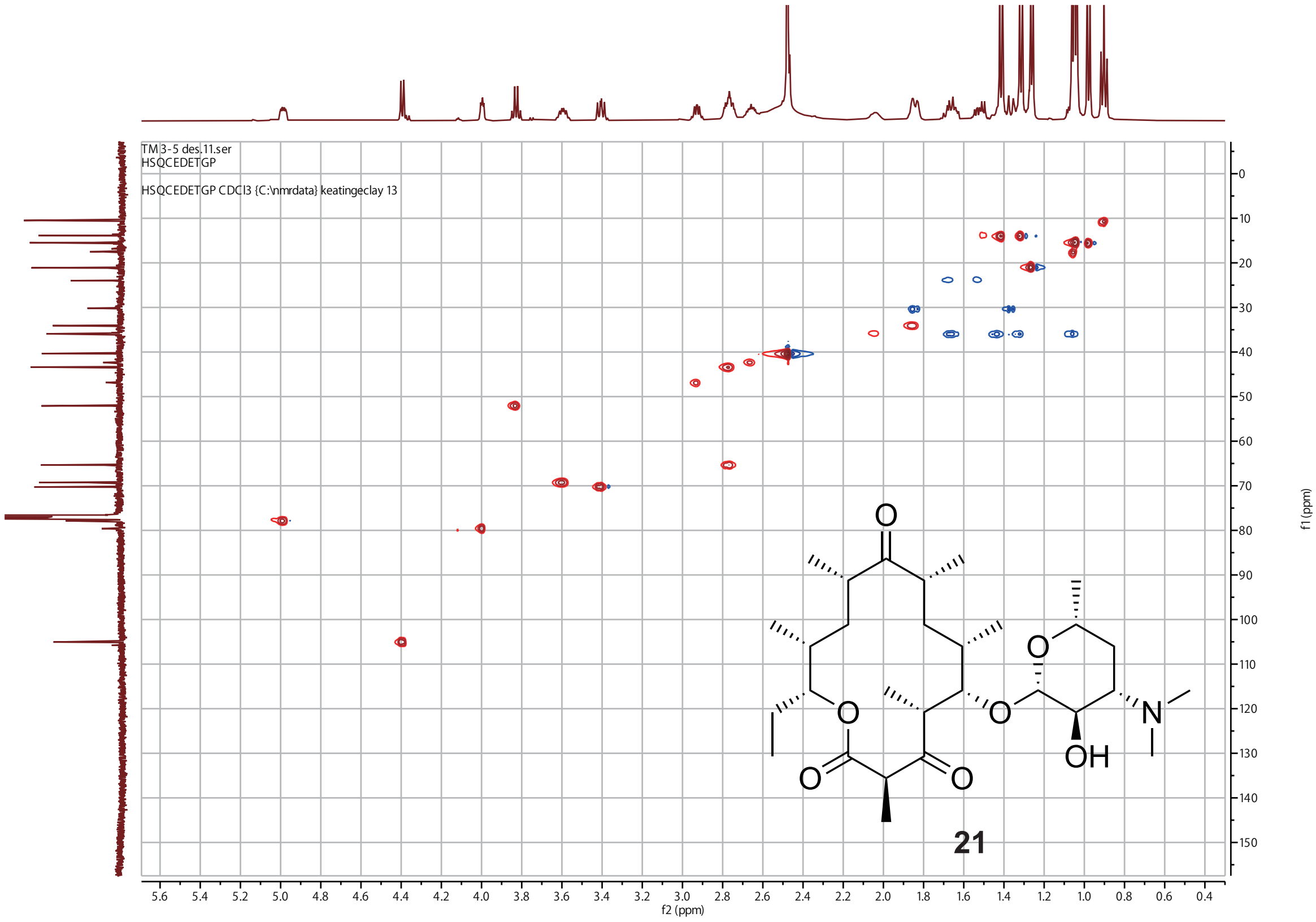

Figure S60. ^{1}H-^{13}C HSQC spectrum of the anticipated **P1-P2-P5-P4-P5-P6-P7**+Des product, **21**.

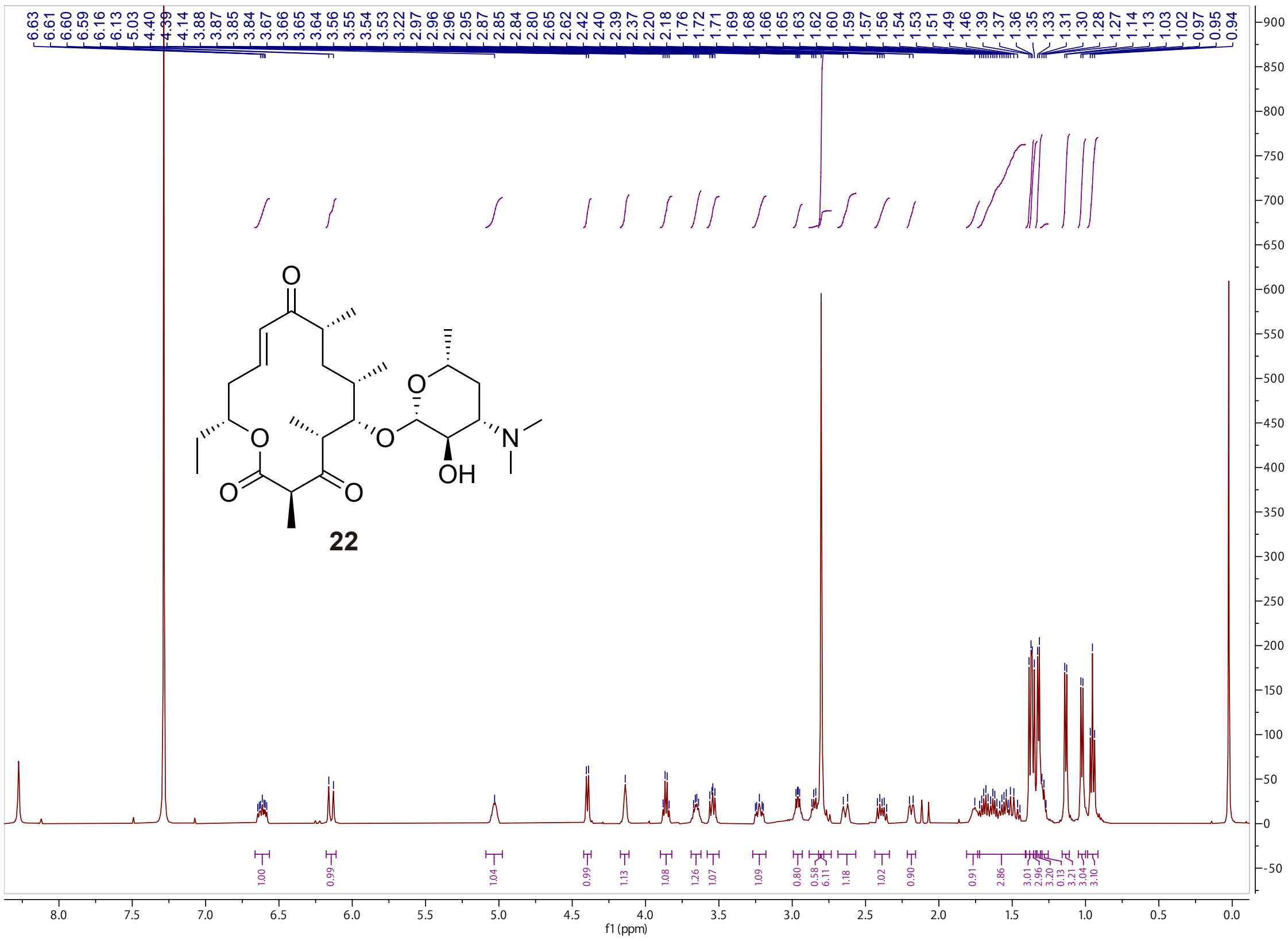

Figure S61. ^{1}H NMR spectrum of the anticipated **P1**-**R6**-**P3**-**P4**-**P5**-**P6**-**P7**+Des product, **22**.

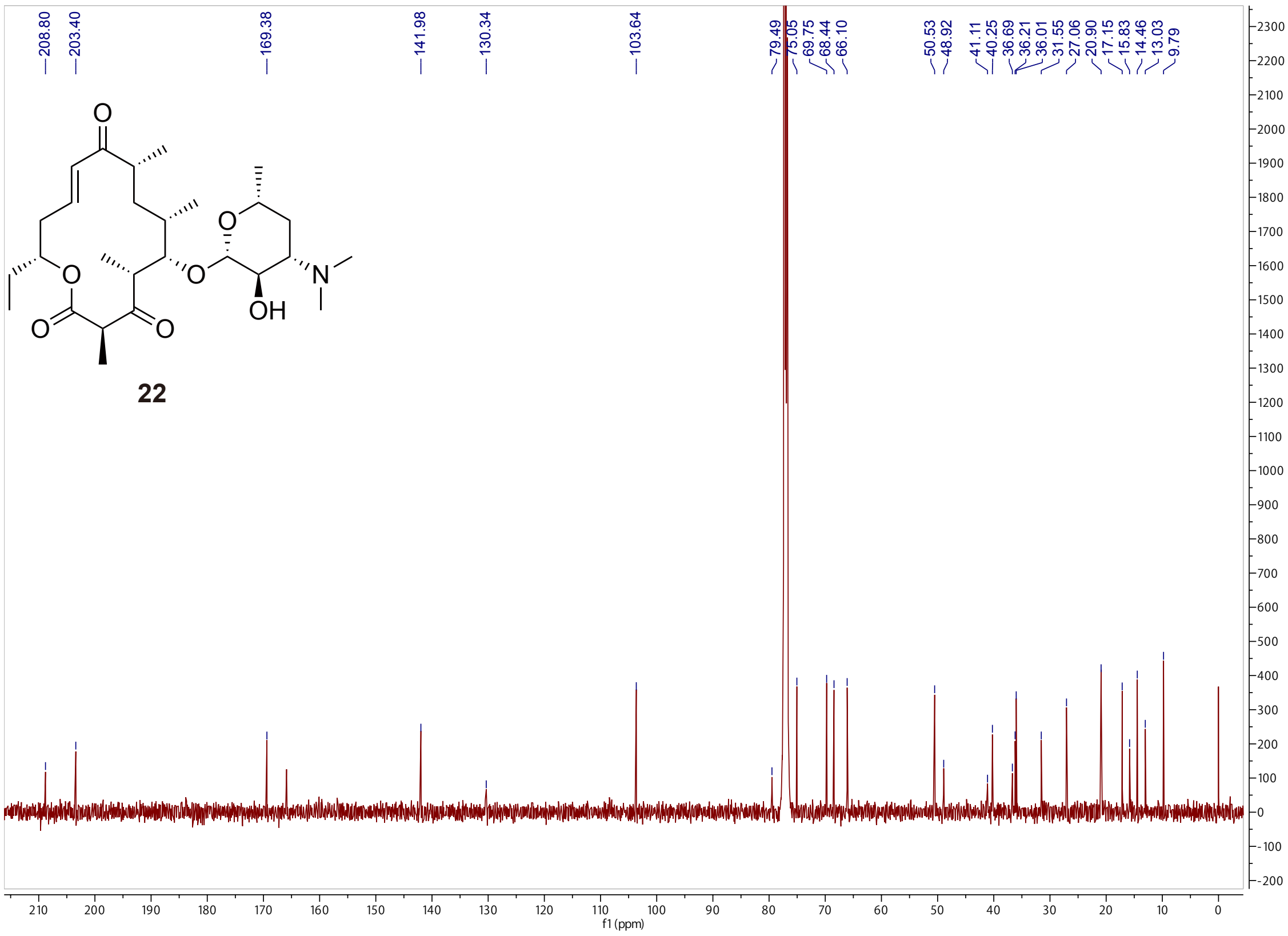

Figure S62. ^{13}C NMR spectrum of the anticipated **P1-R6-P3-P4-P5-P6-P7**+Des product, **22**.

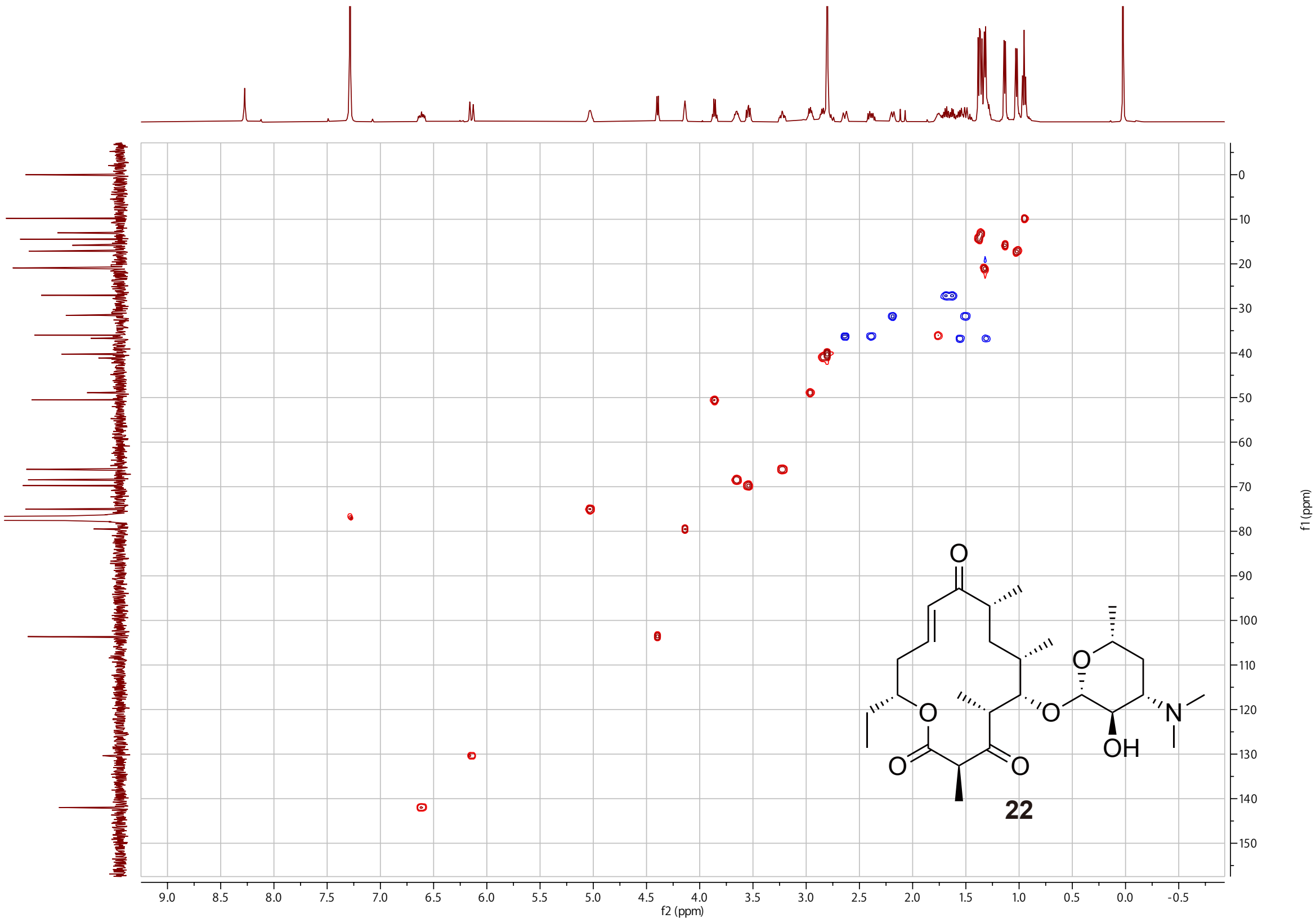

Figure S63. ^{1}H-^{13}C HSQC spectrum of the anticipated **P1-R6-P3-P4-P5-P6-P7**+Des product, **22**.

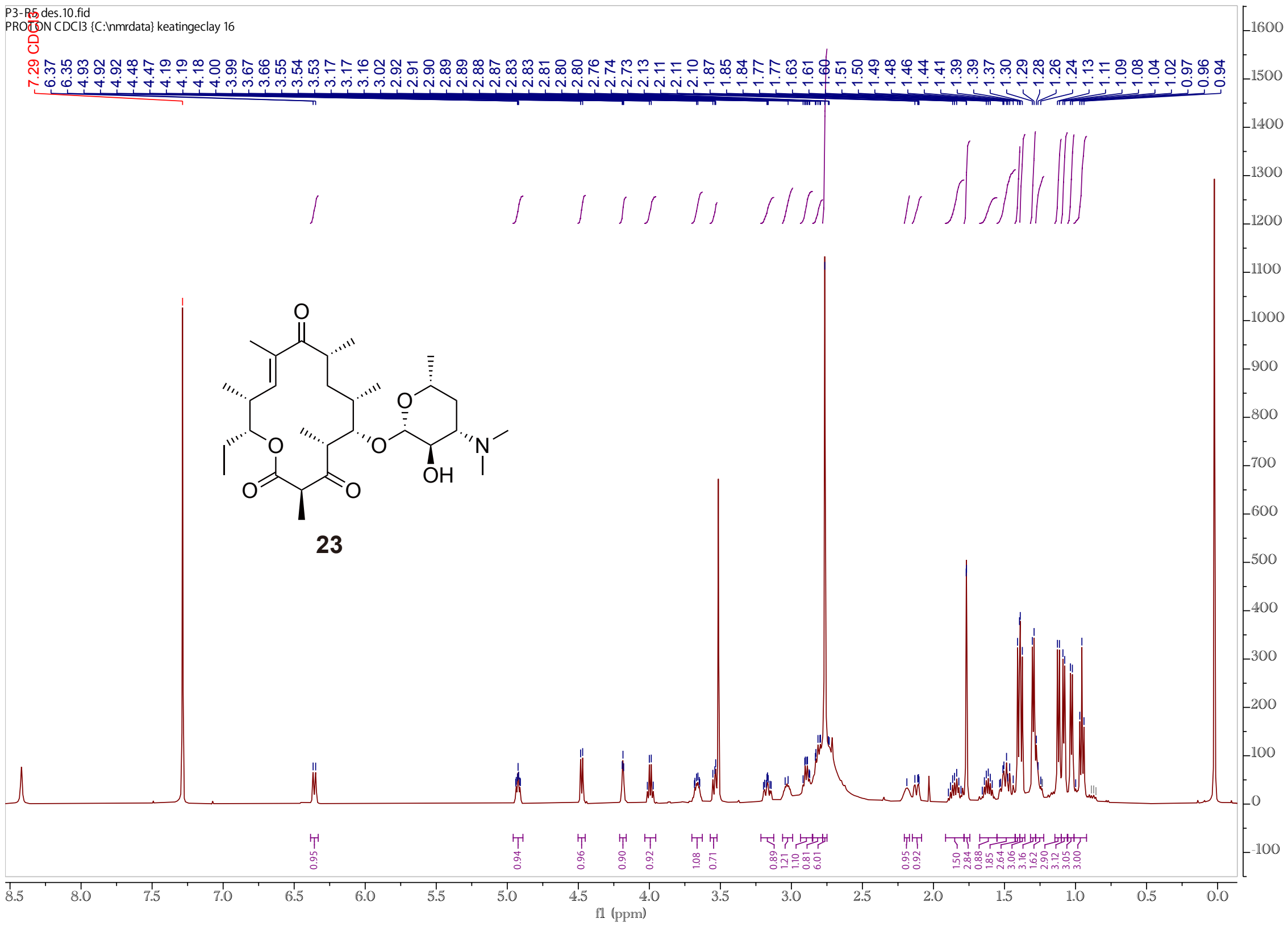

Figure S64. ^{1}H NMR spectrum of the anticipated **P1**-**P2**-**R5**-**P4**-**P5**-**P6**-**P7**+Des product, **23**.

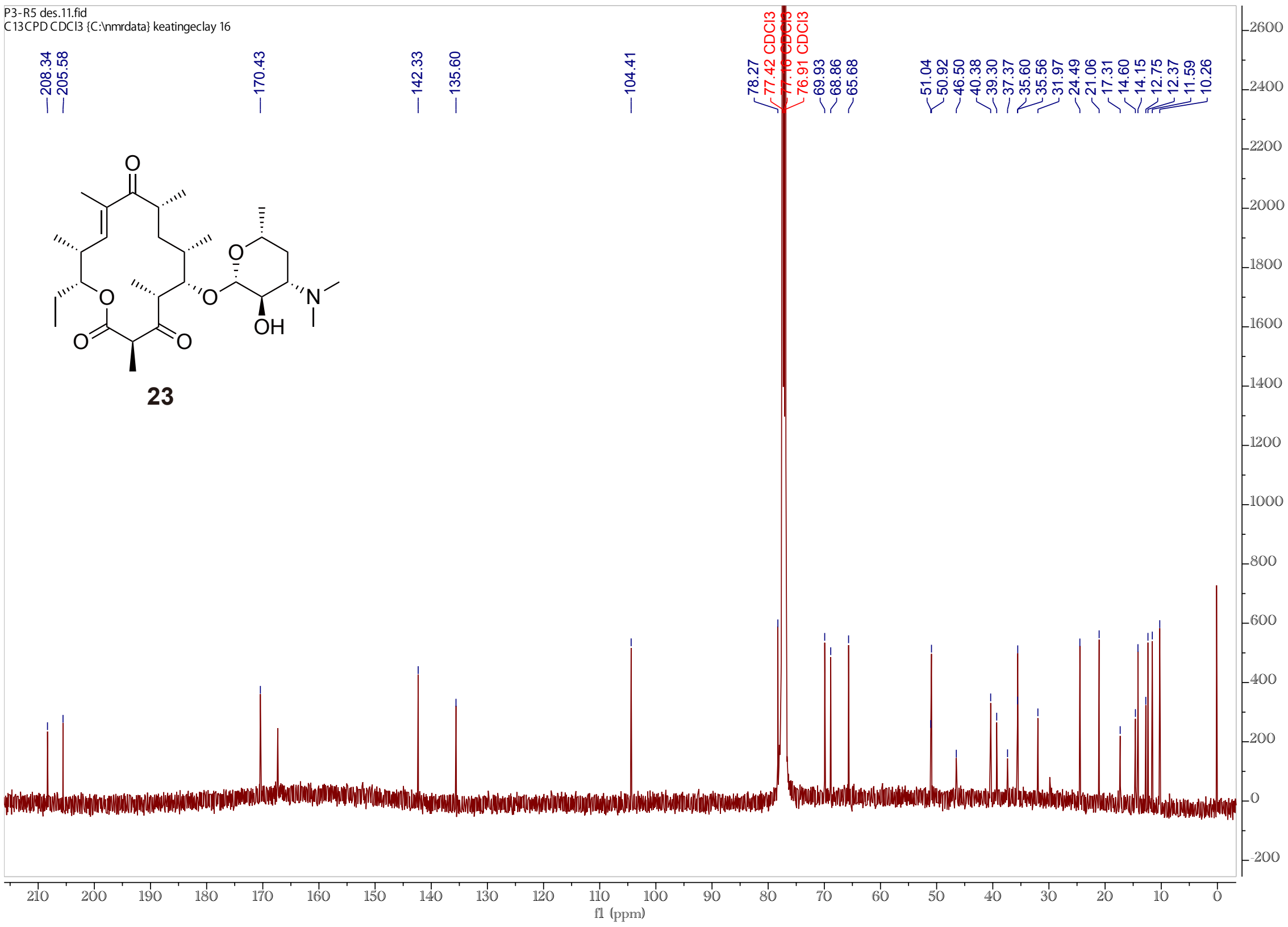

Figure S65. ¹³C NMR spectrum of the anticipated **P1-P2-R5-P4-P5-P6-P7**+Des product, **23**.

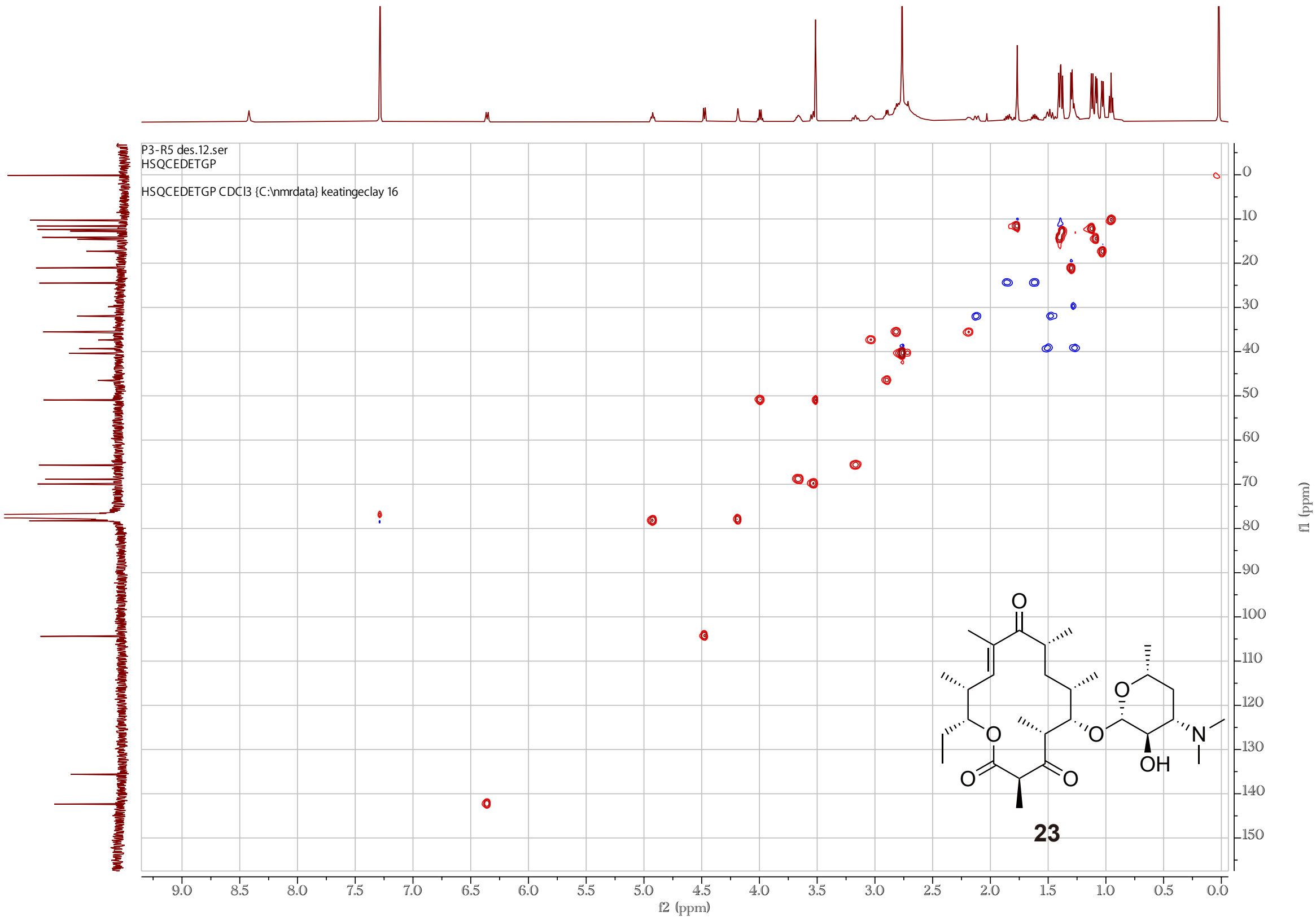

Figure S66. ^{1}H-^{13}C HSQC spectrum of the anticipated **P1-P2-R5-P4-P5-P6-P7**+Des product, **23**.

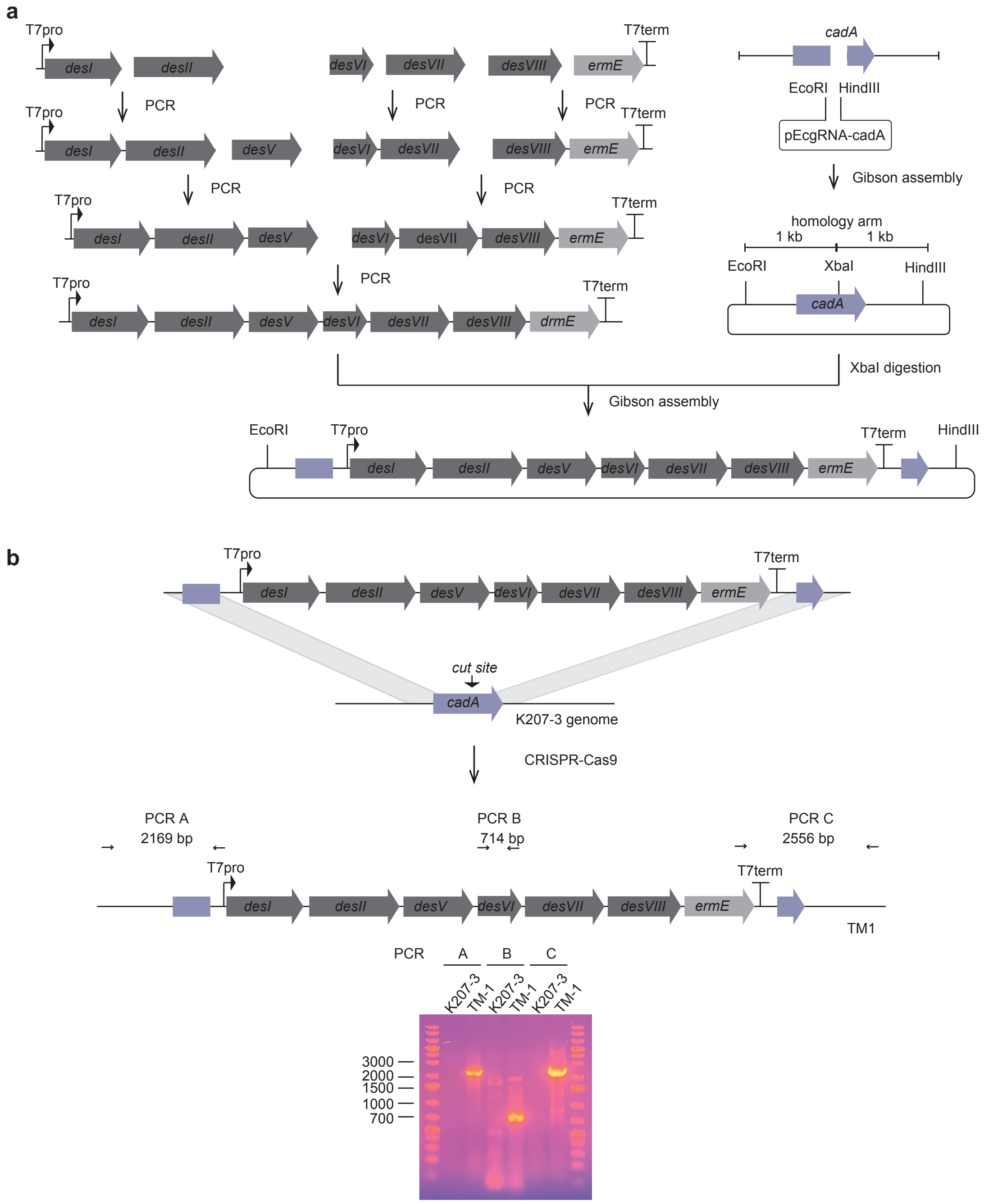

Figure S67. Integration of the desosamine biosynthesis/transfer (Des) operon into *cadA* locus of *E. coli* K207-3. a) Construction of pEcgRNA-ΔcadA:desI,II,V,VI,VII,VIII,ermE. To build the Des operon with short spacer sequences (aaggagatatacc, RBS underlined), each gene was amplified by PCR, and the amplicons were assembled through sequential overlap extension PCRs. The short spacer sequences are from the PCR primers. To construct plasmids that contain homology arms for *cadA* targeting, 1000 bp homology arms were amplified and assembled into the EcoRI/HindIII site of pEcgRNA-cadA through Gibson assembly. The Des operon was cloned into the XbaI site of the resulting plasmid by Gibson assembly to place the operon between the homology arms. The operon harboring the homology arms was cut from

the plasmid and used as the donor DNA for CRISPR/Cas9. b) Schematic representation of the genome integration by CRISPR/Cas9. PCRs A, B and C on genomic DNA confirmed the integration.

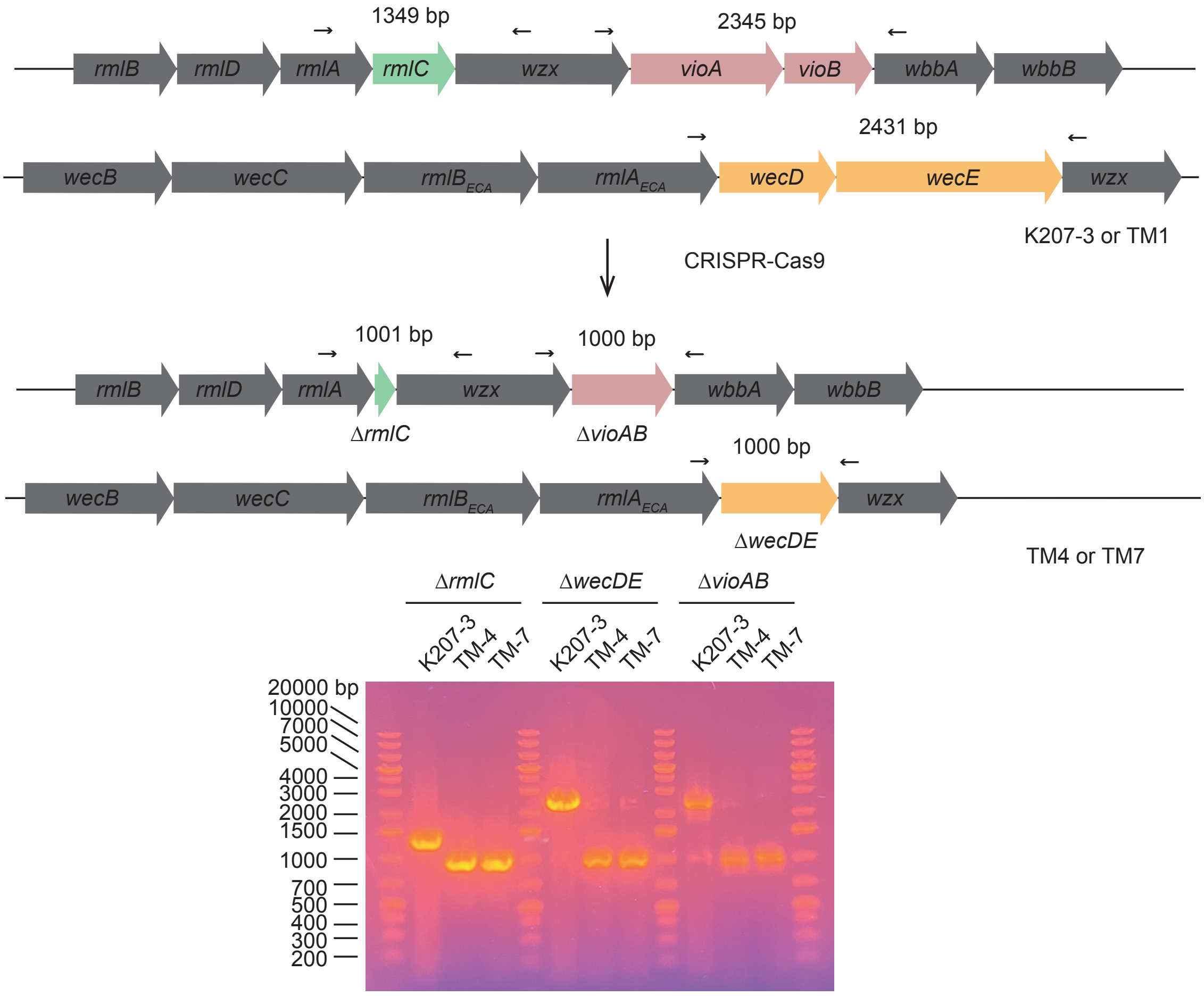

Figure S68. Inactivation of pathways competing with desosamine biosynthesis. Schematic for gene inactivation by CRISPR/Cas9. All gene inactivations are designed in frame to avoid the polar effect. Colony PCR confirmed gene inactivations.

a

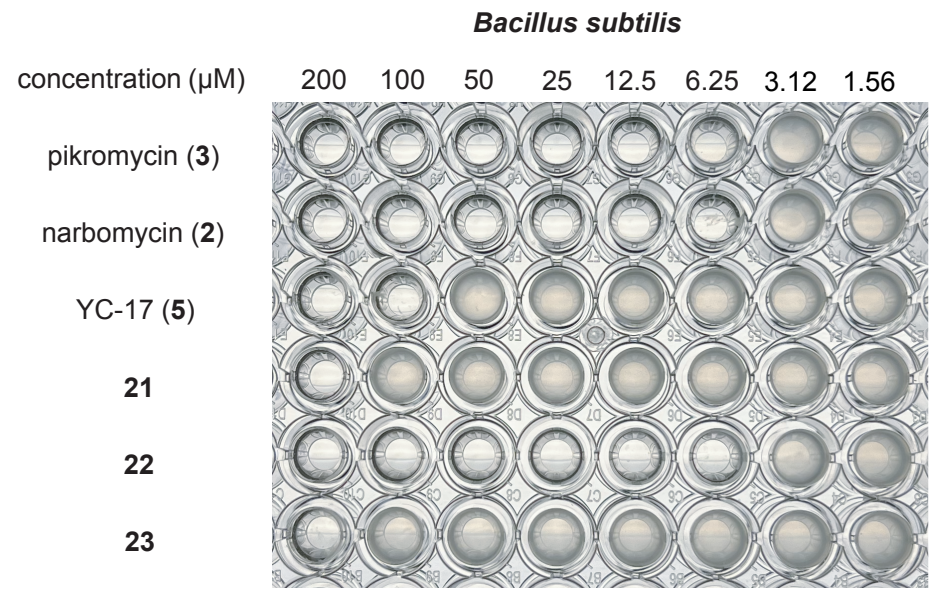

MIC values

pikromycin (**3**)	narbomycin (**2**)	YC-17 (**5**)	**21**	**22**	**23**
6.25 μM	6.25 μM	100 μM	200 μM	6.25 μM	200 μM

b

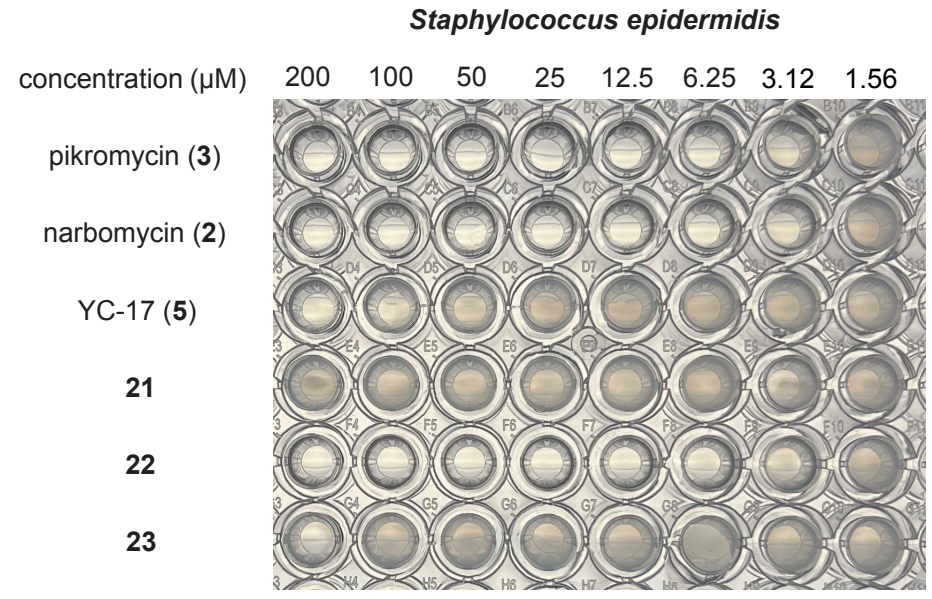

MIC values

pikromycin (**3**)	narbomycin (**2**)	YC-17 (**5**)	**21**	**22**	**23**
6.25 μM	6.25 μM	200 μM	> 200 μM	6.25 μM	200 μM

c

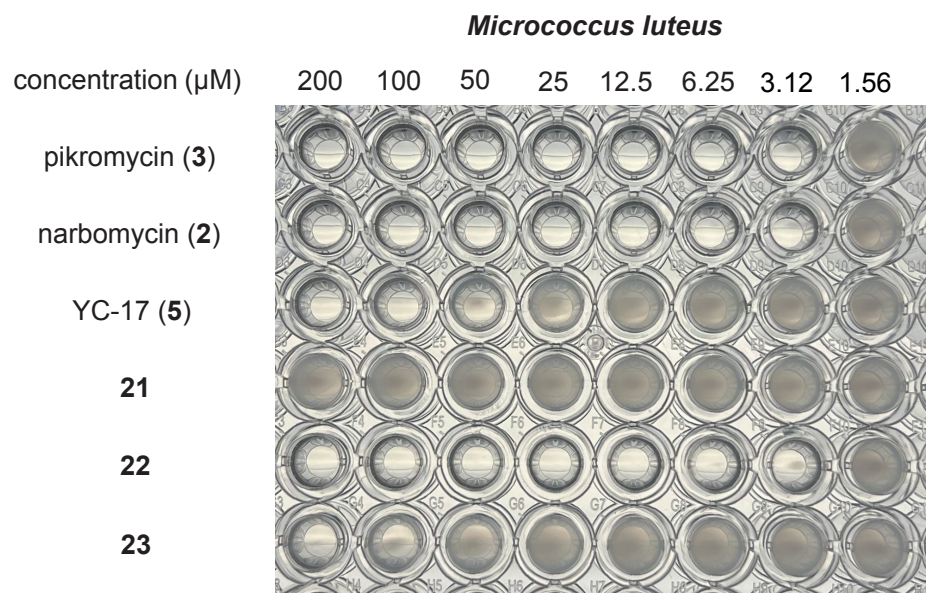

MIC values

pikromycin (**3**)	narbomycin (**2**)	YC-17 (**5**)	**21**	**22**	**23**
3.12 μM	3.12 μM	100 μM	> 200 μM	3.12 μM	200 μM

d

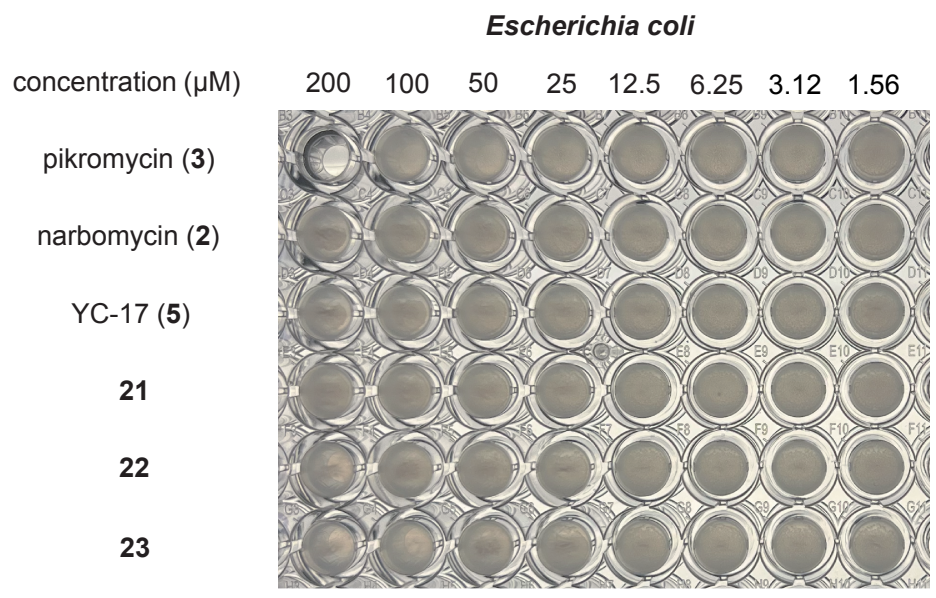

MIC values

pikromycin (**3**)	narbomycin (**2**)	YC-17 (**5**)	**21**	**22**	**23**
200 μM	> 200 μM	> 200 μM	> 200 μM	> 200 μM	> 200 μM

e

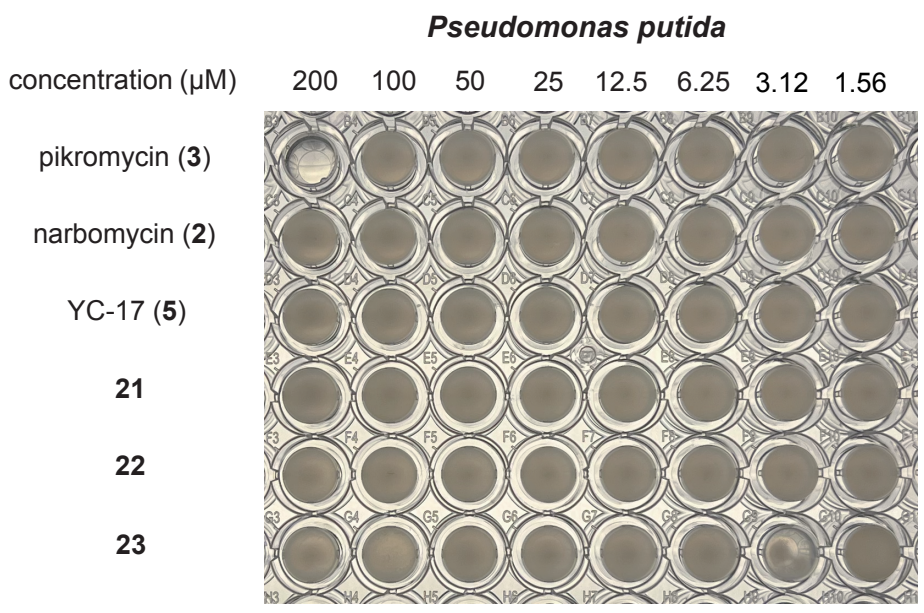

MIC values

pikromycin (**3**)	narbomycin (**2**)	YC-17 (**5**)	**21**	**22**	**23**
200 μM	> 200 μM	> 200 μM	> 200 μM	> 200 μM	> 200 μM

Figure S69. MIC assay. MIC values of purified compounds against both Gram-positive [*Bacillus subtilis* (**a**), *Staphylococcus epidermidis* (**b**), *Micrococcus luteus* (**c**)] and Gram-negative [*Escherichia coli* (**d**) and *Pseudomonas putida* (**e**)] bacteria were evaluated. Cells were grown in a 96-well plate in M-H medium containing 2-fold serial dilutions of purified compounds (**2**, **5**, **21, 22,** and **23** from *E. coli*, **3** from commercial source). The lowest concentration that prevented visible bacterial growth in the wells is reported as the MIC. The MICs obtained for narbomycin, pikromycin, and YC-17 are similar to those previously reported for these compounds on bacteria from the Bacilli class[8,9].

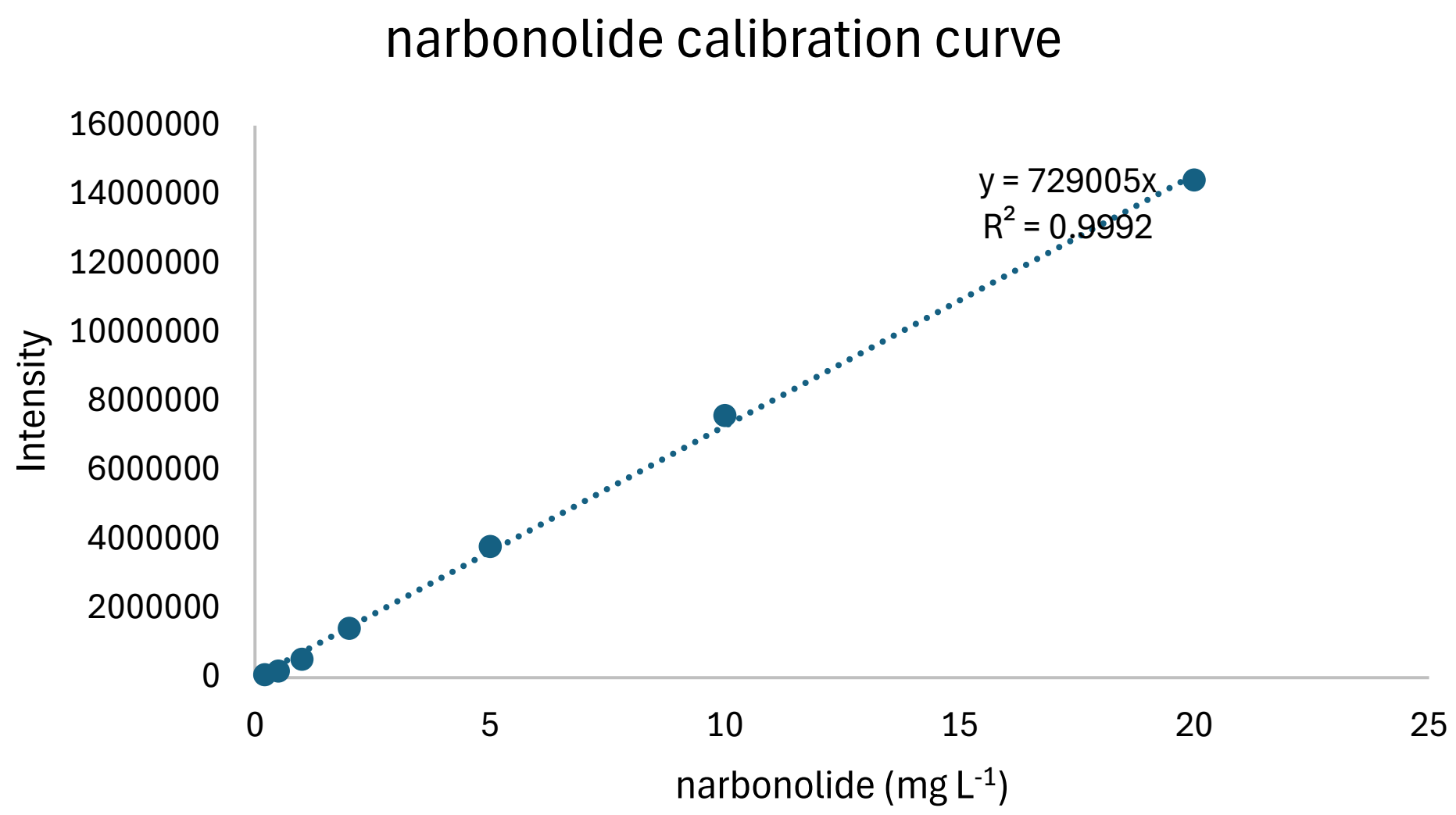

Figure S70. Calibration curve for quantification of narbonolide, 10-dml, and their derivatives. Narbonolide purified from K207-3 cells expressing **P1-P2-P3-P4-P5-P6-P7** was used to generate the calibration curve. Samples (1 μL) were injected into LC/MS, and the EIC peak areas (EIC 510-511) were measured.

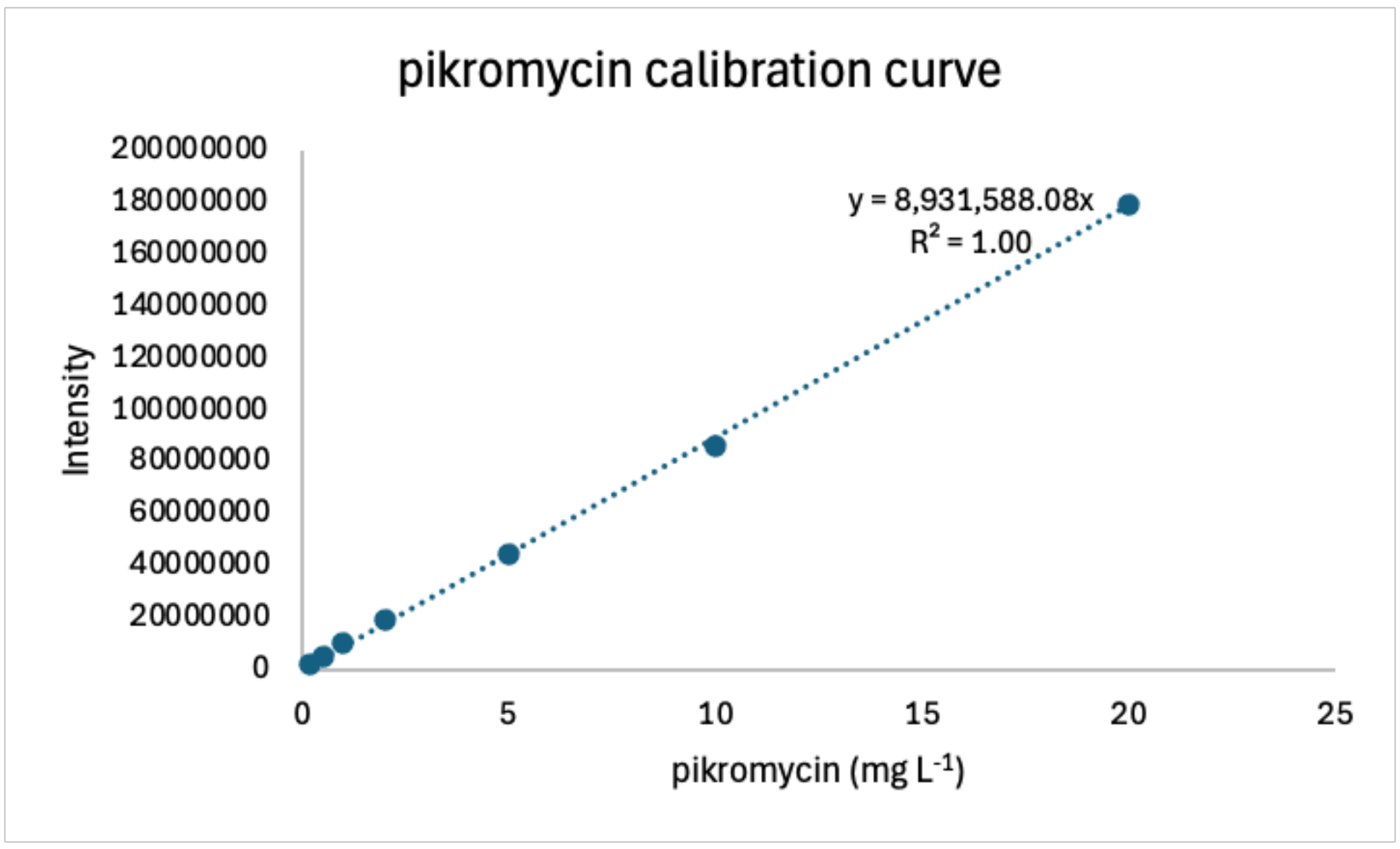

Figure S71. Calibration curve for quantification of narbomycin, pikromycin, YC-17, methymycin, and their derivatives. Commercial pikromycin was used to generate the calibration curve. Samples (1 μL) were injected into LC/MS, and the EIC peak areas (EIC 526-527) were measured.

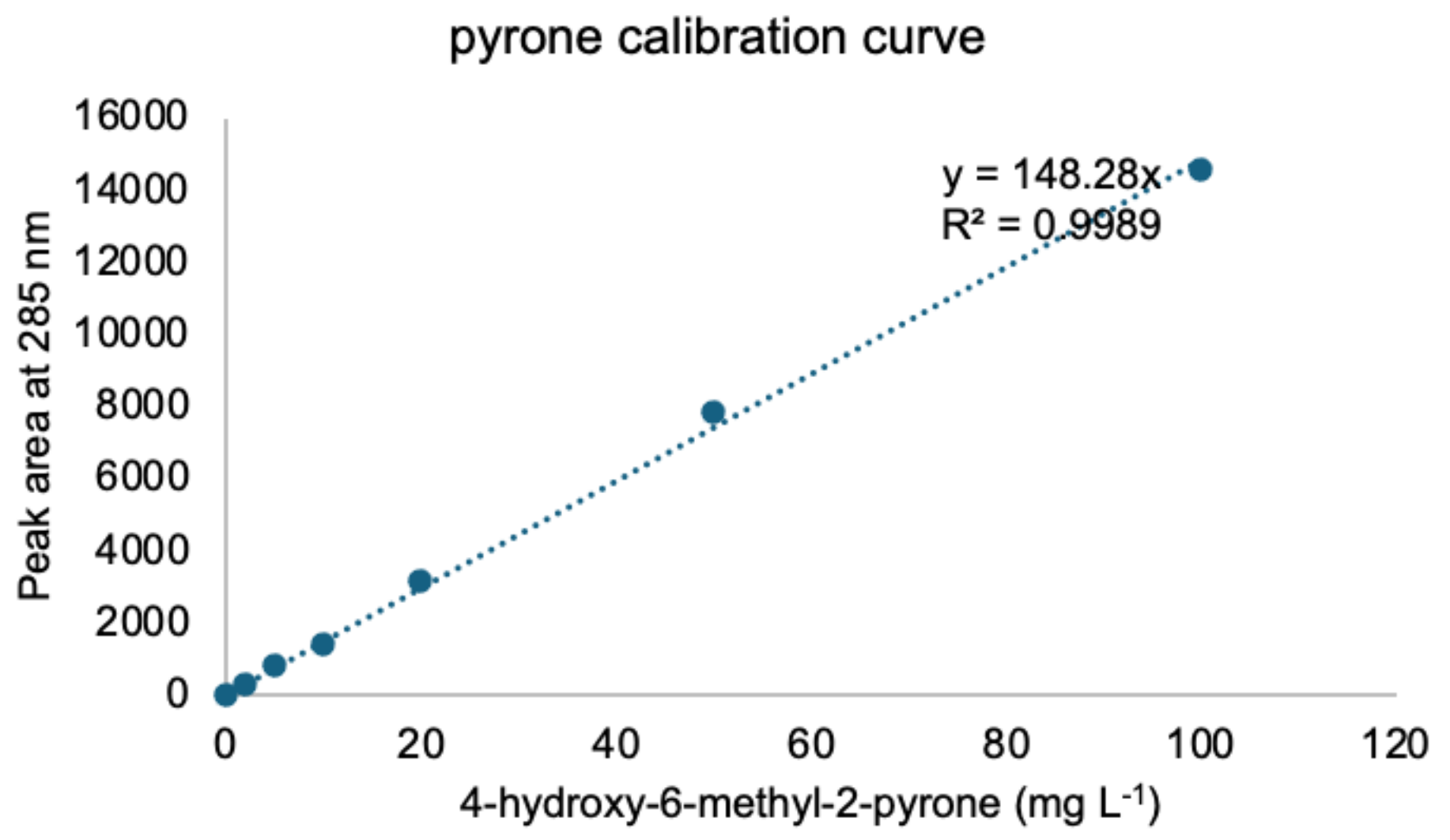

Figure S72. Calibration curve for quantification of pyrone products. 4-hydroxy-6-methy-2-pyrone (purchased from Sigma-Aldrich) was used to generate the calibration curve. Samples (10 µL) were injected into LC/MS, and the UV peak areas at 285 nm were measured.

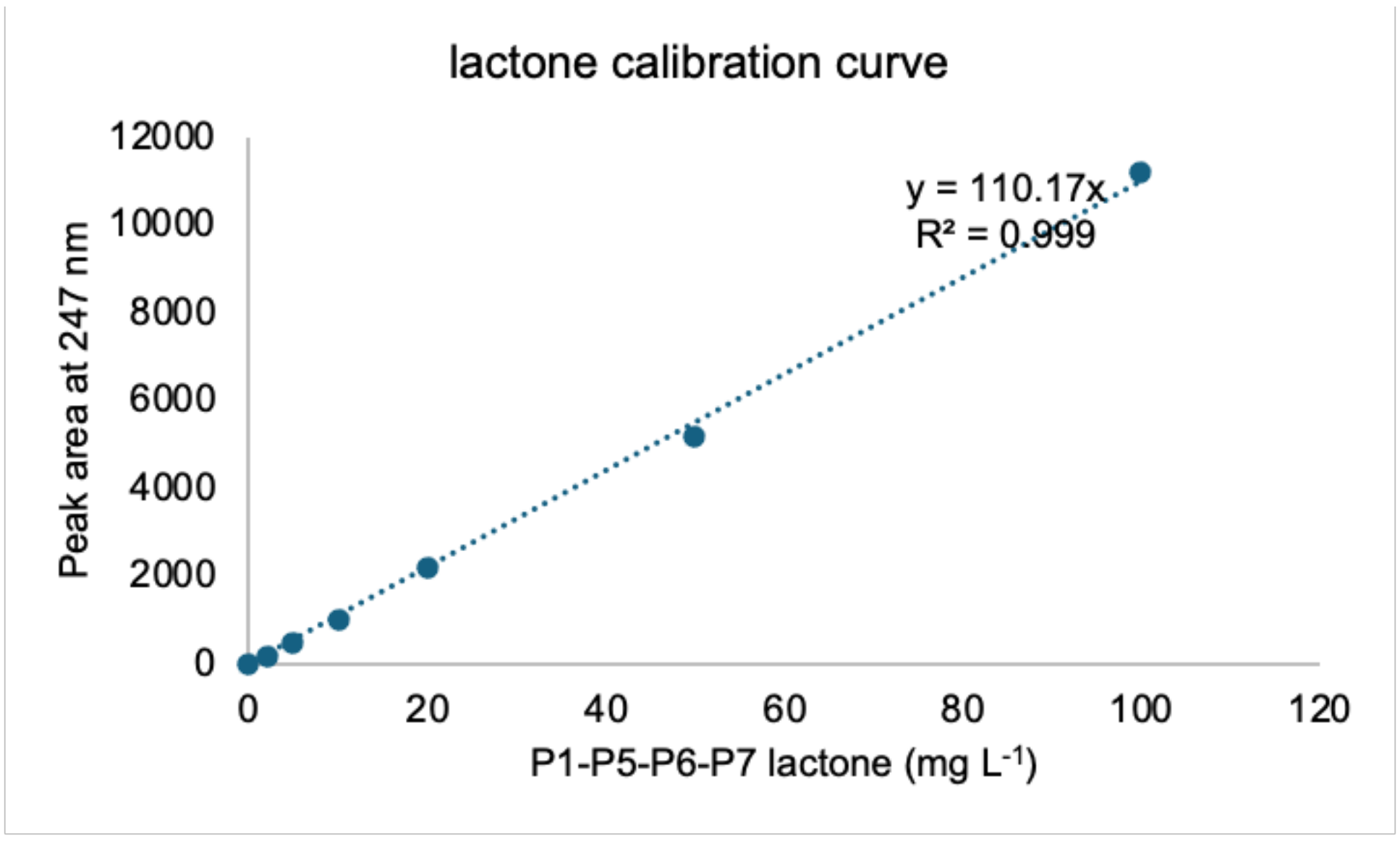

Figure S73. Calibration curve for quantification of δ-lactone products. The purified product of **P1-P5-P6-P7**[10] was used to generate the calibration curve. Samples (10 µL) were injected into LC/MS, and the UV peak areas at 247 nm were measured.

Table S1. Strains used in this study.

Strain	Genotype	Source
Escherichia coli DH5α	*fhuA2Δ(argF-lacZ)U169 phoA glnV44 Φ80Δ(lacZ)M15 gyrA96 recA1 relA1 endA1 thi-1 hsdR17*	NEB
Escherichia coli ATCC 25922	Quality control strain	ATCC
K207-3	F-*ompT hsdS*$_B$ *(r$^-$m$^-$), gal dcm* (DE3), *panD::panDS25A,* D*prpRBCD::*T7prom-*sfp,* T7prom-*prpE, ygfG::*T7prom-*accA1-*T7prom-*pccB*	Ref.[11]
TM1	K207-3 *cadA::*T7prom-*desI desII desV desVI desVII desVIII ermE*	This work
TM2	K207-3 ∆*rmlC*	This work
TM3	K207-3 ∆*rmlC wecDE*	This work
TM4	K207-3 ∆*rmlC wecDE vioAB*	This work
TM5	TM1 ∆*rmlC*	This work
TM6	TM1 ∆*rmlC wecDE*	This work
TM7	TM1 ∆*rmlC wecDE vioAB*	This work
Bacillus subtilis strain 168	*trpC2*	ATCC
Staphylococcus epidermidis ATCC 14990	Quality control strain	ATCC
Micrococcus luteus ATCC 4698	Quality control strain	ATCC
Pseudomonas putida KT2440	*rmo*$^-$ *mod*$^+$	ATCC

Table S2. Plasmids used in this study. *str*, streptomycin resistance gene; *kan*, kanamycin resistance gene; *cat*, chloramphenicol acetyltransferase gene; *bla*, ampicillin resistance gene; *aac(3)-IV*, an aminoglycoside 3-N-acetyltransferase.

Plasmid	Description	Source
pUC19	*E. coli* cloning vector, ColE1 ori, *bla*	NEB
pCDF-1b	*E. coli* expression vector, CloDF13 ori, *str*	Novagene
pET28-b	*E. coli* expression vector, ColE1 ori, *kan*	Novagene
pACYCDuet-1	*E. coli* expression vector, p15A ori, *cat*	Novagene
pMKBAC02	*E. coli* expression BAC, ori2, *aac(3)-IV*	Ref.[3]
pEcCas	*repA101*(Ts) *kan* P_{cas}-*cas9* P_{araB}-*Red* *lacI*^{q} P_{trc}-sgRNA-*pMB1*, sacB, P_{rhaB}-sgRNA-pMB1, pSC101	Addgene [5]
pEcgRNA	pMB1 *aadA* sgRNA, *ccdB, str*	Addgene [5]
pUC19-**S3**DD	pUC19 containing **S3**DD regulator between HindIII and XbaI	Ref.[2]
pUC19-**S5**DD	pUC19 containing **S5**DD regulator between HindIII and XbaI	Ref.[2]
pUC19-**S8**DD	pUC19 containing **S8**DD regulator between HindIII and XbaI	Ref.[2]
pUC19-**E5**DD	pUC19 containing **E5**DD regulator between HindIII and XbaI	This work
pUC19-**P3**DD	pUC19 containing **P3**DD regulator between HindIII and XbaI	This work
pUC19-**P5**DD	pUC19 containing **P5**DD regulator between HindIII and XbaI	This work
pUC19-**P6**DD	pUC19 containing **P6**DD regulator between HindIII and XbaI	This work
pUC19-**A1**DD	pUC19 containing **A1**DD regulator between HindIII and XbaI	This work
pUC19-**S3**DD-**P2**	Cloning plasmid containing **S3**DD-**P2**	Ref.[2]
pUC19-**S5**DD-**P2**	Cloning plasmid containing **S5**DD-**P2**	Ref.[2]
pUC19-**S8**DD-**P2**	Cloning plasmid containing **S8**DD-**P2**	Ref.[2]
pUC19-**E5**DD-**P2**	Cloning plasmid containing **E5**DD-**P2**	This work
pUC19-**S3**DD-**P3**	Cloning plasmid containing **S3**DD-**P3**	Ref.[2]
pUC19-**S5DD**-**P3**	Cloning plasmid containing **S5DD**-**P3**	Ref.[2]
pUC19-**S8**DD-**P3**	Cloning plasmid containing **S8**DD-**P3**	Ref.[2]
pUC19-**E5**DD-**P3**	Cloning plasmid containing **E5**DD-**P3**	This work
pUC19-**P3**DD-**P3**	Cloning plasmid containing **P3**DD-**P3**	This work
pUC19-**S3**DD-**P4**	Cloning plasmid containing **S3**DD-**P4**	Ref.[2]
pUC19-**S5**DD-**P4**	Cloning plasmid containing **S5**DD-**P4**	Ref.[2]
pUC19-**S8**DD-**P4**	Cloning plasmid containing **S8**DD-**P4**	Ref.[2]
pUC19-**E5**DD-**P4**	Cloning plasmid containing **E5**DD-**P4**	This work
pUC19-**S3**DD-**P5**	Cloning plasmid containing **S3**DD-**P5**	Ref.[2]
pUC19-**S5**DD-**P5**	Cloning plasmid containing **S5**DD-**P5**	Ref.[2]
pUC19-**S8**DD-**P5**	Cloning plasmid containing **S8**DD-**P5**	Ref.[2]
pUC19-**E5**DD-**P5**	Cloning plasmid containing **E5**DD-**P5**	This work

pUC19-**P3**DD-**P5**	Cloning plasmid containing **P3**DD-**P5**	This work
pUC19-**A1**DD-**P5**	Cloning plasmid containing **A1**DD-**P5**	This work
pUC19-**S3**DD-**P6**	Cloning plasmid containing **S3**DD-**P6**	Ref.[2]
pUC19-**S5**DD-**P6**	Cloning plasmid containing **S5**DD-**P6**	Ref.[2]
pUC19-**S5**DD-**P6***	Cloning plasmid containing **S5**DD-**P6***	This work
pUC19-**S8**DD-**P6**	Cloning plasmid containing **S8**DD-**P6**	Ref.[2]
pUC19-**E5**DD-**P6**	Cloning plasmid containing **E5**DD-**P6**	This work
pUC19-**P3**DD-**P6**	Cloning plasmid containing **P3**DD-**P6**	This work
pUC19-**E2** [**S8**DD]	Cloning plasmid containing **E2** [**S8**DD]	This work
pUC19-**E6** [**E5**DD]	Cloning plasmid containing **E6** [**E5**DD]	This work
pUC19-**R5** [**S3**DD]	Cloning plasmid containing **S2** [**S3**DD]	This work
pUC19-**R6** [**S5**DD]	Cloning plasmid containing **S2** [**S8**DD]	This work
pCDF-**P1**-**P4**^{N}	Expression plasmid containing **P1**-**P4**^{N}	This work
pET28b-^{C}**P4**-**P7**	Expression plasmid containing ^{C}**P4**-**P7**	This work
pCDF-**P1**-**P2**-**P4**^{N}	Expression plasmid containing **P1**-**P2**-**P4**^{N}	This work
pCDF-**P1**-**P3**-**P4**^{N}	Expression plasmid containing **P1**-**P3**-**P4**^{N}	This work
pCDF-**P1**-**P2**-**P3**-**P4**^{N}	Expression plasmid containing **P1**-**P2**-**P3**-**P4**^{N}	This work
pCDF-**P1**-**P2**-**P3**[**P3**DD]-**P4**^{N}	Expression plasmid containing **P1**-**P2**-**P3**[**P3**DD]-**P4**^{N}	This work
pCDF-**P1**-**P3**-**P3**-**P4**^{N}	Expression plasmid containing **P1**-**P3**-**P3**-**P4**^{N}	This work
pCDF-**P1**-**P4**-**P3**-**P4**^{N}	Expression plasmid containing **P1**-**P4**-**P3**-**P4**^{N}	This work
pCDF-**P1**-**P5**-**P3**-**P4**^{N}	Expression plasmid containing **P1**-**P5**-**P3**-**P4**^{N}	This work
pCDF-**P1**-**P6**-**P3**-**P4**^{N}	Expression plasmid containing **P1**-**P6**-**P3**-**P4**^{N}	This work
pCDF-**P1**-**P2**-**P2**-**P4**^{N}	Expression plasmid containing **P1**-**P2**-**P2**-**P4**^{N}	This work
pCDF-**P1**-**P2**-**P4**-**P4**^{N}	Expression plasmid containing **P1**-**P2**-**P4**-**P4**^{N}	This work
pCDF-**P1**-**P2**-**P5**-**P4**^{N}	Expression plasmid containing **P1**-**P2**-**P5**-**P4**^{N}	This work
pCDF-**P1**-**P2**-**P5**-**P4**^{N}	Expression plasmid containing **P1**-**P2**-**P5**-**P4**^{N}	This work
pCDF-**P1**-**P2**-**P6**-**P4**^{N}	Expression plasmid containing **P1**-**P2**-**P6**-**P4**^{N}	This work
pCDF-**P1**-**E2**-**P3**-**P4**^{N}	Expression plasmid containing **P1**-**E2**-**P3**-**P4**^{N}	This work
pCDF-**P1**-**S2**-**P3**-**P4**^{N}	Expression plasmid containing **P1**-**S2**-**P3**-**P4**^{N}	This work
pCDF-**P1**-**R6**-**P3**-**P4**^{N}	Expression plasmid containing **P1**-**R6**-**P3**-**P4**^{N}	This work
pCDF- **P1**-**P2**-**R5**-**P4**^{N}	Expression plasmid containing **P1**-**P2**-**R5**-**P4**^{N}	This work
pET28-^{C}**P4**-**P7**	Expression plasmid containing ^{C}**P4**-**P7**	This work
pET28-^{C}**P4**-**P4**-**P7**	Expression plasmid containing ^{C}**P4**-**P4**-**P7**	This work
pET28-^{C}**P4**-**P5**-**P7**	Expression plasmid containing ^{C}**P4**-**P5**-**P7**	This work
pET28-^{C}**P4**-**P5**[**P3**DD]-**P7**	Expression plasmid containing ^{C}**P4**-**P5**[**P3**DD]-**P7**	This work
pET28-^{C}**P4**-**P6**-**P7**	Expression plasmid containing ^{C}**P4**-**P6**-**P7**	This work
pET28-^{C}**P4**-**P6**[**P6**DD]-**P7**	Expression plasmid containing ^{C}**P4**-**P6**[**P6**DD]-**P7**	This work
pET28-^{C}**P4**-**P5**-**P6**-**P7**	Expression plasmid containing ^{C}**P4**-**P5**-**P6**-**P7**	This work

pET28-^{C}**P4**-**P5**[**P5**DD]-**P6**-**P7**	Expression plasmid containing ^{C}**P4**-**P5**[**P5**DD]-**P6**-**P7**	This work
pET28-^{C}**P4**-**P5**-**P6**[**P6**DD]-**P7**	Expression plasmid containing ^{C}**P4**-**P5**-**P6**[**P6**DD]-**P7**	This work
pET28-^{C}**P4**-**P5**[**P5**DD]-**P6**[**P6**DD]-**P7**	Expression plasmid containing ^{C}**P4**-**P5**[**P5**DD]-**P6**[**P6**DD]-**P7**	This work
pET28-^{C}**P4**-**P2**-**P6**-**P7**	Expression plasmid containing ^{C}**P4**-**P2**-**P6**-**P7**	This work
pET28-^{C}**P4**-**P3**-**P6**-**P7**	Expression plasmid containing ^{C}**P4**-**P3**-**P6**-**P7**	This work
pET28-^{C}**P4**-**P4**-**P6**-**P7**	Expression plasmid containing ^{C}**P4**-**P4**-**P6**-**P7**	This work
pET28-^{C}**P4**-**P6**-**P6**-**P7**	Expression plasmid containing ^{C}**P4**-**P6**-**P6**-**P7**	This work
pET28-^{C}**P4**-**P5**-**P2**-**P7**	Expression plasmid containing ^{C}**P4**-**P5**-**P2**-**P7**	This work
pET28-^{C}**P4**-**P5**-**P3**-**P7**	Expression plasmid containing ^{C}**P4**-**P5**-**P3**-**P7**	This work
pET28-^{C}**P4**-**P5**-**P4**-**P7**	Expression plasmid containing ^{C}**P4**-**P5**-**P4**-**P7**	This work
pET28-^{C}**P4**-**P5**-**P5**-**P7**	Expression plasmid containing ^{C}**P4**-**P5**-**P5**-**P7**	This work
pET28-^{C}**P4**-**P5**-**E6**-**P7**	Expression plasmid containing ^{C}**P4**-**P5**-**E6**-**P7**	This work
pET28- ^{C}**P4**-**P5**[**A1**DD]-**P5**-**P6**-**P7**	Expression plasmid containing ^{C}**P4**-**P5**[**A1**DD]-**P5**-**P6**-**P7**	This work
pET28-^{C}**P4**-**P5**-**P6***-**P7**	Expression plasmid containing ^{C}**P4**-**P5**-**P6***-**P7**	This work
pDes	pACYC-derived expression plasmid containing *desI*, *desII*, *desV*, *desVI*, *desVII*, *desVIII*, *ermE*	
pDesPikC	pACYC-derived expression plasmid containing *desI*, *desII*, *desV*, *desVI*, *desVII*, *desVIII*, *ermE*, and *pikC*	This work
pPikC	pET21 expression plasmid containing *pikC*	This work
pDesPikAV	pACYC-derived expression plasmid containing *desI*, *desII*, *desV*, *desVI*, *desVII*, *desVIII*, *ermE*, and *pikAV*	This work
pMKBAC02-Des	pMKBAC02-derived expression BAC containing *desI*, *desII*, *desV*, *desVI*, *desVII*, *desVIII*, *ermE*	This work
pACYC-pikAV	Expression of plasmid containing *pikAV*	This work
pACYC-T7prom(-5G)-pikAV	37% relative expression of pACYC-pikAV	This work
pACYC-T7prom(-13T)-pikAV	57% relative expression of pACYC-pikAV	This work
pACYC-T7prom(-16T)-pikAV	72% relative expression of pACYC-pikAV	This work
pACYC-pikAV,pikAV	200% relative expression of pACYC-pikAV	This work
pACYC-pikAV, T7prom(-5G)-pikAV	137% relative expression of pACYC-pikAV	This work
pACYC-pikAV, T7prom(-13T)-pikAV	157% relative expression of pACYC-pikAV	This work
pACYC-pikAV, T7prom(-16T)-pikAV	172% relative expression of pACYC-pikAV	This work
pEcgRNA-cadA	pEcgRNA sgRNA-*cadA*	This work
pEcgRNA-∆cadA	pEcgRNA-*cadA* having 1000 bp homology arms upstream and downstream of an XbaI site	This work
pEcgRNA-∆cadA: desI ,II, V, VI, VII, VIII, ermE	pEcgRNA-∆*cadA:: desI, desII, desV, desVI, desVII, desVIII, ermE*	This work
pEcgRNA-rmlC	pEcgRNA sgRNA-*rmlC*	This work
pEcgRNA-vioAB	pEcgRNA sgRNA-*vioAB*	This work

pEcgRNA-wecDE	pEcgRNA sgRNA-*wecDE*	This work

Table S3. Crystal data and structure refinement for **12**.

Identification code	IR634567
Empirical formula	$C_{19}H_{30}O_5$
Formula weight	338.43
Temperature/K	100
Crystal system	orthorhombic
Space group	$P2_12_12_1$
a/Å	5.9917(3)
b/Å	11.8521(6)
c/Å	26.5142(14)
α/°	90
β/°	90
γ/°	90
Volume/Å^3	1882.89(17)
Z	4
ρ_{calc}g/cm^3	1.194
μ/mm^{-1}	0.690
F(000)	736.0
Crystal size/mm^3	0.23 × 0.05 × 0.02
Radiation	Cu Kα (λ = 1.54184)
2Θ range for data collection/°	6.668 to 158.228
Index ranges	-6 ≤ h ≤ 7, -14 ≤ k ≤ 13, -33 ≤ l ≤ 33
Reflections collected	17112
Independent reflections	3854 [R_{int} = 0.0855, R_{sigma} = 0.0577]
Data/restraints/parameters	3854/0/226
Goodness-of-fit on F^2	1.107
Final R indexes [I>=2σ (I)]	R_1 = 0.0673, wR_2 = 0.1594
Final R indexes [all data]	R_1 = 0.0832, wR_2 = 0.1760
Largest diff. peak/hole / e Å^{-3}	0.42/-0.37
Flack parameter	-0.08(17)

Table S4. Summary of polyketide production. A calibration curve generated using narbonolide (EIC peak areas) was employed for macrolactones, while a calibration curve generated using pikromycin (EIC peak areas) was employed for desosaminylated macrolides (Figs. S70–71). A calibration curve generated using the purified product of **P1**-**P5**-**P6**-**P7**[10] was employed for δ-lactone products (UV peak areas at 247 nm), while a calibration curve generated using 4-hydroxy-6-methy-2-pyrone (purchased from Sigma-Aldrich) was employed for pyrone products (UV peak areas at 285 nm) (Figs. S72–73). Data are mean ± s.d., n = 3 independent experiments (Supplementary Data 8).

Strain	Product	Productivity (mgL^{-1})
K207-3 + **P1**-**P4**-**P7**	**7**	28 ± 2
K207-3 + **P1**-**P2**-**P3**-**P4**-**P7**	**8**	1.6 ± 0.1
K207-3 + **P1**-**P4**-**P5**-**P6**-**P7**	**9**	18 ± 1
K207-3 + **P1**-**P2**-**P3**-**P4**-**P5**-**P6**-**P7**	**1**	23 ± 2
K207-3 + **P1**-**P2**-**P3**[**P3**DD]-**P4**-**P5**[**P5**DD]-**P6**[**P6**DD]-**P7**	**1**	30 ± 2
K207-3 + **P1**-**P2**-**P3**-**P4**-**P5**-**P6**-**P7**+pDesPikAV	**1**	44 ± 2
K207-3 + **P1**-**P2**-**P3**[**P3**DD]-**P4**-**P5**[**P5**DD]-**P6**[**P6**DD]-**P7**+pDesPikAV	**1**	49 ± 4
K207-3 + **P1**-**P2**-**P5**-**P4**-**P5**-**P6**-**P7**	**10**	3.5 ± 0.5
K207-3 + **P1**-**P2**-**P5**-**P4**-**P5**-**P6**-**P7**	**11**	1.2 ± 0.1
K207-3 + **P1**-**E2**-**P3**-**P4**-**P5**-**P6**-**P7**	**1**	0.10 ± 0.05
K207-3 + **P1**-**P2**-**P3**-**P4**-**P5**-**E6**-**P7**	**1**	18 ± 2
K207-3 + **P1**-**R6**-**P3**-**P4**-**P5**-**P6**-**P7**	**12**	4.6 ± 1.9
K207-3 + **P1**-**P2**-**R5**-**P4**-**P5**-**P6**-**P7**	**13**	1.9 ± 1.0
K207-3 + **P1**-**P2**-**R5**-**P4**-**P5**-**P6**-**P7**	**14**	4.4 ± 0.0
K207-3 + **P1**-**P2**-**P3**-**P4**-**P5**-**P6***-**P7**	**4**	28 ± 4.7
K207-3 + **P1**-**P2**-**P5**-**P4**-**P5**-**P6***-**P7**	**15**	4.8 ± 1.4
K207-3 + **P1**-**R6**-**P3**-**P4**-**P5**-**P6***-**P7**	**16**	trace
K207-3 + **P1**-**P2**-**R5**-**P4**-**P5**-**P6***-**P7**	**17**	12.4 ± 3.2
K207-3 + **P1**-**P2**-**P4**-**P5**-**P6**-**P7**	**18**	32 ± 1
K207-3 + **P1**-**P2**-**P3**-**P4**-**P5**-**P5**-**P6**-**P7**	**1**	7.1 ± 1.0
K207-3 + **P1**-**P2**-**P3**-**P4**-**P5**-**P5**-**P6**-**P7**	**20**	1.9 ± 0.1
K207-3 + **P1**-**P2**-**P3**-**P4**-**P5**-**P6**-**P7** + pDes	**2**	1.1 ± 0.2
K207-3 + **P1**-**P2**-**P3**-**P4**-**P5**-**P6**-**P7** + pDesPikC	**3**	0.45 ± 0.02
K207-3 + **P1**-**P2**-**P3**-**P4**-**P5**-**P6**-**P7** + pDesPikC + pPikC	**3**	0.58 ± 0.02
K207-3 + **P1**-**P2**-**P3**-**P4**-**P5**-**P6***-**P7** + pDes	**5**	0.63 ± 0.04
K207-3 + **P1**-**P2**-**P3**-**P4**-**P5**-**P6***-**P7** + pDesPikC	**6**	0.27 ± 0.05
K207-3 + **P1**-**P2**-**P5**-**P4**-**P5**-**P6**-**P7** + pDes	**21**	0.87 ± 0.20
K207-3 + **P1**-**R6**-**P3**-**P4**-**P5**-**P6**-**P7** + pDes	**22**	0.0032 ± 0.0014

K207-3 + **P1**-**R6**-**P3**-**P4**-**P5**-**P6***-**P7** + pDes	**24**	0.0014 ± 0.0002
K207-3 + **P1**-**P2**-**R5**-**P4**-**P5**-**P6**-**P7** + pDes	**23**	0.0012 ± 0.0001
K207-3 + **P1**-**P2**-**R5**-**P4**-**P5**-**P6***-**P7** + pDes	**25**	0.0016 ± 0.0001
K207-3 + **P1**-**P2**-**P3**-**P4**-**P5**-**P6**-**P7** + pMKBAC02-Des operon	**2**	0.73 ± 0.16
TM1 + **P1**-**P2**-**P3**-**P4**-**P5**-**P6**-**P7**	**2**	0.63 ± 0.12
TM4 + **P1**-**P2**-**P3**-**P4**-**P5**-**P6**-**P7** + pDes	**2**	12 ± 2
TM7 + **P1**-**P2**-**P3**-**P4**-**P5**-**P6**-**P7**	**2**	8.9 ± 1.7
TM7 + **P1**-**P2**-**P3**-**P4**-**P5**-**P6**-**P7** + pDes	**2**	20 ± 2
TM7 + **P1**-**P2**-**P3**-**P4**-**P5**-**P6**-**P7** + pDesPikAV	**2**	31 ± 4
TM7 +**P1**-**P2**-**P3**[**P3**DD]-**P4**-**P5**[**P5**DD]-**P6**[**P6**DD]-**P7**+ pDesPikAV	**2**	37 ± 3
TM7 + **P1**-**P2**-**P3**-**P4**-**P5**-**P6***-**P7** + pDesPikAV	**5**	13 ± 2.6
TM7 + **P1**-**P2**-**P5**-**P4**-**P5**-**P6**-**P7** + pDesPikAV	**21**	2.7 ± 0.3
TM7 + **P1**-**R6**-**P3**-**P4**-**P5**-**P6**-**P7** + pDesPikAV	**22**	1.2 ± 0.2
TM7 + **P1**-**P2**-**R5**-**P4**-**P5**-**P6**-**P7** + pDesPikAV	**23**	1.8 ± 0.1
TM7 + **P1**-**R6**-**P3**-**P4**-**P5**-**P6***-**P7** + pDesPikAV	**24**	0.50 ± 0.02
TM7 + **P1**-**P2**-**R5**-**P4**-**P5**-**P6***-**P7** + pDesPikAV	**25**	0.31 ± 0.05
TM7 + **P1**-**R6**-**P3**-**P4**-**P5**-**P6**-**P7**	**22**	1.0 ± 0.1
TM7 + **P1**-**P2**-**R5**-**P4**-**P5**-**P6**-**P7**	**23**	1.5 ± 0.2
TM7 + **P1**-**R6**-**P3**-**P4**-**P5**-**P6***-**P7**	**24**	0.32 ± 0.05
TM7 + **P1**-**P2**-**R5**-**P4**-**P5**-**P6***-**P7**	**25**	0.24 ± 0.06

REFERENCES

1. Motohashi, K. Seamless Ligation Cloning Extract (SLiCE) Method Using Cell Lysates from Laboratory Escherichia coli Strains and its Application to SLiP Site-Directed Mutagenesis. *Methods Mol Biol* **1498**, 349-357 (2017).
2. Ray, K.A. et al. Assessing and harnessing updated polyketide synthase modules through combinatorial engineering. *Nat Commun* **15**, 6485 (2024).
3. Zadeh, S.M. et al. beta-Hydroxylation of alpha-amino-beta-hydroxylbutanoyl-glycyluridine catalyzed by a nonheme hydroxylase ensures the maturation of caprazamycin. *Commun Chem* **5**, 87 (2022).
4. Jiang, Y. et al. Multigene editing in the Escherichia coli genome via the CRISPR-Cas9 system. *Appl Environ Microbiol* **81**, 2506-14 (2015).
5. Li, Q. et al. A modified pCas/pTargetF system for CRISPR-Cas9-assisted genome editing in Escherichia coli. *Acta Biochim Biophys Sin (Shanghai)* **53**, 620-627 (2021).
6. Miyazawa, T., Fitzgerald, B.J. & Keatinge-Clay, A.T. Preparative production of an enantiomeric pair by engineered polyketide synthases. *Chem Commun (Camb)* **57**, 8762-8765 (2021).
7. Imburgio, D., Rong, M., Ma, K. & McAllister, W.T. Studies of promoter recognition and start site selection by T7 RNA polymerase using a comprehensive collection of promoter variants. *Biochemistry* **39**, 10419-30 (2000).
8. Gupta, S., Lakshmanan, V., Kim, B.S., Fecik, R. & Reynolds, K.A. Generation of novel pikromycin antibiotic products through mutasynthesis. *Chembiochem* **9**, 1609-16 (2008).
9. Shinde, P.B. et al. Combinatorial biosynthesis and antibacterial evaluation of glycosylated derivatives of 12-membered macrolide antibiotic YC-17. *J Biotechnol* **168**, 142-8 (2013).
10. Zhang, J., Bista, R., Miyazawa, T. & Keatinge-Clay, A.T. Boosting titers of engineered triketide and tetraketide synthases to record levels through T7 promoter tuning. *Metab Eng* **78**, 93-98 (2023).
11. Murli, S., Kennedy, J., Dayem, L.C., Carney, J.R. & Kealey, J.T. Metabolic engineering of Escherichia coli for improved 6-deoxyerythronolide B production. *J Ind Microbiol Biotechnol* **30**, 500-9 (2003).
